# MEMOTE for standardized genome-scale metabolic model testing

**DOI:** 10.1038/s41587-020-0446-y

**Published:** 2020-03-02

**Authors:** Christian Lieven, Moritz E. Beber, Brett G. Olivier, Frank T. Bergmann, Meric Ataman, Parizad Babaei, Jennifer A. Bartell, Lars M. Blank, Siddharth Chauhan, Kevin Correia, Christian Diener, Andreas Dräger, Birgitta E. Ebert, Janaka N. Edirisinghe, José P. Faria, Adam M. Feist, Georgios Fengos, Ronan M. T. Fleming, Beatriz García-Jiménez, Vassily Hatzimanikatis, Wout van Helvoirt, Christopher S. Henry, Henning Hermjakob, Markus J. Herrgård, Ali Kaafarani, Hyun Uk Kim, Zachary King, Steffen Klamt, Edda Klipp, Jasper J. Koehorst, Matthias König, Meiyappan Lakshmanan, Dong-Yup Lee, Sang Yup Lee, Sunjae Lee, Nathan E. Lewis, Filipe Liu, Hongwu Ma, Daniel Machado, Radhakrishnan Mahadevan, Paulo Maia, Adil Mardinoglu, Gregory L. Medlock, Jonathan M. Monk, Jens Nielsen, Lars Keld Nielsen, Juan Nogales, Intawat Nookaew, Bernhard O. Palsson, Jason A. Papin, Kiran R. Patil, Mark Poolman, Nathan D. Price, Osbaldo Resendis-Antonio, Anne Richelle, Isabel Rocha, Benjamín J. Sánchez, Peter J. Schaap, Rahuman S. Malik Sheriff, Saeed Shoaie, Nikolaus Sonnenschein, Bas Teusink, Paulo Vilaça, Jon Olav Vik, Judith A. H. Wodke, Joana C. Xavier, Qianqian Yuan, Maksim Zakhartsev, Cheng Zhang

**Affiliations:** 1 Novo Nordisk Foundation Center for Biosustainability, Technical University of Denmark, Lyngby, Denmark; 2 Systems Biology Lab, Amsterdam Institute of Molecular and Life Sciences (AIMMS), Vrije Universiteit Amsterdam, Amsterdam, the Netherlands; 3 BioQUANT/COS, Heidelberg University, Heidelberg, Germany; 4 Ecole Polytechnique Fédérale de Lausanne, Laboratory of Computational Systems Biotechnology, Lausanne, Switzerland; 5 iAMB-Institute of Applied Microbiology, ABBt-Aachen Biology and Biotechnology, RWTH Aachen University, Aachen, Germany; 6 Department of Bioengineering, University of California, La Jolla, CA USA; 7 Department of Chemical Engineering and Applied Chemistry, University of Toronto, Toronto, Ontario Canada; 8 Human Systems Biology Laboratory, Instituto Nacional de Medicina Genomica & Coordinación de la Investigación Científica-Red de Apoyo a la Investigación, UNAM, Mexico City, Mexico; 9 Institute for Systems Biology, Seattle, WA USA; 10 Computational Systems Biology of Infection and Antimicrobial-Resistant Pathogens, Institute for Biomedical Informatics (IBMI), Tübingen, Germany; 11 Department of Computer Science, University of Tübingen, Tübingen, Germany; 12 German Center for Infection Research (DZIF), partner site Tübingen, Tübingen, Germany; 13 Australian Institute for Bioengineering and Nanotechnology, The University of Queensland, Brisbane, Queensland Australia; 14 Argonne National Laboratory, Lemont, IL USA; 15 Analytical Biosciences, Division of Systems Biomedicine and Pharmacology, Leiden Academic Centre for Drug Research, Leiden University, Leiden, the Netherlands; 16 Department of Systems Biology, Centro Nacional de Biotecnología, Consejo Superior de Investigaciones Científicas (CNB-CSIC), Madrid, Spain; 17 Department of Animal and Aquacultural Sciences, Faculty of Biosciences, Norwegian University of Life Sciences, Oslo, Norway; 18 Hanze University of Applied Sciences, Groningen, the Netherlands; 19 European Bioinformatics Institute, European Molecular Biology Laboratory (EMBL-EBI), Wellcome Trust Genome Campus, Cambridge, UK; 20 Department of Chemical and Biomolecular Engineering (BK21 Plus Program), BioProcess Engineering Research Center, BioInformatics Research Center, Institute for the BioCentury, Korea Advanced Institute of Science and Technology (KAIST), Daejeon, Korea; 21 Analysis and Redesign of Biological Networks, Max Planck Institute for Dynamics of Complex Technical Systems Magdeburg, Magdeburg, Germany; 22 Theoretical Biophysics, Humboldt-Universität zu Berlin, Berlin, Germany; 23 Department of Agrotechnology and Food Sciences, Laboratory of Systems and Synthetic Biology, Wageningen University & Research, Wageningen, the Netherlands; 24 Bioprocessing Technology Institute, Agency for Science, Technology and Research (A*STAR), Singapore, Singapore; 25 School of Chemical Engineering Sungkyunkwan University, Jangan-gu Suwon, Gyeonggi-do Republic of Korea; 26 Science for Life Laboratory, KTH - Royal Institute of Technology, Stockholm, Sweden; 27 Centre for Host-Microbiome Interactions, Faculty of Dentistry, Oral & Craniofacial Sciences, King’s College London, London, UK; 28 Department of Pediatrics and Novo Nordisk Foundation Center for Biosustainability, University of California, San Diego School of Medicine, La Jolla, CA USA; 29 Key Laboratory of Systems Microbial Biotechnology, Tianjin Institute of Industrial Biotechnology, Chinese Academy of Sciences, Tianjin, P.R. China; 30 Structural and Computational Biology Unit, European Molecular Biology Laboratory (EMBL), Heidelberg, Germany; 31 Institute of Biomaterials and Biomedical Engineering, University of Toronto, Toronto, Canada; 32 SilicoLife Lda., Braga, Portugal; 33 Department of Biomedical Engineering, University of Virginia, Charlottesville, Virginia USA; 34 Chalmers University of Technology, Department of Biology and Biological Engineering, Division of Systems and Synthetic Biology, Göteborg, Sweden; 35 Department of Biomedical Informatics, College of Medicine, University of Arkansas for Medical Sciences (UAMS), Little Rock, AR USA; 36 Oxford Brookes University, Oxford, UK; 37 Centre of Biological Engineering, University of Minho, Braga, Portugal; 38 Instituto de Tecnologia Química e Biológica António Xavier, Universidade Nova de Lisboa (ITQB-NOVA), Oeiras, Portugal; 39 Institute for Molecular Evolution, Heinrich-Heine-University Düsseldorf, Düsseldorf, Germany; 40 Present Address: Centro de Biotecnología y Genómica de Plantas (CBGP, UPM-INIA), Universidad Politécnica de Madrid (UPM) – Instituto Nacional de Investigación y Tecnología Agraria y Alimentaria (INIA), Madrid, Spain

**Keywords:** Computational models, Software, Biochemical networks

**To the Editor** — Reconstructing metabolic reaction networks enables the development of testable hypotheses of an organism’s metabolism under different conditions^1^. State-of-the-art genome-scale metabolic models (GEMs) can include thousands of metabolites and reactions that are assigned to subcellular locations. Gene–protein–reaction (GPR) rules and annotations using database information can add meta-information to GEMs. GEMs with metadata can be built using standard reconstruction protocols^2^, and guidelines have been put in place for tracking provenance and enabling interoperability, but a standardized means of quality control for GEMs is lacking^3^. Here we report a community effort to develop a test suite named MEMOTE (for metabolic model tests) to assess GEM quality.

Incompatible description formats and missing annotations^4^ limit GEM reuse. Moreover, numerical errors^5^ and omission of essential cofactors^6^ in a single biomass objective function can have substantial impact on the predictive performance of a GEM. Failure to make checks for flux cycles and imbalances can render model predictions untrustworthy^7^.

Every year, increasing numbers of manually curated and automatically generated GEMs are published, including those for human and cancer tissue models^8^. We believe that it is essential to optimize GEM reproducibility and reuse. Researchers need models that are software-agnostic, with components that have standardized, database-independent identifiers. Default conditions and mathematically specified modeling formulations must be precisely defined to allow reproduction of the original model predictions. Models must produce feasible phenotypes under various conditions. Finally, data used to build any model must be made available in a reusable format.

A dual approach could be used to improve GEM reuse and reproducibility. First, we advocate adoption of the latest version of the Systems Biology Markup Language (SBML) level 3 flux balance constraints (SBML3FBC) package^9^ as the primary description and exchange format. The SBML3FBC package adds structured, semantic descriptions for domain-specific model components such as flux bounds, multiple linear objective functions, GPR rules, metabolite chemical formulas, charge and annotations. The SBML and constraint-based modeling communities collaboratively develop this package, updating it based on user input. It has been adopted by a wide range of constraint-based modeling software and public model repositories (http://cbmpy.sourceforge.net/ and refs. ^10–15^), and should therefore be considered the standard for encoding GEMs.

Second, we present MEMOTE (/’mi:moʊt/ in international phonetic alphabet notation), an open-source Python software that represents a unified approach to ensure the formally correct definition of SBML3FBC and provides quality control and continuous quality assurance of metabolic models with tools and best practices already used in software development^16,17^. MEMOTE accepts stoichiometric models encoded in SBML3FBC and previous versions as input. In addition to structural validation analogous to the SBML validator^18^, MEMOTE benchmarks metabolic models using consensus tests from four general areas: annotation, basic tests, biomass reaction and stoichiometry.

Annotation tests check that a model is annotated according to community standards with minimum information required in annotation of models (MIRIAM)-compliant cross-references^19^, that all primary identifiers belong to the same namespace rather than being fractured across several namespaces, and that components are described using Systems Biology Ontology (SBO) terms^20^. A lack of explicit, standardized annotations complicates the use, comparison and extension of GEMs, and thus strongly hampers collaboration^3,4^.

Basic tests check the formal correctness of a model and verify the presence of components such as metabolites, compartments, reactions and genes. These tests also check for metabolite formula and charge information, and GPR rules. General quality metrics, such as the degree of metabolic coverage representing the ratio of reactions and genes^21^, are also checked.

A model is tested for production of biomass precursors in different conditions, for biomass consistency, for nonzero growth rate and for direct precursors. The biomass reaction is based on the biomass composition of the modeled organism and expresses its ability to produce the necessary precursors for in silico cell growth and maintenance. Thus, an extensive, well-formed biomass reaction is crucial for accurate predictions with a GEM^6^.

Stoichiometric inconsistency, erroneously produced energy metabolites^7^ and permanently blocked reactions are identified by MEMOTE. Errors in stoichiometries may result in the production of ATP or redox cofactors from nothing^2^ and are detrimental to the performance of the model when using flux-based analysis^4^.

MEMOTE enables a quick comparison of any two given models, in which individual test results are quantified and condensed to calculate an overall score (Supplementary Note 1). In addition to these consensus tests, researchers can supply experimental data from growth and gene perturbation studies in a range of input formats (.csv, .tsv, .xls or .xslx) in MEMOTE. To support reproducibility, researchers can configure MEMOTE to recognize specific data types as input to predefined experimental tests for model validation (Supplementary Note 2).

There are two main workflows for MEMOTE (Fig. 1a and Supplementary Figs. 1–3). For peer review, MEMOTE can produce either a ‘snapshot report’ or a ‘diff report’ that display MEMOTE test results of one single or multiple models, respectively. For model reconstruction, MEMOTE helps users to create a version-controlled repository of the model and to activate continuous integration toward building a ‘history report’ that records the results of each tracked edit of the model. Although a model repository can be used offline, we encourage community collaboration via distributed version control development platforms, such as GitHub (https://github.com), GitLab (https://gitlab.com/) or BioModels^12^ (http://wwwdev.ebi.ac.uk/biomodels/). MEMOTE is tightly integrated with GitHub. Models generated and versioned in MEMOTE can easily be uploaded to GitLab and BioModels. Collaborative model reconstruction with MEMOTE as benchmark can occur using all three software platforms (Fig. 1b).

**Fig. 1:**
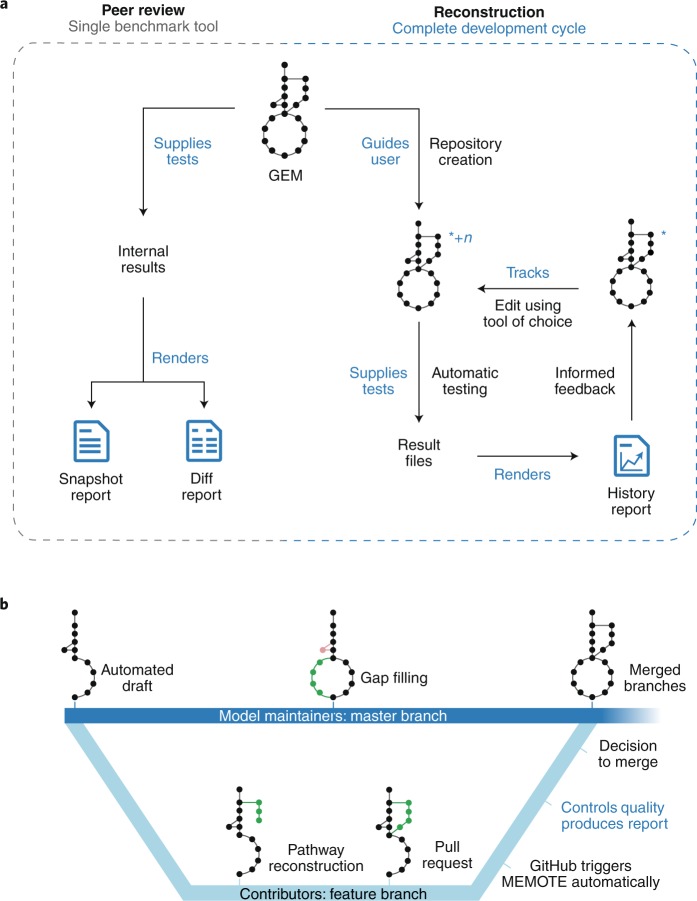
Graphical summary of MEMOTE. **a**, Graphical representation of the two principal workflows in detail. For peer review, MEMOTE serves as a benchmark tool generating a comprehensive, human-readable report, which quantifies the model’s performance (Supplementary Figs. 1 and 2). With this information, a definitive assessment of model quality can be made by editors, reviewers and users. This workflow is accessible through a web interface (https://memote.io) or locally through a command line interface. For model reconstruction, MEMOTE helps users to create a version-controlled repository for the model (indicated by the blue asterisk), and to activate continuous integration. The model is tested using MEMOTE’s library of test cases, the results are saved, and an initial report of the model is generated. This constitutes the first iteration of the development cycle. Now, users may edit the model using their preferred reconstruction tool and subsequently export it to SBML3FBC, thus creating a new version (indicated by +*n*). This will restart the cycle by running the tests automatically, saving the results for each version and including them incrementally in a report on the entire history of results. This serves as a guide toward a functional, high-quality GEM (Supplementary Fig. 3). This workflow is accessible through the command line only. **b**, Both, GitHub and GitLab support a branching strategy, which model builders could use to curate different parts of the model simultaneously or to invite external experts to improve specific model features. MEMOTE further enables model authors to act as gatekeepers, choosing to accept only high-quality contributions. Identification of functional differences happens in the form of a comparative ‘diff’ report, whereas for file-based discrepancies MEMOTE capitalizes on the platform’s ability to show the line-by-line changes between different versions of a model. For this purpose, the model is written in a sorted YAML format^28^ after every change. Bold blue text denotes actions performed by MEMOTE.

We validated MEMOTE using models from seven GEM collections (Fig. 2, Supplementary Table 1 and Supplementary Methods), that comprise manually and (semi)-automatically reconstructed GEMs (10,780 models in total). Most GEM collections have already made models available in SMBL format. A nonlinear dimensional reduction of the normalized test results (Supplementary Methods) using *t*-distributed stochastic neighbor embedding (*t*-SNE; Fig. 2a) indicates that models from the same source are generally more similar to each other than to models from other sources. Nevertheless, several model sources reveal internal subgroupings (Fig. 2a). With the exception of Path2Models^22^, which relies on pathway resources that contain problematic reaction information on stoichiometry and directionality^23^, automatically reconstructed GEMs were stoichiometrically consistent (Fig. 2b) and mass-balanced (Supplementary Fig. 4). Of the manually reconstructed GEMs we tested, most models in BiGG^13^ are stoichiometrically consistent, but there is wide variation among published models, with ~70% of models having at least one stoichiometrically unbalanced metabolite. Stoichiometrically inconsistent models cannot be mass-balanced, but missing formula annotations, from which molecular masses are calculated, further contribute to reactions being counted as unbalanced. The problems that we identified in published models underpin the need for application of MEMOTE during peer-review process (but ideally before submission) of GEMs.

**Fig. 2:**
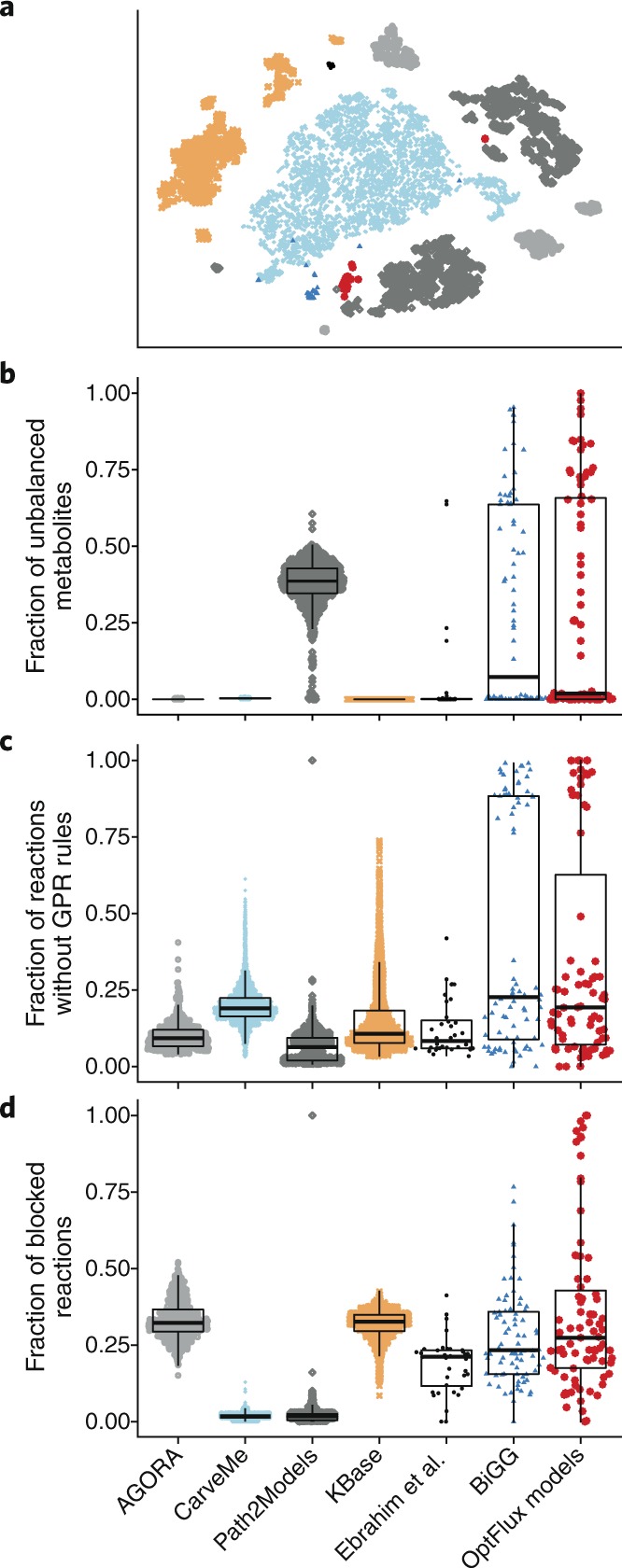
Quality of manually reconstructed GEMs from collections without quality control or quality assurance. **a**, Depicted is a *t*-SNE two-dimensional reduction of models using normalized test features as input. Only GEMs from the BiGG collection form a single albeit small cluster. Models from all other collections are grouped in several fragmented but distinct clusters. **b**–**d**, SinaPlots^29^ of each collection overlaid with box and whisker plots to indicate 25%, 50% (median) and 75% quantiles. GEMs from collections built in a modern automated pipeline (AGORA, CarveMe, KBase) are stoichiometrically consistent, whereas models from the older Path2Models collection are up to 50% stoichiometrically inconsistent (**b**). Manually reconstructed models (BiGG, Ebrahim et al.^30^, OptFlux models) contain varying degrees of inconsistent GEMs. GPR rules are essential for in silico knockout studies, but also serve to justify the presence of a reaction (**c**). Generally, the fraction of reactions without GPR rules is low (~15%). Yet a distinct group of models from the collections of Ebrahim et al. and OptFlux lack GPR rules for >75% of their reactions. Most models from the CarveMe and Path2Models collections contain very few blocked reactions, whereas for models from the other collections the number of blocked reactions lies mostly between 10% and 30% (**d**). Again, models from the collections of Ebrahim et al. and the Optflux models show the largest variance. Source Data

During GEM reconstruction, metabolic reactions are defined based on functional gene annotations, and this information is output as GPR rules. We found that ~15% of reactions in models we tested are not annotated with GPR rules (Fig. 2c). For published models, subgroups of models contain up to 85% of reactions without GPR rules. This could be due to a large number of modeling-specific reactions, spontaneous reactions^24^ and known reactions with undiscovered genes, or if GPR rules were annotated in nonstandard ways.

CarveMe^25^ and Path2Models^22^ have a very low fraction of universally blocked reactions, whereas models from AGORA^26^ and KBase^14^ contain ~30% blocked reactions, and BiGG^13^ models and OptFlux^15^ models contain ~20% blocked reactions (Fig. 2d). Similarly, orphan and dead-end metabolites (Supplementary Figs. 5 and 6) are also present in all of these published collections. We note that blocked reactions and dead-end metabolites are not indicators of low-quality models but that a large proportion (for example, >50%) of universally blocked reactions can indicate problems in reconstruction that need solving.

AGORA, KBase and BiGG are the only collections with SBML-compliant metabolite and reaction annotations. Gene annotations are only present in KBase models and selected BiGG models (Supplementary Figs. 7–9). Each collection uses its own system of identifiers for each model component, but there is some overlap between all three (Supplementary Figs. 10 and 11), and partial overlaps for models from KBase and BiGG (Supplementary Figs. 12–16), or AGORA and BiGG (Supplementary Figs. 17 and 18), but not KBase and AGORA. BiGG is the only collection with models using MetaNetX^27^ annotations (Supplementary Fig. 19). MetaNetX consolidates biochemical namespaces by establishing a mapping between them through a set of unique identifiers. Hence, knowing the MetaNetX identifier for a given entity often means also knowing the identifiers for other databases (Supplementary Methods).

MEMOTE tests cover semantic and conceptual requirements, which are fundamental to SBML3FBC and constraint-based modeling, respectively. They are extensible to allow the validation of a model’s performance against experimental data and can be executed as a stand-alone tool or integrated into existing reconstruction pipelines. Capitalizing on robust workflows established in modern software development, MEMOTE promotes openness and collaboration by granting the community tangible metrics to support their research and to discuss assumptions or limitations openly.

Application of a set of defined metabolic model tests is not dependent on implementation in MEMOTE, and for some users it may be more desirable to implement each test separately to streamline the user experience.

We propose that an independent, central library of tests and a tool to run them offers an unbiased approach to quality control because the tests are continuously reviewed by the community. This resource will be maintained under stewardship of Nikolaus Sonnenschein by the openCOBRA consortium (https://github.com/opencobra). To encourage integration as opposed to duplication, MEMOTE provides a Python application programing interface (API) as well as being available as a web service. MEMOTE has already been integrated in several services and tools (Supplementary Note 3). We discuss alternatives and future perspectives of MEMOTE in Supplementary Notes 4 and 5, respectively.

We recommend that MEMOTE users reach out to GEM authors to report any errors and thereby enable community improvement of models as resources. Using inconsistent GEMs for hypothesis generation could lead researchers down blind alleys, so we weighed the influence of ‘consistency’ and ‘stoichiometric consistency’ and SBO terms higher than tests for metabolite, reaction and gene annotations.

We are committed to keeping MEMOTE open to support community principles. Robust benchmarking will only work if it is actively supported by the whole community, and we call on any interested experts to join this endeavor and enable its continual improvement.

## Reporting Summary

Further information on research design is available in the Nature Research Reporting Summary linked to this article.

## Supplementary information

Supplementary Materials Supplementary Figs. 1–162, Notes 1–5, Methods, and Tables 1 and 2

Reporting Summary

## Data Availability

The model collection is available at 10.5281/zenodo.2636858. Individual results and aggregated tables, as well as analysis code, are available at 10.5281/zenodo.2638234.

## Source data

Source Data Fig. 2

## Code availability

MEMOTE source code is available at https://github.com/opencobra/memote under the Apache license, version 2.0. Supporting documentation is available at https://memote.readthedocs.io/en/latest/. The MEMOTE web interface is hosted at https://memote.io. A detailed list of all tests in MEMOTE is available at https://memote.readthedocs.io/en/latest/autoapi/index.html.
