# Publisher Correction: MEMOTE for standardized genome-scale metabolic model testing

**DOI:** 10.1038/s41587-020-0477-4

**Published:** 2020-03-19

**Authors:** Christian Lieven, Moritz E. Beber, Brett G. Olivier, Frank T. Bergmann, Meric Ataman, Parizad Babaei, Jennifer A. Bartell, Lars M. Blank, Siddharth Chauhan, Kevin Correia, Christian Diener, Andreas Dräger, Birgitta E. Ebert, Janaka N. Edirisinghe, José P. Faria, Adam M. Feist, Georgios Fengos, Ronan M. T. Fleming, Beatriz García-Jiménez, Vassily Hatzimanikatis, Wout van Helvoirt, Christopher S. Henry, Henning Hermjakob, Markus J. Herrgård, Ali Kaafarani, Hyun Uk Kim, Zachary King, Steffen Klamt, Edda Klipp, Jasper J. Koehorst, Matthias König, Meiyappan Lakshmanan, Dong-Yup Lee, Sang Yup Lee, Sunjae Lee, Nathan E. Lewis, Filipe Liu, Hongwu Ma, Daniel Machado, Radhakrishnan Mahadevan, Paulo Maia, Adil Mardinoglu, Gregory L. Medlock, Jonathan M. Monk, Jens Nielsen, Lars Keld Nielsen, Juan Nogales, Intawat Nookaew, Bernhard O. Palsson, Jason A. Papin, Kiran R. Patil, Mark Poolman, Nathan D. Price, Osbaldo Resendis-Antonio, Anne Richelle, Isabel Rocha, Benjamín J. Sánchez, Peter J. Schaap, Rahuman S. Malik Sheriff, Saeed Shoaie, Nikolaus Sonnenschein, Bas Teusink, Paulo Vilaça, Jon Olav Vik, Judith A. H. Wodke, Joana C. Xavier, Qianqian Yuan, Maksim Zakhartsev, Cheng Zhang

**Affiliations:** 1 Novo Nordisk Foundation Center for Biosustainability, Technical University of Denmark, Lyngby, Denmark; 2 Systems Biology Lab, Amsterdam Institute of Molecular and Life Sciences (AIMMS), Vrije Universiteit Amsterdam, Amsterdam, the Netherlands; 3 BioQUANT/COS, Heidelberg University, Heidelberg, Germany; 4 Ecole Polytechnique Fédérale de Lausanne, Laboratory of Computational Systems Biotechnology, Lausanne, Switzerland; 5 iAMB-Institute of Applied Microbiology, ABBt-Aachen Biology and Biotechnology, RWTH Aachen University, Aachen, Germany; 6 Department of Bioengineering, University of California, La Jolla, CA USA; 7 Department of Chemical Engineering and Applied Chemistry, University of Toronto, Toronto, Ontario Canada; 8 Human Systems Biology Laboratory, Instituto Nacional de Medicina Genomica & Coordinación de la Investigación Científica-Red de Apoyo a la Investigación, UNAM, Mexico City, Mexico; 9 Institute for Systems Biology, Seattle, WA USA; 10 Computational Systems Biology of Infection and Antimicrobial-Resistant Pathogens, Institute for Biomedical Informatics (IBMI), Tübingen, Germany; 11 Department of Computer Science, University of Tübingen, Tübingen, Germany; 12 German Center for Infection Research (DZIF), partner site Tübingen, Tübingen, Germany; 13 Australian Institute for Bioengineering and Nanotechnology, The University of Queensland, Brisbane, Queensland Australia; 14 Argonne National Laboratory, Lemont, IL USA; 15 Analytical Biosciences, Division of Systems Biomedicine and Pharmacology, Leiden Academic Centre for Drug Research, Leiden University, Leiden, the Netherlands; 16 Department of Systems Biology, Centro Nacional de Biotecnología, Consejo Superior de Investigaciones Científicas (CNB-CSIC), Madrid, Spain; 17 Department of Animal and Aquacultural Sciences, Faculty of Biosciences, Norwegian University of Life Sciences, Oslo, Norway; 18 Hanze University of Applied Sciences, Groningen, the Netherlands; 19 European Bioinformatics Institute, European Molecular Biology Laboratory (EMBL-EBI), Wellcome Trust Genome Campus, Cambridge, UK; 20 Department of Chemical and Biomolecular Engineering (BK21 Plus Program), BioProcess Engineering Research Center, BioInformatics Research Center, Institute for the BioCentury, Korea Advanced Institute of Science and Technology (KAIST), Daejeon, Korea; 21 Analysis and Redesign of Biological Networks, Max Planck Institute for Dynamics of Complex Technical Systems Magdeburg, Magdeburg, Germany; 22 Theoretical Biophysics, Humboldt-Universität zu Berlin, Berlin, Germany; 23 Department of Agrotechnology and Food Sciences, Laboratory of Systems and Synthetic Biology, Wageningen University & Research, Wageningen, the Netherlands; 24 Bioprocessing Technology Institute, Agency for Science, Technology and Research (A*STAR), Singapore, Singapore; 25 School of Chemical Engineering Sungkyunkwan University, Jangan-gu Suwon, Gyeonggi-do Republic of Korea; 26 Science for Life Laboratory, KTH -Royal Institute of Technology, Stockholm, Sweden; 27 Centre for Host-Microbiome Interactions, Faculty of Dentistry, Oral & Craniofacial Sciences, King’s College London, London, UK; 28 Department of Pediatrics and Novo Nordisk Foundation Center for Biosustainability, University of California, San Diego School of Medicine, La Jolla, CA USA; 29 Key Laboratory of Systems Microbial Biotechnology, Tianjin Institute of Industrial Biotechnology, Chinese Academy of Sciences, Tianjin, P.R. China; 30 Structural and Computational Biology Unit, European Molecular Biology Laboratory (EMBL), Heidelberg, Germany; 31 Institute of Biomaterials and Biomedical Engineering, University of Toronto, Toronto, Canada; 32 SilicoLife Lda., Braga, Portugal; 33 Department of Biomedical Engineering, University of Virginia, Charlottesville, Virginia USA; 34 Chalmers University of Technology, Department of Biology and Biological Engineering, Division of Systems and Synthetic Biology, Göteborg, Sweden; 35 Department of Biomedical Informatics, College of Medicine, University of Arkansas for Medical Sciences (UAMS), Little Rock, AR USA; 36 Oxford Brookes University, Oxford, UK; 37 Centre of Biological Engineering, University of Minho, Braga, Portugal; 38 Instituto de Tecnologia Química e Biológica António Xavier, Universidade Nova de Lisboa (ITQB-NOVA), Oeiras, Portugal; 39 Institute for Molecular Evolution, Heinrich-Heine-University Düsseldorf, Düsseldorf, Germany; 40 Present Address: Centro de Biotecnología y Genómica de Plantas (CBGP, UPM-INIA), Universidad Politécnica de Madrid (UPM) – Instituto Nacional de Investigación y Tecnología Agraria y Alimentaria (INIA), Madrid, Spain

**Keywords:** Computational models, Software, Biochemical networks

Correction to: *Nature Biotechnology* 10.1038/s41587-020-0446-y, published online 2 March 2020.

This paper was originally published under standard Springer Nature copyright (© The Author(s), under exclusive licence to Springer Nature America, Inc.). It is now available as an open-access paper under a Creative Commons Attribution 4.0 International license. The error has been corrected in the HTML and PDF versions of the article.

