# Supplementary material for: MEMOTE for standardized genome-scale metabolic model testing
Source: Nat Biotechnol. 2020 Mar 2;38(3):272–6. doi: 10.1038/s41587-020-0446-y (PMC7082222; doi:10.1038/s41587-020-0446-y)
Supplement: Supplementary file 1 — Supplementary Figs. 1–162, Notes 1–5, Methods, and Tables 1 and 2 [file 41587_2020_446_MOESM1_ESM.pdf]

nature biotechnology

SUPPLEMENTARY INFORMATION

https://doi.org/10.1038/s41587-020-0446-y

In the format provided by the authors and unedited.

MEMOTE for standardized genome-scale metabolic model testing

Christian Lieven[1,41], Moritz E. Beber[1,41], Brett G. Olivier[2], Frank T. Bergmann[3], Meric Ataman[4], Parizad Babaei[1], Jennifer A. Bartell[1], Lars M. Blank[5], Siddharth Chauhan[6], Kevin Correia[7], Christian Diener[8,9], Andreas Dräger[10,11,12], Birgitta E. Ebert[5,13], Janaka N. Edirisinghe[14], José P. Faria[14], Adam M. Feist[1,6], Georgios Fengos[4], Ronan M. T. Fleming[15], Beatriz García-Jiménez[16,40], Vassily Hatzimanikatis[4], Wout van Helvoirt[17,18], Christopher S. Henry[14], Henning Hermjakob[19], Markus J. Herrgård[1], Ali Kaafarani[1], Hyun Uk Kim[20], Zachary King[6], Steffen Klamt[21], Edda Klipp[22], Jasper J. Koehorst[23], Matthias König[22], Meiyappan Lakshmanan[24], Dong-Yup Lee[24,25], Sang Yup Lee[1,20], Sunjae Lee[26,27], Nathan E. Lewis[6,28], Filipe Liu[14], Hongwu Ma[29], Daniel Machado[30], Radhakrishnan Mahadevan[7,31], Paulo Maia[32], Adil Mardinoglu[26,27], Gregory L. Medlock[33], Jonathan M. Monk[6], Jens Nielsen[1,34], Lars Keld Nielsen[1,13], Juan Nogales[16], Intawat Nookaew[34,35], Bernhard O. Palsson[1,6], Jason A. Papin[33], Kiran R. Patil[30], Mark Poolman[36], Nathan D. Price[9], Osbaldo Resendis-Antonio[8], Anne Richelle[28], Isabel Rocha[37,38], Benjamín J. Sánchez[1,34], Peter J. Schaap[23], Rahuman S. Malik Sheriff[19], Saeed Shoaie[26,27], Nikolaus Sonnenschein[1] ✉, Bas Teusink[2], Paulo Vilaça[32], Jon Olav Vik[17], Judith A. H. Wodke[22], Joana C. Xavier[39], Qianqian Yuan[29], Maksim Zakhartsev[17] and Cheng Zhang[26]

[1]Novo Nordisk Foundation Center for Biosustainability, Technical University of Denmark, Lyngby, Denmark. [2]Systems Biology Lab, Amsterdam Institute of Molecular and Life Sciences (AIMMS), Vrije Universiteit Amsterdam, Amsterdam, the Netherlands. [3]BioQUANT/COS, Heidelberg University, Heidelberg, Germany. [4]Ecole Polytechnique Fédérale de Lausanne, Laboratory of Computational Systems Biotechnology, Lausanne, Switzerland. [5]iAMB-Institute of Applied Microbiology, ABBt-Aachen Biology and Biotechnology, RWTH Aachen University, Aachen, Germany. [6]Department of Bioengineering, University of California, La Jolla, CA, USA. [7]Department of Chemical Engineering and Applied Chemistry, University of Toronto, Toronto, Ontario, Canada. [8]Human Systems Biology Laboratory, Instituto Nacional de Medicina Genomica & Coordinación de la Investigación Científica-Red de Apoyo a la Investigación, UNAM, Mexico City, Mexico. [9]Institute for Systems Biology, Seattle, WA, USA. [10]Computational Systems Biology of Infection and Antimicrobial-Resistant Pathogens, Institute for Biomedical Informatics (IBMI), Tübingen, Germany. [11]Department of Computer Science, University of Tübingen, Tübingen, Germany. [12]German Center for Infection Research (DZIF), partner site Tübingen, Tübingen, Germany. [13]Australian Institute for Bioengineering and Nanotechnology, The University of Queensland, Brisbane, Queensland, Australia. [14]Argonne National Laboratory, Lemont, IL, USA. [15]Analytical Biosciences, Division of Systems Biomedicine and Pharmacology, Leiden Academic Centre for Drug Research, Leiden University, Leiden, the Netherlands. [16]Department of Systems Biology, Centro Nacional de Biotecnología, Consejo Superior de Investigaciones Científicas (CNB-CSIC), Madrid, Spain. [17]Department of Animal and Aquacultural Sciences, Faculty of Biosciences, Norwegian University of Life Sciences, Oslo, Norway. [18]Hanze University of Applied Sciences, Groningen, the Netherlands. [19]European Bioinformatics Institute, European Molecular Biology Laboratory (EMBL-EBI), Wellcome Trust Genome Campus, Cambridge, UK. [20]Department of Chemical and Biomolecular Engineering (BK21 Plus Program), BioProcess Engineering Research Center, BioInformatics Research Center, Institute for the BioCentury, Korea Advanced Institute of Science and Technology (KAIST), Daejeon, Korea. [21]Analysis and Redesign of Biological Networks, Max Planck Institute for Dynamics of Complex Technical Systems Magdeburg, Magdeburg, Germany. [22]Theoretical Biophysics, Humboldt-Universität zu Berlin, Berlin, Germany. [23]Department of Agrotechnology and Food Sciences, Laboratory of Systems and Synthetic Biology, Wageningen University & Research, Wageningen, the Netherlands. [24]Bioprocessing Technology Institute, Agency for Science, Technology and Research (A*STAR), Singapore, Singapore. [25]School of Chemical Engineering Sungkyunkwan University, Jangan-gu Suwon, Gyeonggi-do, Republic of Korea. [26]Science for Life Laboratory, KTH - Royal Institute of Technology, Stockholm, Sweden. [27]Centre for Host-Microbiome Interactions, Faculty of Dentistry, Oral & Craniofacial Sciences, King's College London, London, UK. [28]Department of Pediatrics and Novo Nordisk Foundation Center for Biosustainability, University of California, San Diego School of Medicine, La Jolla, CA, USA. [29]Key Laboratory of Systems Microbial Biotechnology, Tianjin Institute of Industrial Biotechnology, Chinese Academy of Sciences, Tianjin, P.R. China. [30]Structural and Computational Biology Unit, European Molecular Biology Laboratory (EMBL), Heidelberg, Germany. [31]Institute of Biomaterials and Biomedical Engineering, University of Toronto, Toronto, Canada. [32]SilicoLife Lda., Braga, Portugal. [33]Department of Biomedical Engineering, University of Virginia, Charlottesville, Virginia, USA. [34]Chalmers University of Technology, Department of Biology and Biological Engineering, Division of Systems and Synthetic Biology, Göteborg, Sweden. [35]Department of Biomedical Informatics, College of Medicine, University of Arkansas for Medical Sciences (UAMS), Little Rock, AR, USA. [36]Oxford Brookes University, Oxford, UK. [37]Centre of Biological Engineering, University of Minho, Braga, Portugal. [38]Instituto de Tecnologia Química e Biológica António Xavier, Universidade Nova de Lisboa (ITQB-NOVA), Oeiras, Portugal. [39]Institute for Molecular Evolution, Heinrich-Heine-University Düsseldorf, Düsseldorf, Germany. [40]Present address: Centro de Biotecnología y Genómica de Plantas (CBGP, UPM-INIA), Universidad Politécnica de Madrid (UPM) – Instituto Nacional de Investigación y Tecnología Agraria y Alimentaria (INIA), Madrid, Spain. [41]These authors contributed equally: Christian Lieven,

Supplementary Material

Contents

1 Supplementary Figures: Arranged as they appear in the manuscript **2**
1.1 From Supplementary Note 1 . . . 2
1.2 From Supplementary Material . . . 4

2 Supplementary Note 1: Guide on how to understand the reports **12**
2.1 Understanding the reports . . . 12
2.1.1 Orientation . . . 12
2.1.1.1 Toolbar . . . 12
2.1.1.2 Main Body . . . 15
2.1.1.3 Test Results . . . 15
2.1.2 Interpretation . . . 15
2.1.2.1 Color . . . 16
2.1.2.2 Score . . . 16
2.1.3 Paradigms . . . 17
2.1.3.1 "Reconstructions" and "Models" . . . 17
2.1.3.2 "Lumped" and "Split" Biomass Reaction . . . 17
2.1.3.3 "Average" and "Unique" Metabolites . . . 17

3 Supplementary Note 2: Validation against experimental data **17**

4 Supplementary Note 3: Integration in third party tools and services **20**

5 Supplementary Note 4: Discussion of alternatives to memote **20**

6 Supplementary Note 5: Outlook **20**

7 Supplementary Methods **21**
7.1 Tested models . . . 21
7.2 Clustering . . . 21
7.3 Test Suite . . . 26
7.3.1 Summary of Observations . . . 28
7.3.2 Scores . . . 28
7.3.3 Independent Section . . . 29
7.3.3.1 Consistency . . . 29
7.3.3.2 Annotation - Metabolites . . . 33
7.3.3.3 Annotation - Reactions . . . 47
7.3.3.4 Annotation - Genes . . . 59
7.3.3.5 Annotation - SBO Terms . . . 71
7.3.4 Specific Section . . . 78
7.3.4.1 SBML . . . 78

7.3.4.2 Basic Information 79
7.3.4.3 Metabolite Information 83
7.3.4.4 Reaction Information 86
7.3.4.5 Gene-Protein-Reaction (GPR) Association 91
7.3.4.6 Biomass 93
7.3.4.7 Energy Metabolism 97
7.3.4.8 Network Topology 99
7.3.4.9 Matrix Conditioning 103

8 Author Contributions **105**

Supplementary References **106**

1 Supplementary Figures: Arranged as they appear in the manuscript

To see the figures in context, please refer to the corresponding sections below.

1.1 From Supplementary Note 1

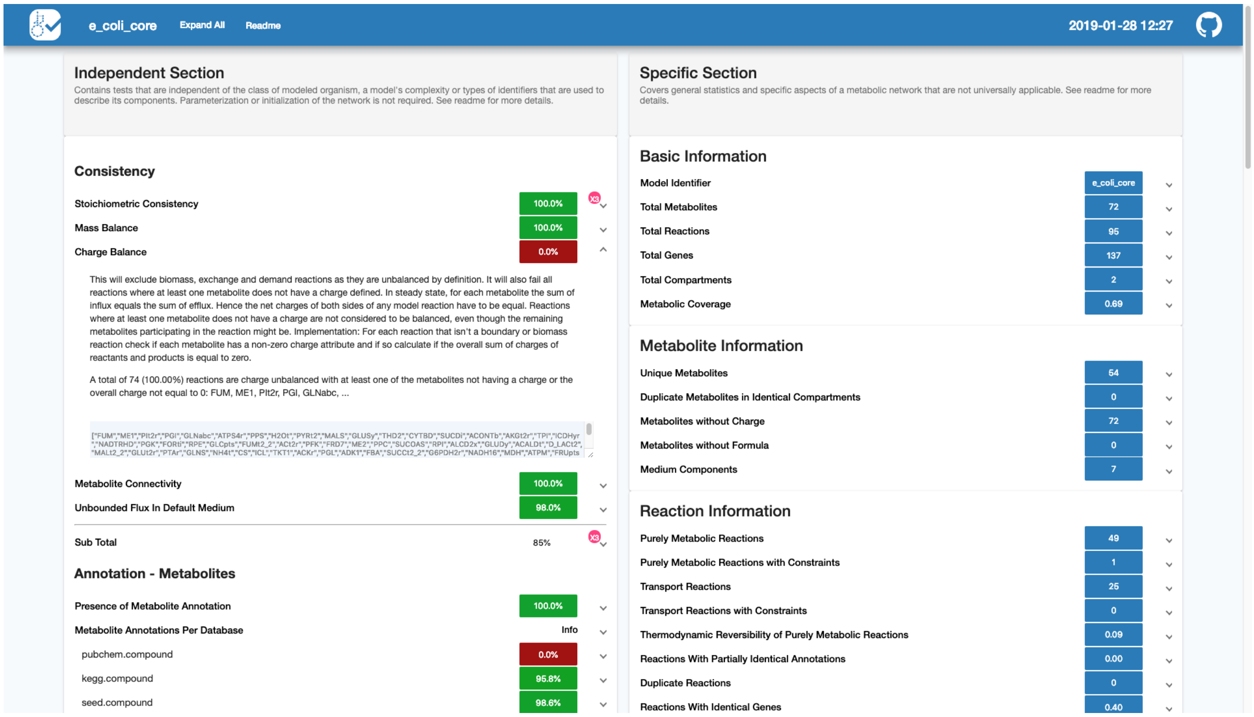

Figure 1: Snapshot Report

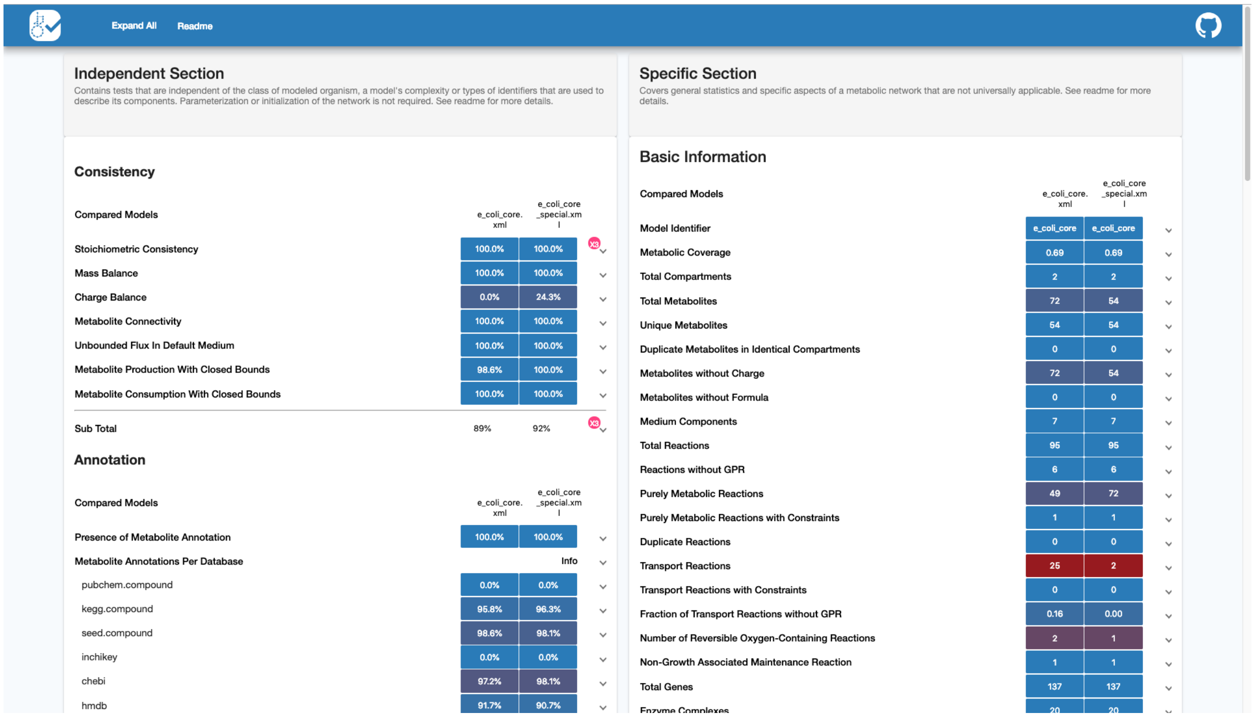

Figure 2: Diff Report

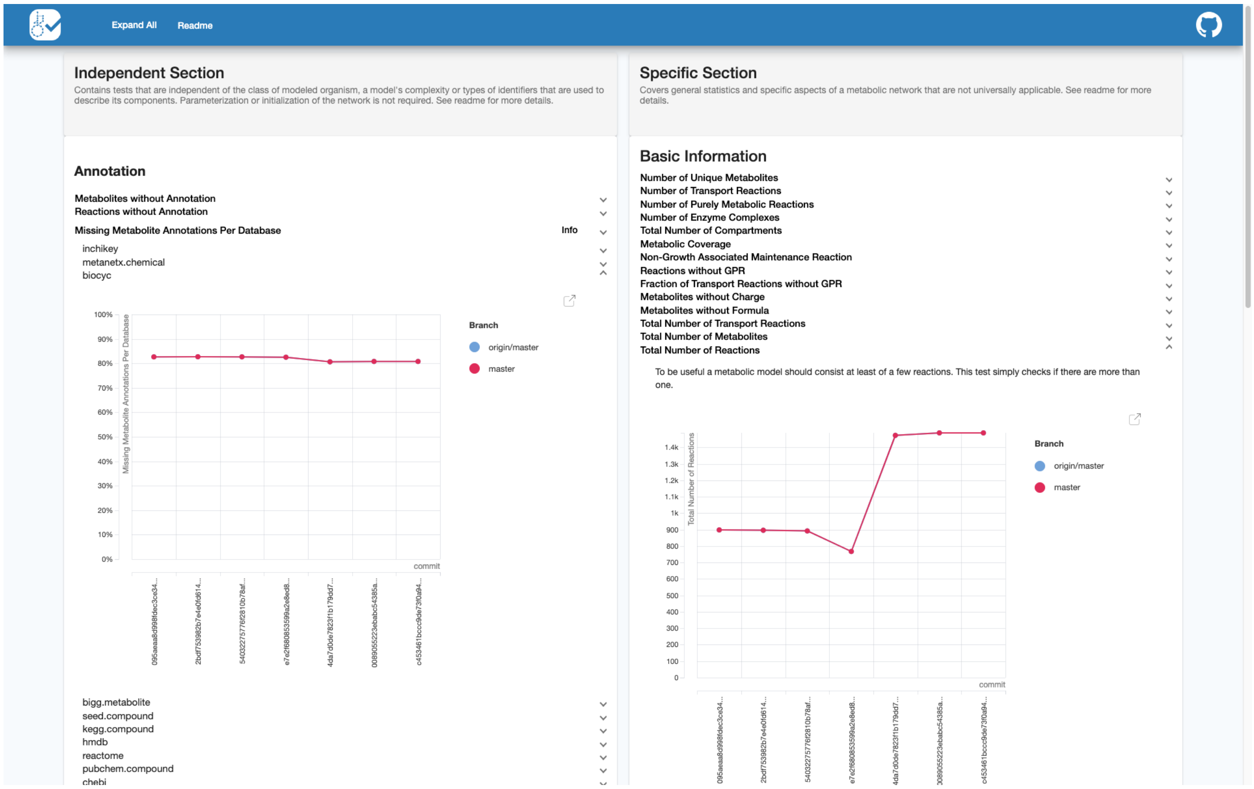

Figure 3: History Report

1.2 From Supplementary Material

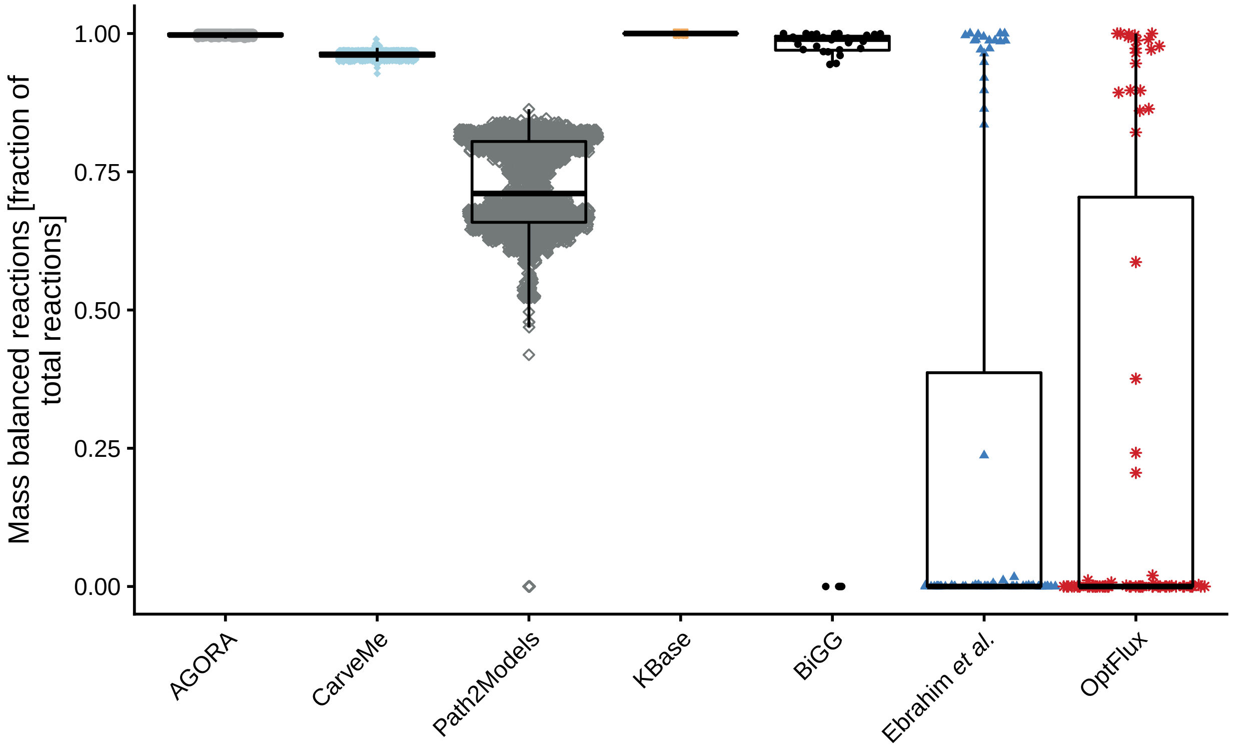

Figure 4: Mass Balance. Please note that any reaction where at least one metabolite lacks a formula annotation is considered as unbalanced for the purpose of this test.

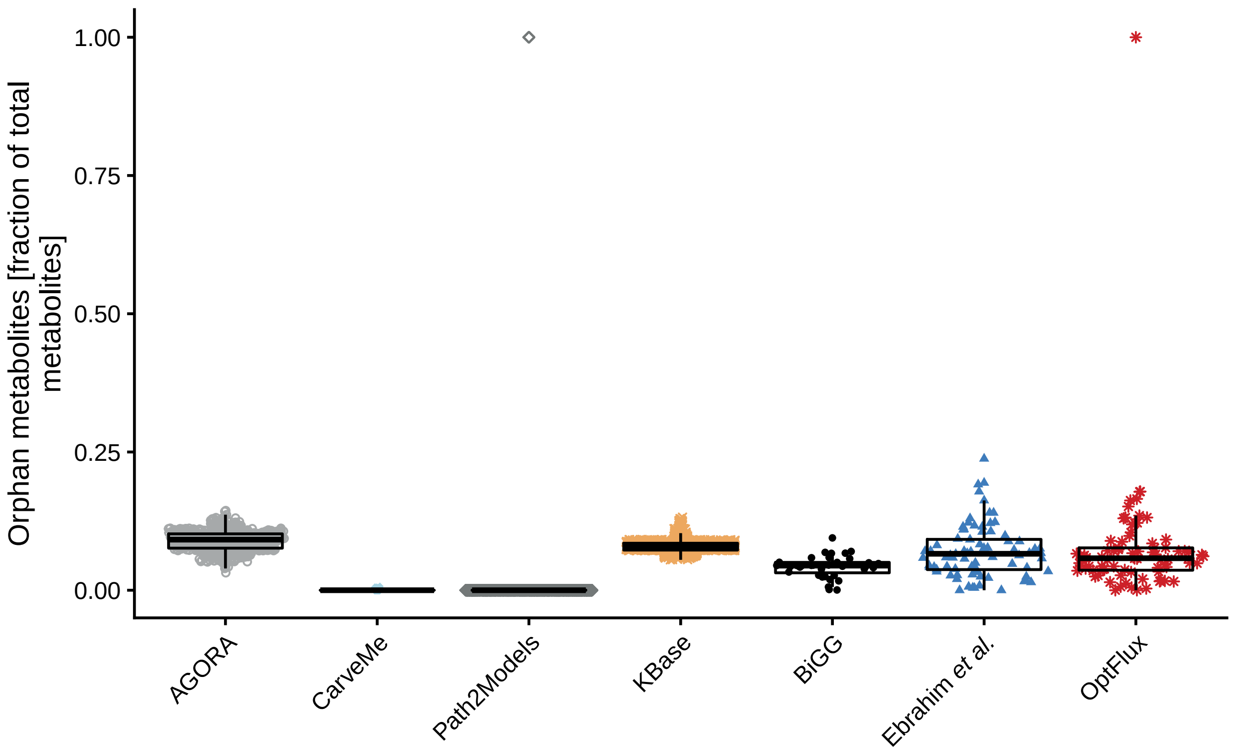

Figure 5: Orphan Metabolites

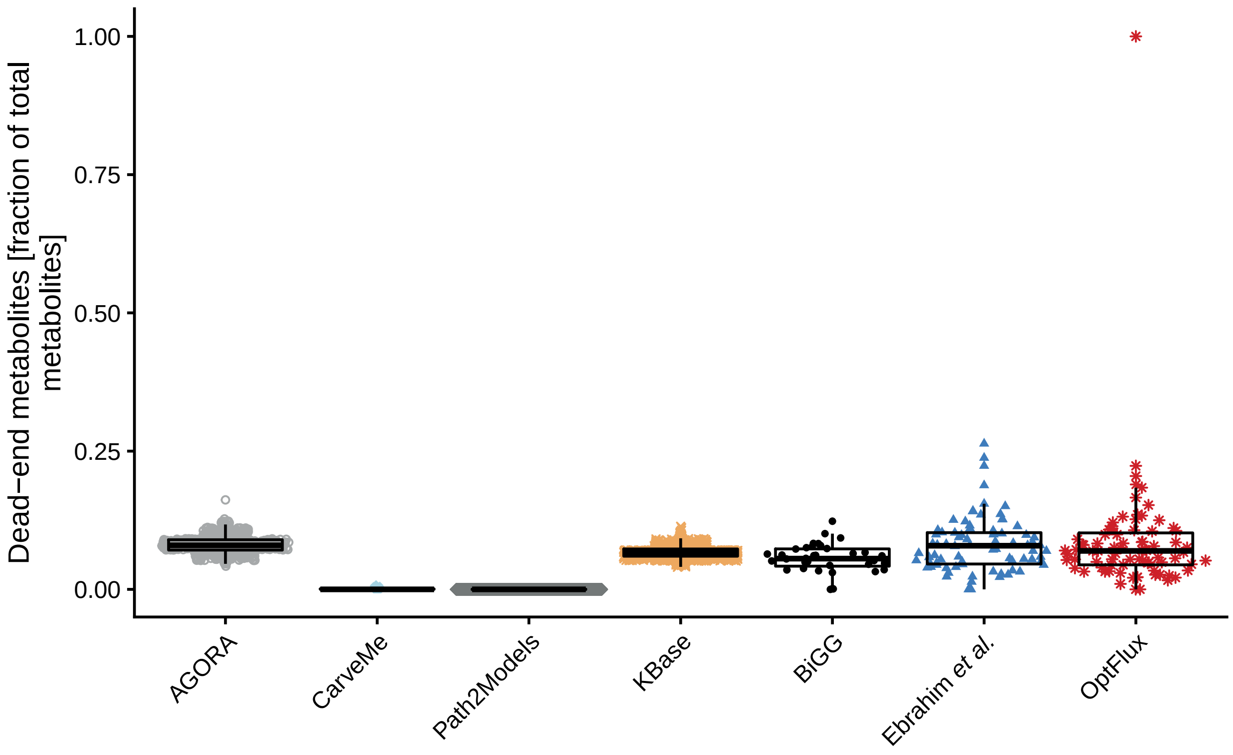

Figure 6: Dead-end Metabolites

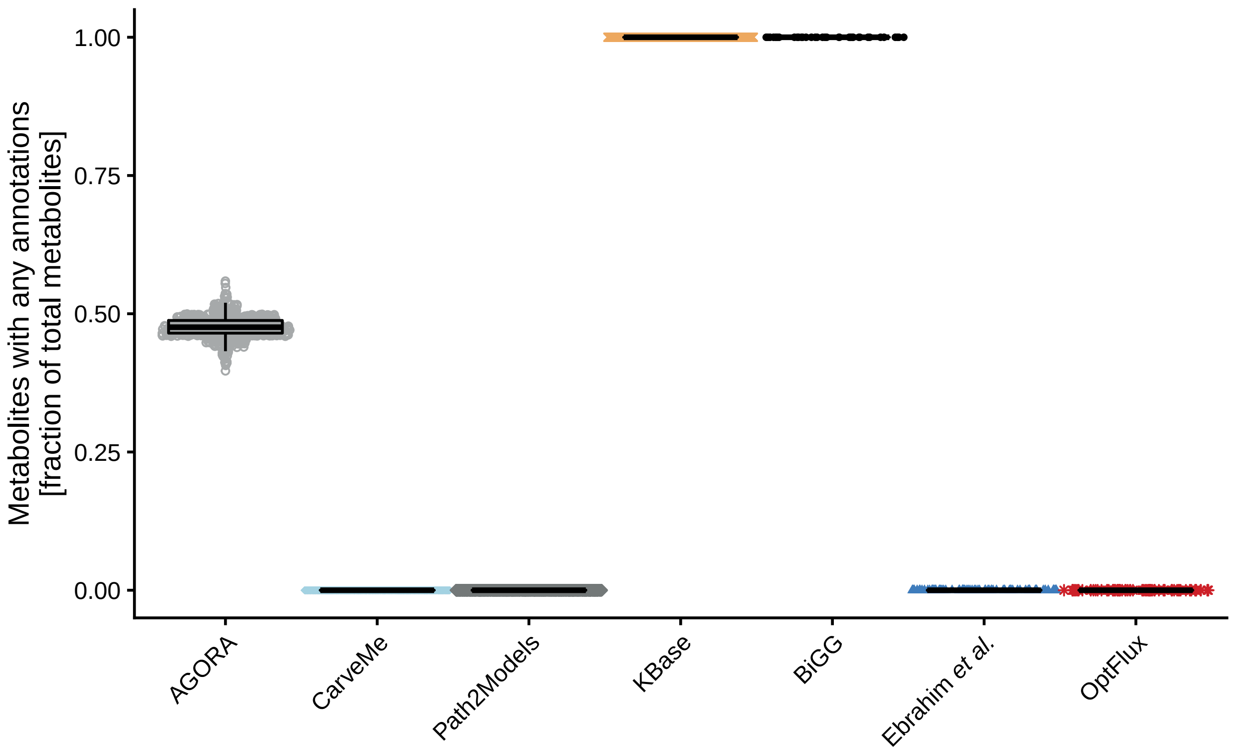

Figure 7: Presence of Metabolite Annotation

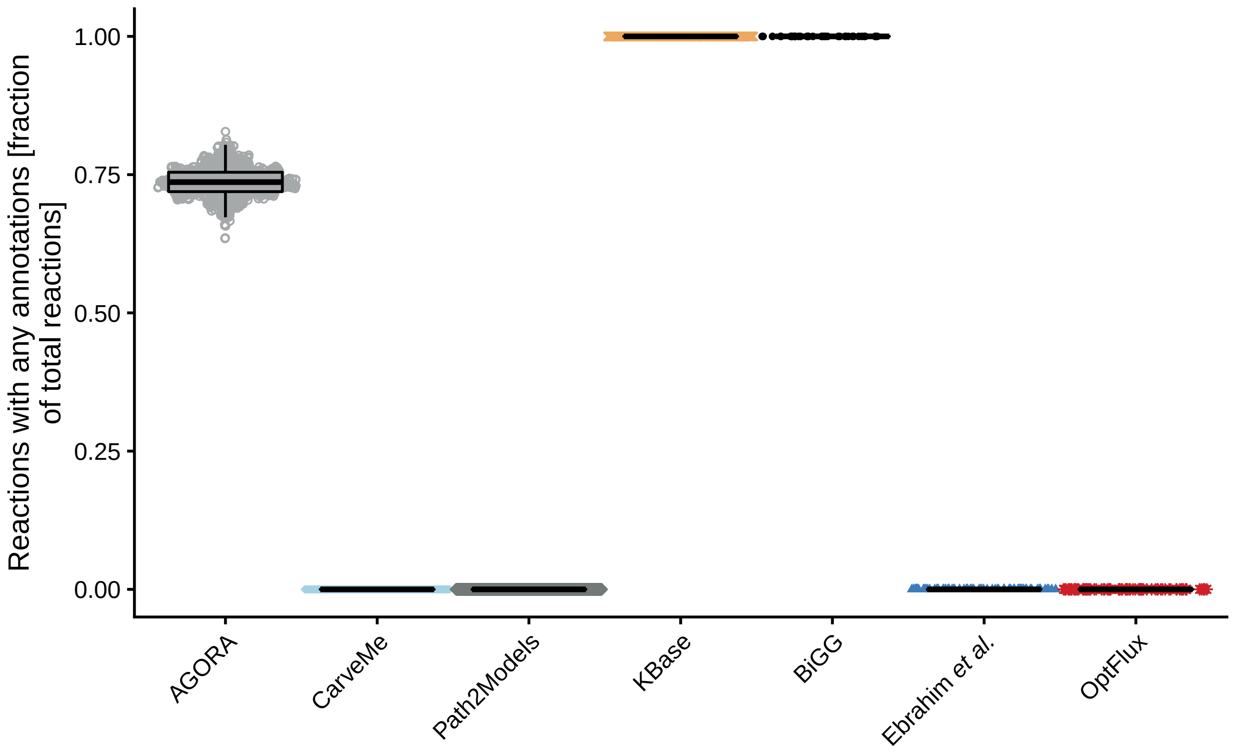

Figure 8: Presence of Reaction Annotation

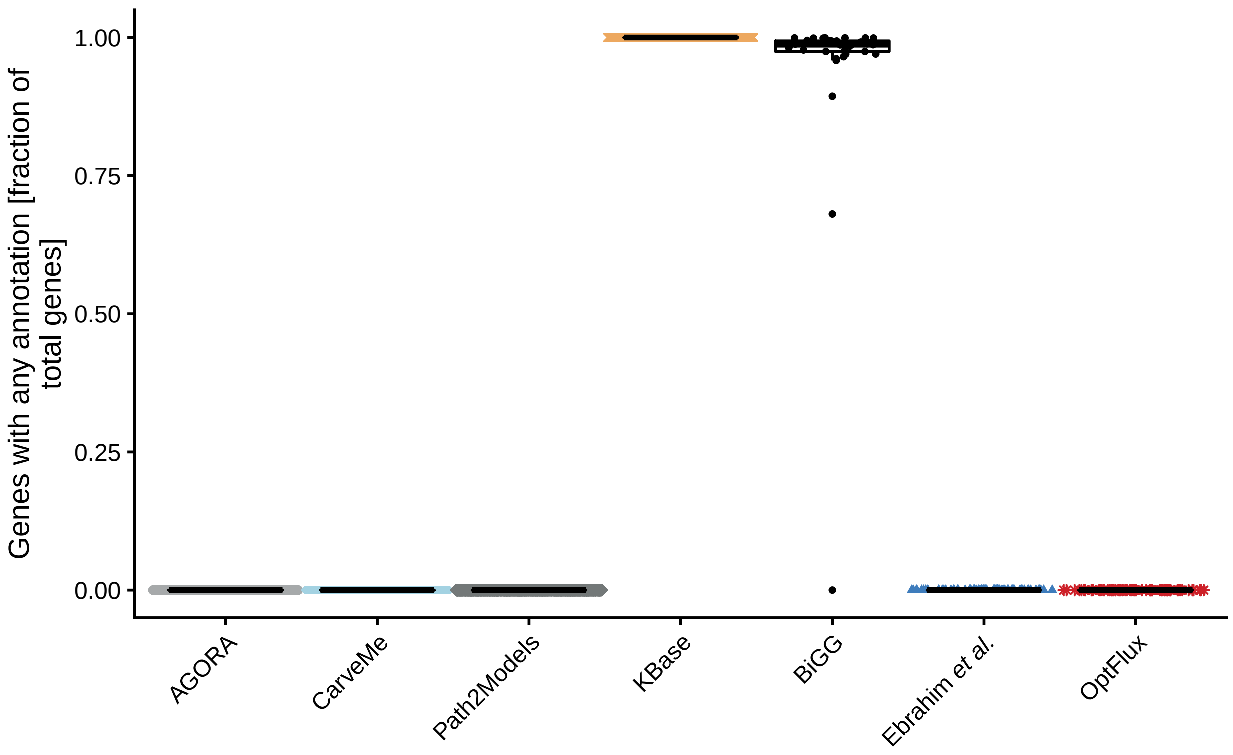

Figure 9: Presence of Gene Annotation

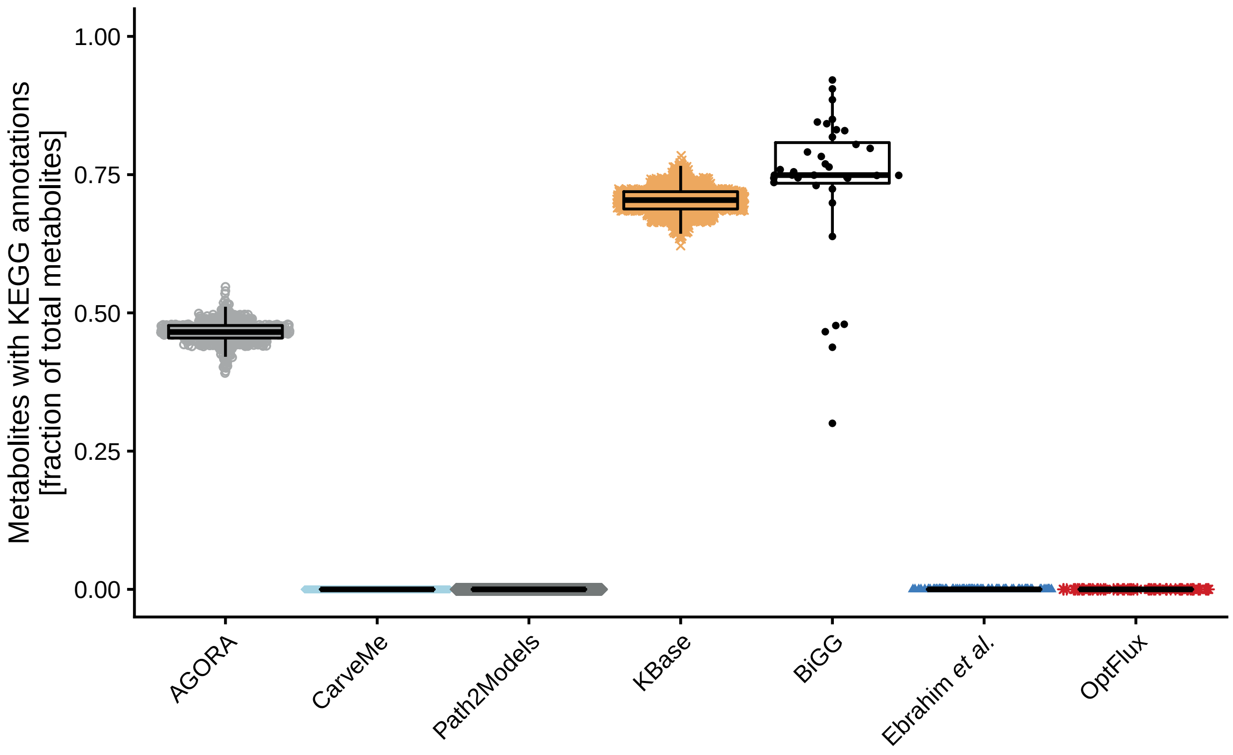

Figure 10: Metabolite KEGG.compound Annotation

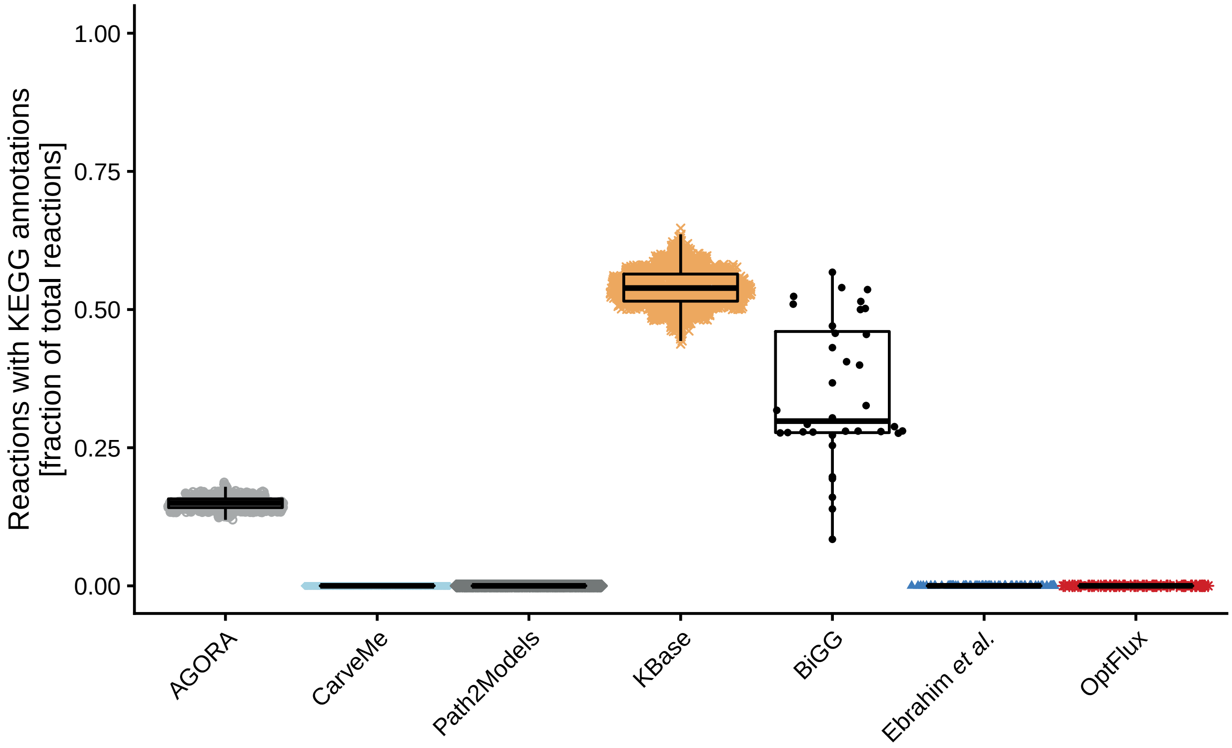

Figure 11: Reaction KEGG.reaction Annotation

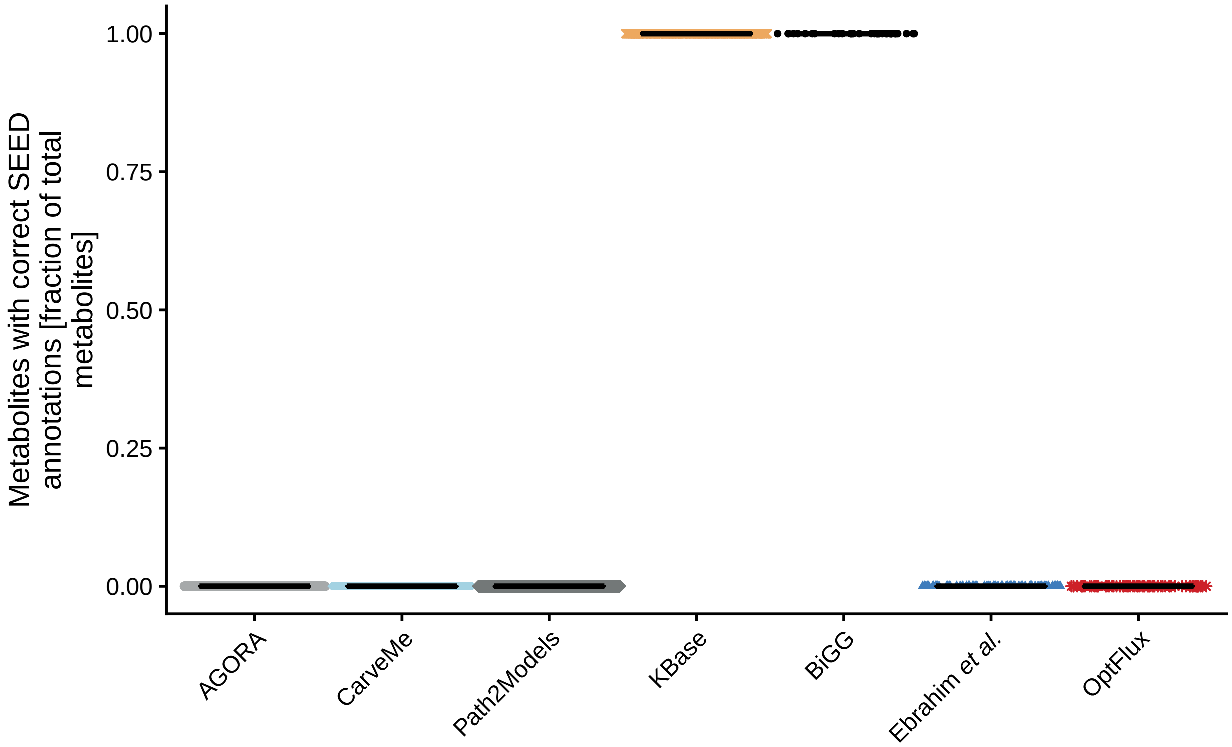

Figure 12: Correct Metabolite SEED.compound Annotation

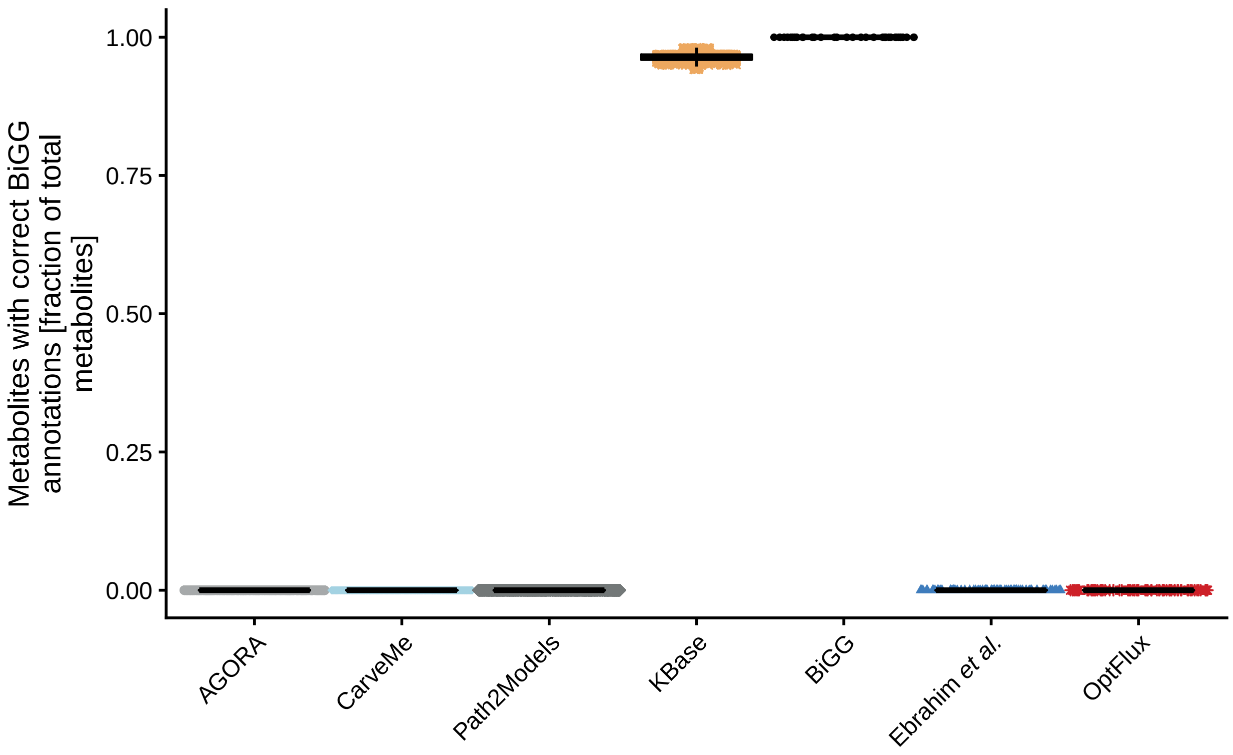

Figure 13: Correct Metabolite BiGG.metabolite Annotation

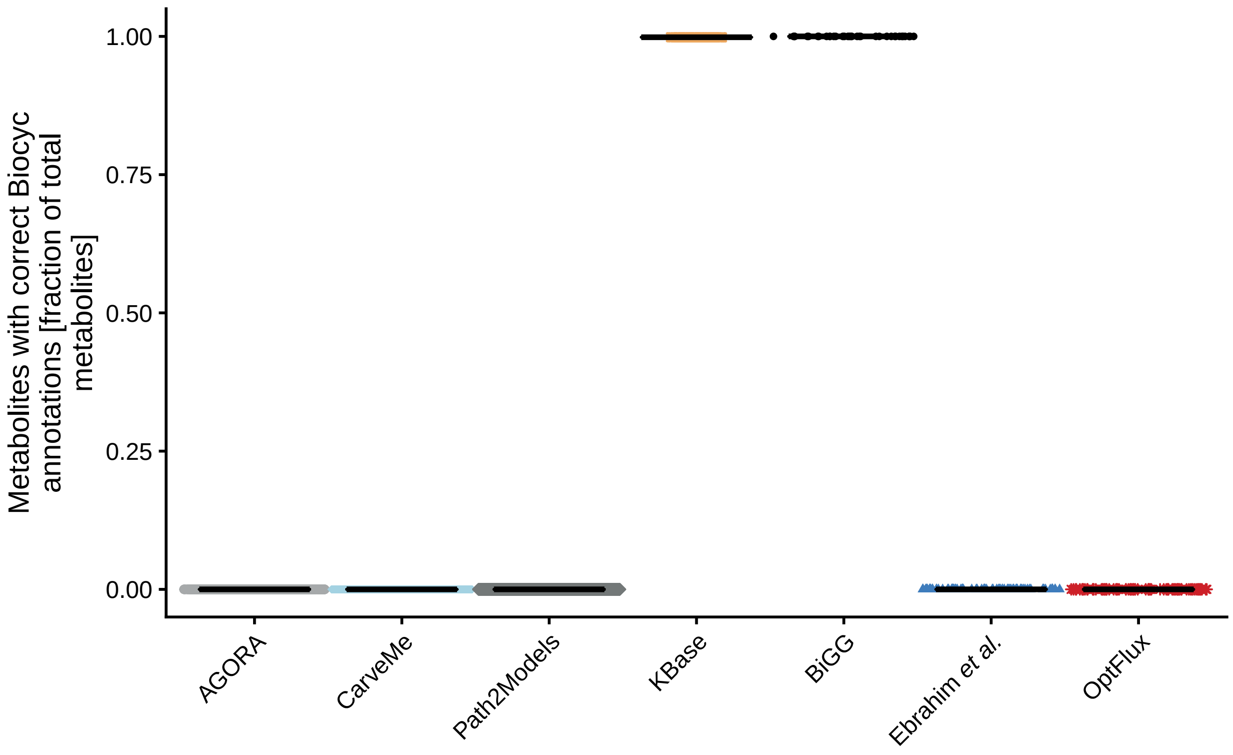

Figure 14: Correct Metabolite BioCyc Annotation

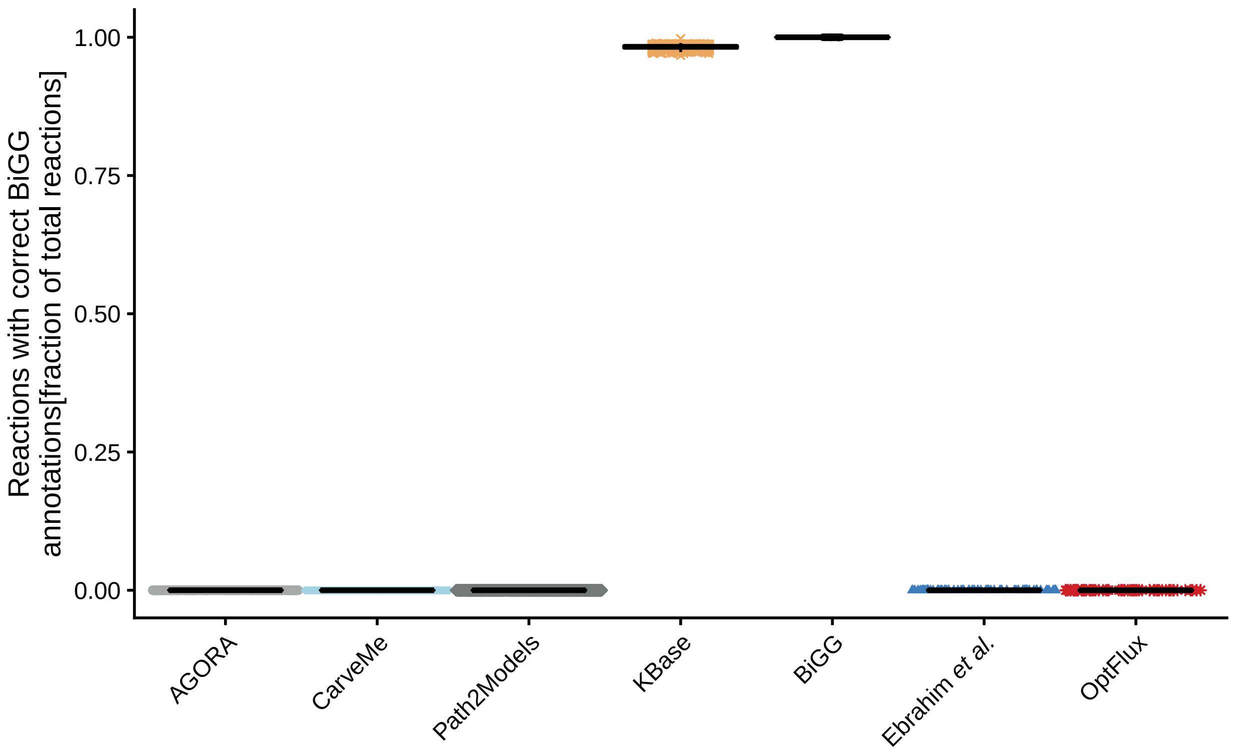

Figure 15: Correct Reaction BiGG.reaction Annotation

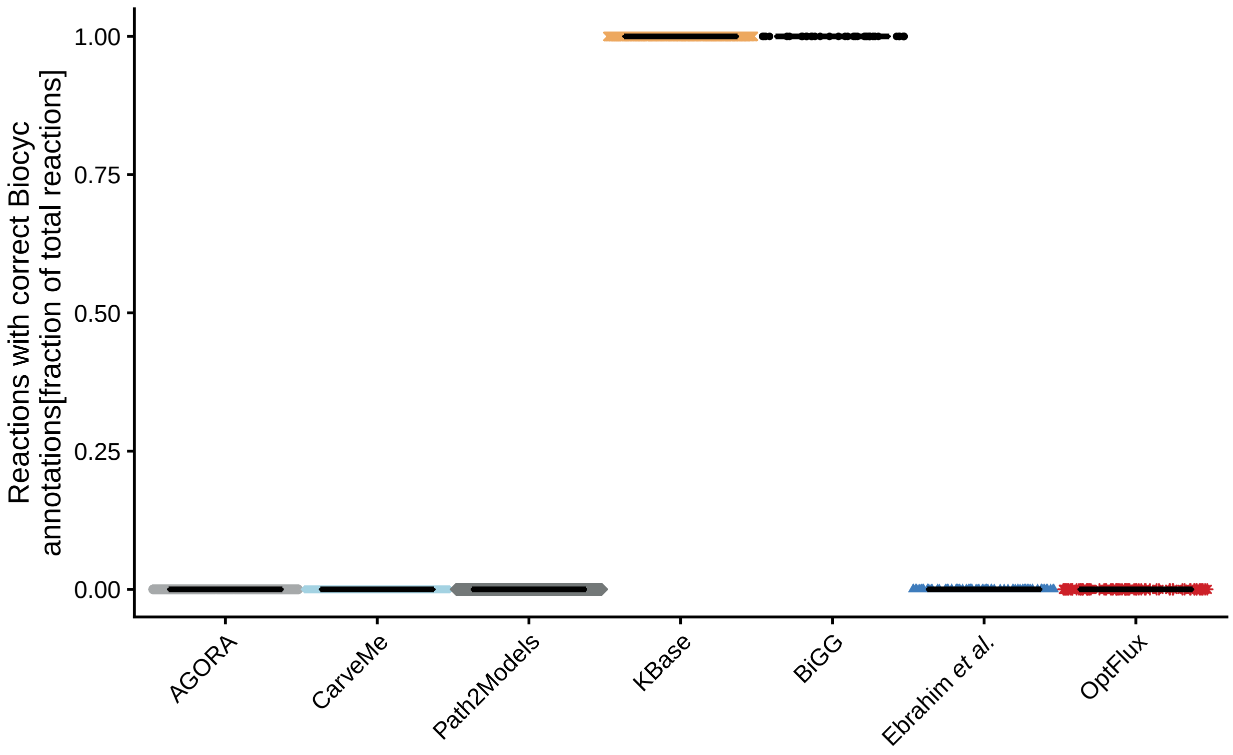

Figure 16: Correct Reaction BioCyc Annotation

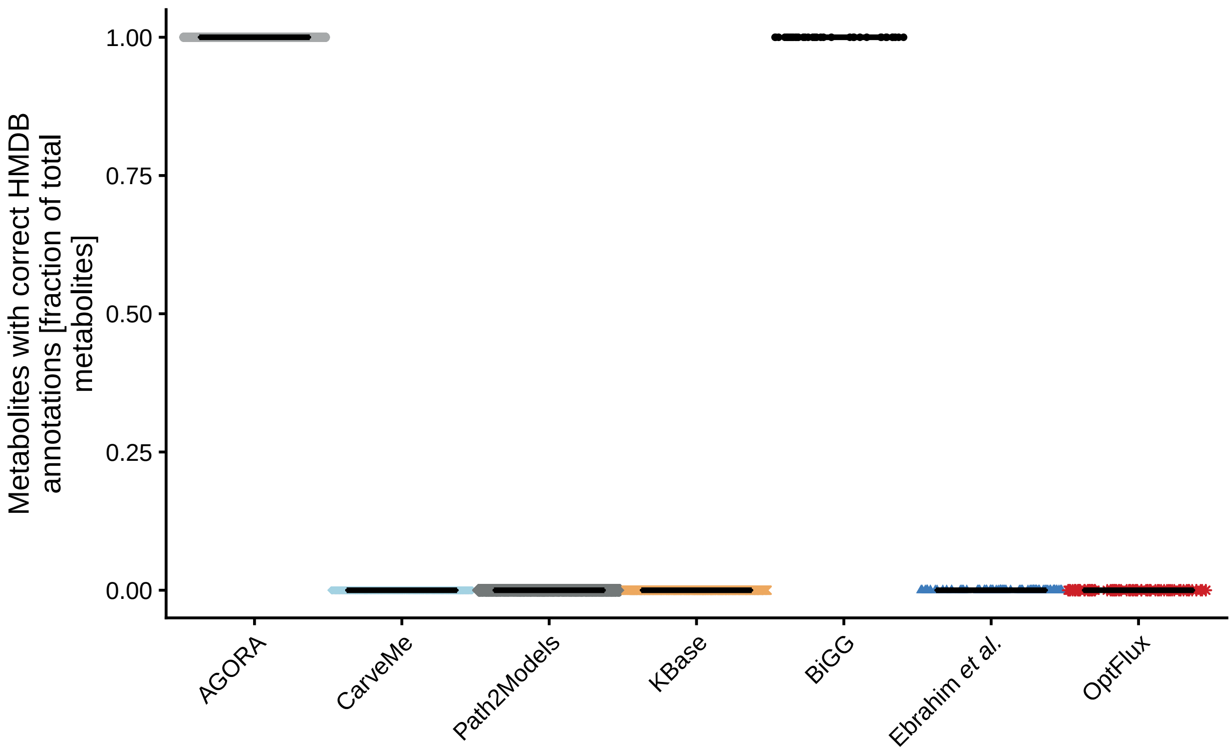

Figure 17: Correct Metabolite HMDB Annotation

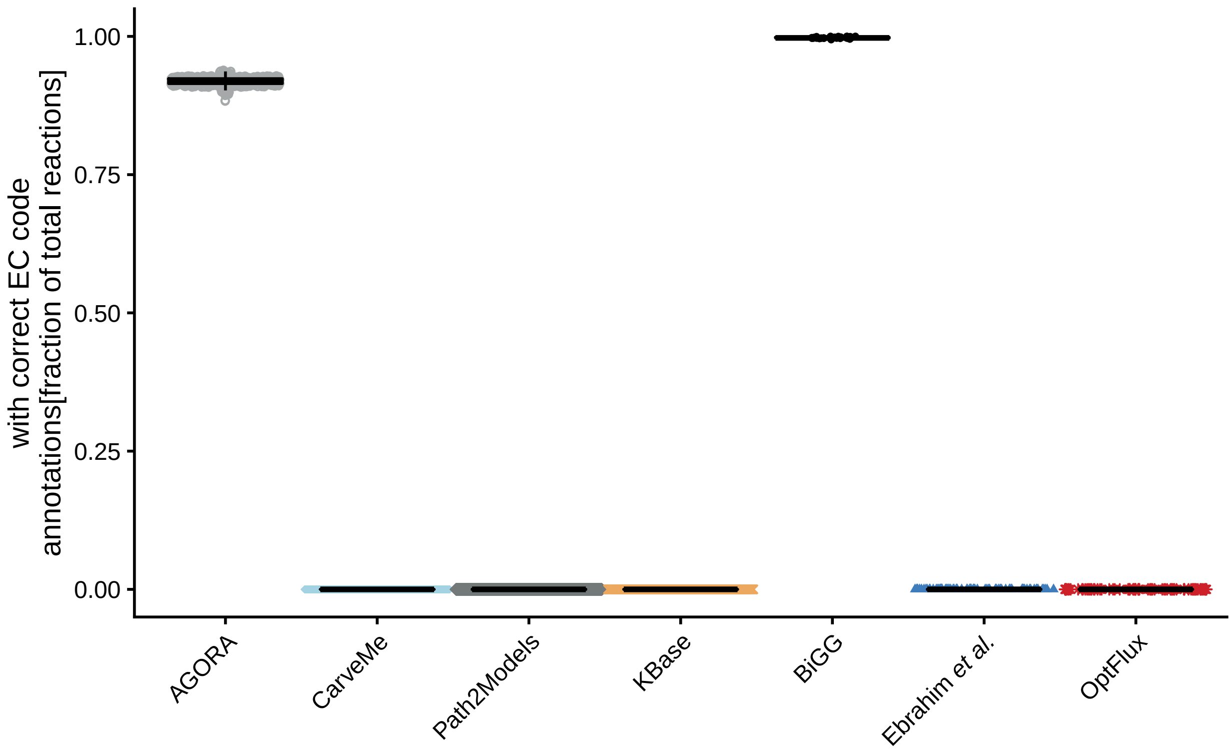

Figure 18: Correct Reaction Enzyme Classification Annotation

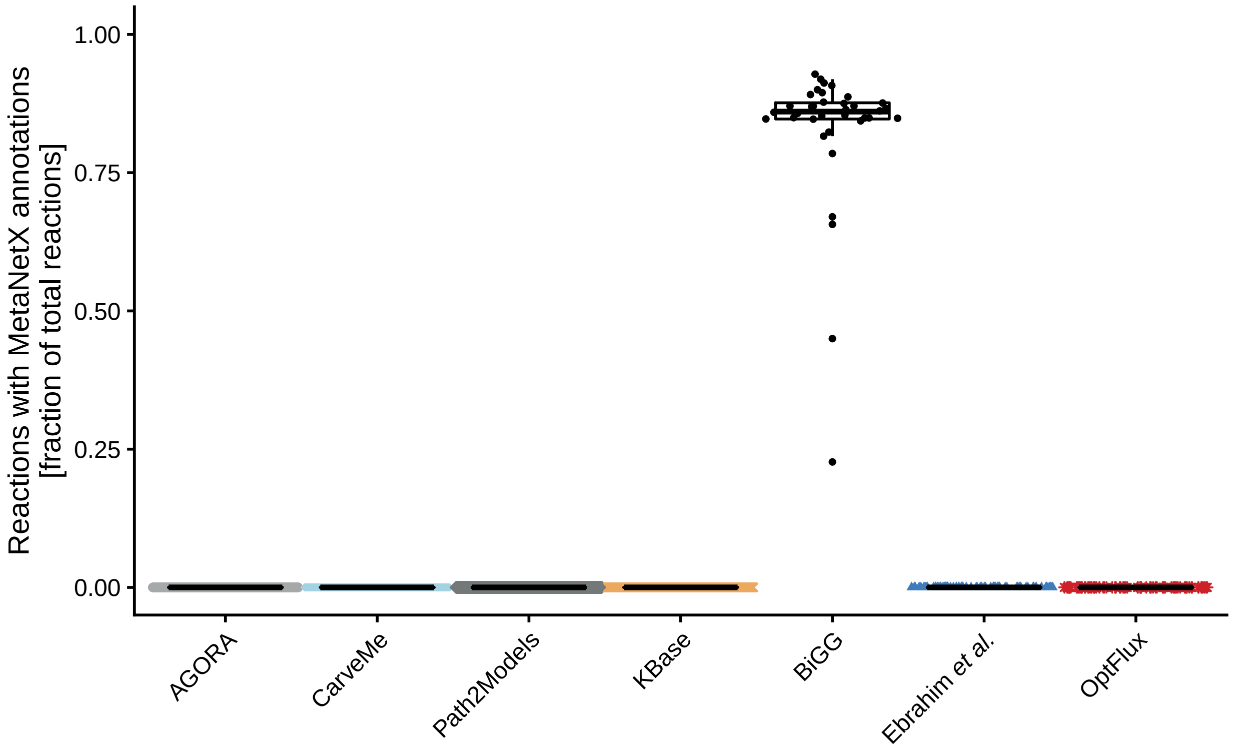

Figure 19: Reaction MetaNetX.reaction Annotation

2 Supplementary Note 1: Guide on how to understand the reports

The following text is a verbatim copy modified to work in print taken from memote's documentation at the time of publication. For an updated version please check the latest memote documentation.

2.1 Understanding the reports

Memote will return one of four possible outputs. If your preferred workflow is to benchmark one or several genome-scale metabolic models (GSM) memote generates either a snapshot (Figure 20) or a diff report (Figure 21), respectively. For the reconstruction workflow the primary output is a history report (Figure 22). This will only work if the provided input models are formatted correctly in the systems biology markup language (SBML). However, if a provided model is not a valid SBML file, memote composes a report enumerating errors and warnings from the SBML validator the order of appearance. To better understand the output of the error report we refer the reader to this section of the SBML documentation. In this section, we will focus on how to understand the snapshot, diff and history reports.

2.1.1 Orientation

2.1.1.1 Toolbar

In all three reports, the blue toolbar at the top shows (from left to right) the memote logo, a button which expands and collapses all test results, a button which displays the readme and the github

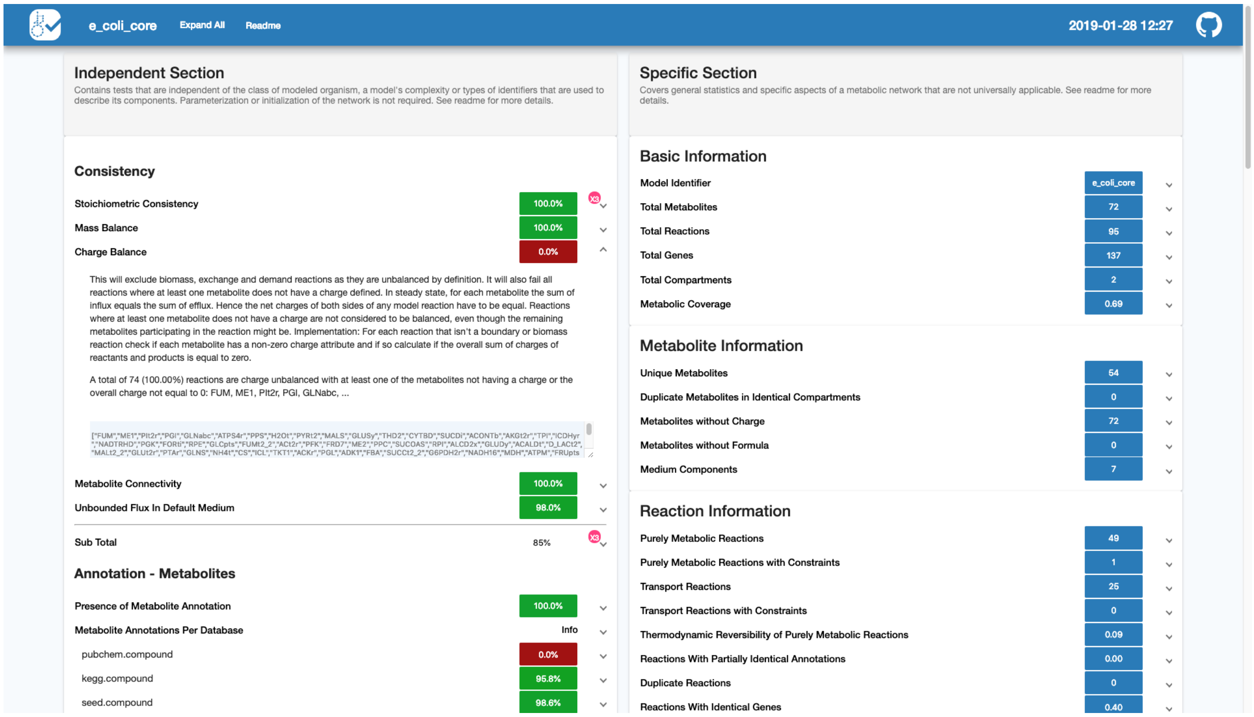

Figure 20: Snapshot Report

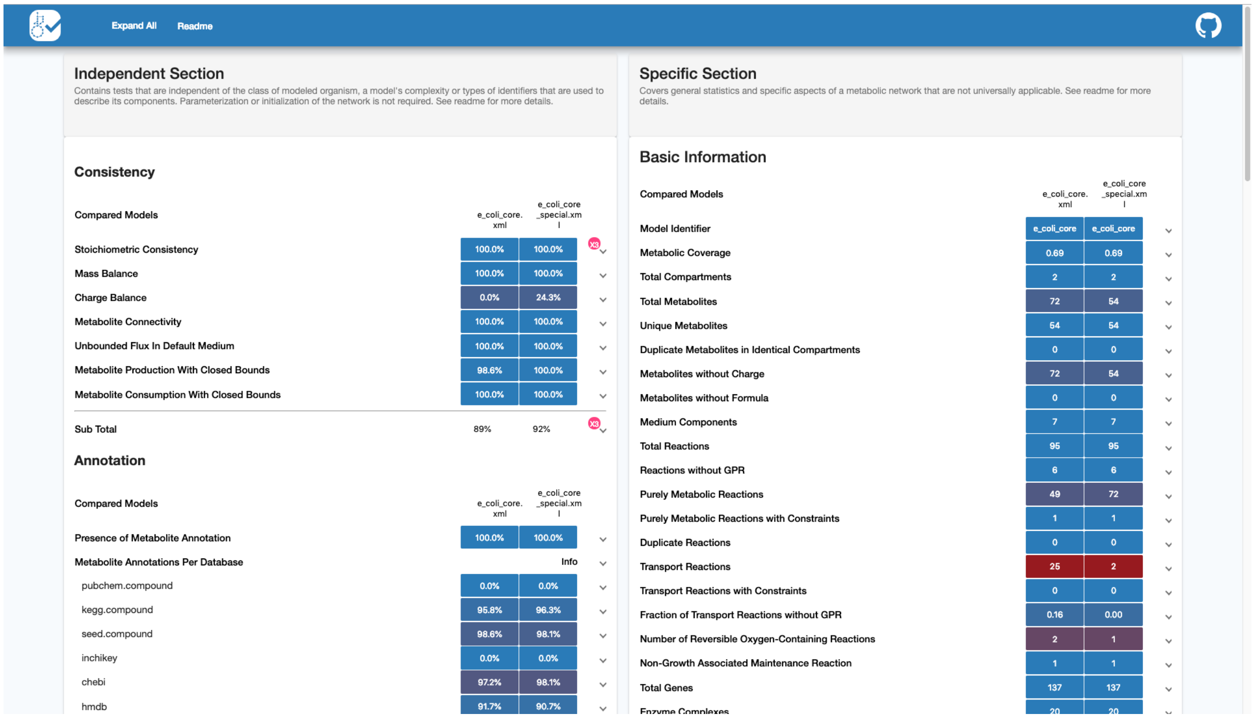

Figure 21: Diff Report

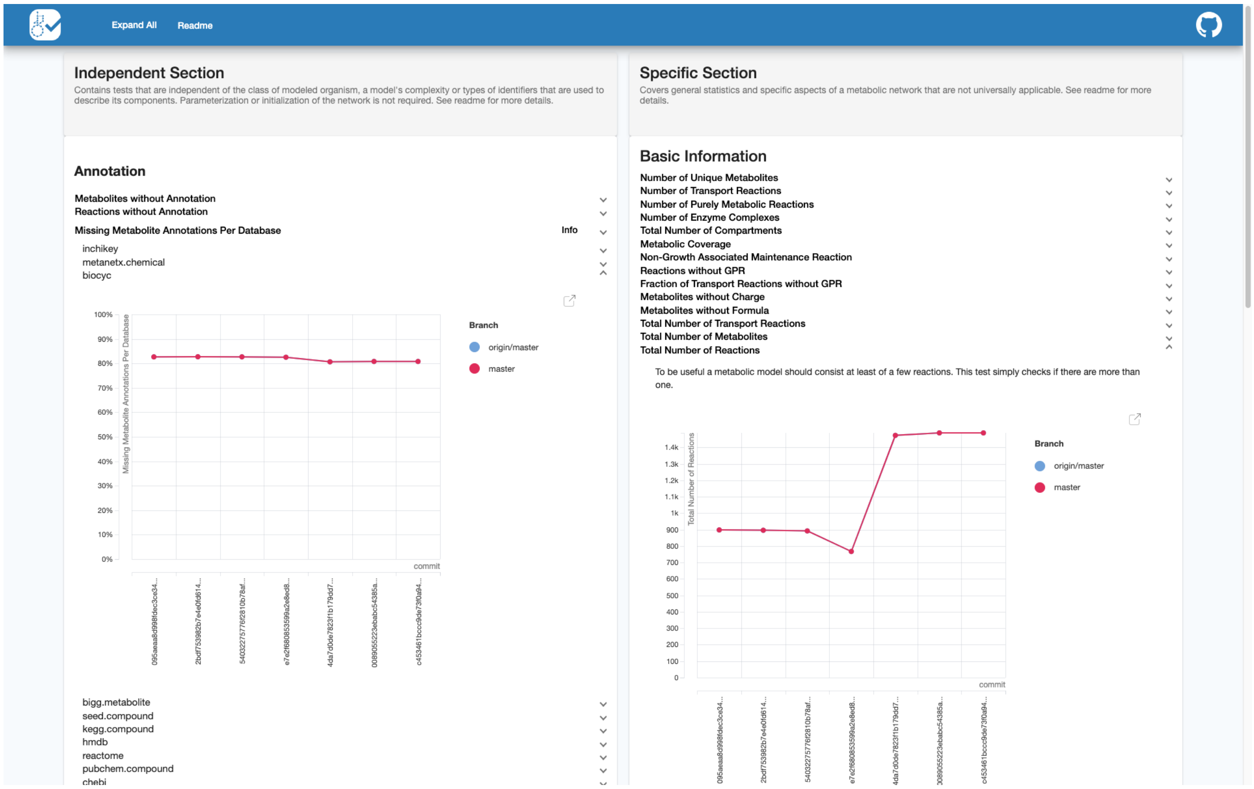

Figure 22: History Report

icon which links to memote's github page. On the snapshot report, the toolbar will also display the identifier of the tested GEM and a timestamp showing when the test run was initiated.

2.1.1.2 Main Body

The main body of the reports is divided into an independent section to the left and a specific section to the right.

The tests in the independent section are agnostic of the type of modeled organism, preferred modeling paradigms, the complexity of a genome-scale metabolic model (GEM) or the types of identifiers that are used to describe its components. The tests in this section focus on testing adherence to fundamental principles of constraint-based modeling: mass, charge and stoichiometric balance as well as the presence of annotations. The results in this section can be normalized, and thus enable a comparison of GEMs. The **Score** at the bottom of the page summarises the results to further simplify comparison. While calculating an overall score for this section allows for the quick comparison of any two given models at a glance, we recommend a thorough analysis of all results with respect to the desired use case.

The specific section on the right provides model specific statistics and covers aspects of a metabolic network that can not be normalized without introducing bias. For instance, dedicated quality control of the biomass equation only applies to GEMs which are used to investigate cell growth, i.e., those for which a biomass equation has been generated. Some tests in this section are also influenced by whether the tested GEM represents a prokaryote or a eukaryote. Therefore the results cannot be generalized and direct comparisons ought to take bias into account.

2.1.1.3 Test Results

Test results are arranged in rows with the title visible to the left and the result on the right. The result is displayed as white text in a coloured rectangle detailed below in the subsection **Color**.

By default only the minimum information is visible as indicated by an arrow pointing down right of the result. Clicking anywhere in the row will expand the result revealing a description of the concept behind the test, its implementation and a brief summary of the result. In addition, there is a text field which contains plain text representations of Python objects which can be copied and pasted into Python code for follow up procedures.

Some tests carry out one operation on several parameters and therefore deviate slightly from the descriptions above. Expanding the title row reveals only the description, while rows of the individual parameters reveal the text fields.

In the history report, instead of text fields scatterplots show how the respective metrics developed over the commit history for each branch of a repository. By clicking an entry in the legend, it is possible to toggle its visibility in the plot.

2.1.2 Interpretation

The variety of constraints-based modeling approaches and differences between various organisms compound the assessment of GSMs. While memote facilitates model assessment it can only do so

within limitations. Please bear in mind the diversity of Paradigms that challenge some of memote's results.

2.1.2.1 Color

Snapshot Report

Results without highlights are kept in the main blue color of the memote color scheme. Scored results (Figure G1) will be marked with a gradient ranging from red to green denoting a low or a high score respectively:

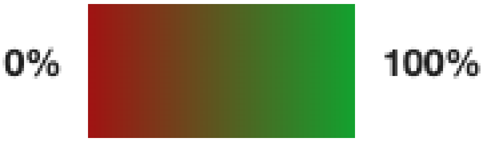

Figure 23: Snapshot Report Score Gradient

Diff Report The colour in the Diff Report (Figure G2) depends on the ratio of the sample minimum to the sample maximum. Result sets where the sample minimum and the sample maximum are identical will be coloured in the main blue color of the memote color scheme. Result sets where the sample minimum is very small relative to the sample maximum will appear red. This ratio is calculated with as

$$1 - (Min/Max)) * 100$$

.

This is then mapped to the following gradient:

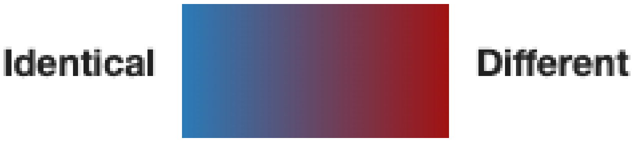

Figure 24: Diff Report Ratio Gradient

2.1.2.2 Score

Each test in the independent section provides a relative measure of completeness with regard to the tested property. The final score is the weighted sum of all individual test results normalized by the maximally achievable score, i.e., all individual results at 100%. Individual tests can be weighted, but it is also possible to apply weighting to entire subsections. Hence the final score is calculated:

$$TotalScore = \frac{\sum_{Subsections} weight_{subsection} \times (\sum_{Tests} weight_{test} \times TestScore)}{MaxScore}$$

Weights for sections and individual tests are indicated by a white number inside a magenta badge. No badge means that the weight defaults to 1.

The subsections “Consistency” and “Annotation - SBO” have weights of 3 and 2, respectively. The test “Stoichiometric Consistency” itself is weighted 3 times stronger than the remaining tests in the “Consistency” subsection. The remaining subsections and tests which cover annotations of metabolites, reactions and genes have weights of 1 (Supplementary Figure G1).

2.1.3 Paradigms

2.1.3.1 “Reconstructions” and “Models”

Some authors may publish metabolic networks which are parameterized, ready to run flux balance analysis (FBA), these are referred to simply as ‘models’. Alternatively, others may publish unconstrained metabolic knowledge bases (referred to as ‘reconstructions’), from which several models can be derived by applying different constraints. Both can be encoded in SBML. With having an independent test section, we attempt to make both ‘models’ and ‘reconstructions’ comparable, although a user should be aware that this difference exists and is subject to some discussion. Please note that some tests in the specific section may error for a reconstruction as they require initialization.

2.1.3.2 “Lumped” and “Split” Biomass Reaction

There are two basic ways of specifying the biomass composition. The most common is a single lumped reaction containing all biomass precursors. Alternatively, the biomass equation can be split into several reactions each focusing on a different macromolecular component for instance a (1 gDW ash) + b (1 gDW phospholipids) + c (free fatty acids)+ d (1 gDW carbohydrates) + e (1 gDW protein) + f (1 gDW RNA) + g (1 gDW DNA) + h (vitamins/cofactors) + x ATP + x H2O-> 1 gDCW biomass + x ADP + x H + x Pi. The benefit of either approach depends very much on the use cases which are discussed by the community. Memote employs heuristics to identify the type of biomass which may fail to distinguish edge cases.

2.1.3.3 “Average” and “Unique” Metabolites

A metabolite consisting of a fixed core with variable branches such as a membrane lipid is sometimes implemented by averaging over the distribution of individual lipid species. The resulting pseudo-metabolite is assigned an average chemical formula, which requires scaling of stoichiometries of associated reactions to avoid floating point numbers in the chemical formulae. An alternative approach is to implement each species as a distinct metabolite in the model, which increases the total count of reactions. Memote cannot yet distinguish between these paradigms, which means that results in the specific sections that rely on the total number of reactions or scaling of stoichiometric parameters may be biased.

3 Supplementary Note 2: Validation against experimental data

To compare model predictions to experimental measurements, a researcher would typically write a short script. The reproducibility of this script may be limited by the original author’s style of writing code, whether the code has been rigorously checked for errors, and whether it is dependent

on obsolete libraries. The latter, so called software rot, arises from a lack of active maintenance (Beaulieu-Jones and Greene 2017).

In contrast, with memote researchers may optionally define a configuration file (in YAML format) in which they can set the medium and FBA objective. This file can be used by researchers without prior programming experience. It configures memote to execute clearly defined, formulaic operations, which are unit tested. Lastly, it confers the burden of maintenance to the memote community represented through this consortium. This does not only distribute the necessity for funding onto many shoulders, but also increases the likelihood of the codebase keeping up with advances in its core dependencies, i.e., keeping software rot at bay. The development of the COBRAToolbox (Heirendt et al. 2017) and cobrapy (Ebrahim et al. 2013) are pertinent examples of community projects that operate on a similar strategy. Moreover, frequent versioning ensures that users can return to previous versions to re-run analyses.

Setting up a version-controlled model repository not only allows researchers to publish a 'default' unspecific GEM of the investigated organism, but also reproducible instructions on how to obtain a model that is specific to the organism in a defined experimental context including, and validated against the data supporting this context. This formulaic approach of deriving a GEM into a condition-specific form supports Heavner and Price's (Heavner and Price 2015) call for more transparency and reproducibility in metabolic network reconstruction (25).

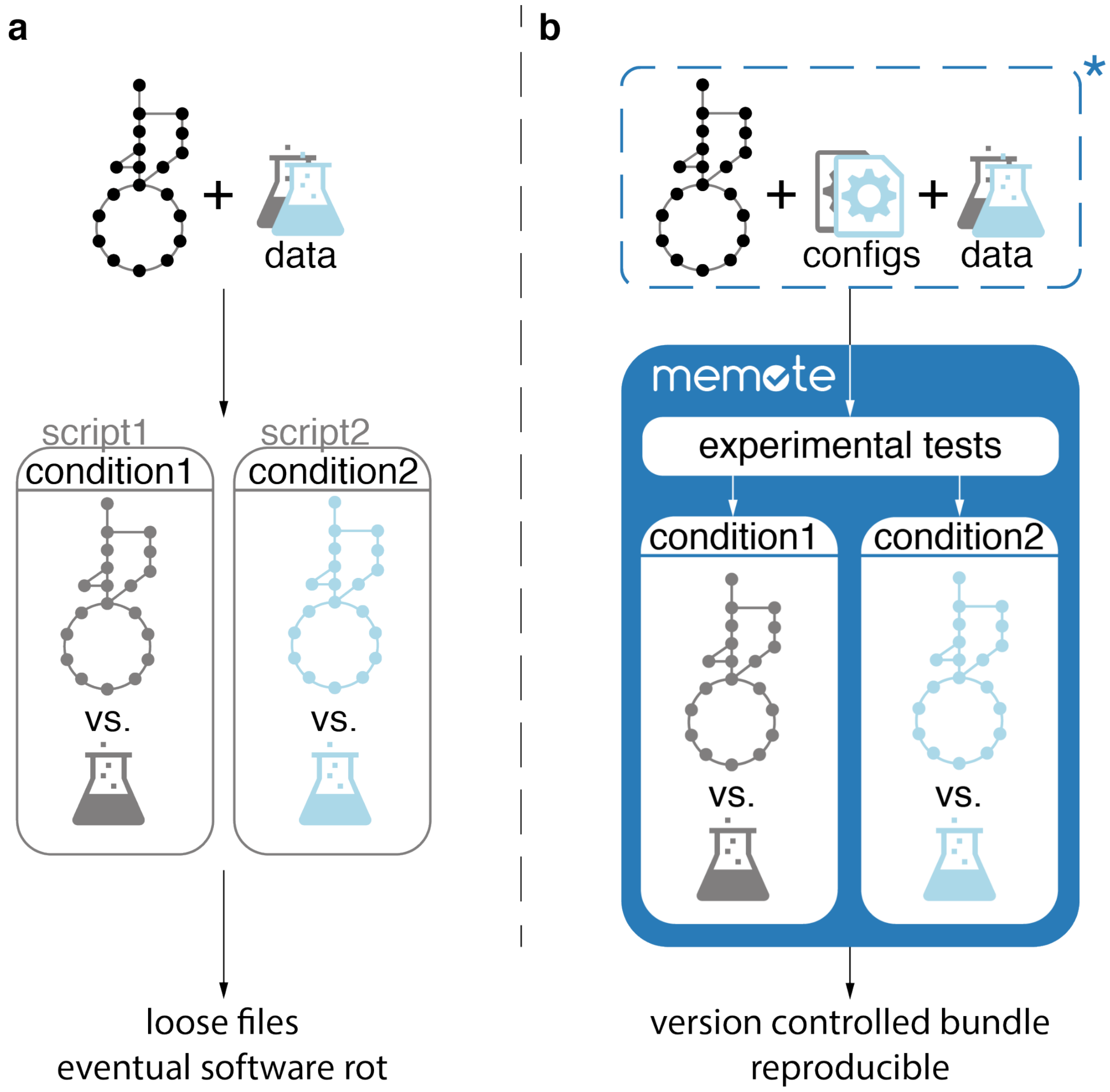

Figure 25: Experimental tests can be tailored to a specific condition through the use of one or several configuration files (configs). (a) To validate GEMs against experimental data measured in specific conditions, researchers usually write their scripts which constrain the model. This is problematic as scripts can vary a lot and they are, unless actively maintained, susceptible to software rot. (b) With memote, user-defined configuration files replace scripts, which allows the experimental validation of GEMs to be unified and formalized. Bundling the model, configuration files, and experimental data within a version-controlled repository (indicated by the blue asterisk*) facilitates reproducibility.

4 Supplementary Note 3: Integration in third party tools and services

Memote's core functions are available through a python API and the online service is available through either a web interface or a programmatic REST API. We have integrated memote in KBase (Arkin et al. 2018) as an app, OptFlux (Rocha et al. 2010) (version 3.4) as a plug-in and link to it from the BiGG Models Database (King et al. 2015). We plan to integrate it with BioModels (Li et al. 2010), and the RAVEN toolbox (Agren et al. 2013).

5 Supplementary Note 4: Discussion of alternatives to memote

The cloud-based, distributed version control for GEMs encoded as SBML3FBC is only one possible implementation approach for version control and collaboration. Alternatives include Pathway Tools (Karp et al. 2009) which internally stores organism data in the form of a database, and AuReMe (Aite et al. 2018), which allows users to interact with a database by wikis. Although databases offer greater capacity and speed than single, large data files, the programmatic or form-based interaction and more complex setup procedure required for databases may not be easily accessible to a broad community. We see Memote in combination with GitHub, GitLab, or BioModels as a means of version control that is simple to set up and easy to manage.

For quality control, alternatives include rBioNet (Thorleifsson and Thiele 2011), an extension to the COBRAToolbox (Heirendt et al. 2017). It primarily focuses on guiding reconstruction by flagging operations which violate SOPs but also provides functions which print basic information such as the amount of model components and dead-end metabolites. Memote may be more widely adopted because no license for MATLAB is required. gsmodutils (Gilbert et al. 2019) is another option but is less accessible to a wider community due to the need for proficiency in Python for use. We note that owing to the exchange format of SBML, memote is fully compatible with rBioNet and gsmodutils.

6 Supplementary Note 5: Outlook

In future, memote could be extended to provide support for tests based on multi-omics data (Hackett et al. 2016). Moreover, to distribute all files of a model repository together, the model, supporting data and scripts could be automatically bundled into one ZIP-based archive file (so-called COMBINE archive) (Bergmann et al. 2014). These archives can include a formal description of simulation experiments to ensure exchangeability and reproducibility (Waltemath et al. 2011).

The tests that memote offers only apply to stoichiometric models. However, the underlying principles behind memote could be applied to other modeling paradigms, i.e., to models of metabolism and expression (ME-models) (OBrien et al. 2013), kinetic (Vasilakou et al. 2016), or even systems pharmacological models (Thiel et al. 2017).

7 Supplementary Methods

To simplify interpretation, the following figures are grouped by the sections of their corresponding test cases as they appear in a snapshot report. The code that was used to generate the data and figures has been deposited on GitHub https://github.com/biosustain/memote-meta-study.

7.1 Tested models

We tested models from seven GEM collections comprising manually and (semi)-automatically reconstructed GEMs (10,780 models in total): (i) 801 semi-automatically built reconstructions of human gut bacteria from the AGORA (Magnúsdóttir et al. 2016) collection (version 1.03; not condition-specific and including post-publication corrections (Babaei et al. 2018), (Magnúsdóttir et al. 2018)), (ii) 2,641 models from the Path2Models (Büchel et al. 2013) branch of the BioModels (Li et al. 2010), (Novere 2006), (Chelliah et al. 2014) database hosting models automatically generated from pathway resources, and (iii) 5,511 and (iv) 1,632 models automatically reconstructed using CarveME (Machado et al. 2018) and the Department of Energy's Knowledge Base (KBase) (Arkin et al. 2018) based on bacterial genomes in NCBI RefSeq, respectively. Furthermore, 36 manually reconstructed models from the (v) the BiGG25 database and two collections of published models as available from (vi) Ebrahim et al. (Ebrahim et al. 2015) (80 models) and (vii) the OptFlux (Rocha et al. 2010) software (79 models), of which 39 models are likely identical based on a filename comparison

Two collections contained models in non-standard formats that were omitted entirely (15 from Ebrahim et al and 49 from OptFlux)

In order to respect the limited resources on the DTU high performance computing infrastructure, we set a maximum time limit for running the memote test suite. This introduced a bias against large models. Additionally, certain models failed the testing procedure. In the following we tabulate the total size of the collections as well as the final number of tested models. The results are shown in Table 1.

7.2 Clustering

In order to perform the clustering analyses, we used all normalized test metrics excluding some particular cases. Excluded are the Sections 7.3.4.2 & 7.3.4.6 because the basic information only contains unnormalized model dimensions and because a biomass formulation is not present in all models. We further removed individual biomass related test cases, as well as the metabolic coverage since that is not properly normalized. Additionally, test cases that contained errors were penalized with the worst metric of one.

To determine the most relevant tests to discriminate between model collections, we built a classifier using a random forest (Breiman 2001) over the collections and normalized test results (0.99 accuracy and 0.01% out-of-bag (OOB) error). Then, the importance of each variable, i.e., test case, was ranked with the Mean Decrease in Accuracy (MDA) (Louppe et al. 2013). This metric measures the total decrease in accuracy, averaged over all trees of the forest, when the value of a given variable is permuted in the OOB samples. Figure 29 represents the 15 most discriminant features on average (see last column) and their independent relevance by collection. The higher the decrease

Table 1: Number of tested models.

Collection	Number of Models	Tested Models	%
AGORA	818	801	97.9
CarveMe	5587	5511	98.6
Path2Models	2641	2641	100.0
KBase	1637	1632	99.7
BiGG*	36	36	100.0
Ebrahim *et al.*†	83	80	96.4
OptFlux Models†	100	79	79.0

* Please note that we removed the large number of *Escherichia coli* strain models from the BiGG collection and only included results from the models iJR904, iAF1260, iJO1366, and iML1515.

† 39 models from these two collections are likely identical based on a filename comparison.

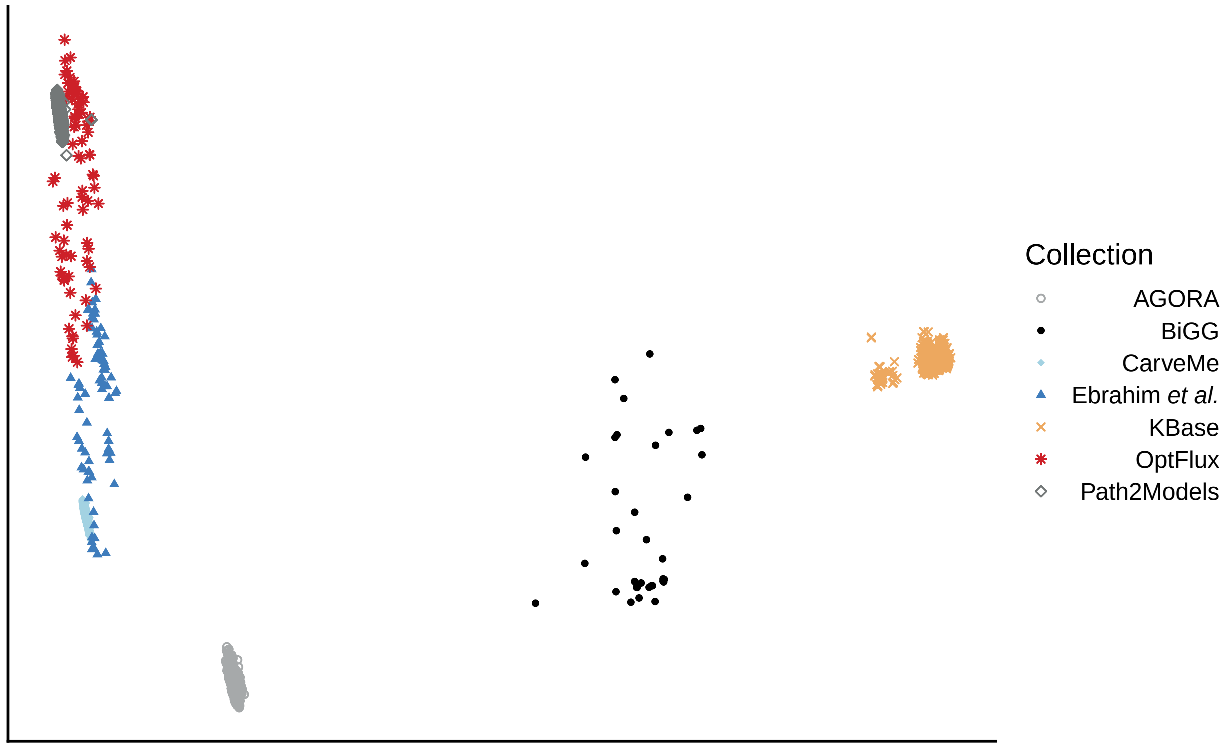

Figure 26: Depicted are the first two components of a principal components analysis of the normalized test features (metrics).

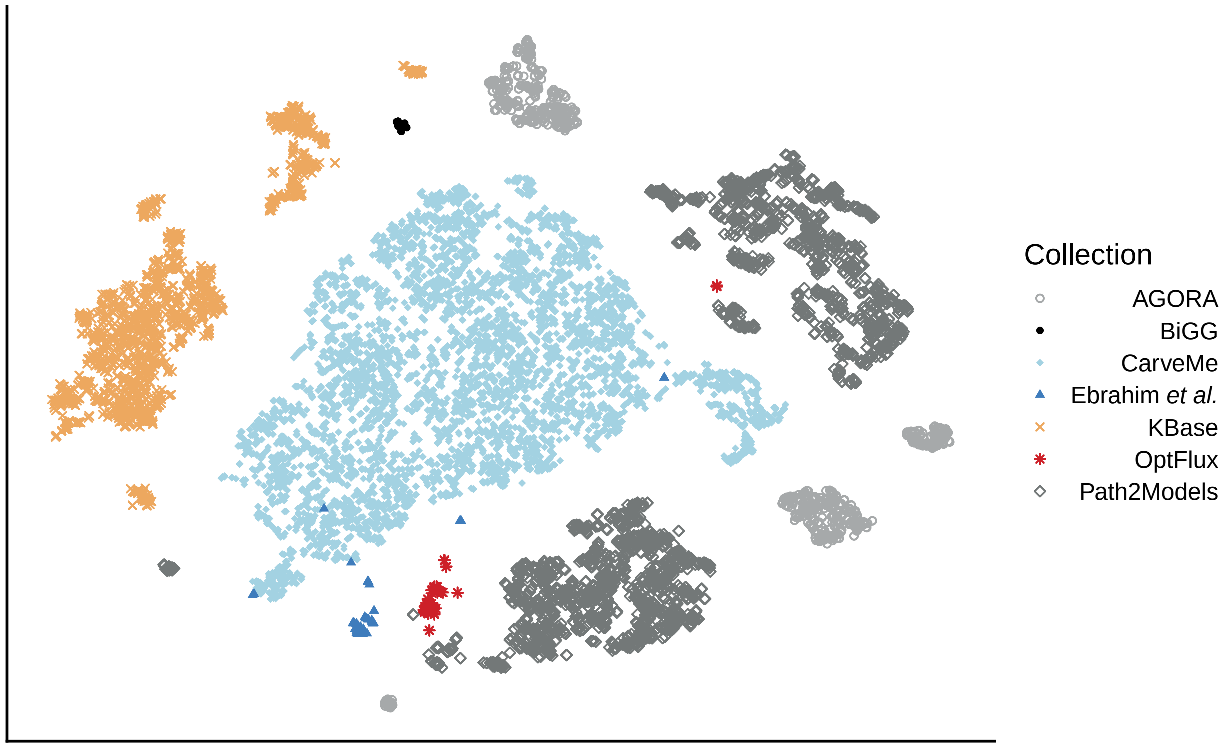

Figure 27: Depicted are the distances between models in higher order space given by the normalized test features reduced to two dimensions using t-SNE.

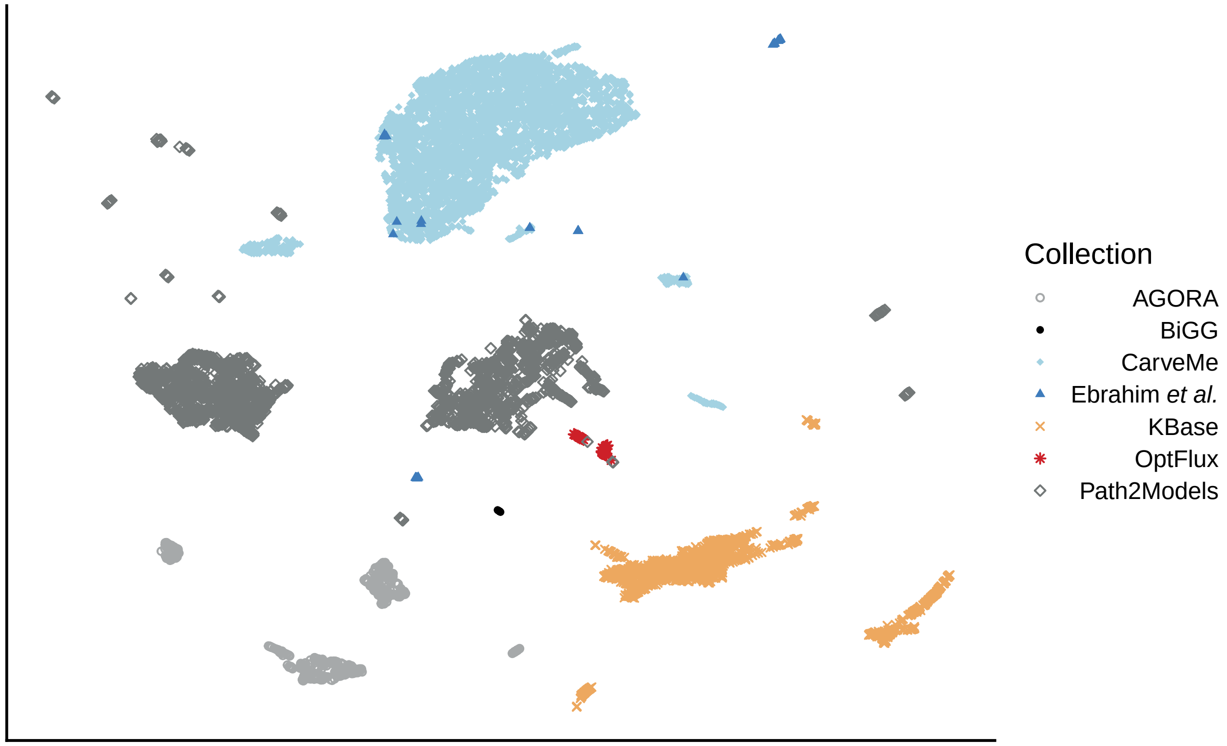

Figure 28: Depicted are the distances between models in higher order space given by the normalized test features reduced to two dimensions using UMAP.

in accuracy, the higher the relative contribution of such a test to differentiate among collections. Thus, the five most discriminant tests are purely metabolic reactions, transport reactions, dead-end metabolites, orphan metabolites, and the presence of a non-growth associated maintenance reaction. Although there is a variable range of importance for each collection, e.g., for CarveMe transport reactions and orphans are more relevant; for Kbase transport reactions; for Ebrahim *et al.* purely metabolic reactions. For a detailed study of the clustering properties, please refer to the *Supplementary Clustering Analysis* notebook.

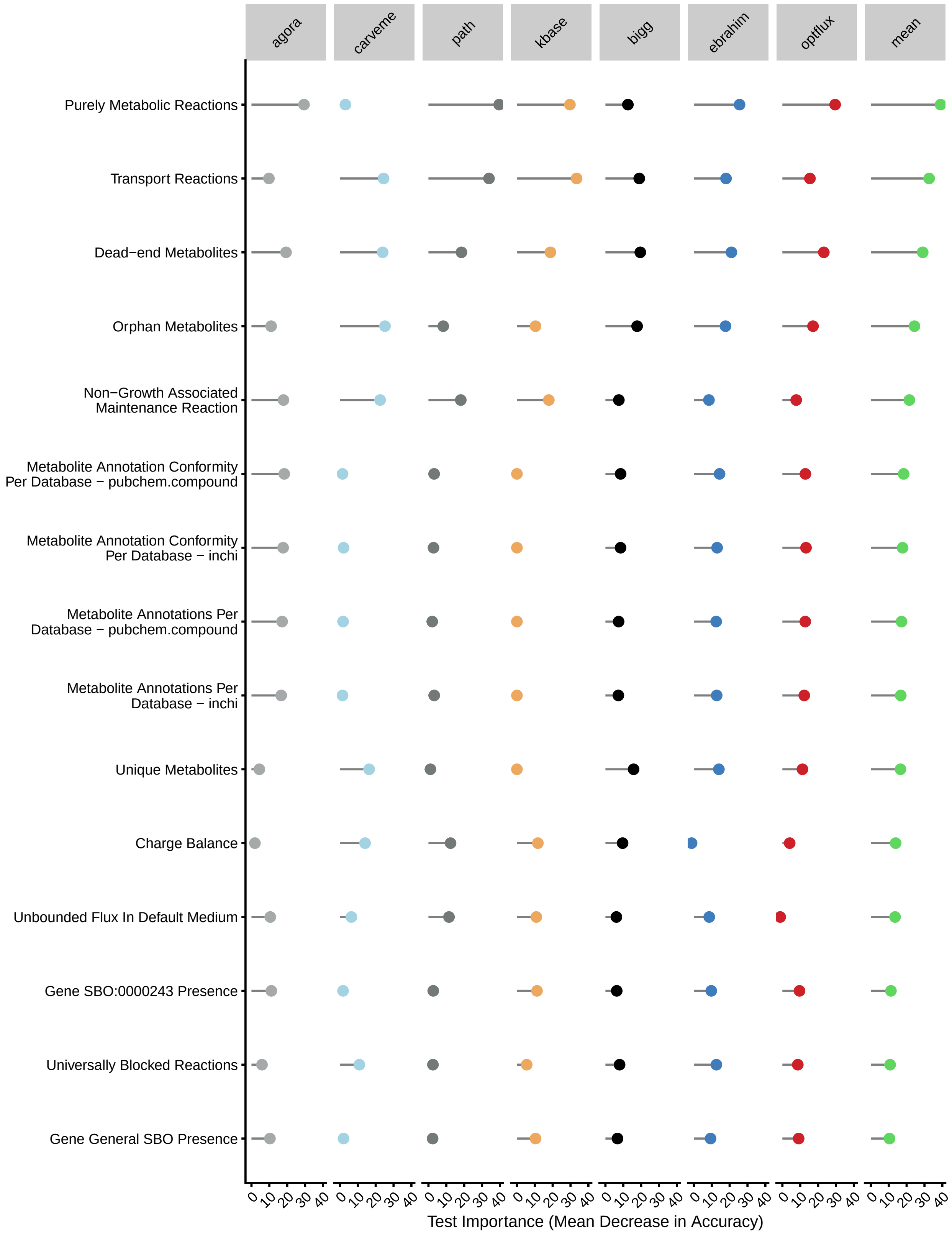

Figure 29: 15 most relevant tests to discriminate among GEM collections, for each collection and the mean. Ranked in decreasing importance according to the *mean decrease in accuracy* metric averaged over all collections (last column), computed over a random forest classification model.

7.3 Test Suite

The database identifiers referenced throughout the Annotation sections belong to common biochemical databases that are listed in Table 2.

Table 2: Biochemical Databases for Model Component Annotation.

Databases	Component Type	URL	Citation
ASAP	gene	http://asap.ahabs.wisc.edu/asap/home.php	(Glasner 2003)
BiGG	reaction, metabolite	http://bigg.ucsd.edu/universal/	(King et al. 2015)
BioCyc	reaction, metabolite	http://biocyc.org	(Caspi et al. 2009)
BRENDA	reaction	http://www.brenda-enzymes.org/	(Jeske et al. 2018)
CCDS	gene	http://www.ncbi.nlm.nih.gov/CCDS/	(Pujar et al. 2017)
ChEBI	metabolite	https://www.ebi.ac.uk/chebi/	(Hastings et al. 2015)
EC-Code	reaction	http://www.enzyme-database.org/	(McDonald, Boyce, and Tipton 2009)
EcoGene	gene	http://ecogene.org/	(Zhou and Rudd 2012)
HMDB	metabolite	http://www.hmdb.ca/	(Wishart et al. 2017)
HPRD	gene	http://www.hprd.org/	(Prasad et al. 2009)
InChI	metabolite	https://www.ebi.ac.uk/chebi/	(Stein, Heller, and Tchekhovskoi 2003)
InChIKey	metabolite	http://cactus.nci.nih.gov/chemical/structure	N/A
Kegg	gene, reaction, metabolite	http://www.kegg.jp/	(Kanehisa 2019)
MetaNetX	reaction, metabolite	http://www.metanetx.org	(Moretti et al. 2015)
NCBI Gene	gene	http://ncbigene.bio2rdf.org/fct	(Richa Agarwala et al. 2017)
NCBI GI	gene	http://www.ncbi.nlm.nih.gov/protein/	(Richa Agarwala et al. 2017)
NCBI Protein	gene	http://www.ncbi.nlm.nih.gov/protein	(Richa Agarwala et al. 2017)
PubChem	metabolite	https://pubchem.ncbi.nlm.nih.gov/	(Richa Agarwala et al. 2017)
Reactome	reaction, metabolite	http://www.reactome.org/	(Fabregat et al. 2017)
RefSeq	gene	http://www.ncbi.nlm.nih.gov/projects/RefSeq/	(Richa Agarwala et al. 2017)
RHEA	reaction	http://www.rhea-db.org/	(Morgat et al. 2016)
SEED	metabolite	http://modelseed.org/	(Henry et al. 2010)
Uniprot	gene	http://www.uniprot.org/	(Consortium 2018)

7.3.1 Summary of Observations

- SBO terms are only used by models from KBase and BiGG (Figure 105).
- Models from Path2Models and Opflux Models are formatted in legacy SBML (< Level 3,Version 1) without FBC package (Figures 117 & 118).
- Models from the collections of Ebrahim *et al.*, and OptFlux Models are highly variable for many specific tests. Models from automatic reconstruction pipelines (AGORA, CarveMe, Path2Models, and KBase) or the controlled BiGG collection are much more similar within each collection yet still different from each other. This could be due to each collection focusing on a distinct set of taxonomies but could also be related to the algorithms and databases behind each collection (Section 7.3.4.8; Figures 130, 132, and 138).
- On biomass:
  - Only for a minority of models in BiGG, Ebrahim *et al.*, and OptFlux Models memote could not identify a biomass reaction (Figure 142).
  - A portion of models in the BiGG collections have inconsistent biomass equations followed by OptFlux Models and models in the collection by Ebrahim *et al.*; all models in the CarveMe and Path2Model collections have inconsistent biomass reactions (Figure 143).
  - Models that cannot be simulated using the default or complete medium exist in Path2Models, BiGG, Ebrahim *et al.*, and OptFlux Models (Figure 144 & 145).
  - Possible artifacts from automatic reconstruction are present in models from AGORA and KBase that grow despite some biomass precursors being blocked when each precursor is optimized individually in default and complete medium (compare Figures 146 & 147 with 144 & 145).
- The average fraction of reactions that participate in stoichiometrically-balanced cycles is larger for models from automatic reconstruction pipelines (AGORA, CarveMe, Path2Models, KBase) than for BiGG, Ebrahim *et al.*, and OptfluxModels (Figure 156). This could be an artifact from automatic reconstruction processes.
- Reactions that involve oxygen are integral to the energy metabolism of many organisms. Not constraining these reactions carefully can lead to predictions that deviate from the expected phenotype, i.e., allowing anaerobic growth that should not be possible. The portion of oxygen-containing reactions that are reversible varies strongly across all seven collections. Models in BiGG have the lowest variance whereas models from Path2Models, Ebrahim *et al.*, and OptFlux vary strongly (Figure 152).

7.3.2 Scores

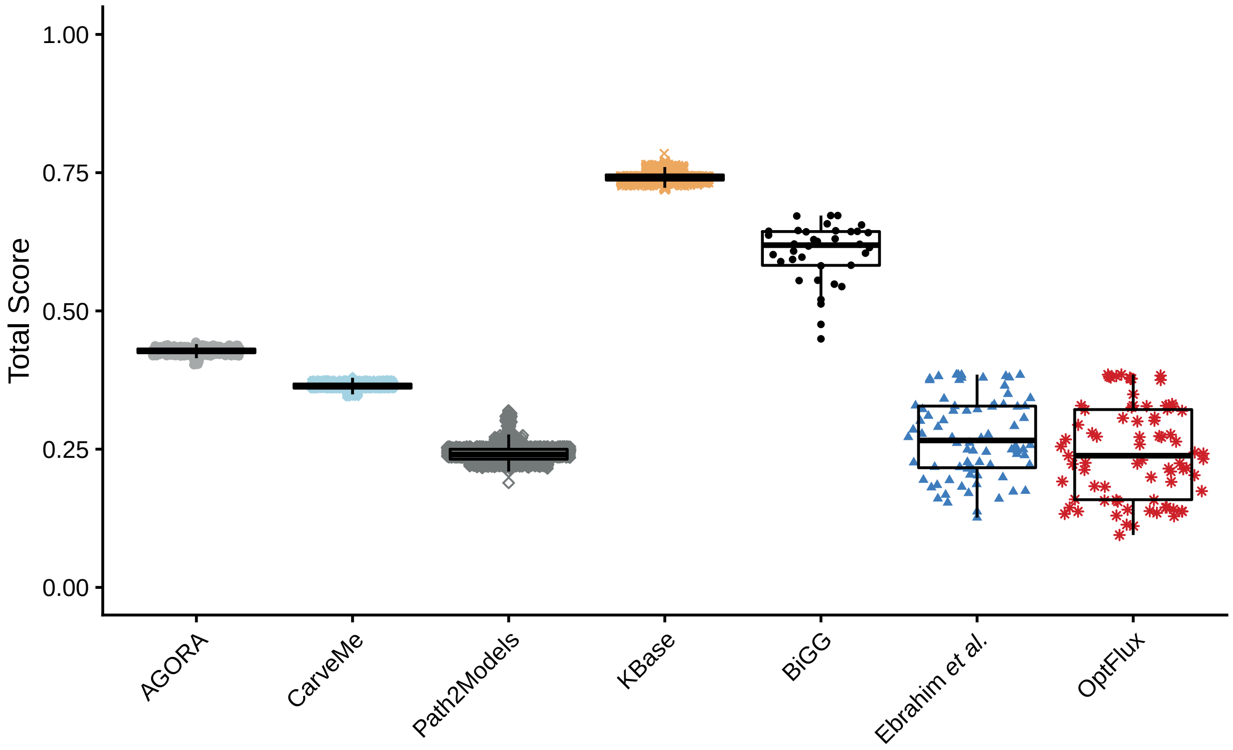

Figure 30: Total Score. Depicted are the sums of all test scores in all independent sections, applying the weights for individual test cases and sections as detailed in the snapshot report.

7.3.3 Independent Section

7.3.3.1 Consistency

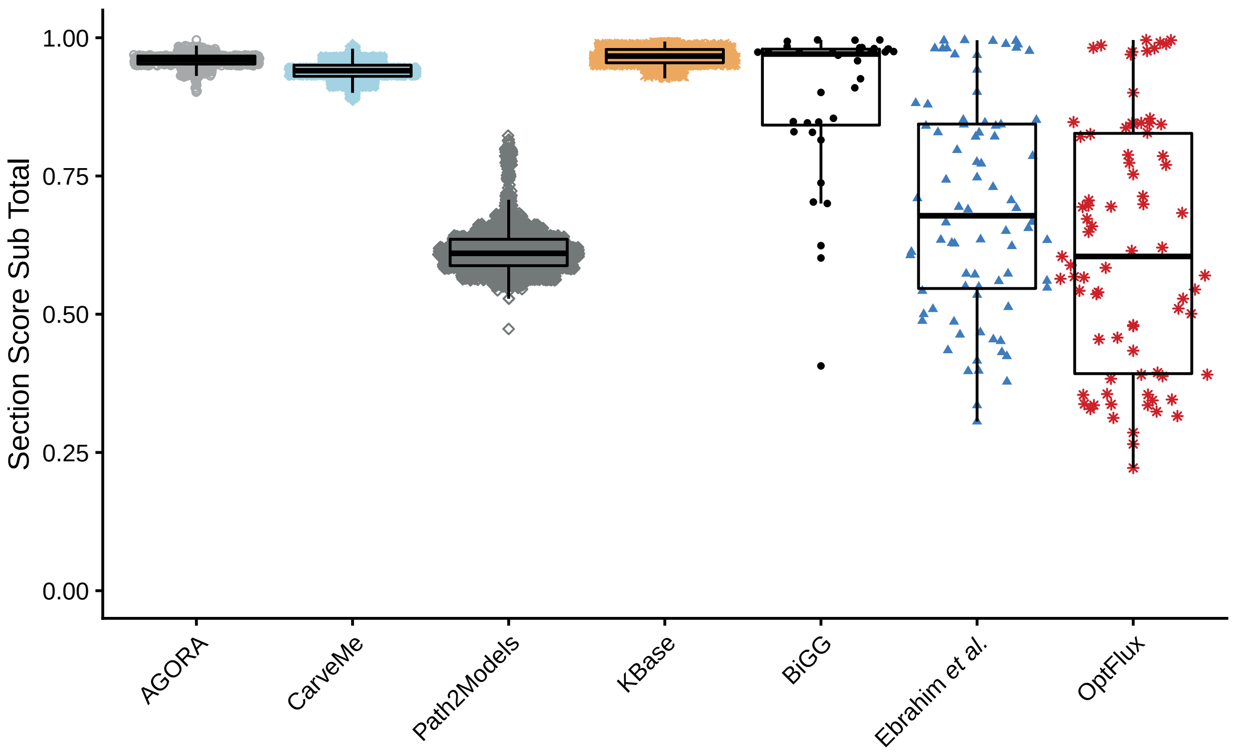

Figure 31: Consistency. Depicted are the sums of all test scores in this section, applying the weights of the individual test cases as detailed in the snapshot report.

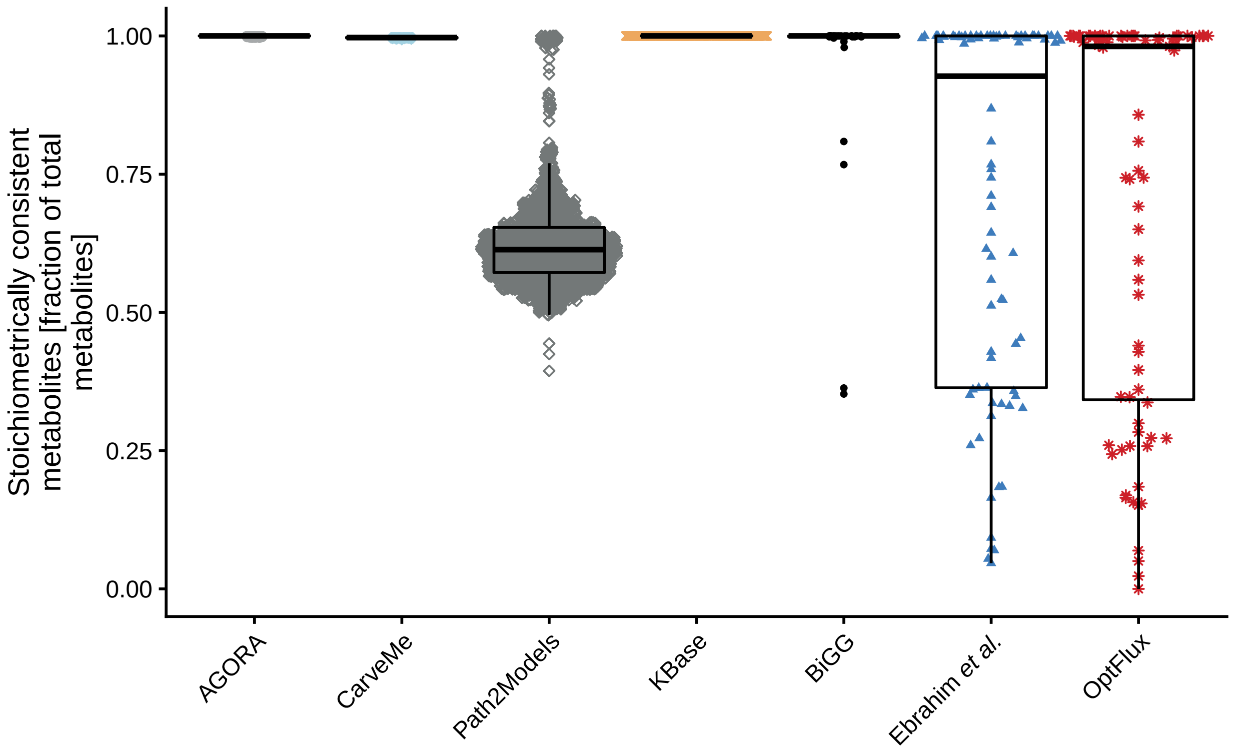

Figure 32: Stoichiometric consistency

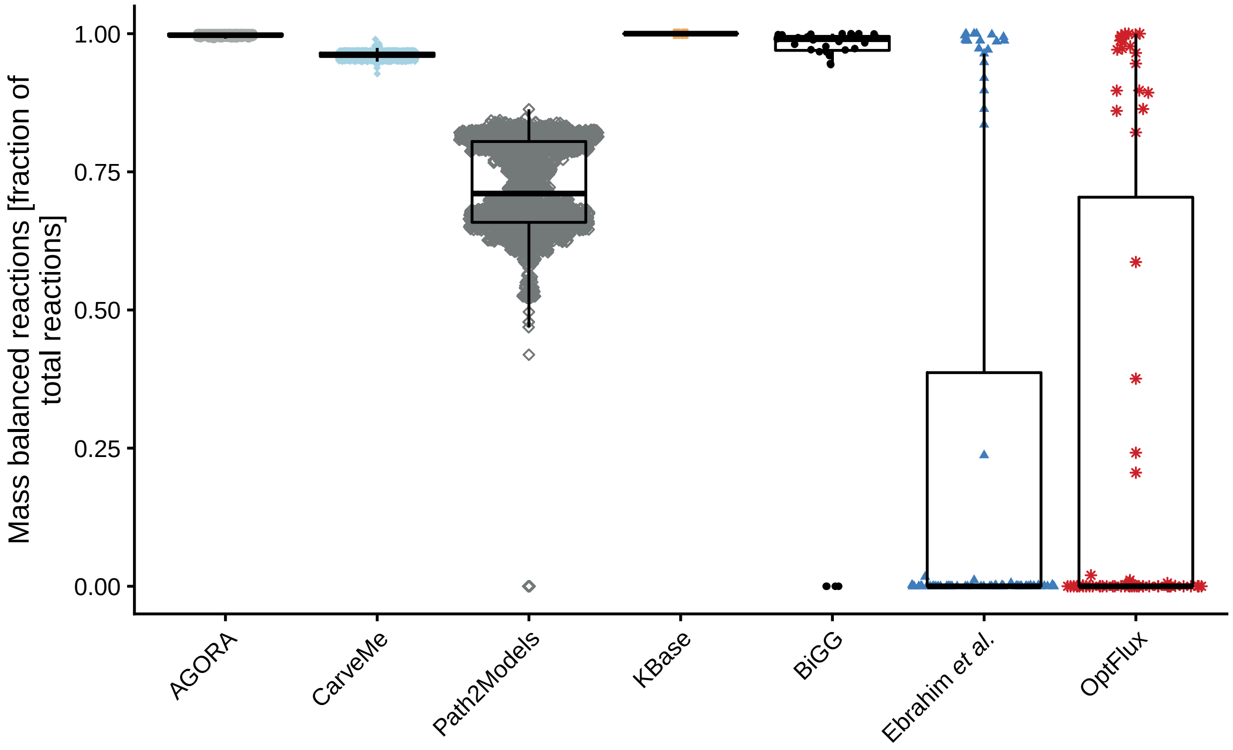

Figure 33: Mass Balance. Please note that any reaction where at least one metabolite lacks a formula annotation is considered as unbalanced for the purpose of this test.

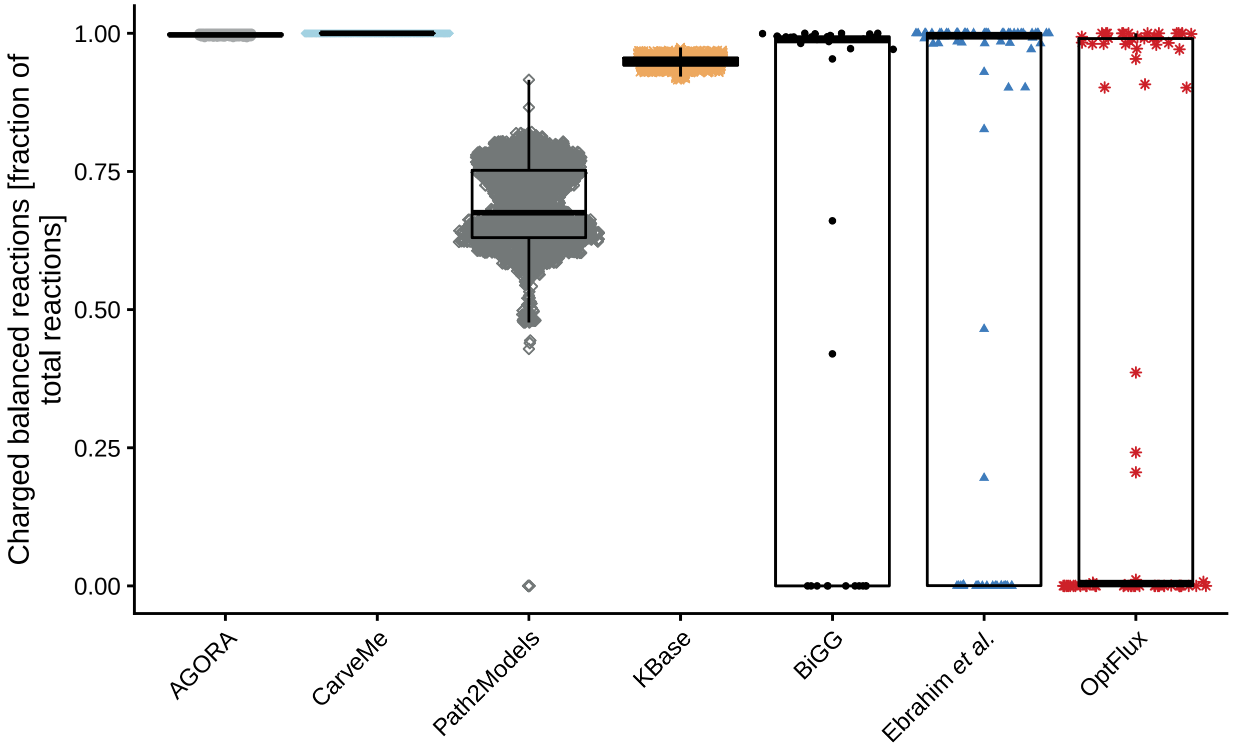

Figure 34: Charge Balance. Please note that any reaction where at least one metabolite lacks charge information is considered as unbalanced for the purpose of this test.

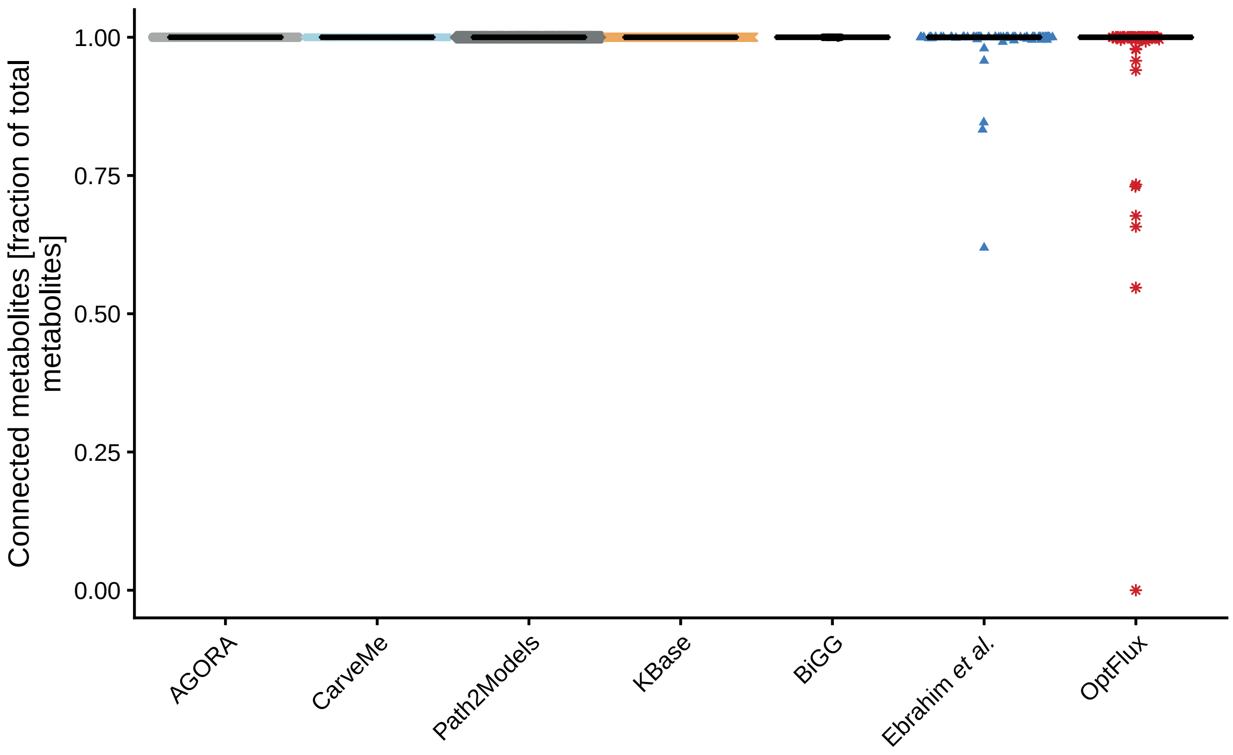

Figure 35: Metabolite Connectivity

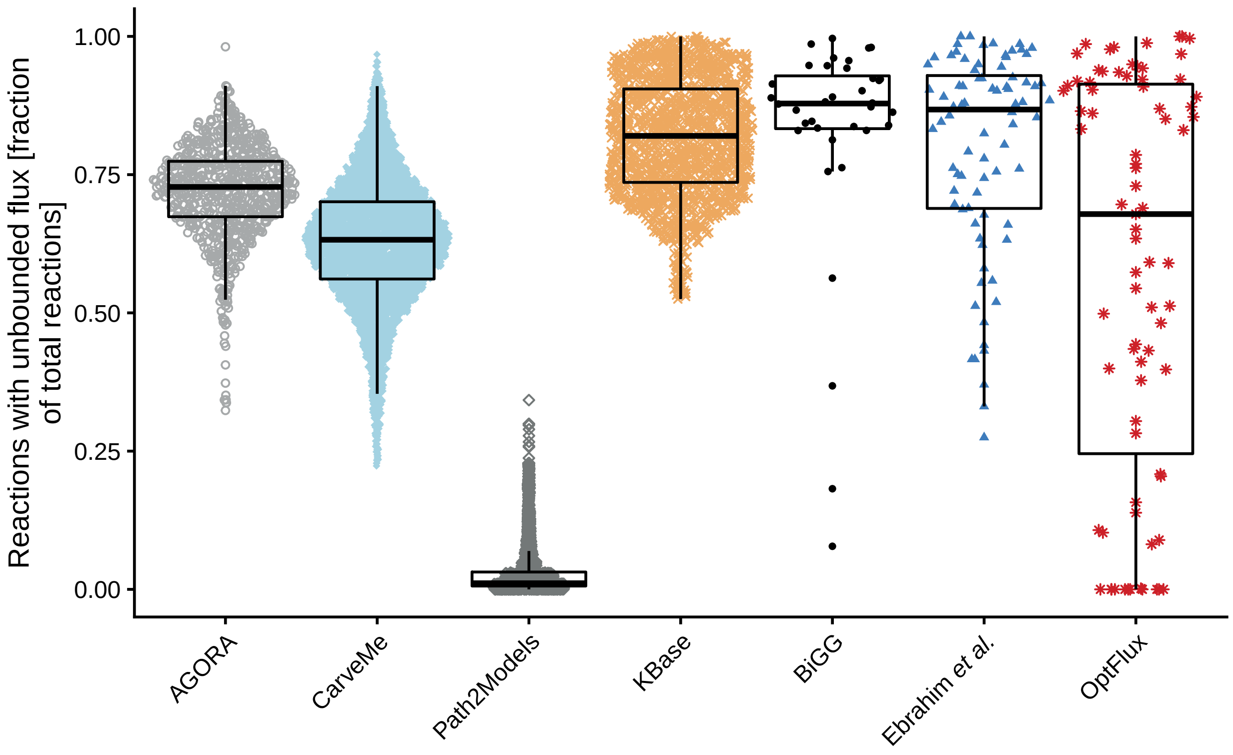

Figure 36: Unbounded Flux in Default Medium

7.3.3.2 Annotation - Metabolites

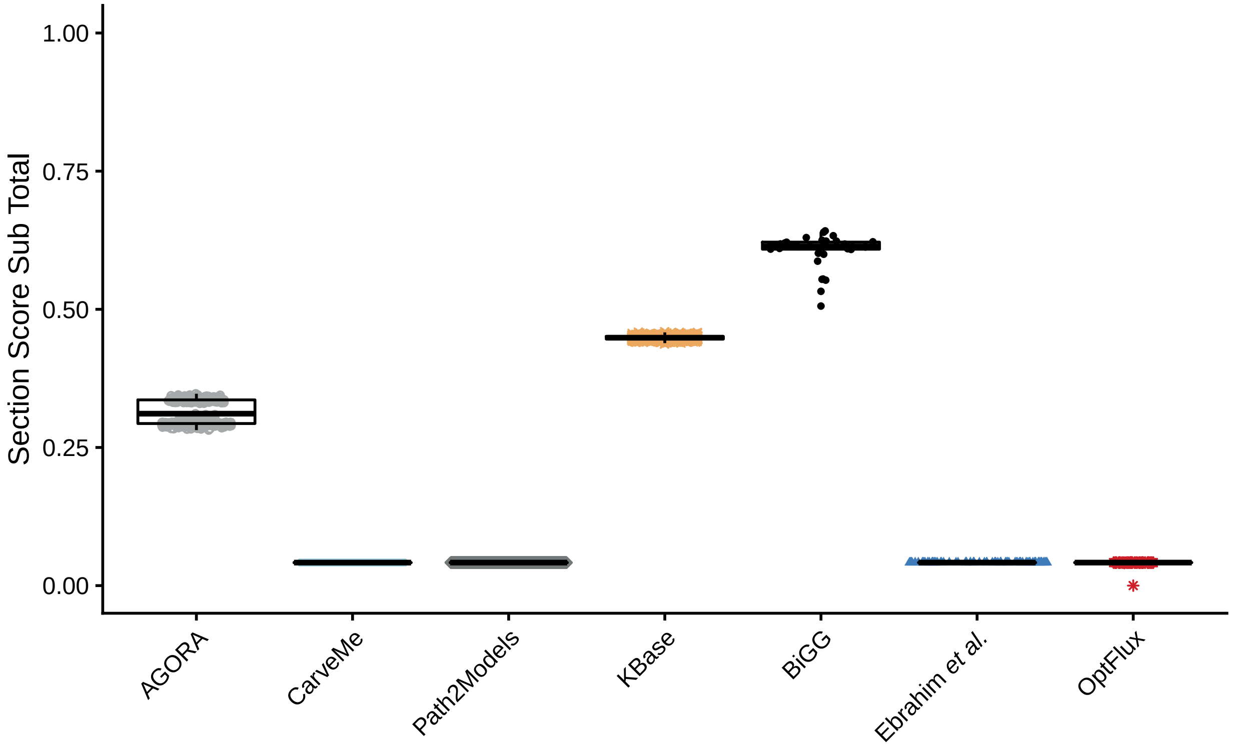

Figure 37: Annotation - Metabolites. Depicted are the sums of all test scores in this section, applying the weights of the individual test cases as detailed in the snapshot report.

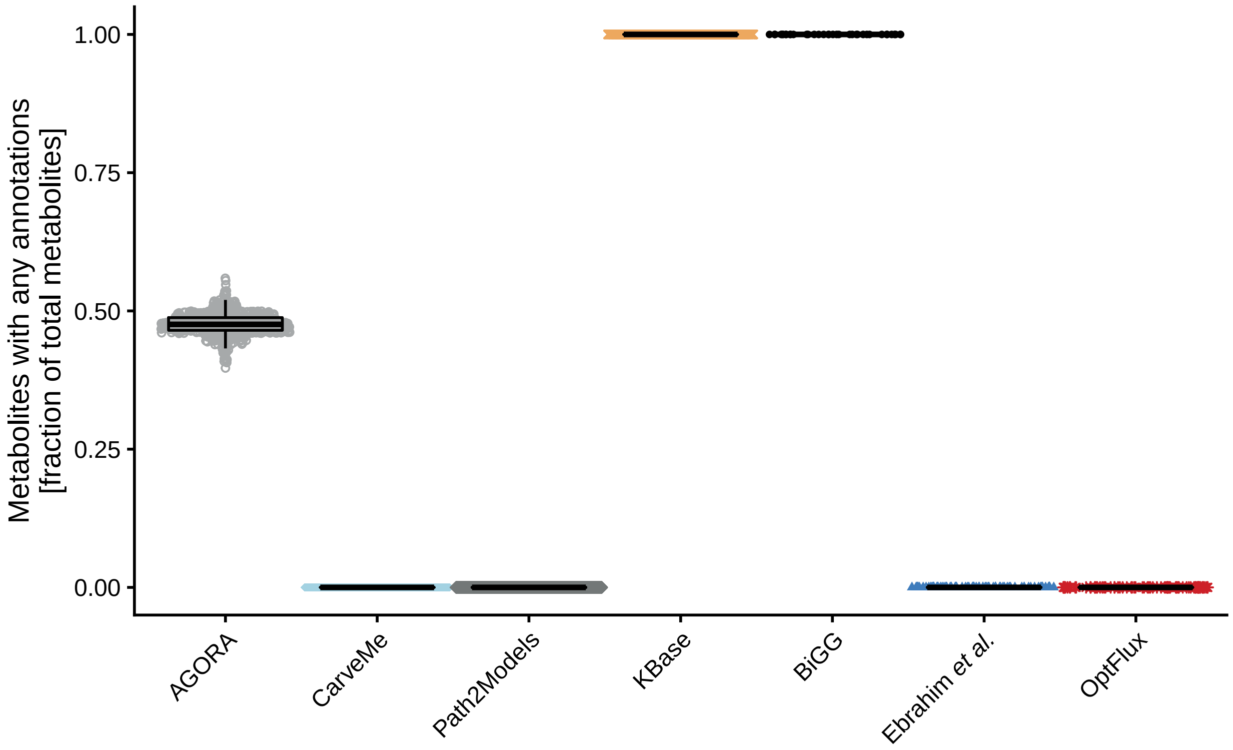

Figure 38: Presence of Metabolite Annotation

7.3.3.2.1 Metabolite Annotations Per Database

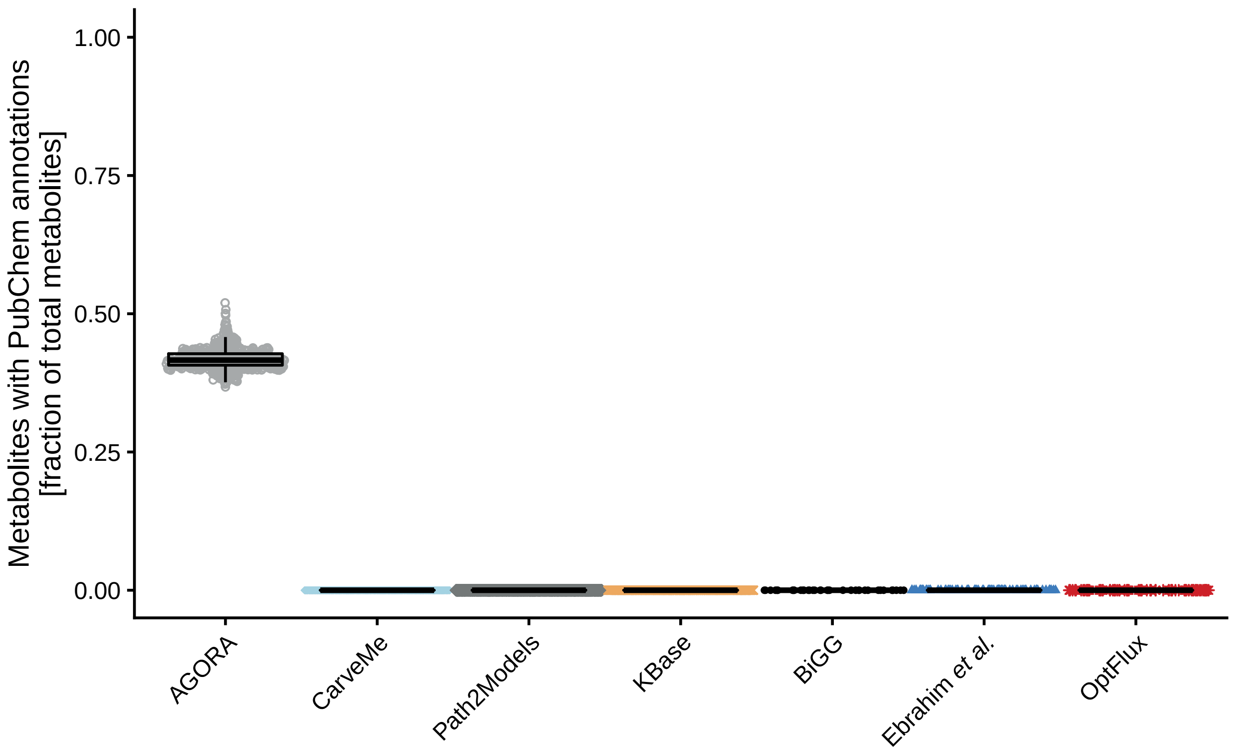

Figure 39: Metabolite Pubchem.compound Annotation

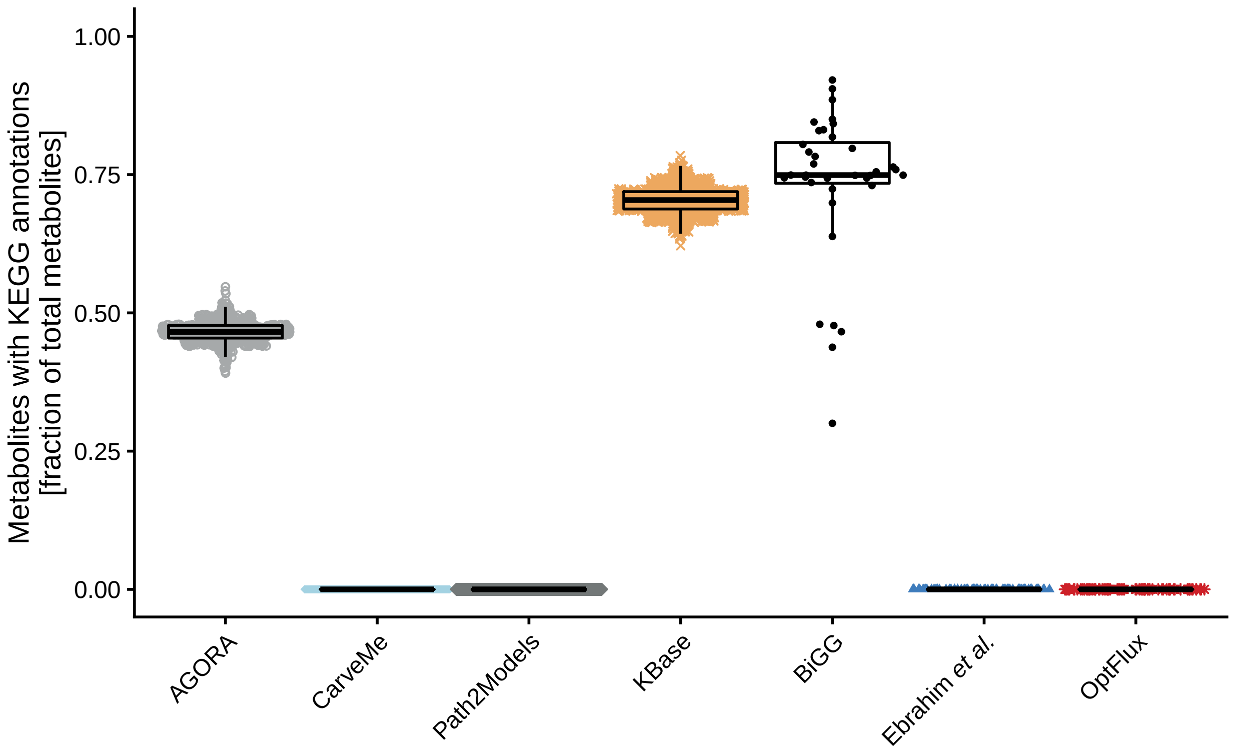

Figure 40: Metabolite KEGG.compound Annotation

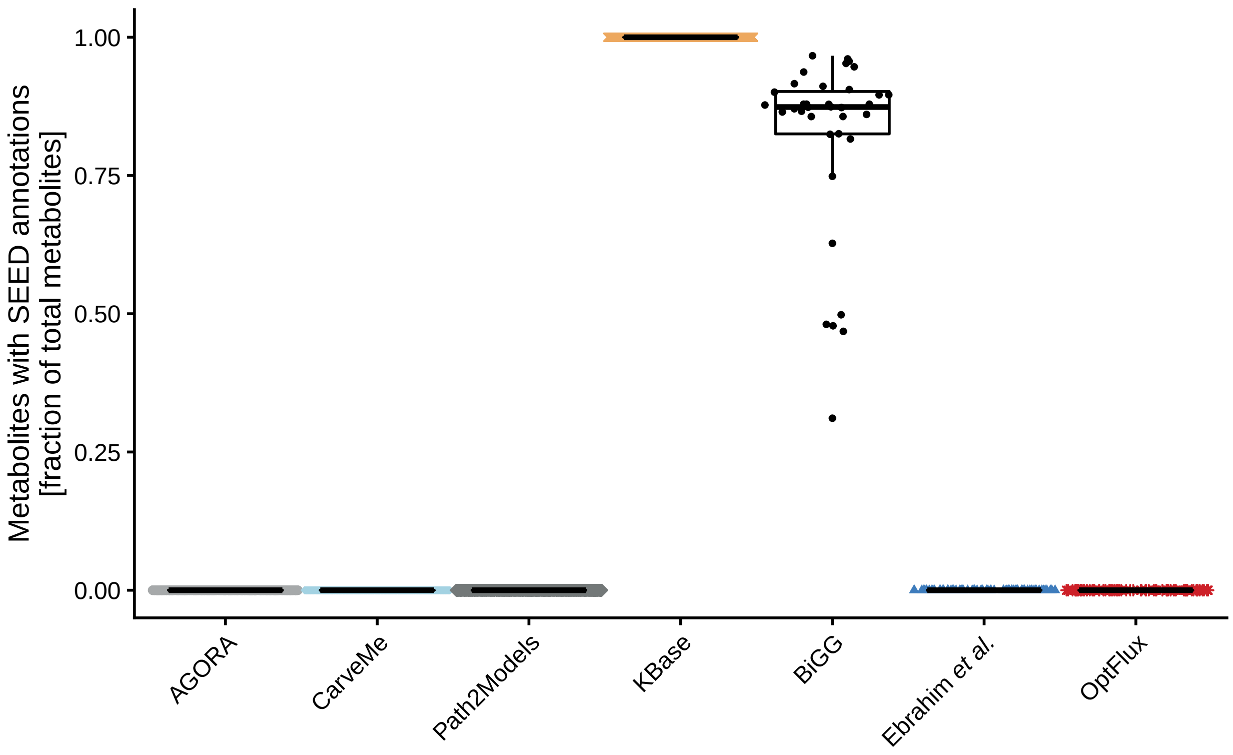

Figure 41: Metabolite SEED.compound Annotation

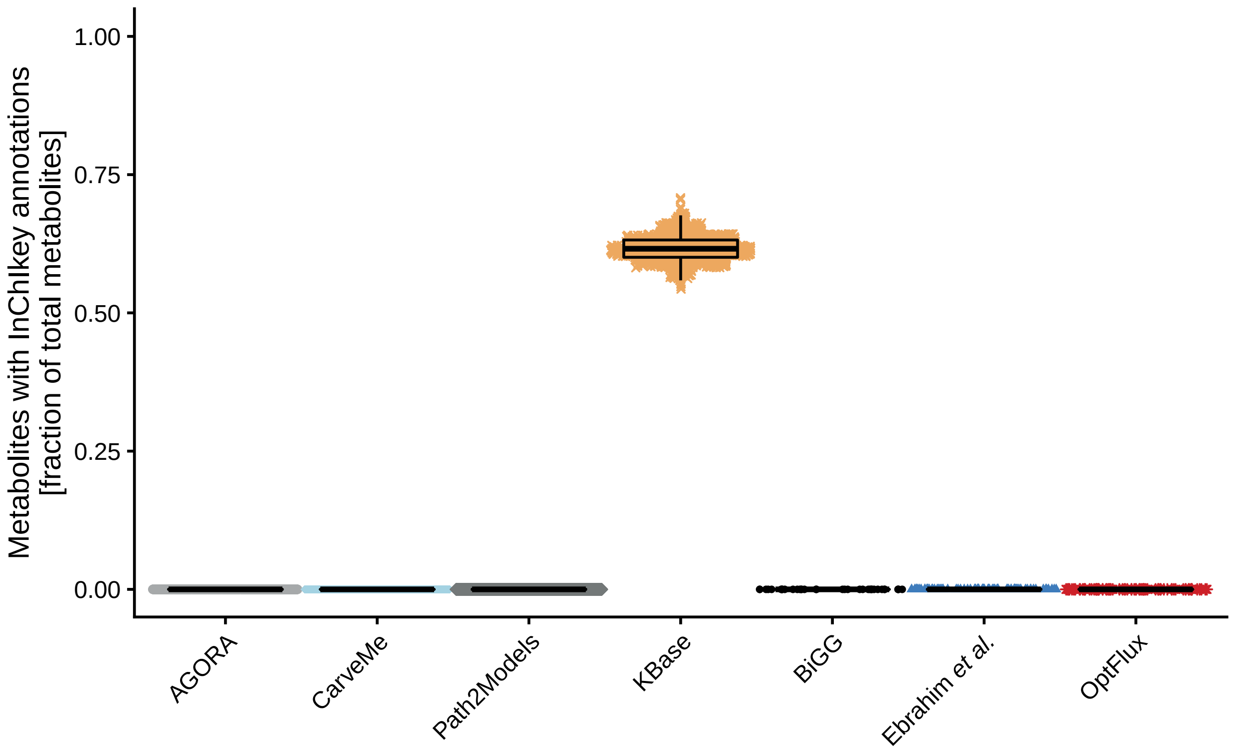

Figure 42: Metabolite InChIKey Annotation

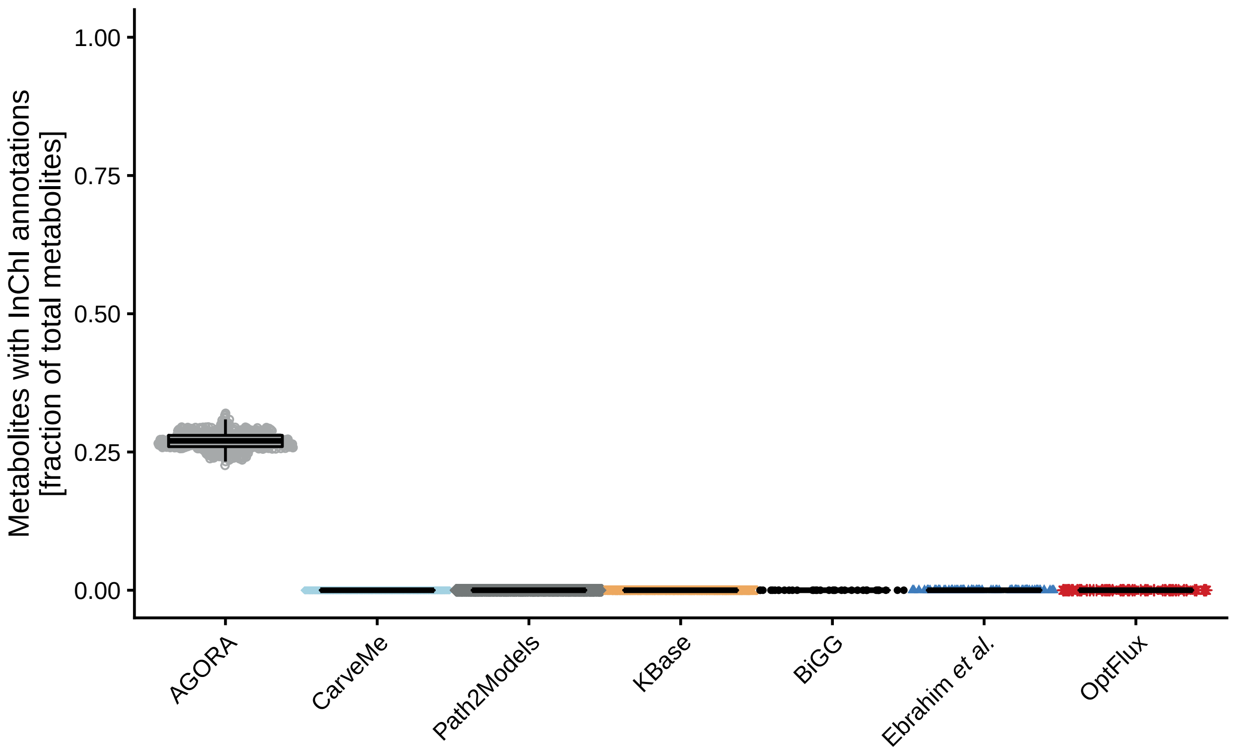

Figure 43: Metabolite InChI Annotation

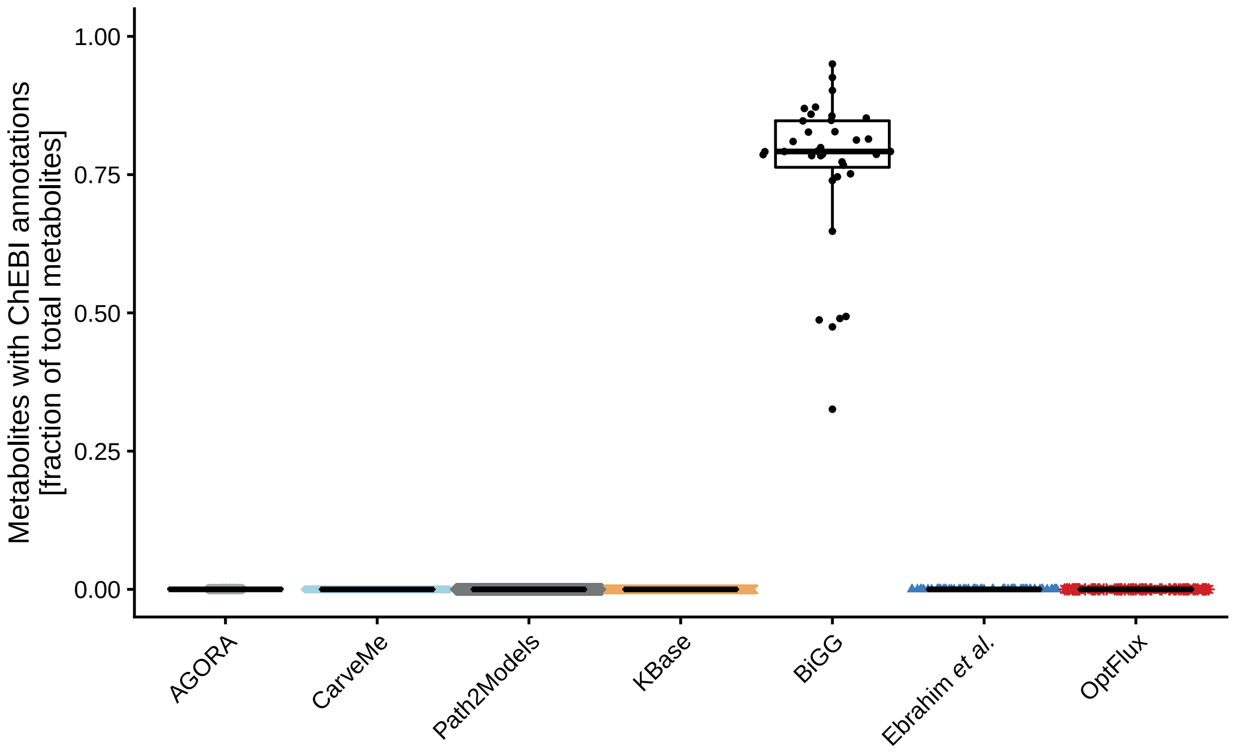

Figure 44: Metabolite ChEBI Annotation

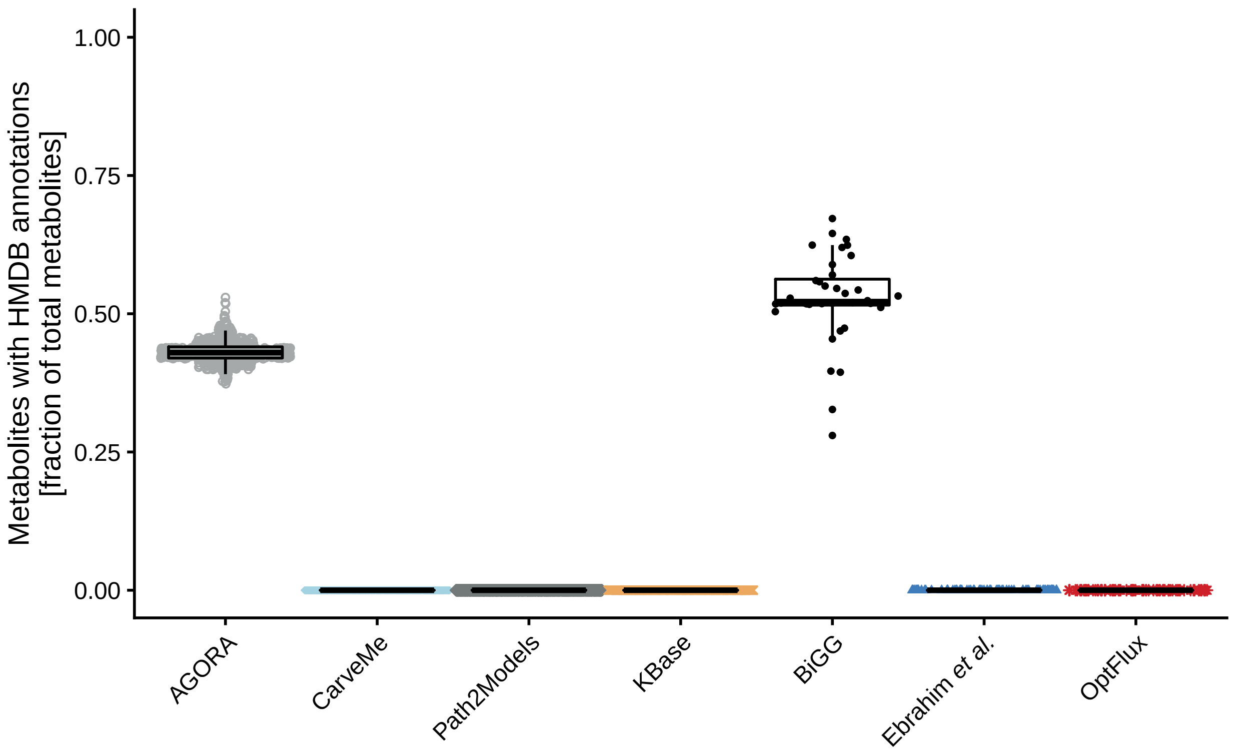

Figure 45: Metabolite HMDB Annotation

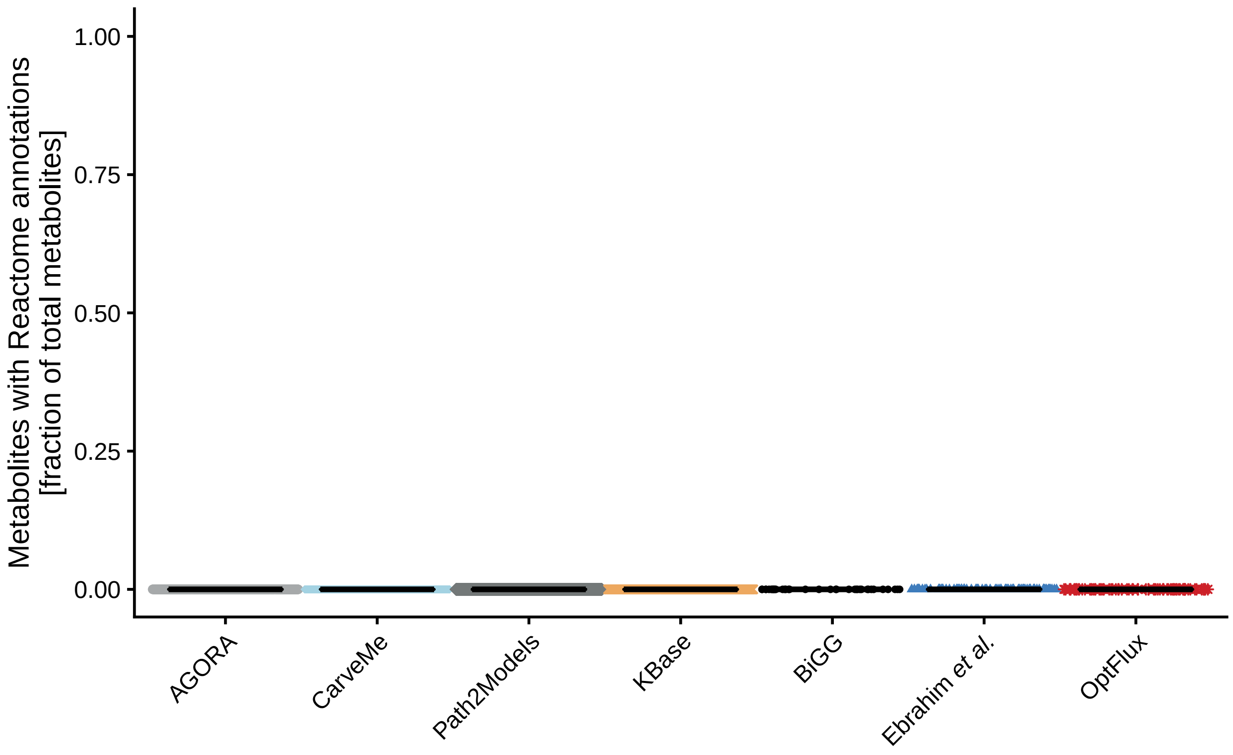

Figure 46: Metabolite Reactome Annotation

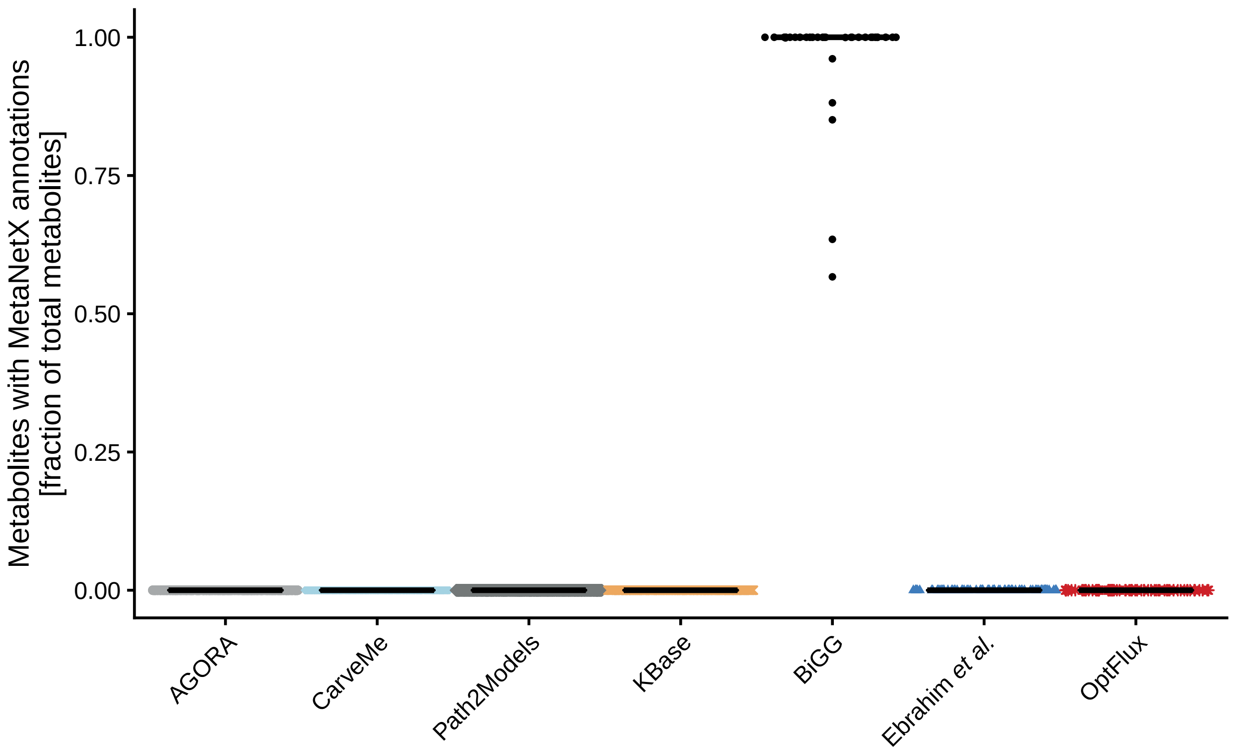

Figure 47: Metabolite MetaNetX.chemical Annotation

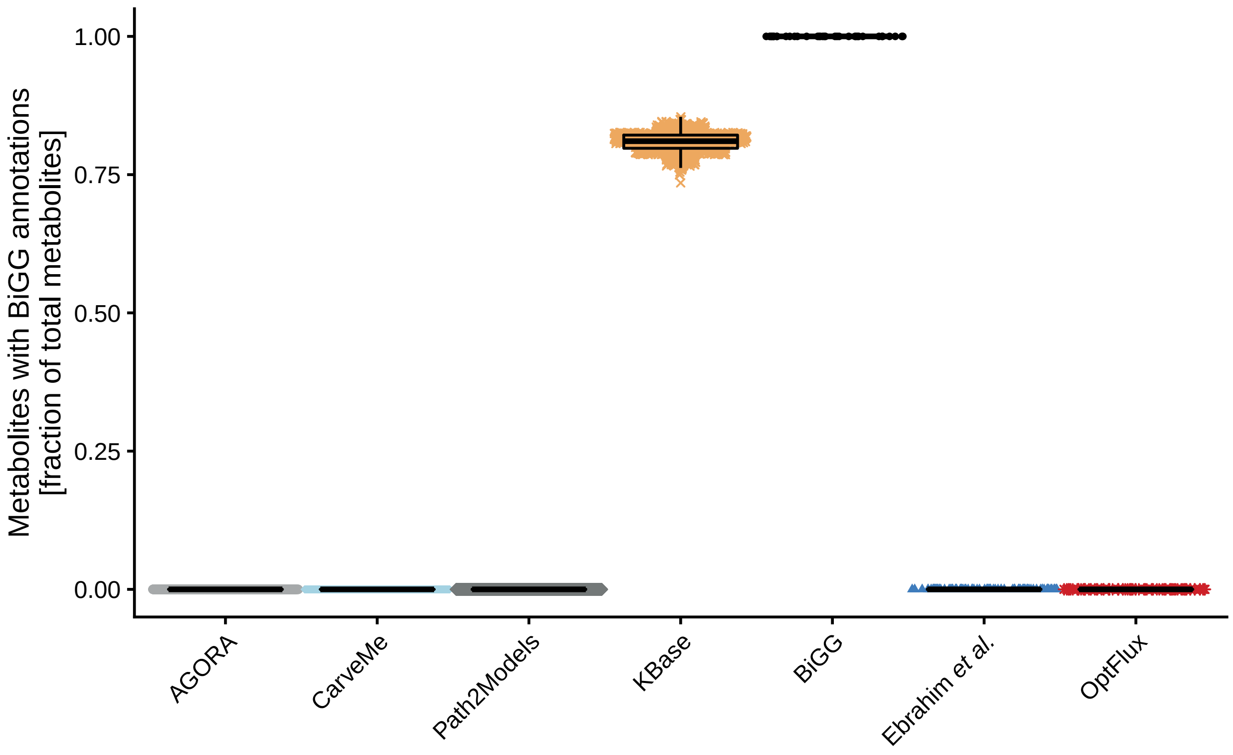

Figure 48: Metabolite BiGG.metabolite Annotation

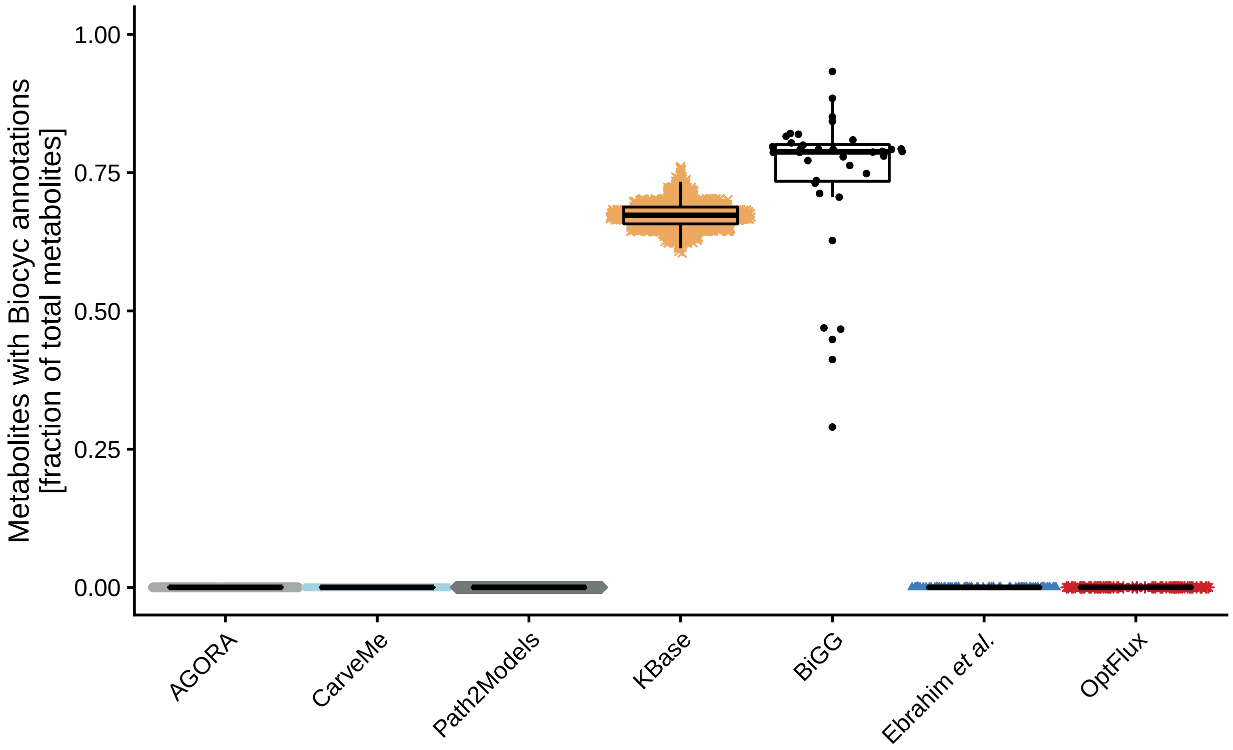

Figure 49: Metabolite BioCyc Annotation

7.3.3.2.2 Metabolite Annotation Conformity per Database

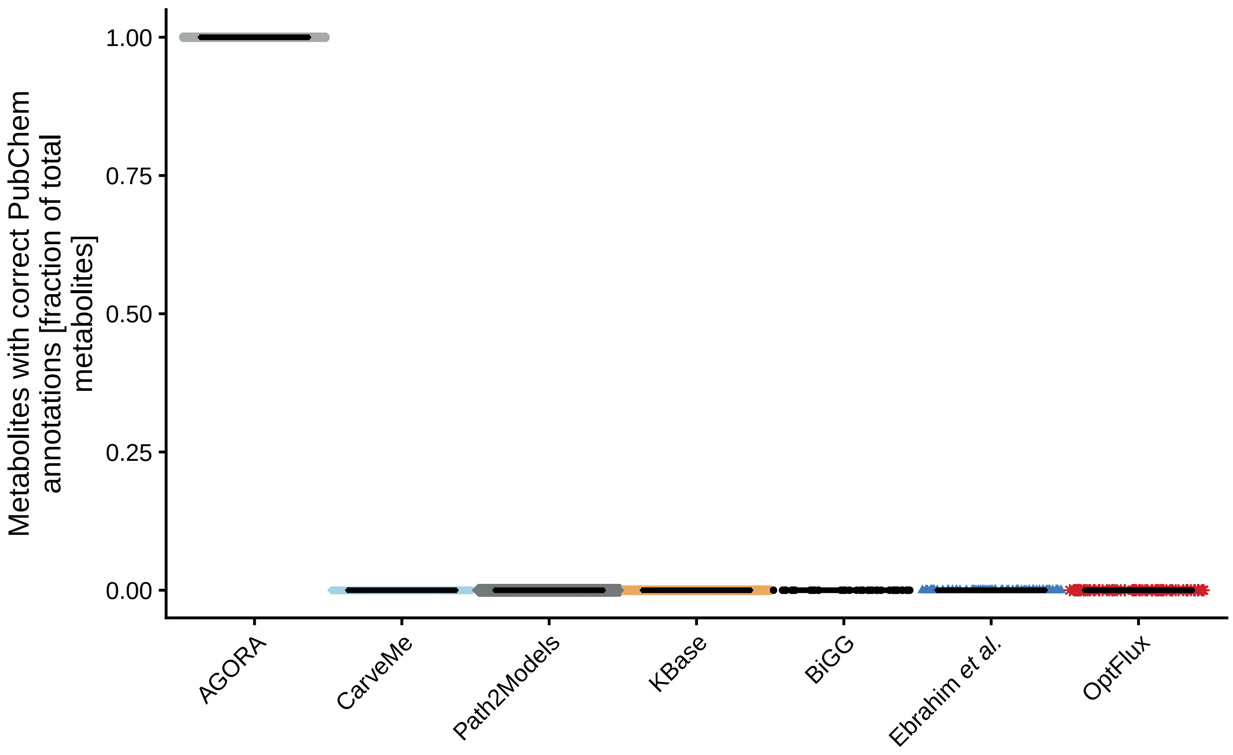

Figure 50: Correct Metabolite Pubchem.compound Annotation

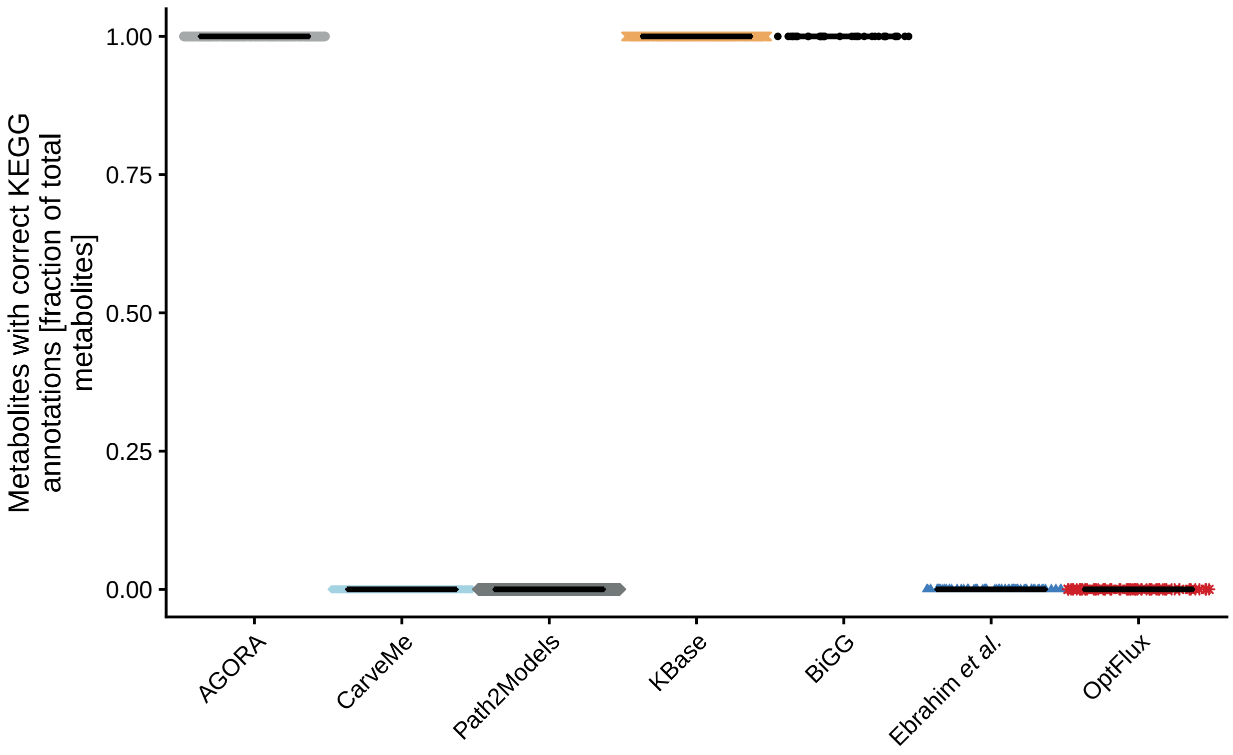

Figure 51: Correct Metabolite KEGG.compound Annotation

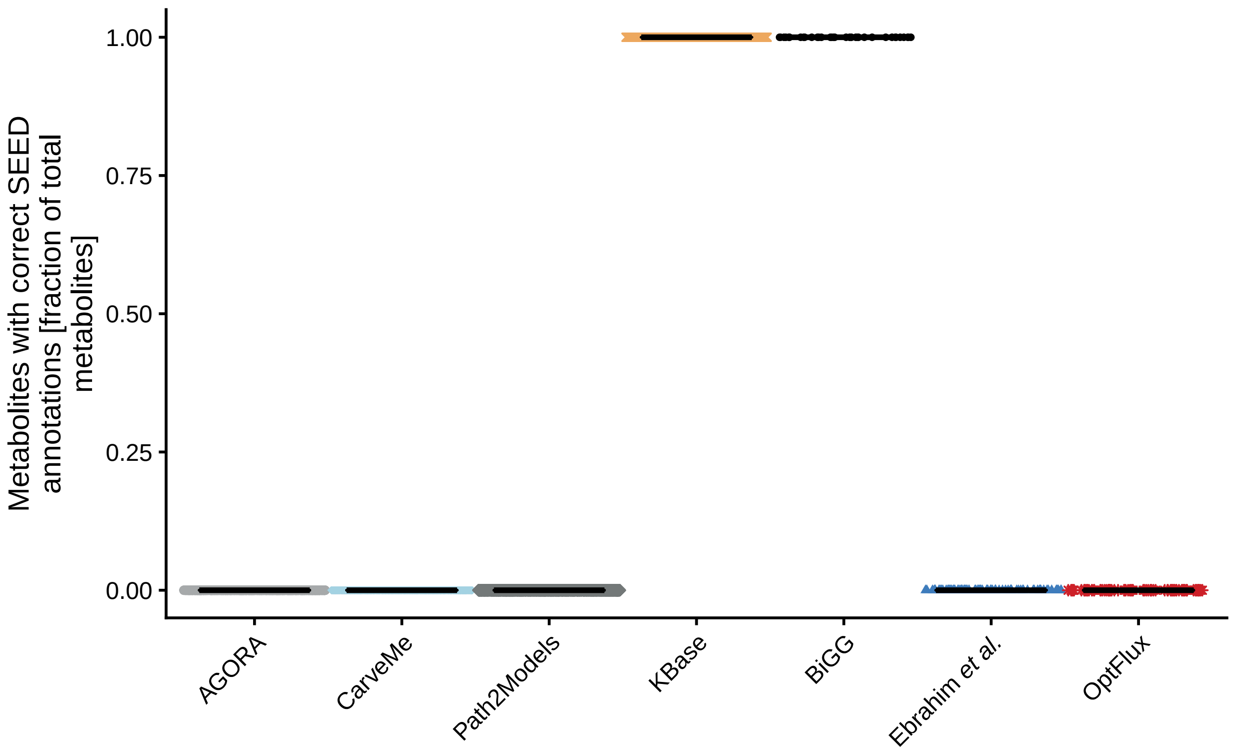

Figure 52: Correct Metabolite SEED.compound Annotation

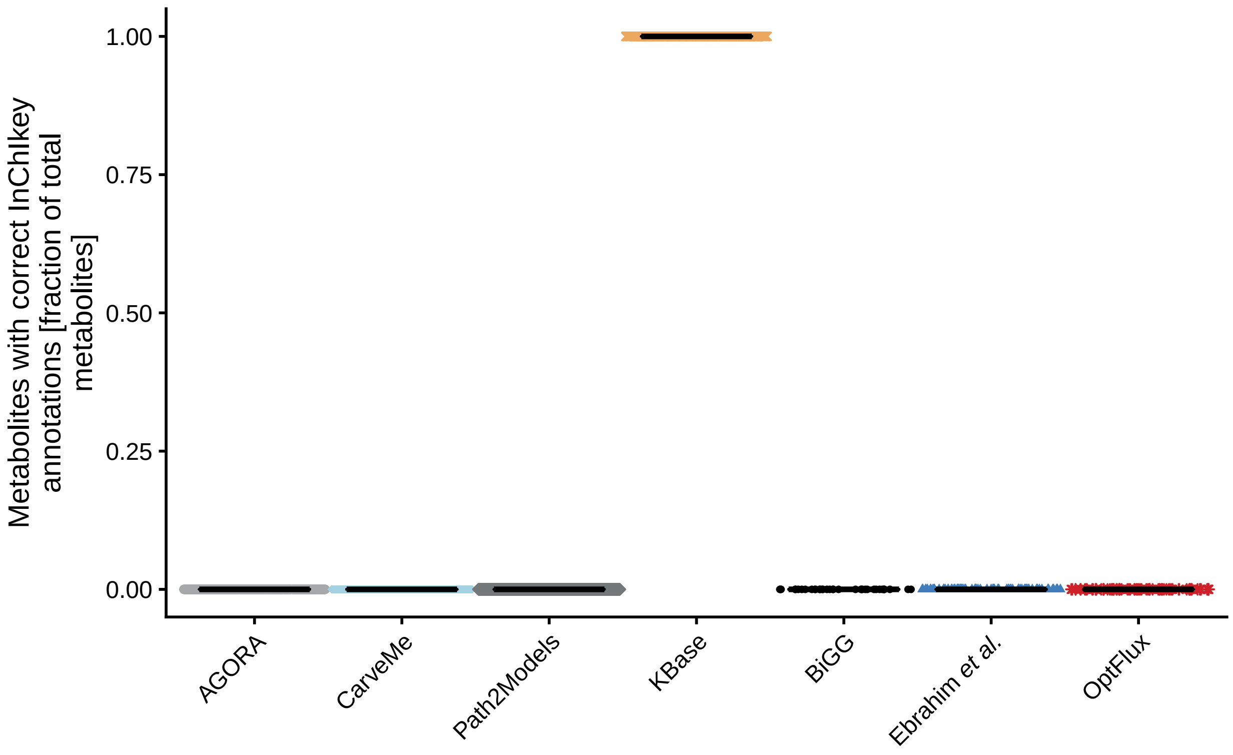

Figure 53: Correct Metabolite InChIKey Annotation

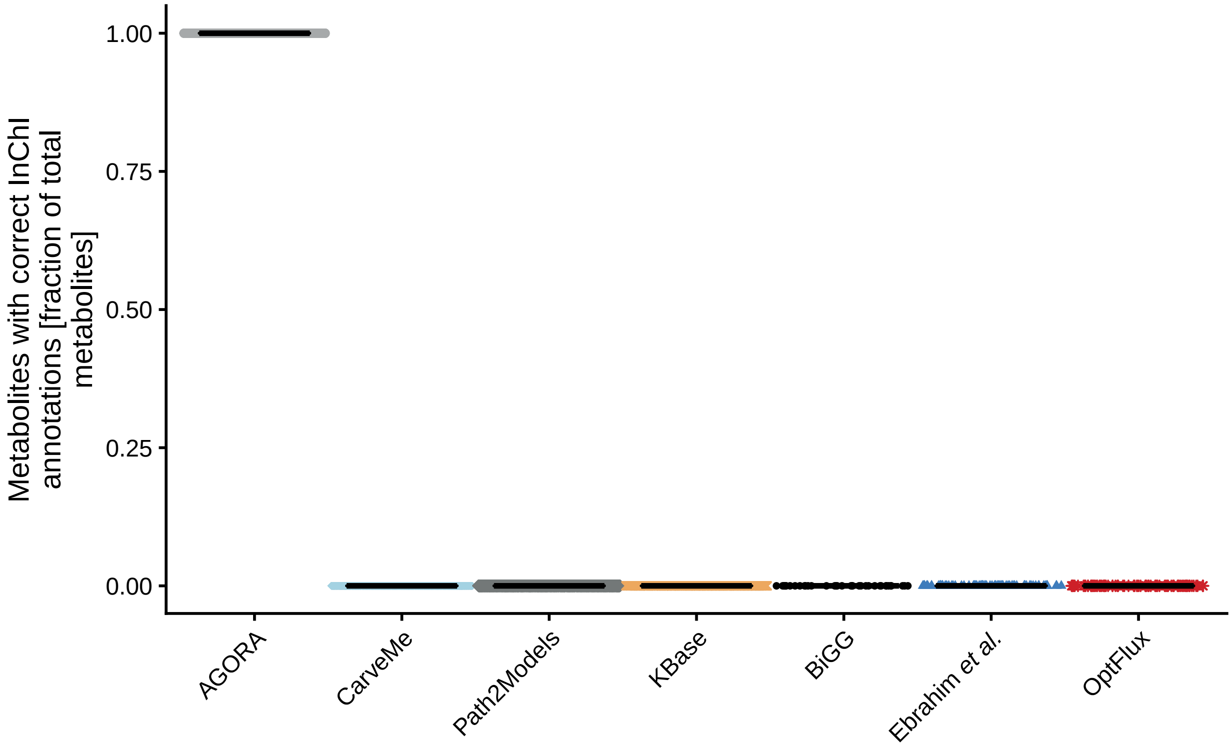

Figure 54: Correct Metabolite InChI Annotation

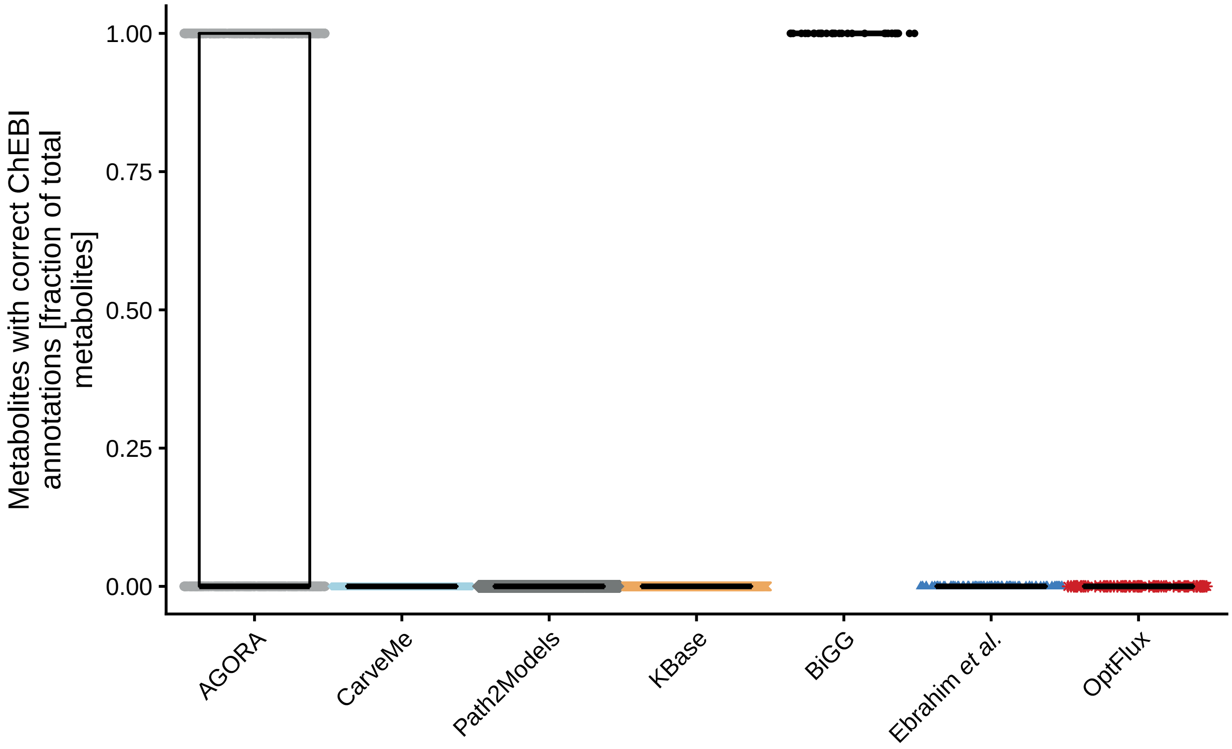

Figure 55: Correct Metabolite ChEBI Annotation

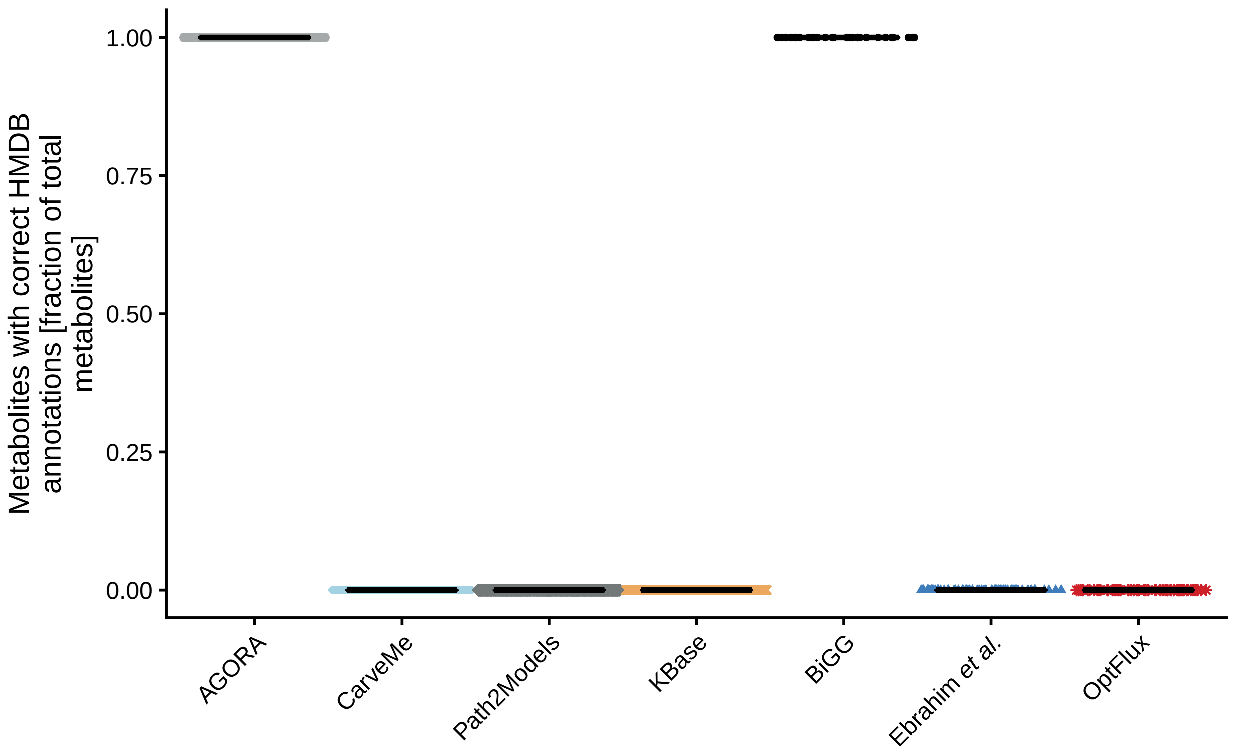

Figure 56: Correct Metabolite HMDB Annotation

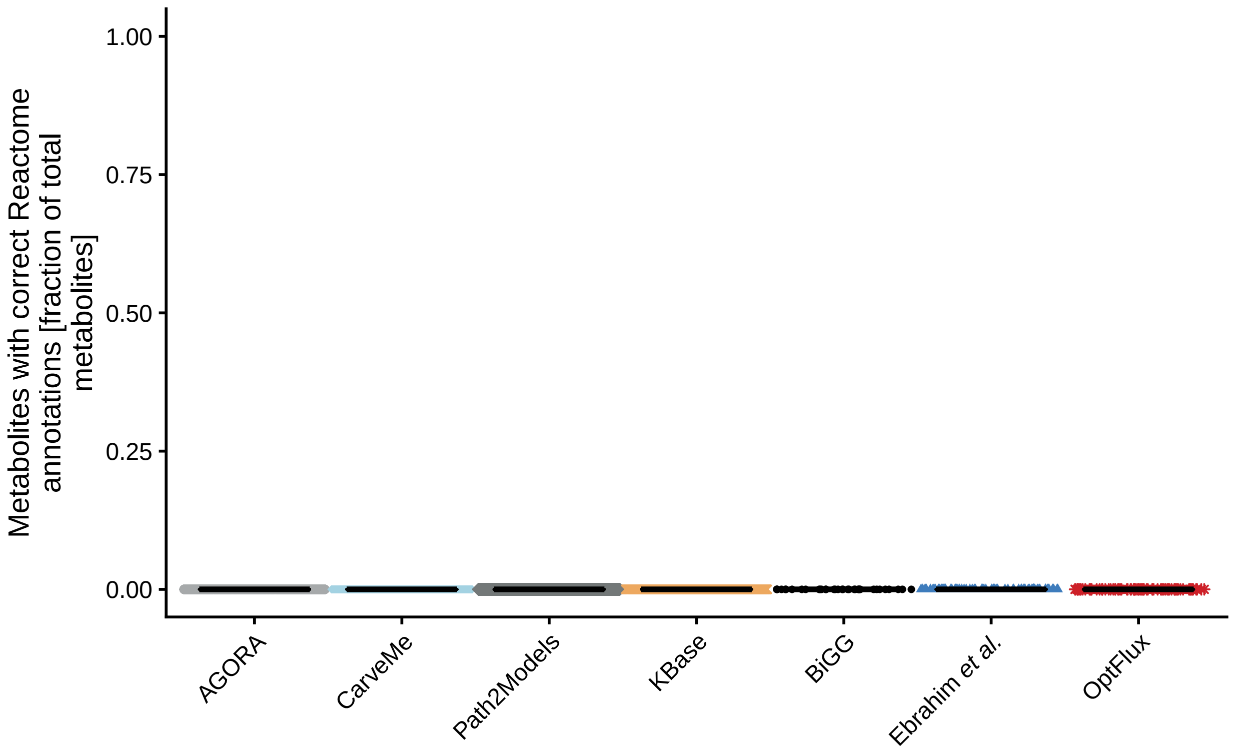

Figure 57: Correct Metabolite Reactome Annotation

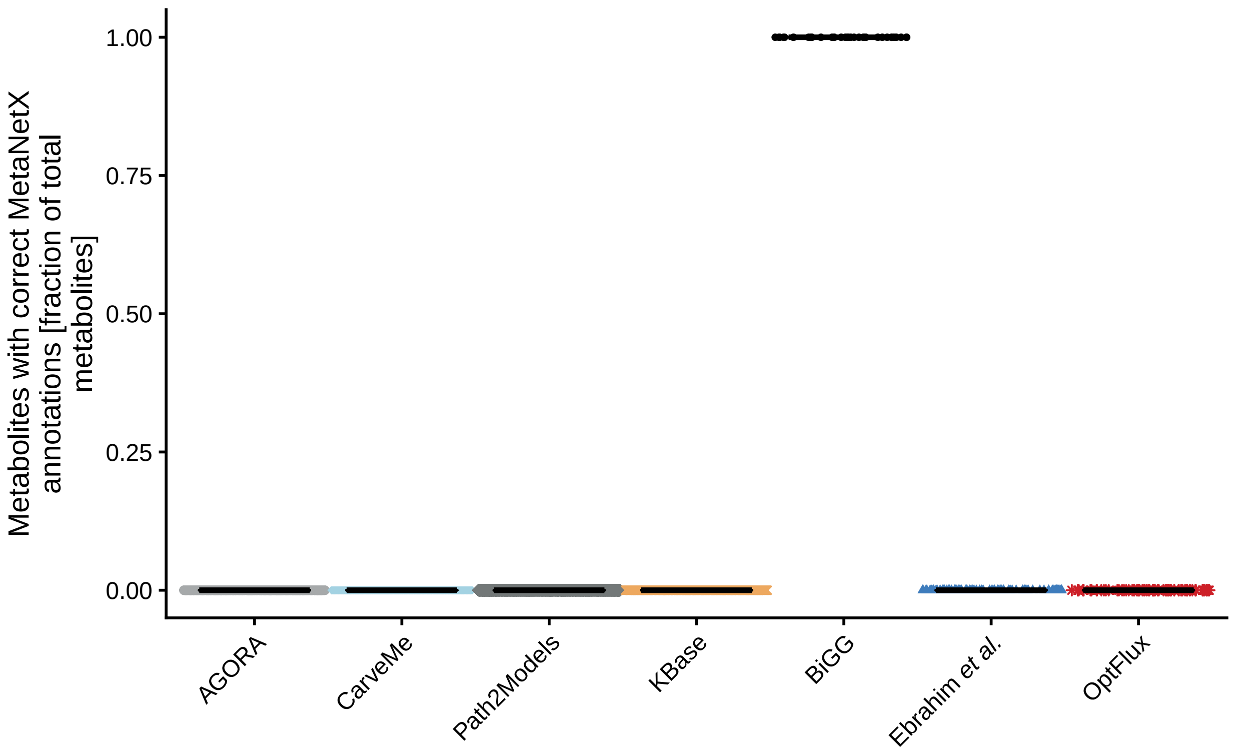

Figure 58: Correct Metabolite MetaNetX.chemical Annotation

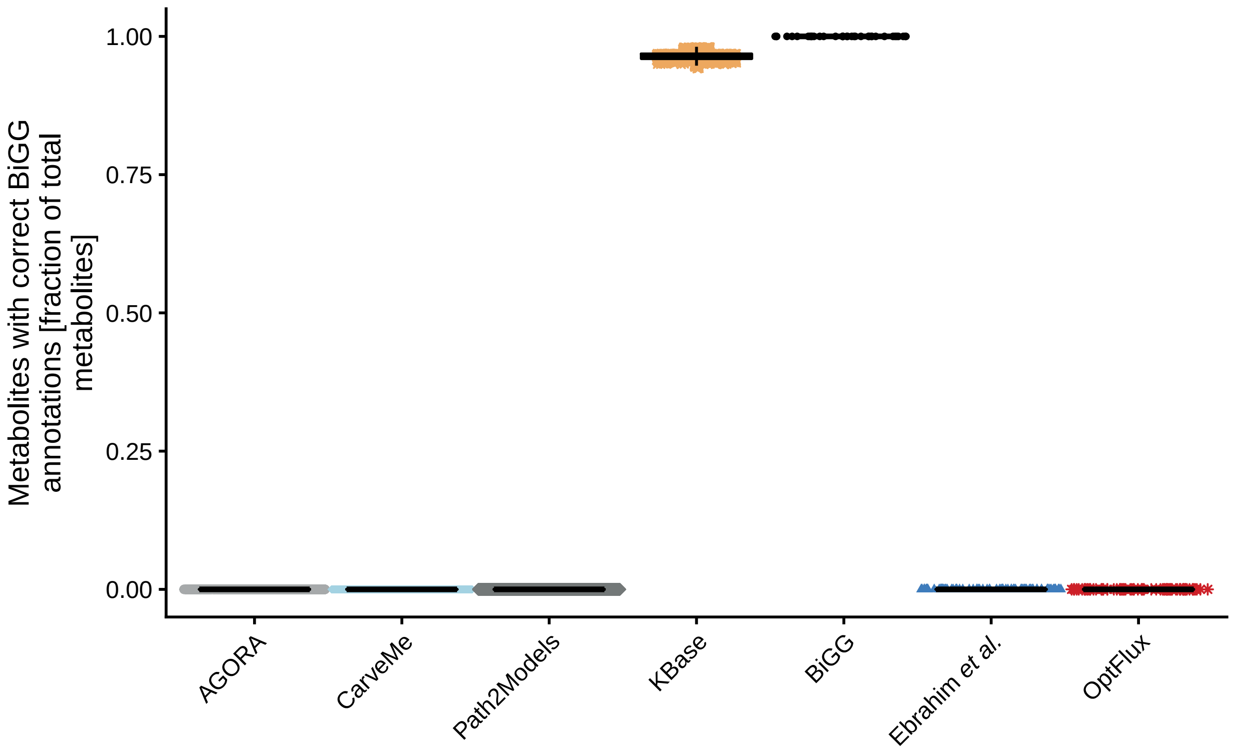

Figure 59: Correct Metabolite BiGG.metabolite Annotation

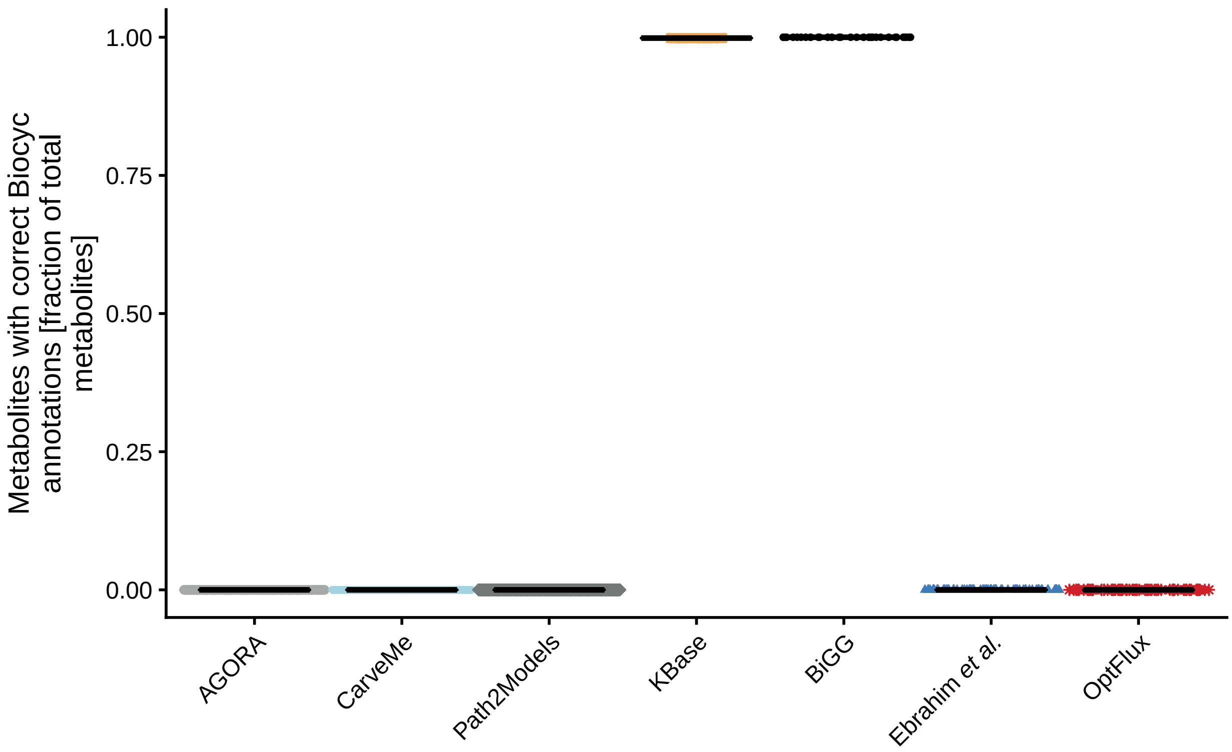

Figure 60: Correct Metabolite BioCyc Annotation

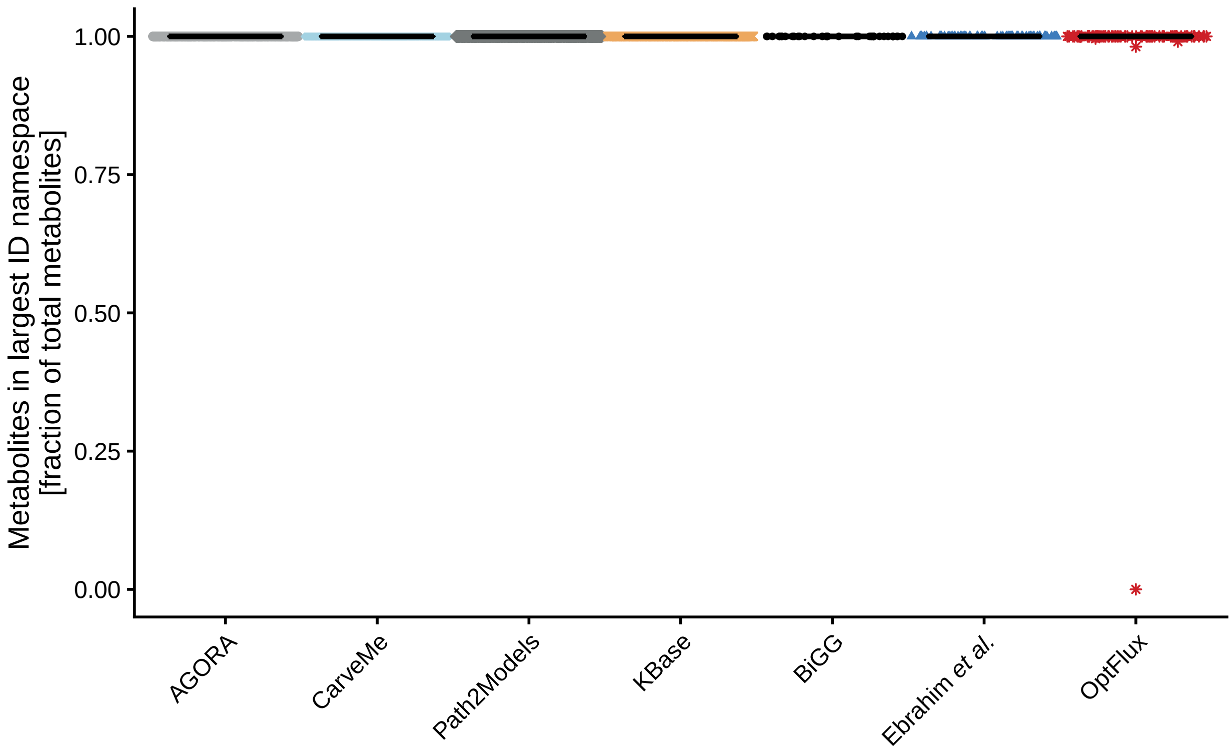

Figure 61: Uniform Metabolite Identifier Namespace

7.3.3.3 Annotation - Reactions

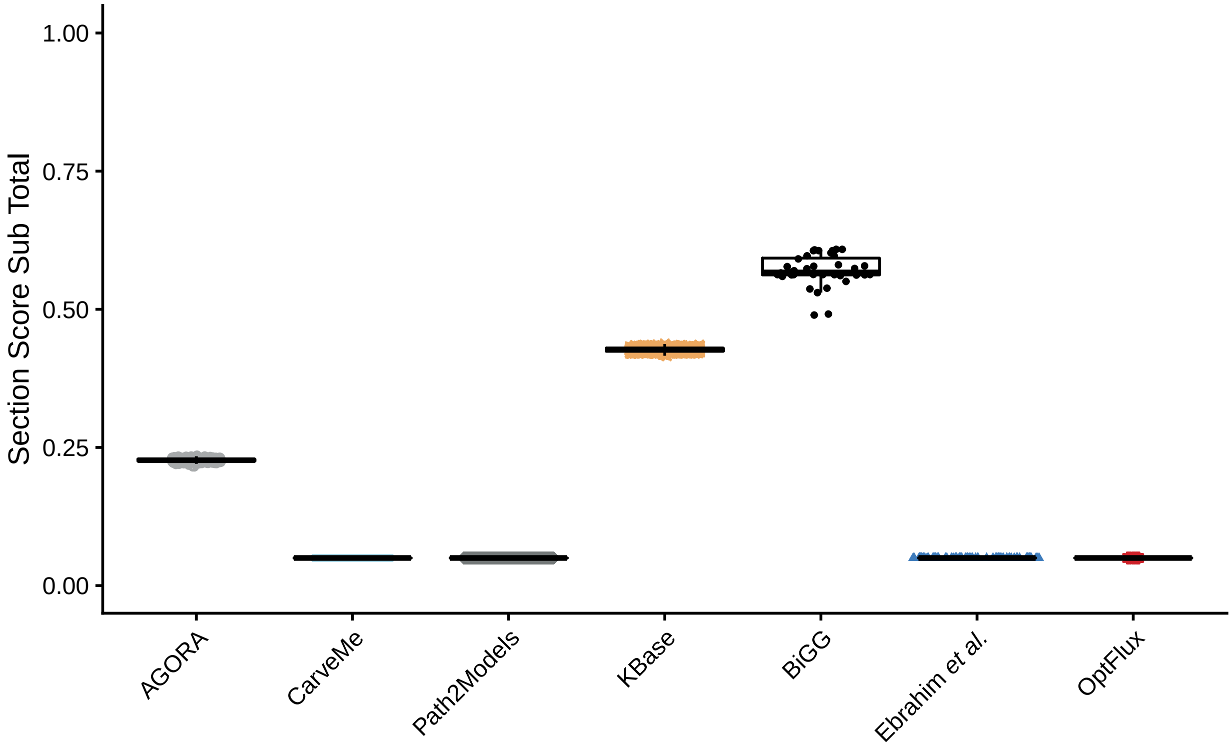

Figure 62: Annotation - Reactions. Depicted are the sums of all test scores in this section, applying the weights of the individual test cases as detailed in the snapshot report.

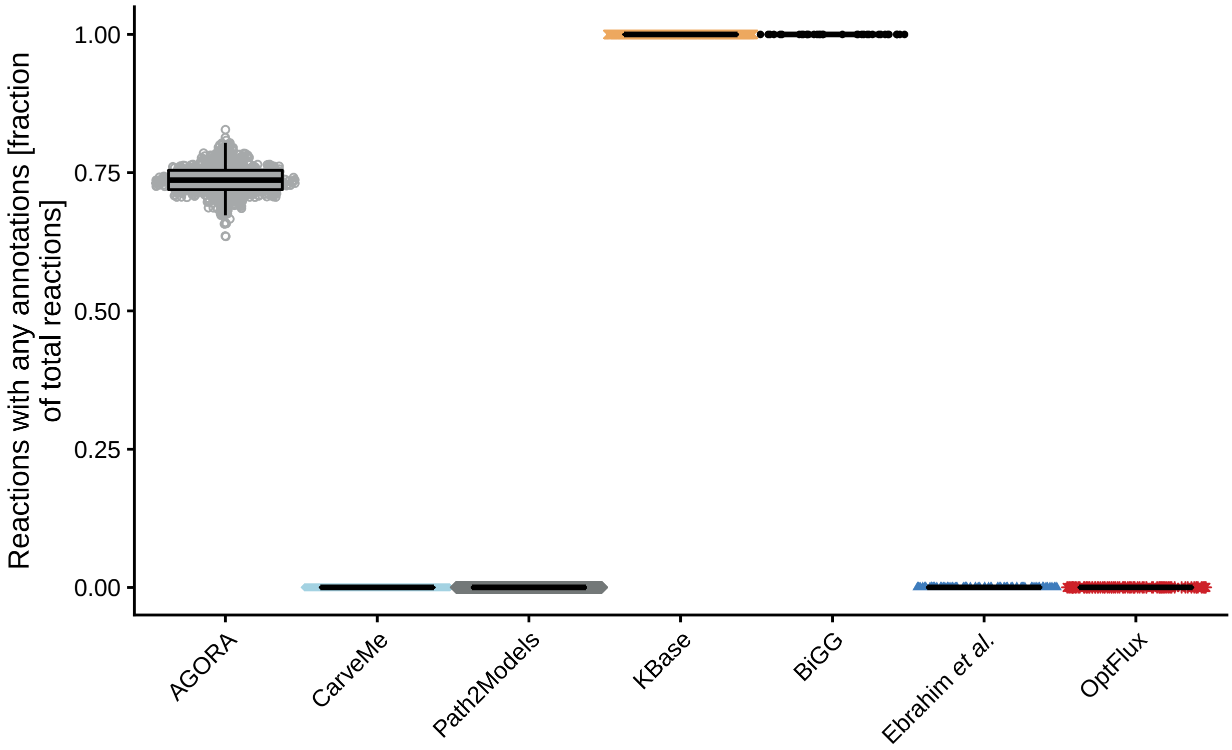

Figure 63: Presence of Reaction Annotation

7.3.3.3.1 Reaction Annotations Per Database

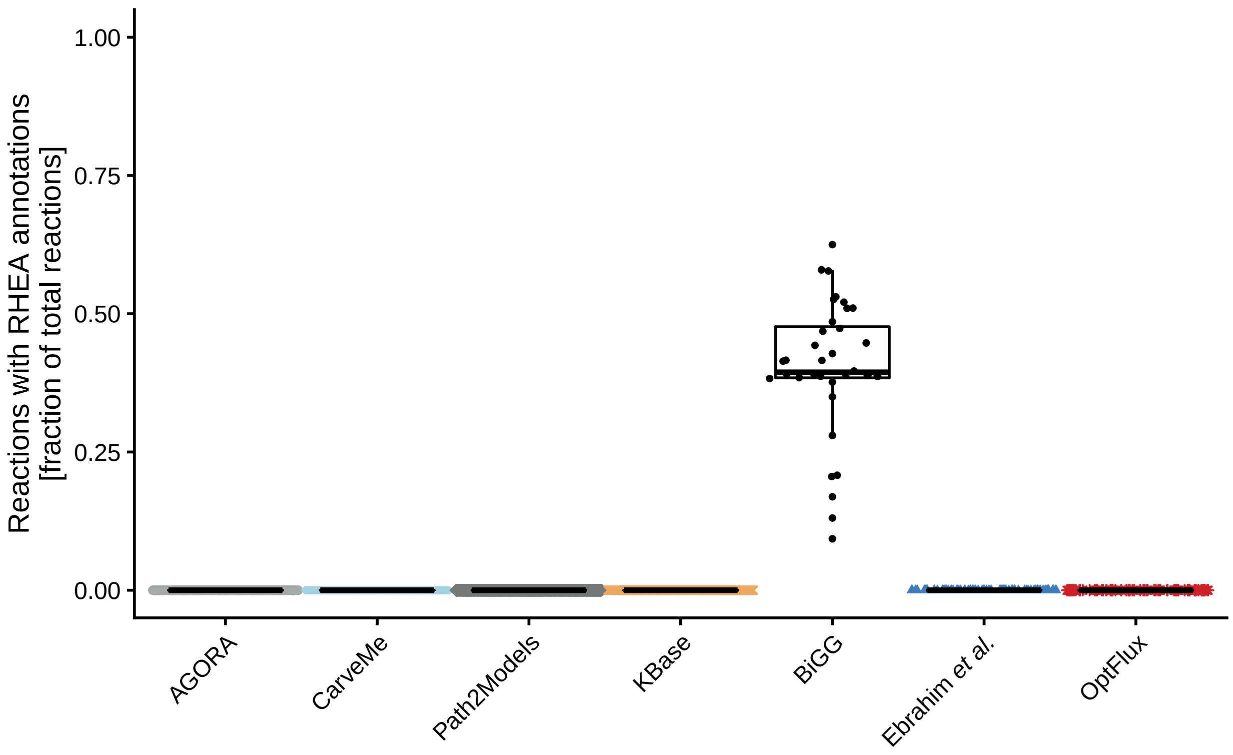

Figure 64: Reaction Rhea Annotation

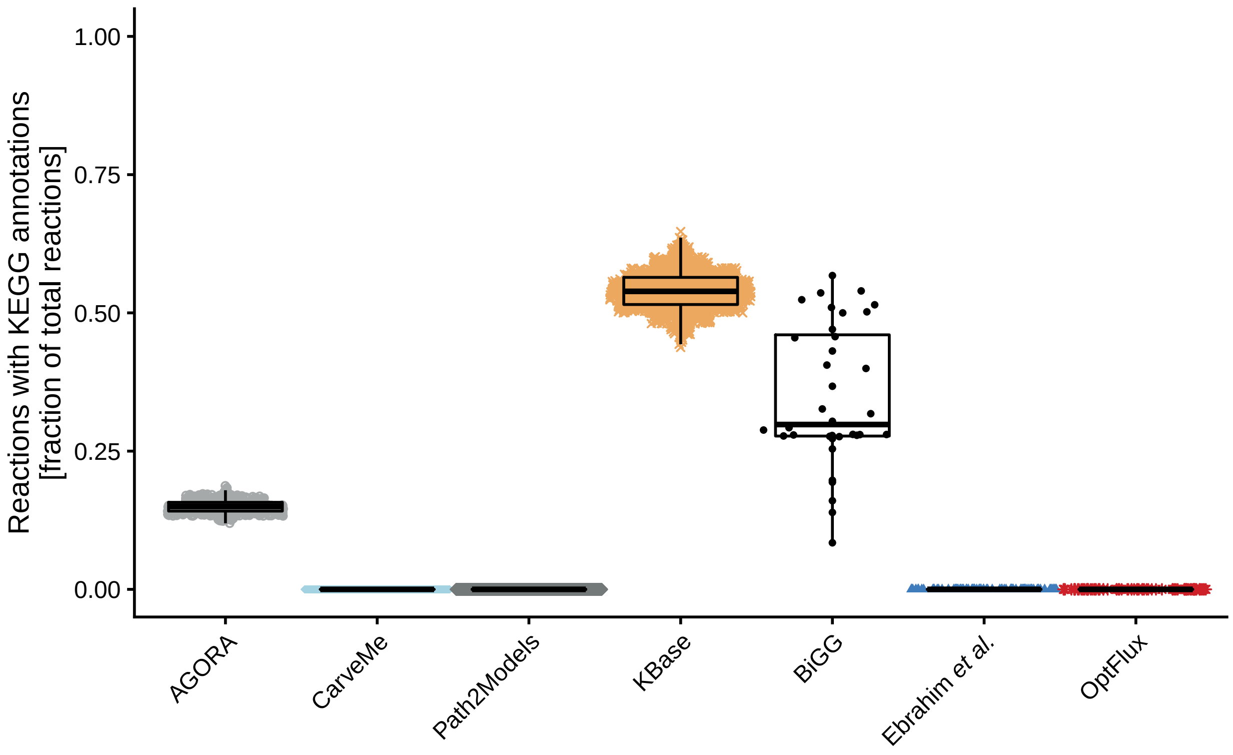

Figure 65: Reaction KEGG.reaction Annotation

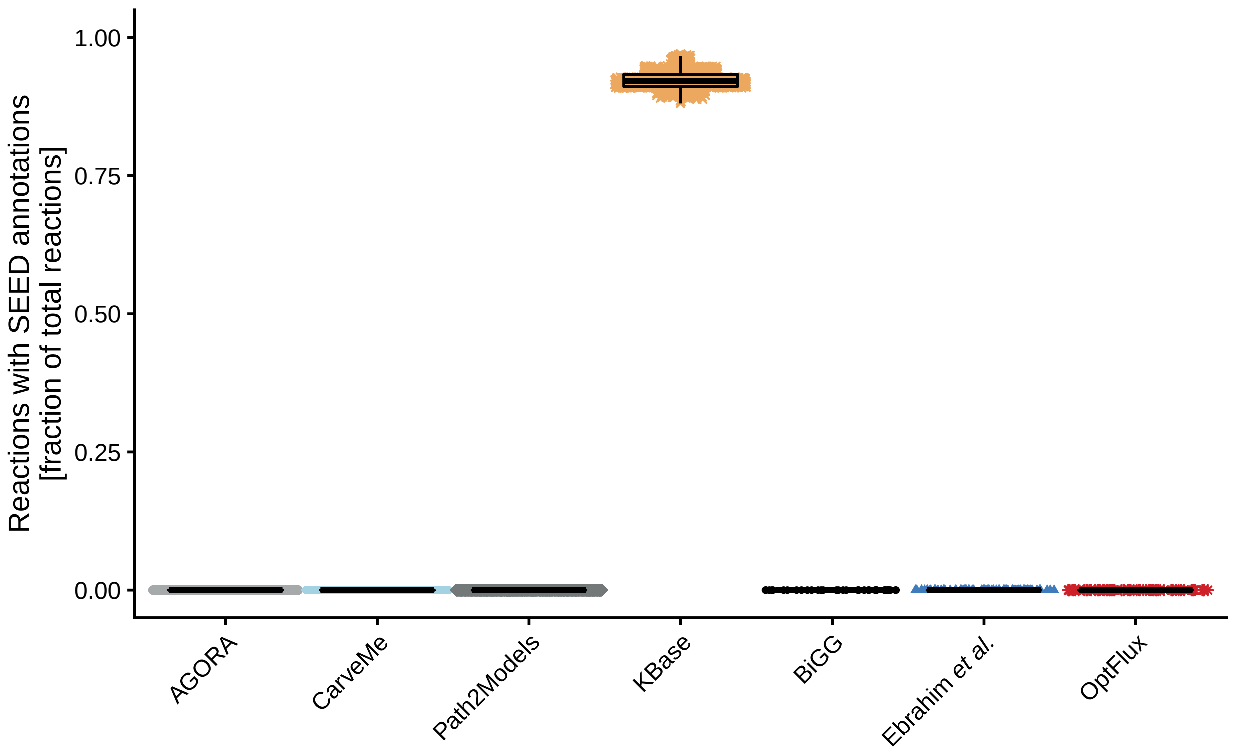

Figure 66: Reaction SEED.reaction Annotation

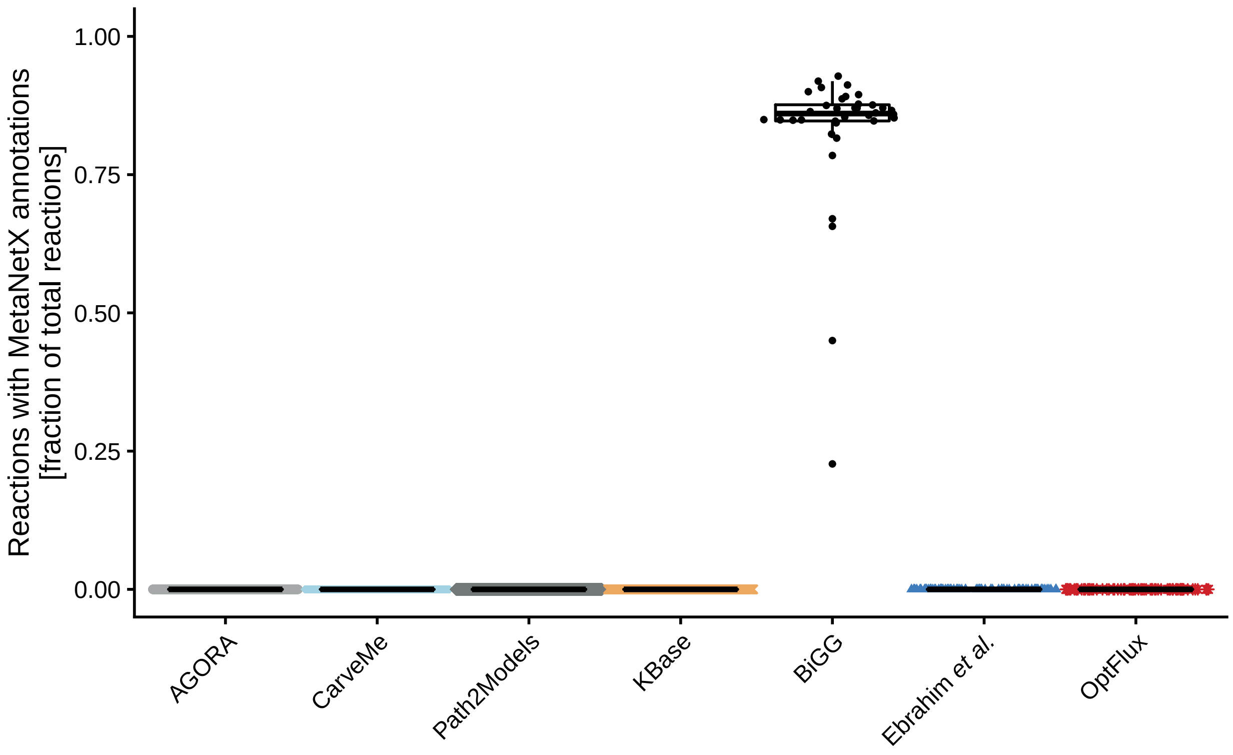

Figure 67: Reaction MetaNetX.reaction Annotation

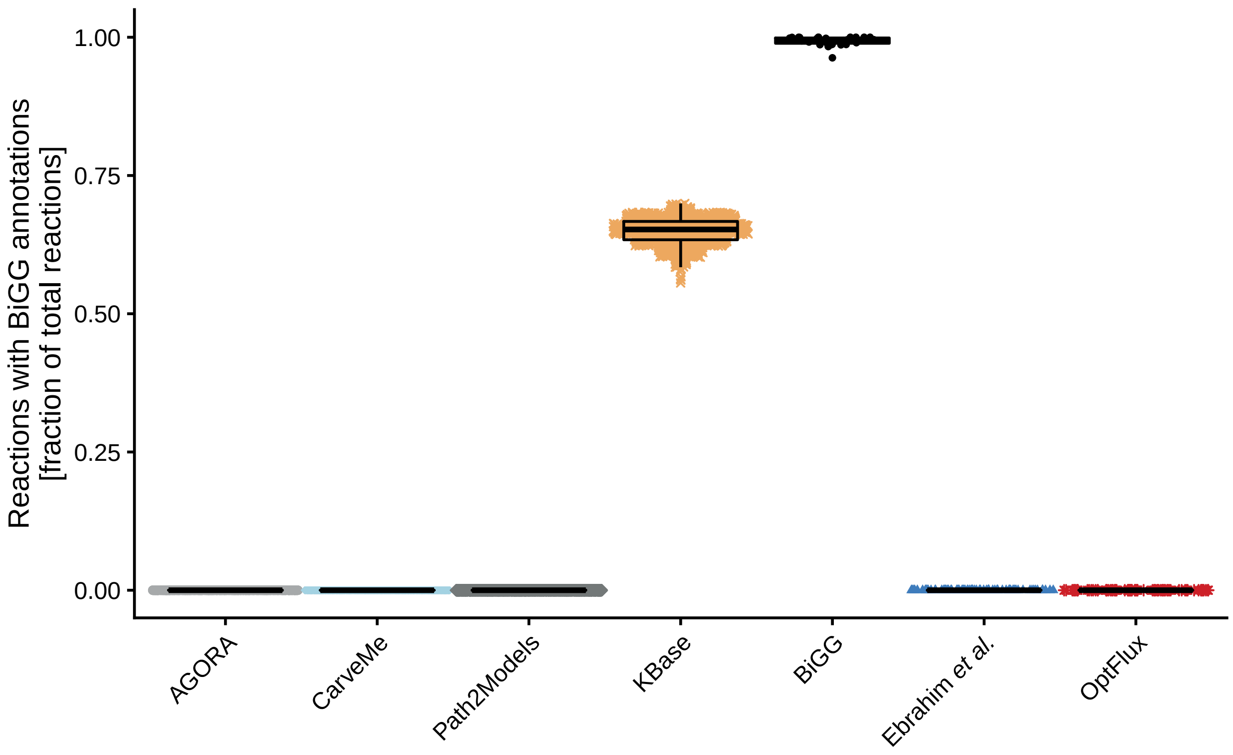

Figure 68: Reaction BiGG.reaction Annotation

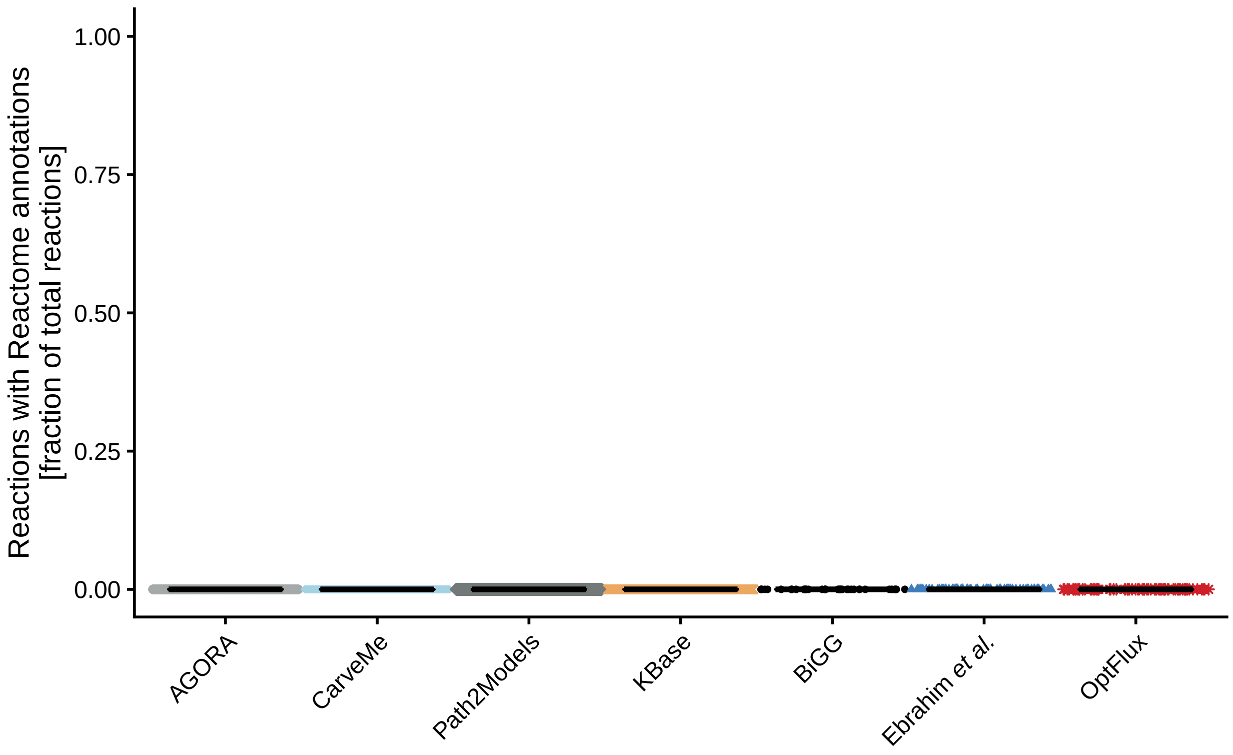

Figure 69: Reaction Reactome Annotation

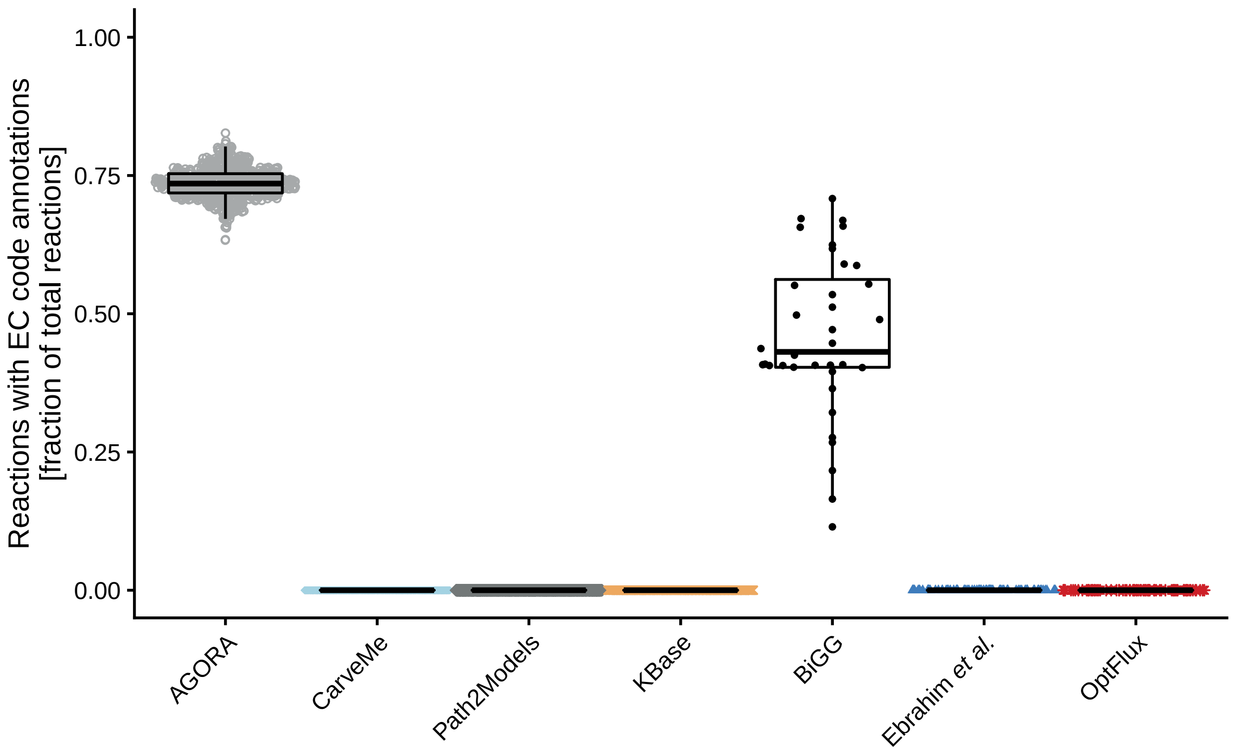

Figure 70: Reaction Enzyme Classification Annotation

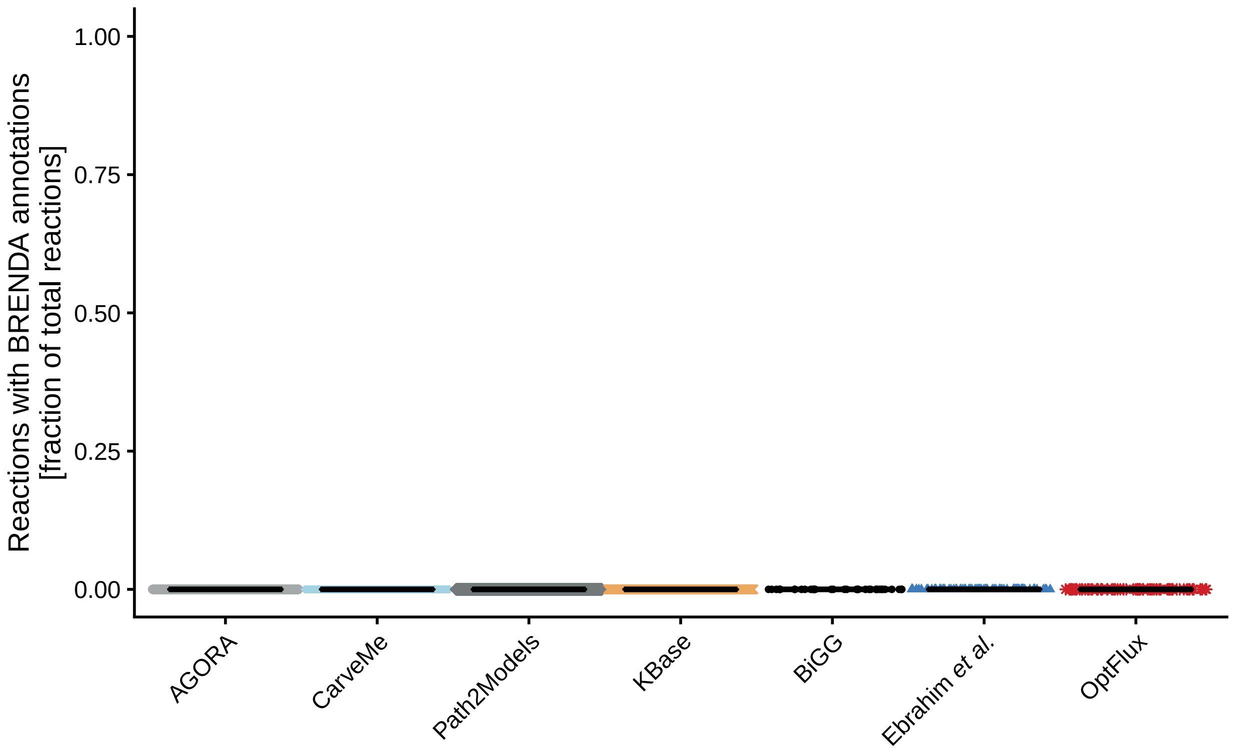

Figure 71: Reaction BRENDA Annotation

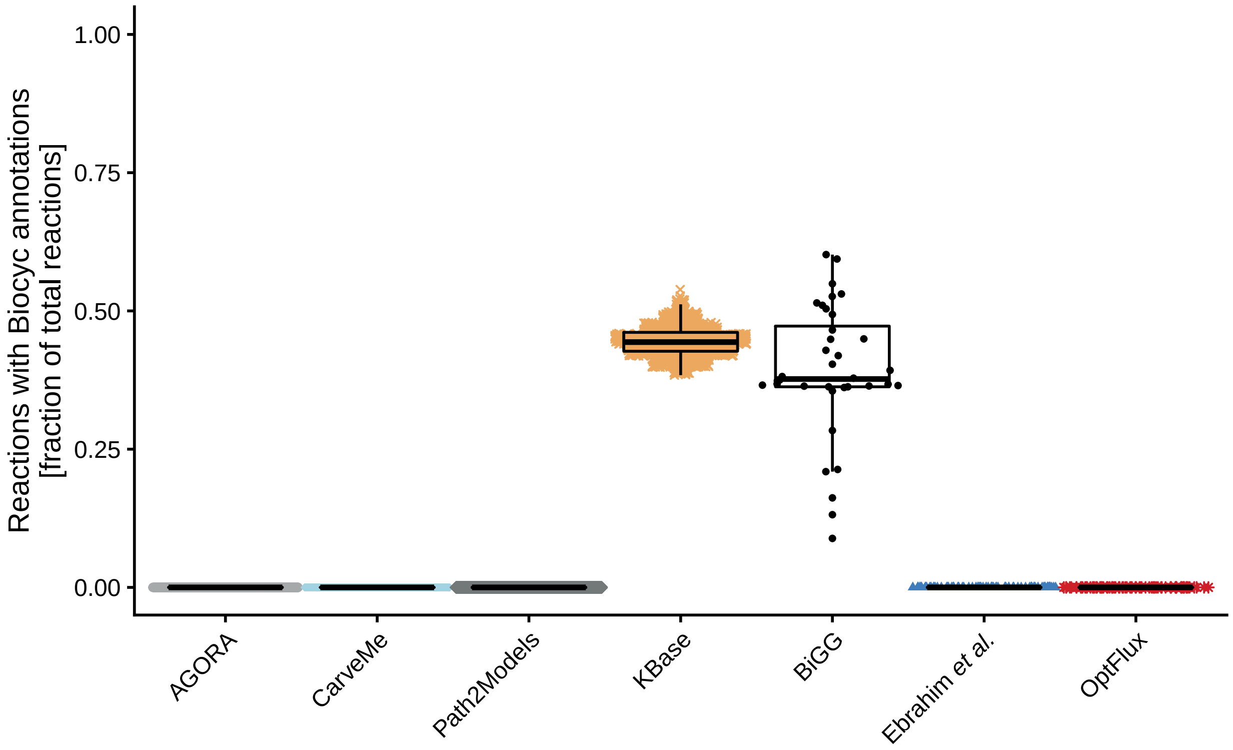

Figure 72: Reaction BioCyc Annotation

7.3.3.3.2 Reaction Annotation Conformity Per Database

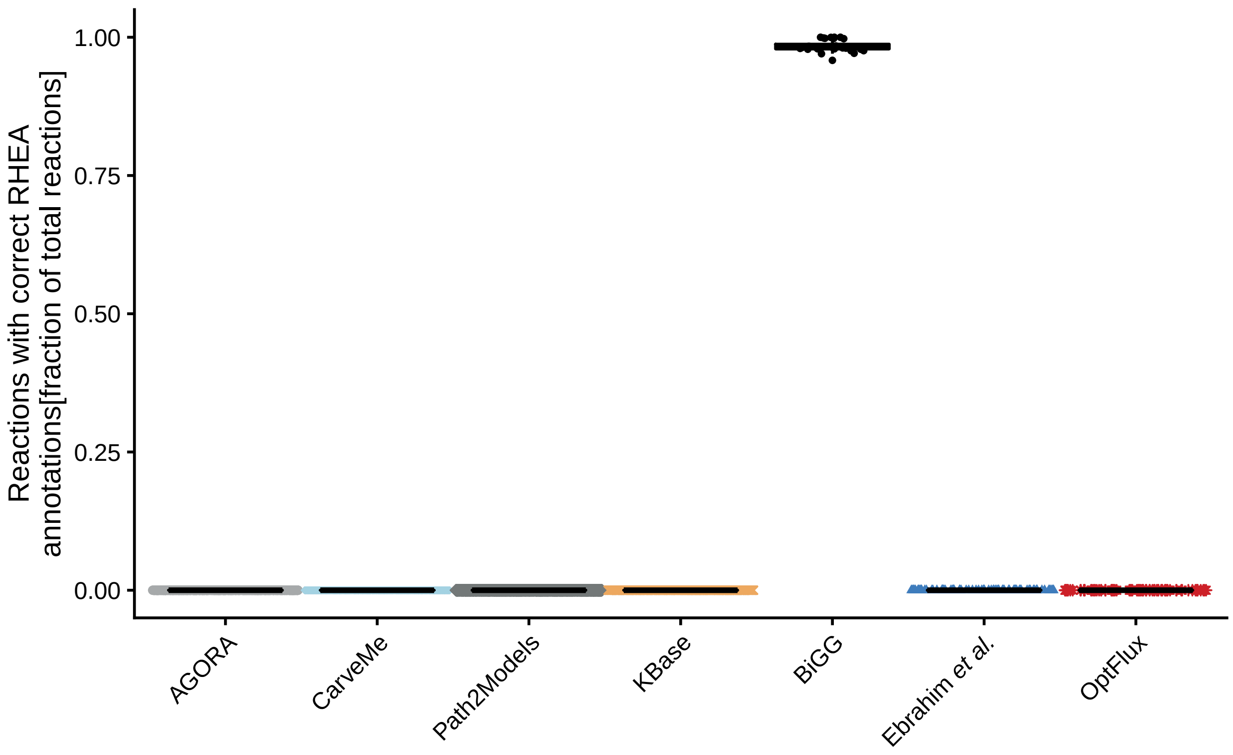

Figure 73: Correct Reaction Rhea Annotation

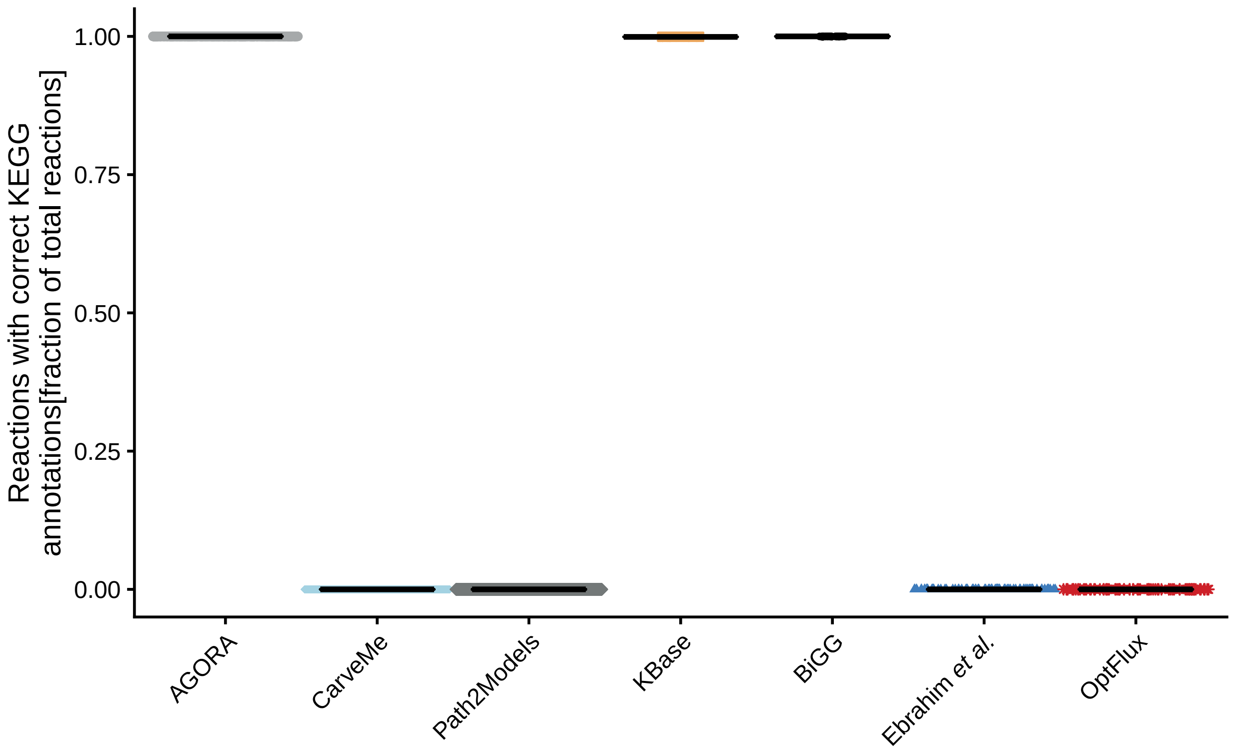

Figure 74: Correct Reaction KEGG.reaction Annotation

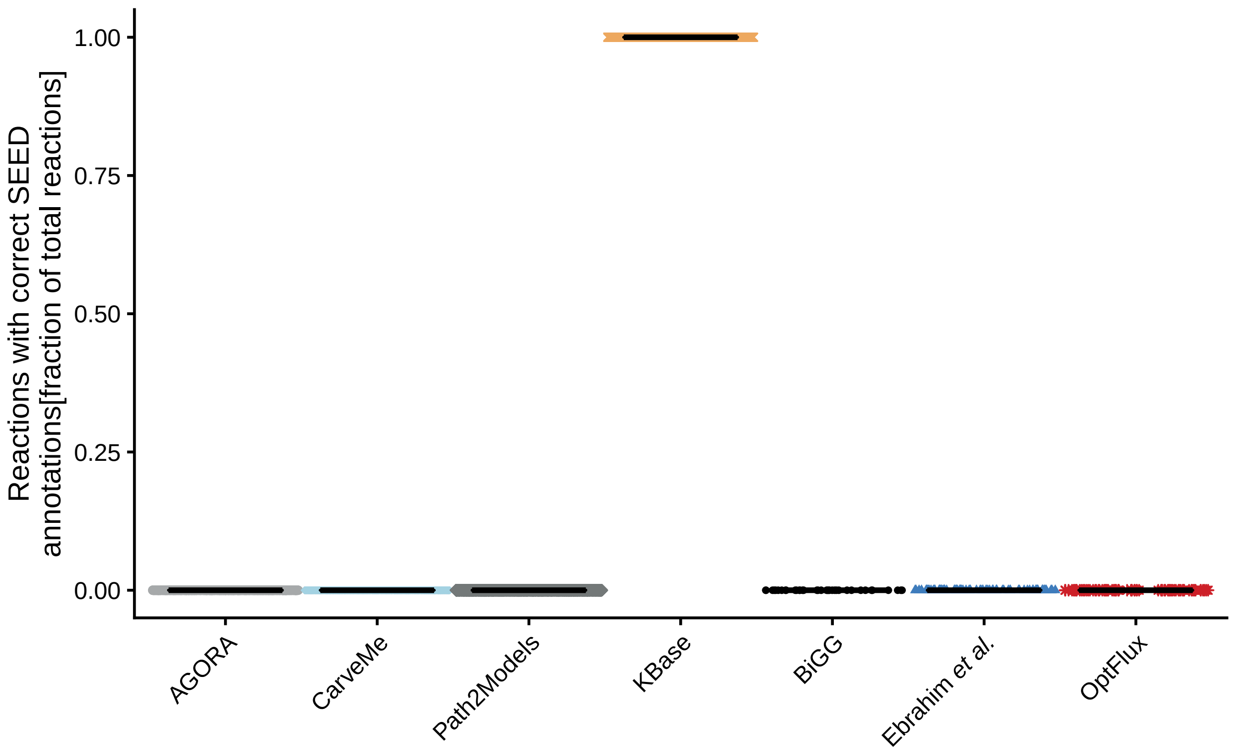

Figure 75: Correct Reaction SEED.reaction Annotation

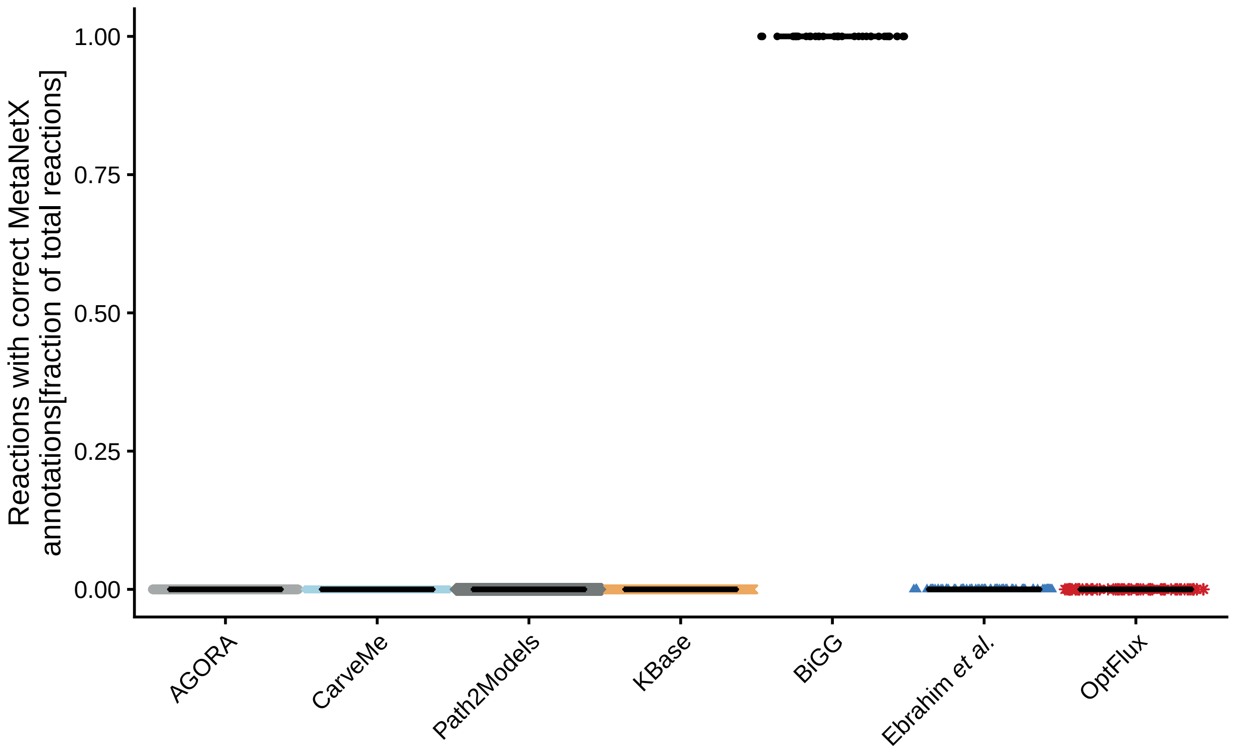

Figure 76: Correct Reaction MetaNetX.reaction Annotation

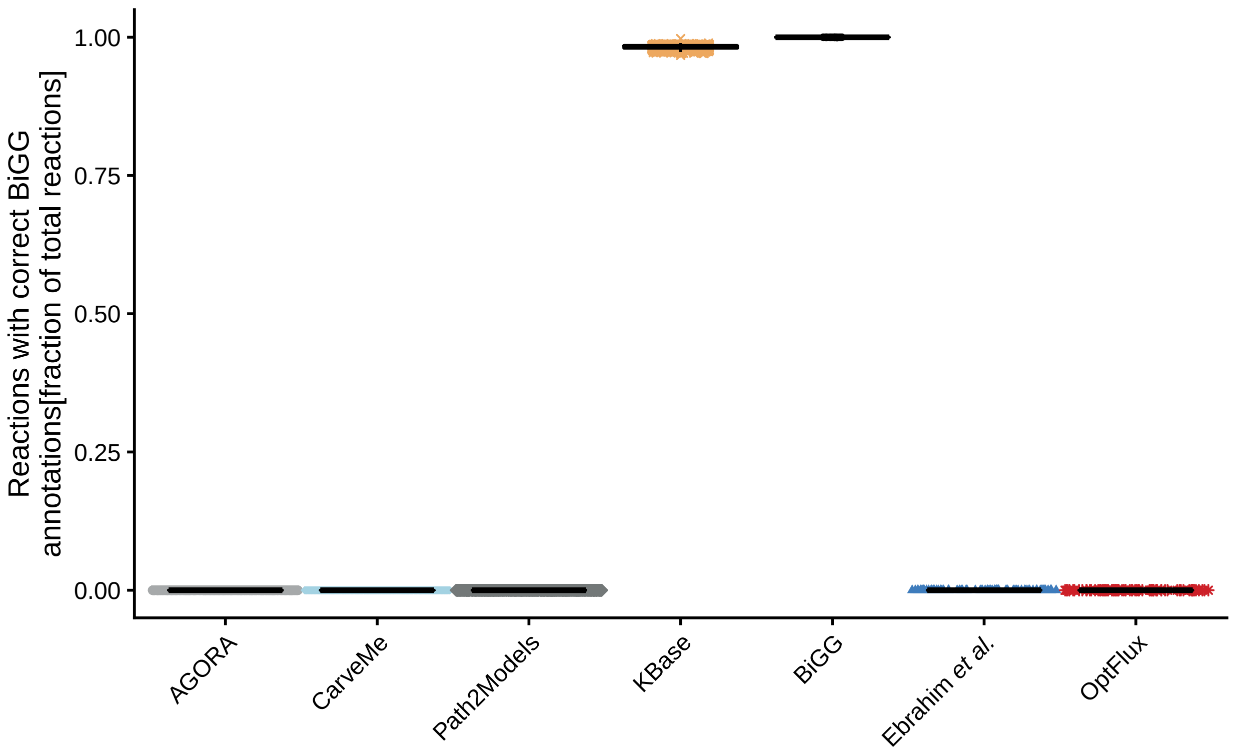

Figure 77: Correct Reaction BiGG.reaction Annotation

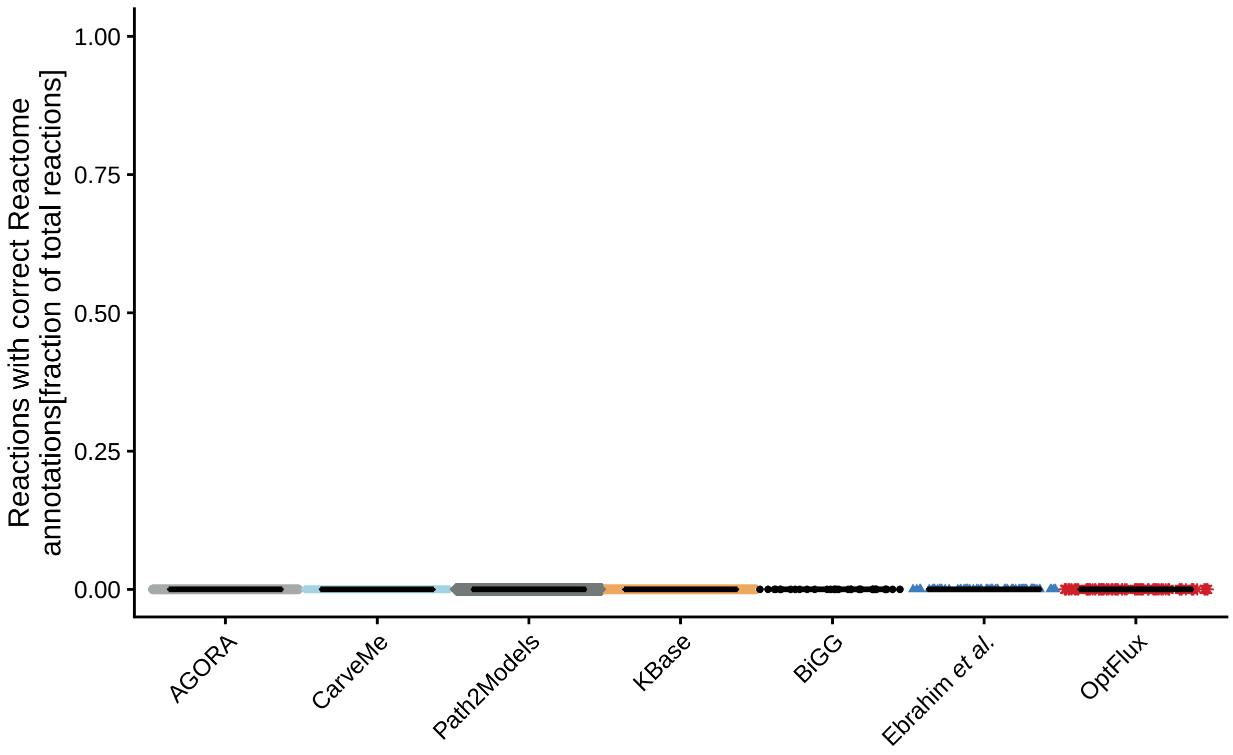

Figure 78: Correct Reaction Reactome Annotation

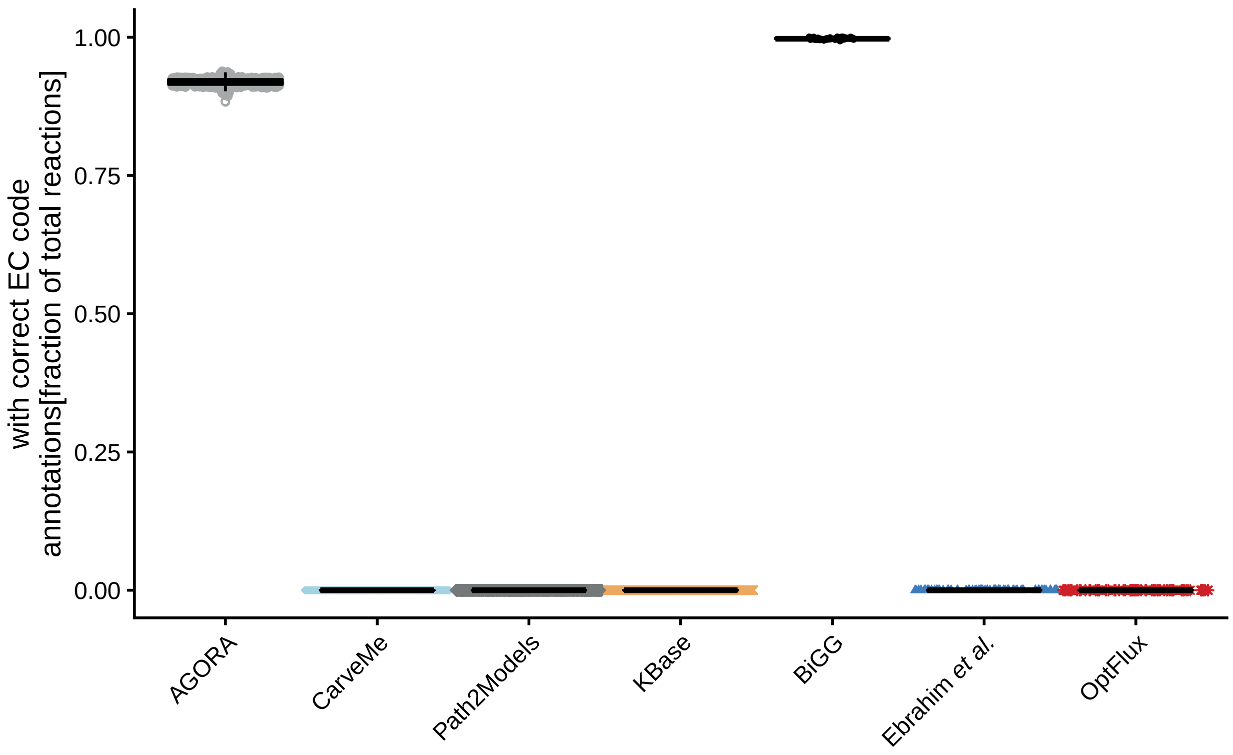

Figure 79: Correct Reaction Enzyme Classification Annotation

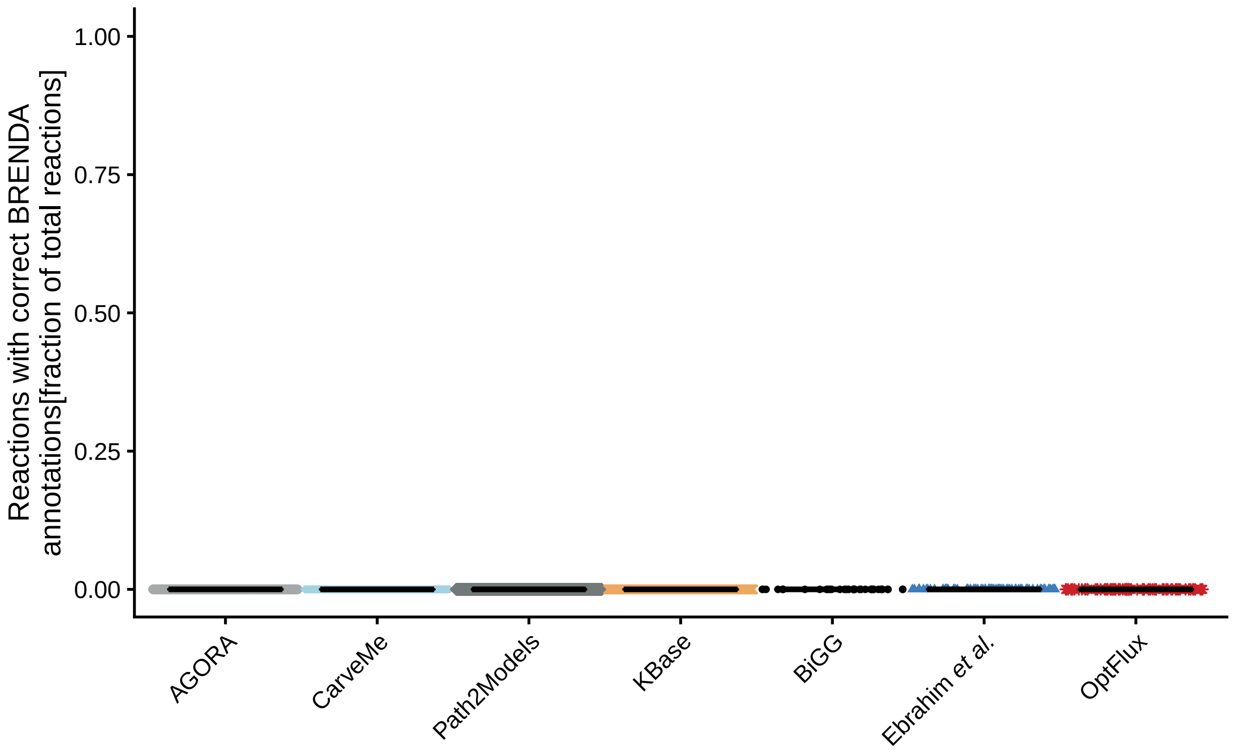

Figure 80: Correct Reaction BRENDA Annotation

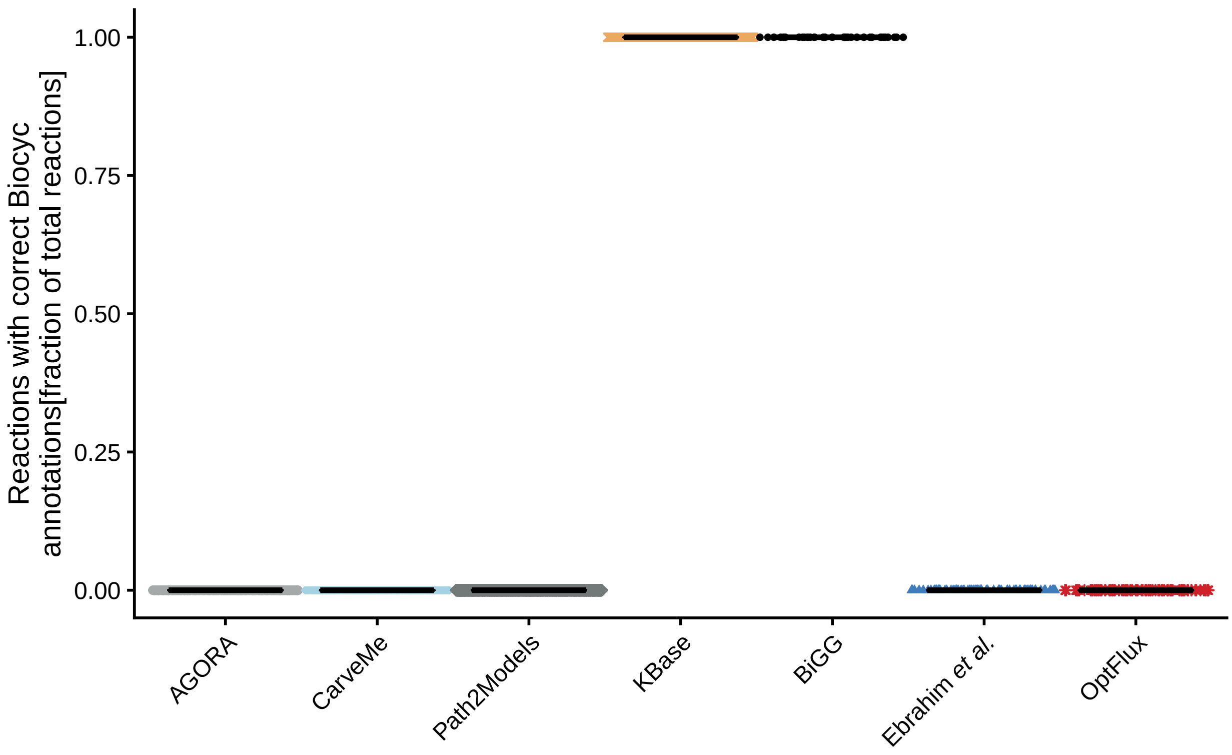

Figure 81: Correct Reaction BioCyc Annotation

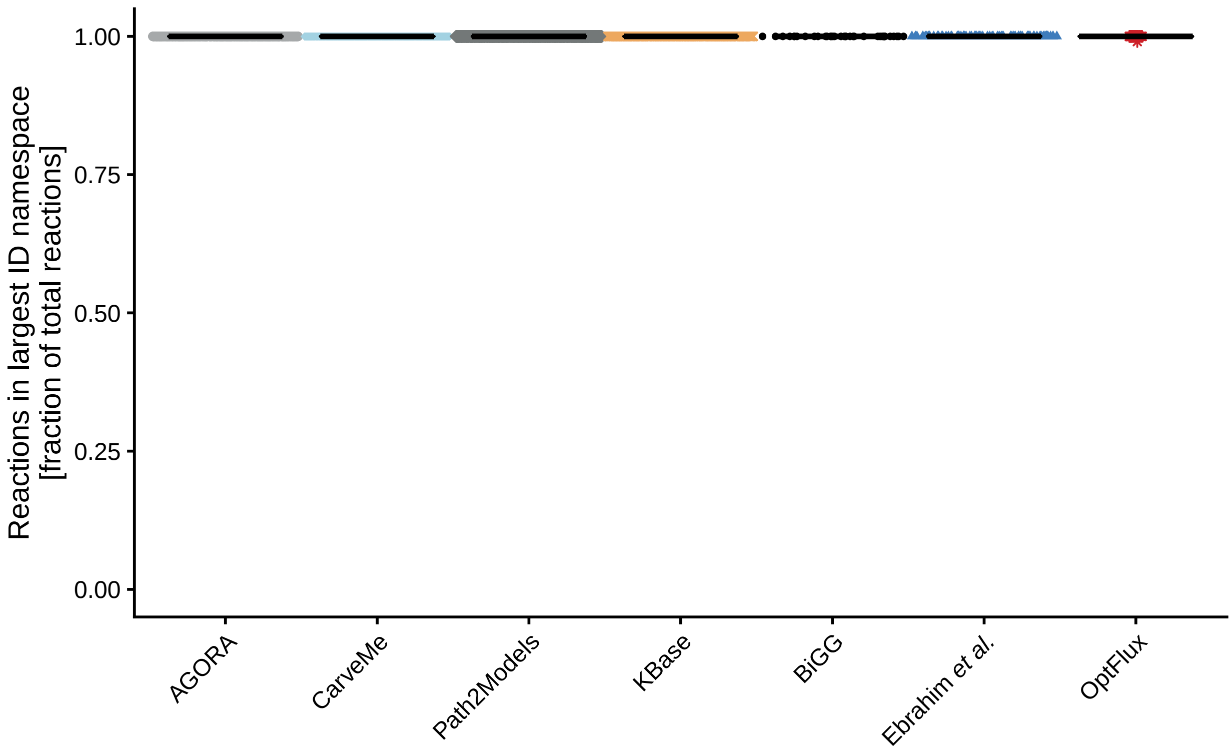

Figure 82: Uniform Reaction Identifier Namespace

7.3.3.4 Annotation - Genes

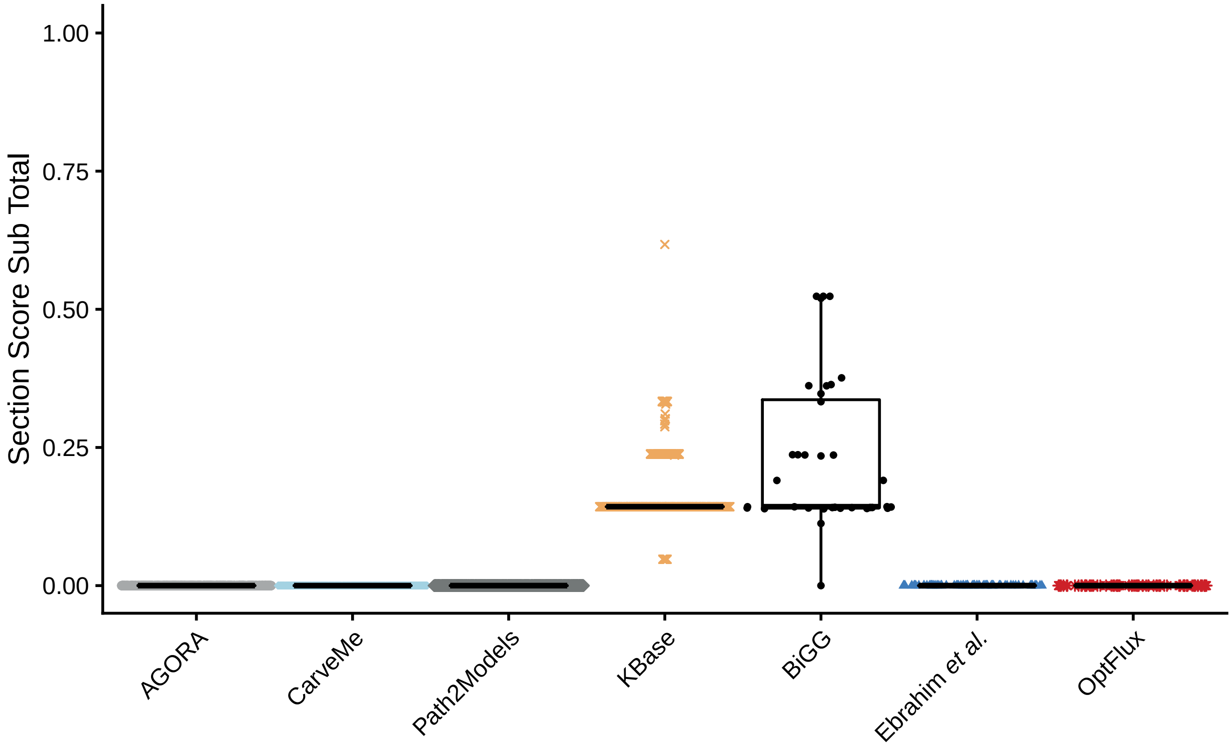

Figure 83: Annotation - Genes. Depicted are the sums of all test scores in this section, applying the weights of the individual test cases as detailed in the snapshot report.

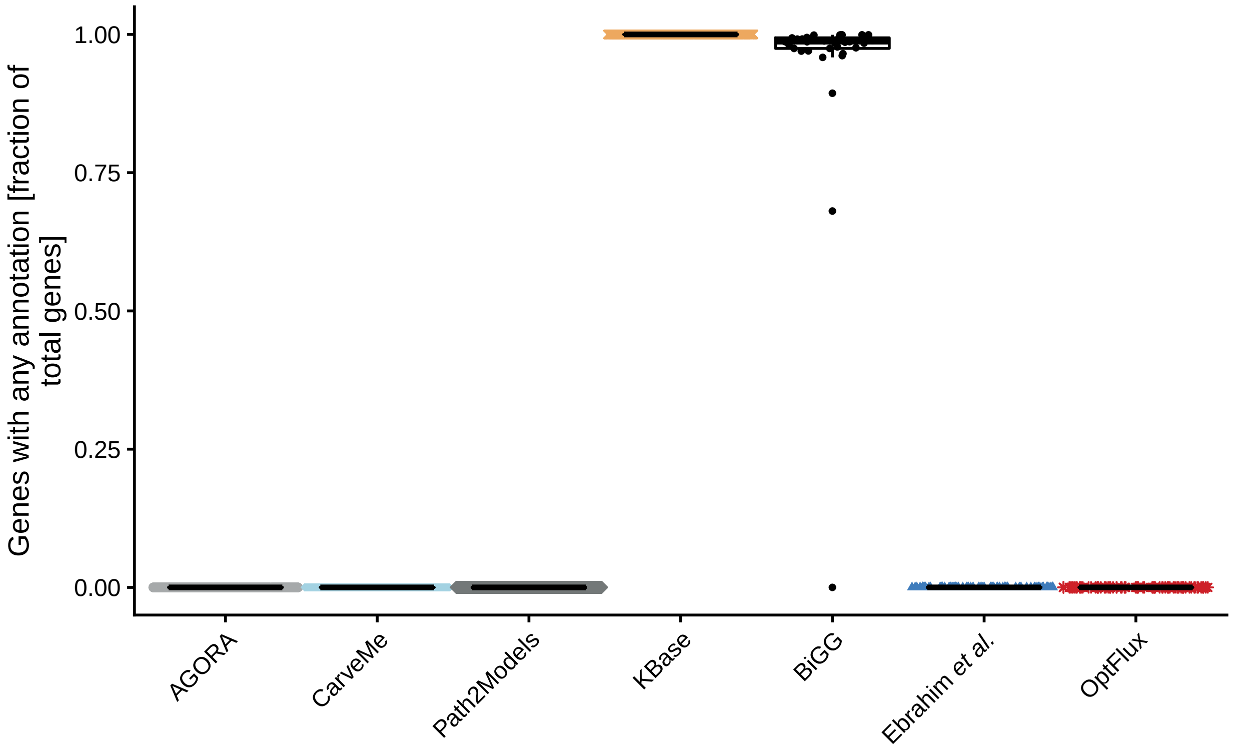

Figure 84: Presence of Gene Annotation

7.3.3.4.1 Gene Annotations Per Database

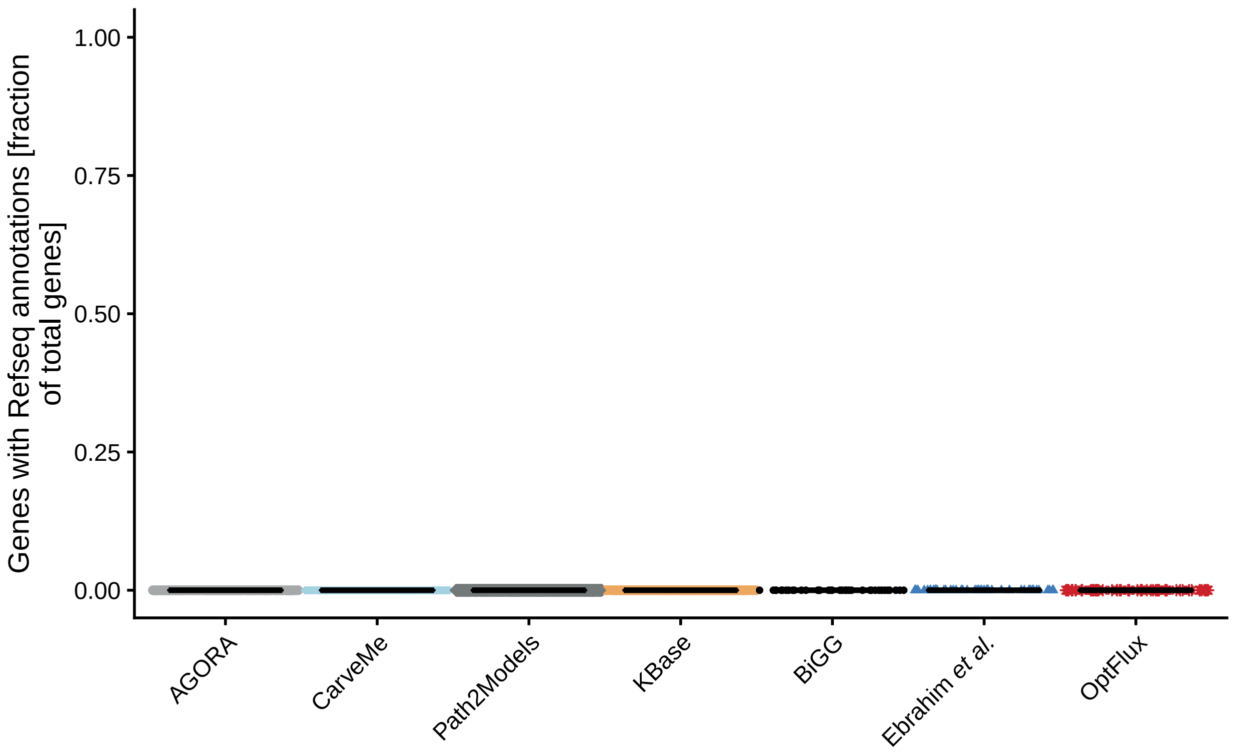

Figure 85: Gene RefSeq Annotation

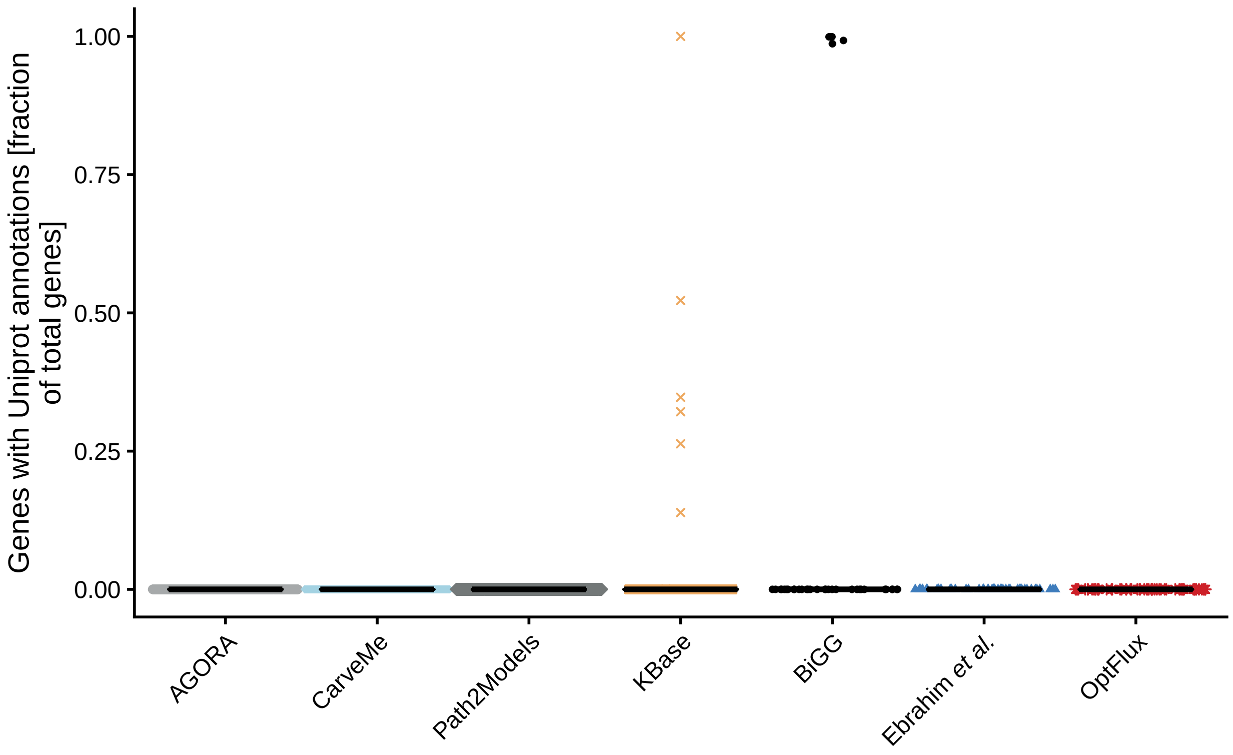

Figure 86: Gene UniProt Annotation

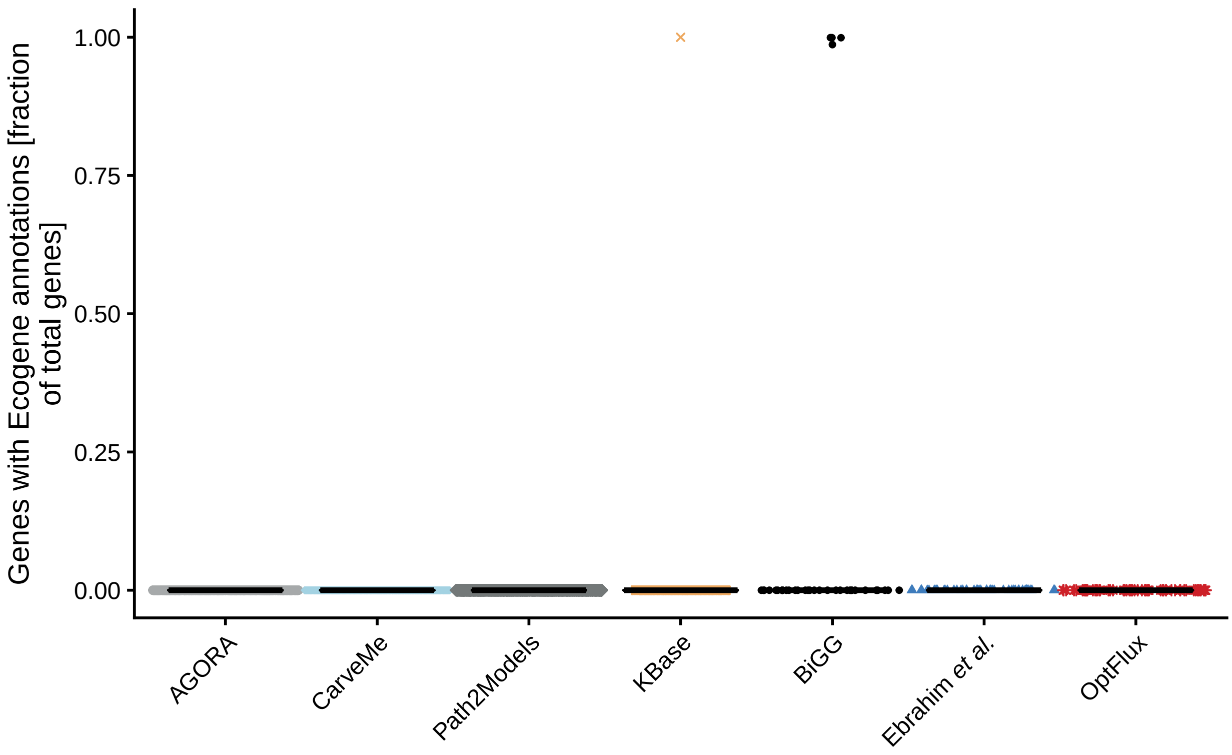

Figure 87: Gene EcoGene Annotation

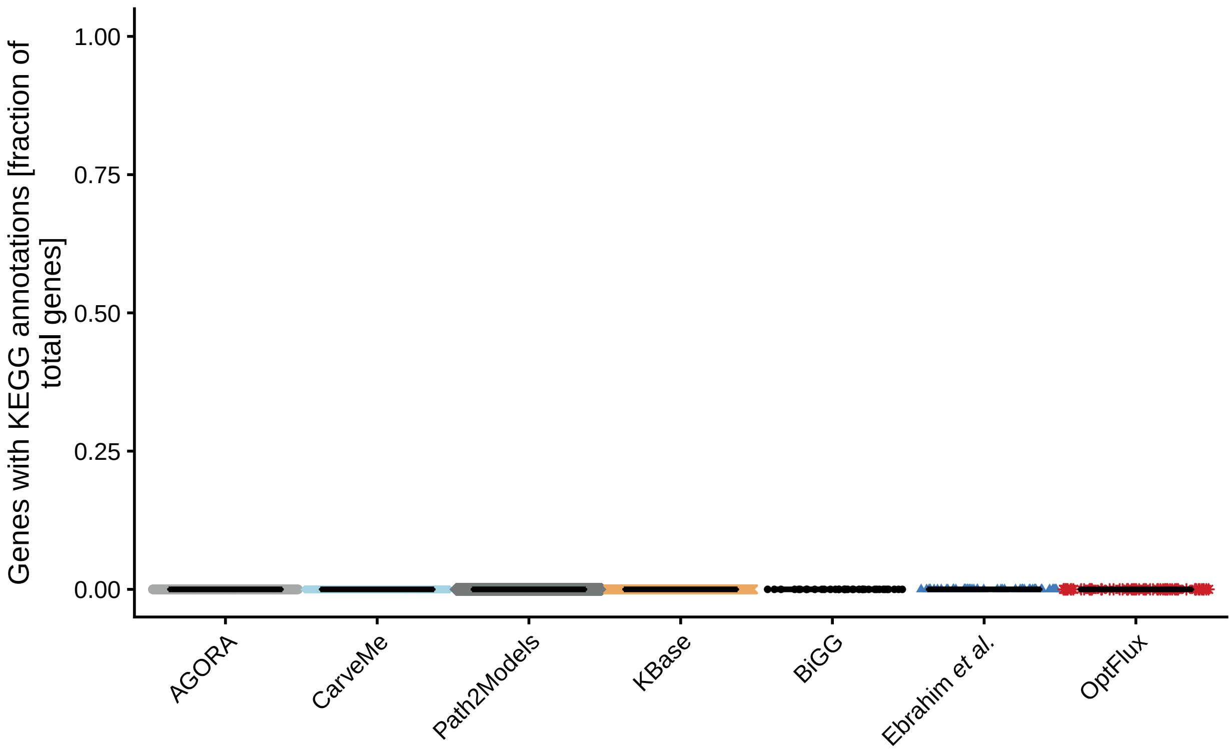

Figure 88: Gene KEGG.genes Annotation

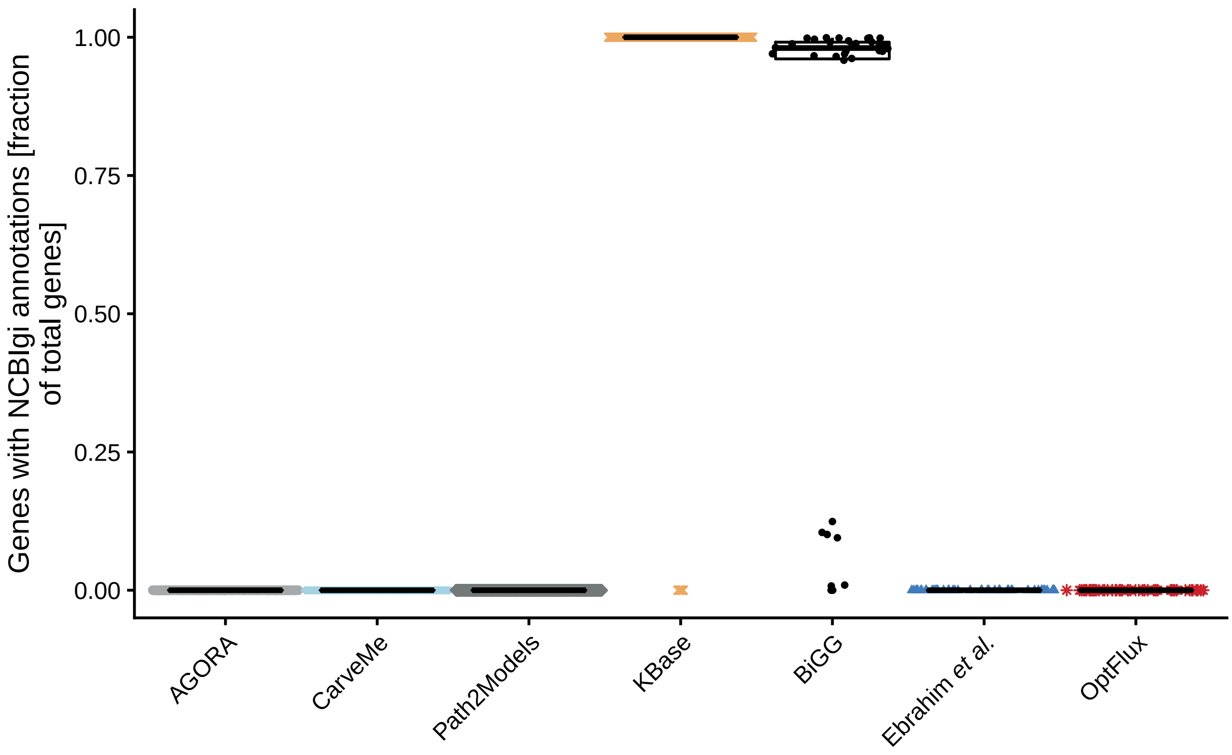

Figure 89: Gene NCBIgi Annotation

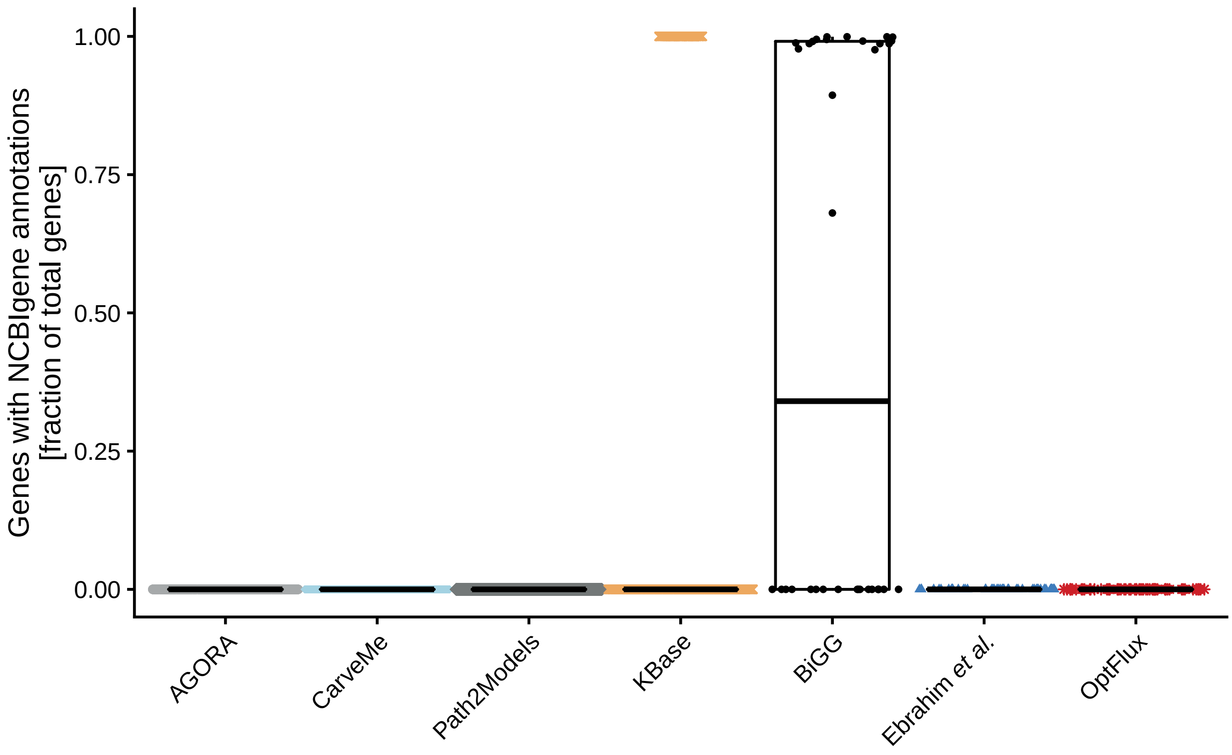

Figure 90: Gene NCBIgene Annotation

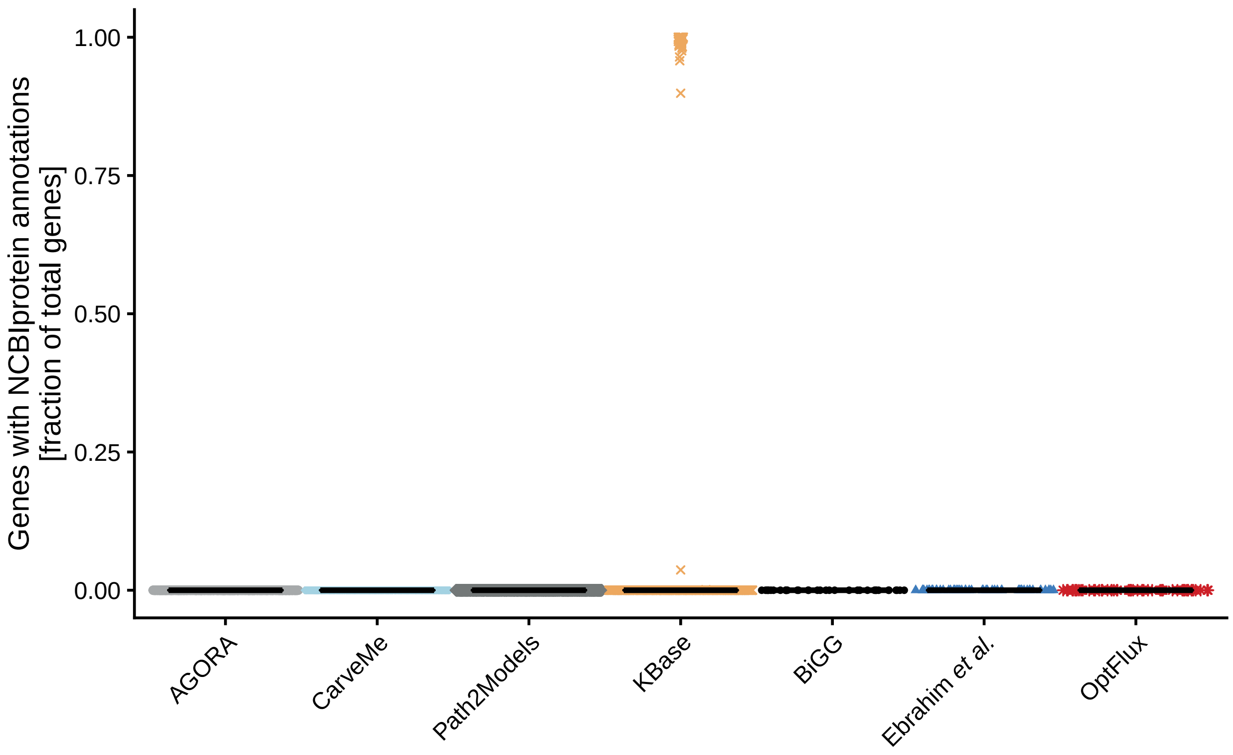

Figure 91: Gene NCBIprotein Annotation

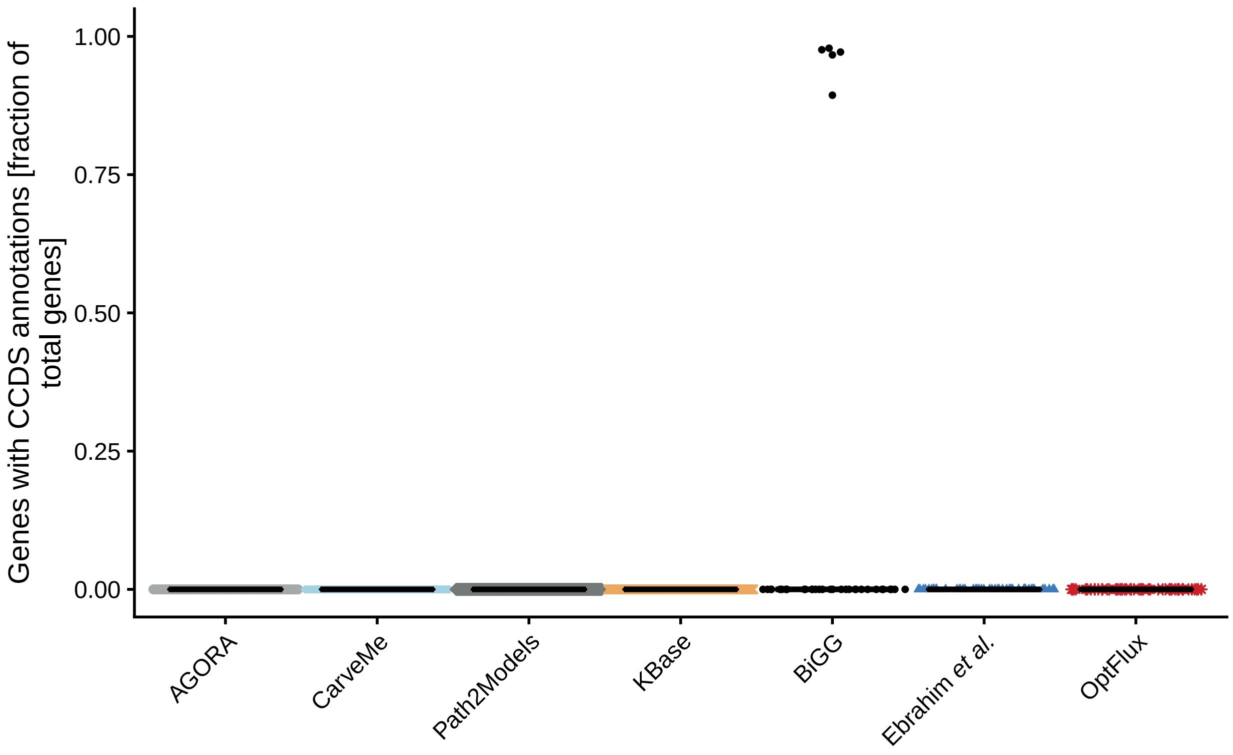

Figure 92: Gene CCDS Annotation

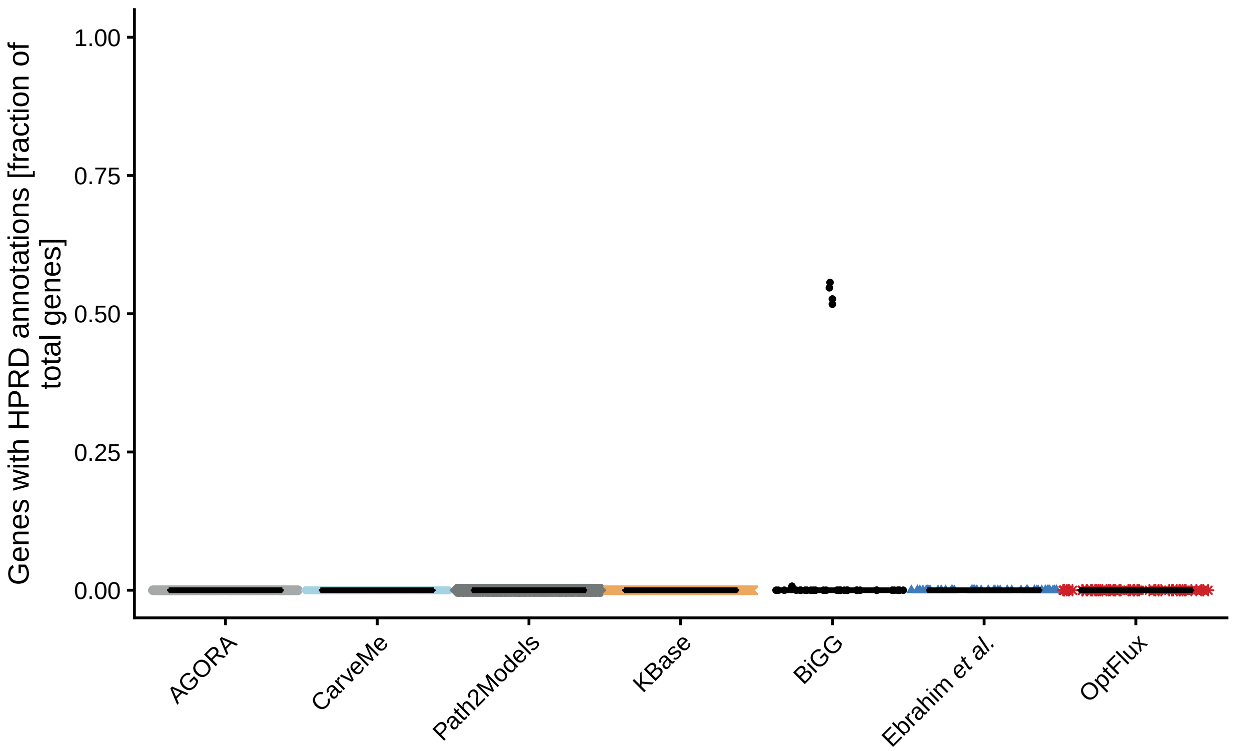

Figure 93: Gene HPRD Annotation

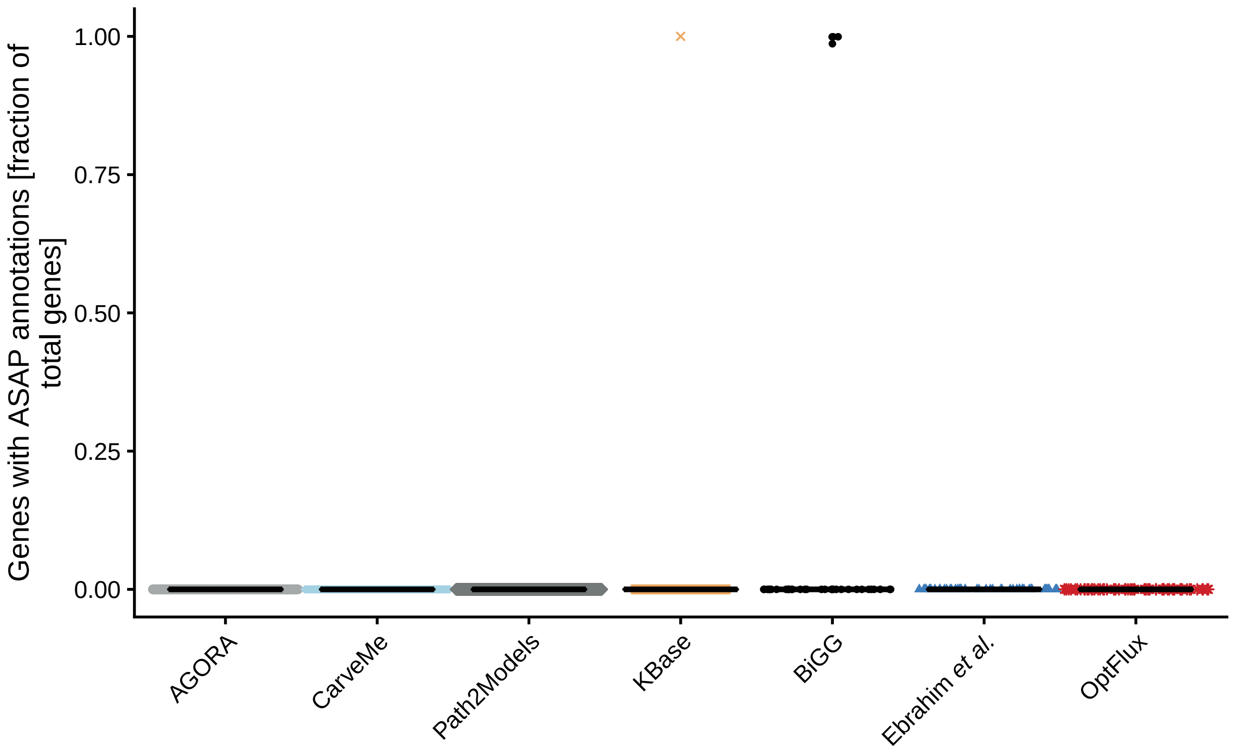

Figure 94: Gene ASAP Annotation

7.3.3.4.2 Gene Annotation Conformity Per Database

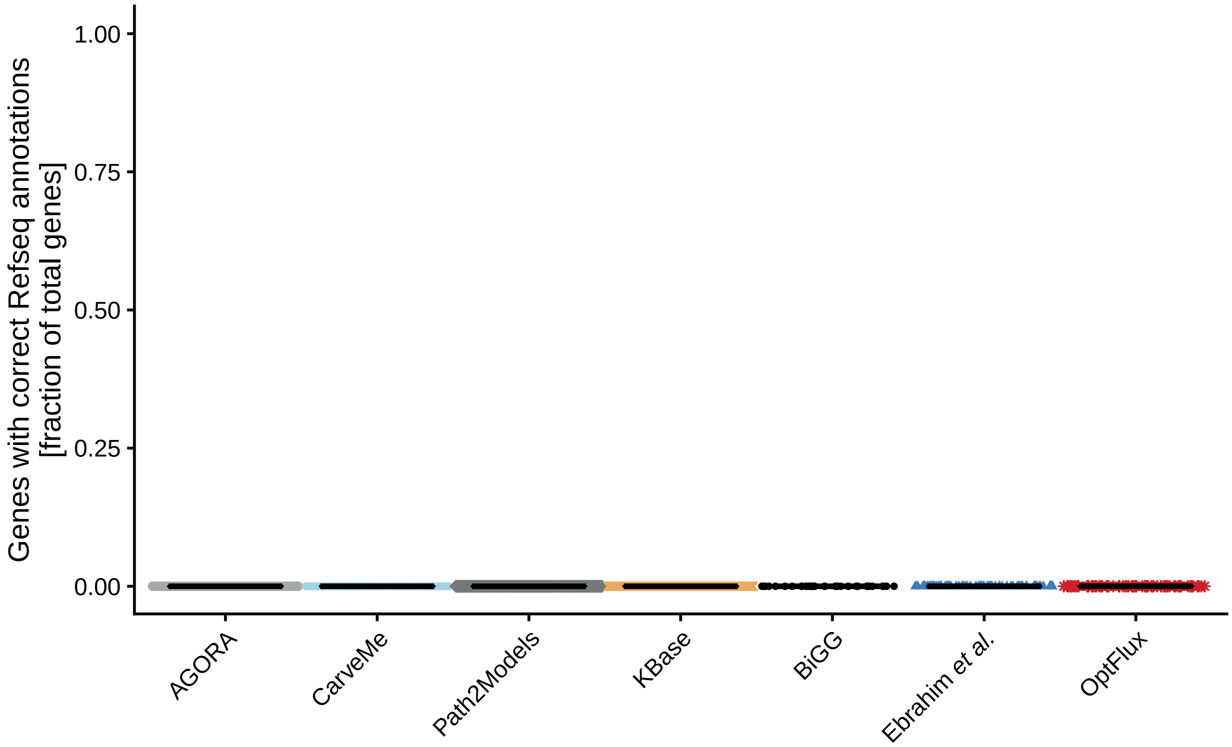

Figure 95: Correct Gene RefSeq Annotation

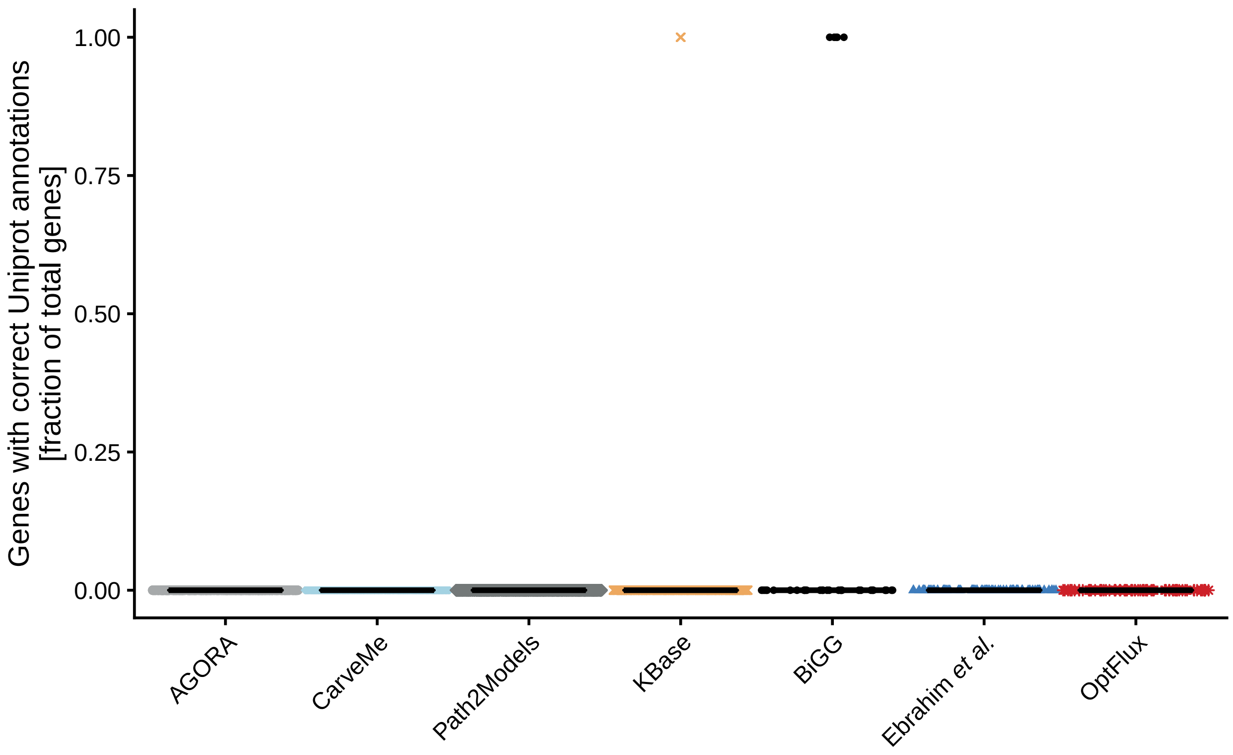

Figure 96: Correct Gene UniProt Annotation

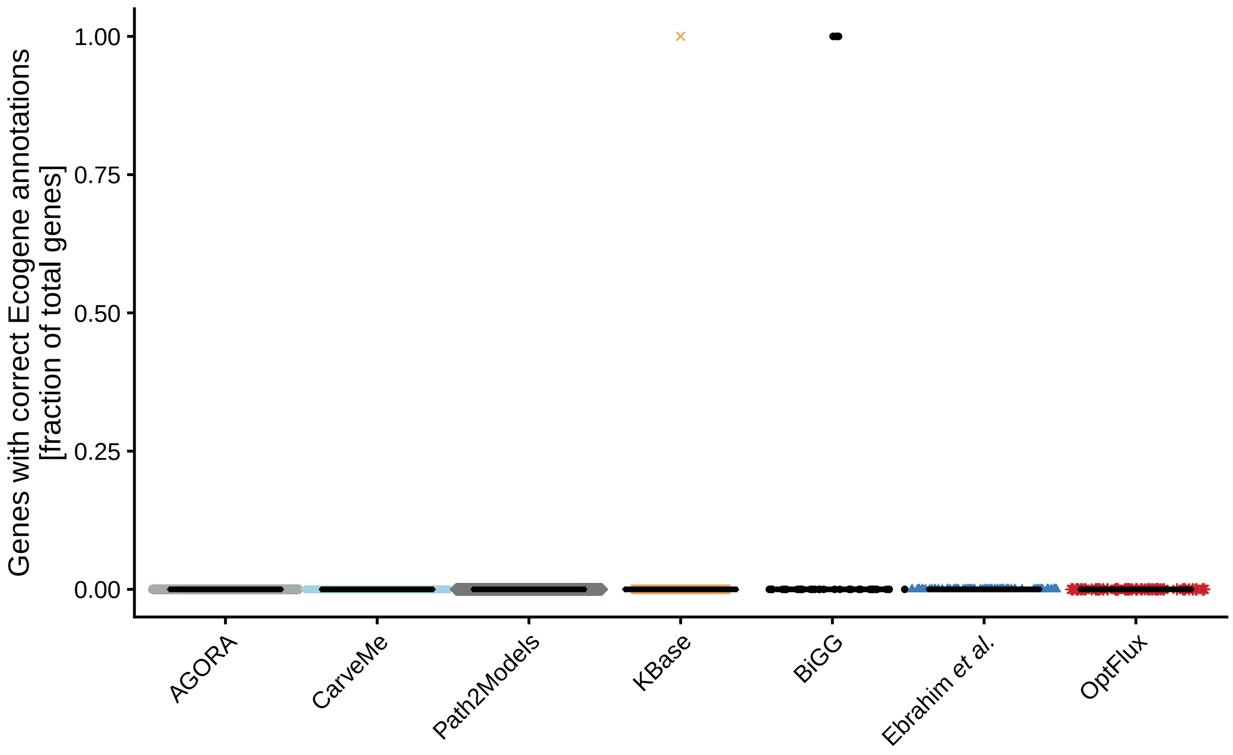

Figure 97: Correct Gene EcoGene Annotation

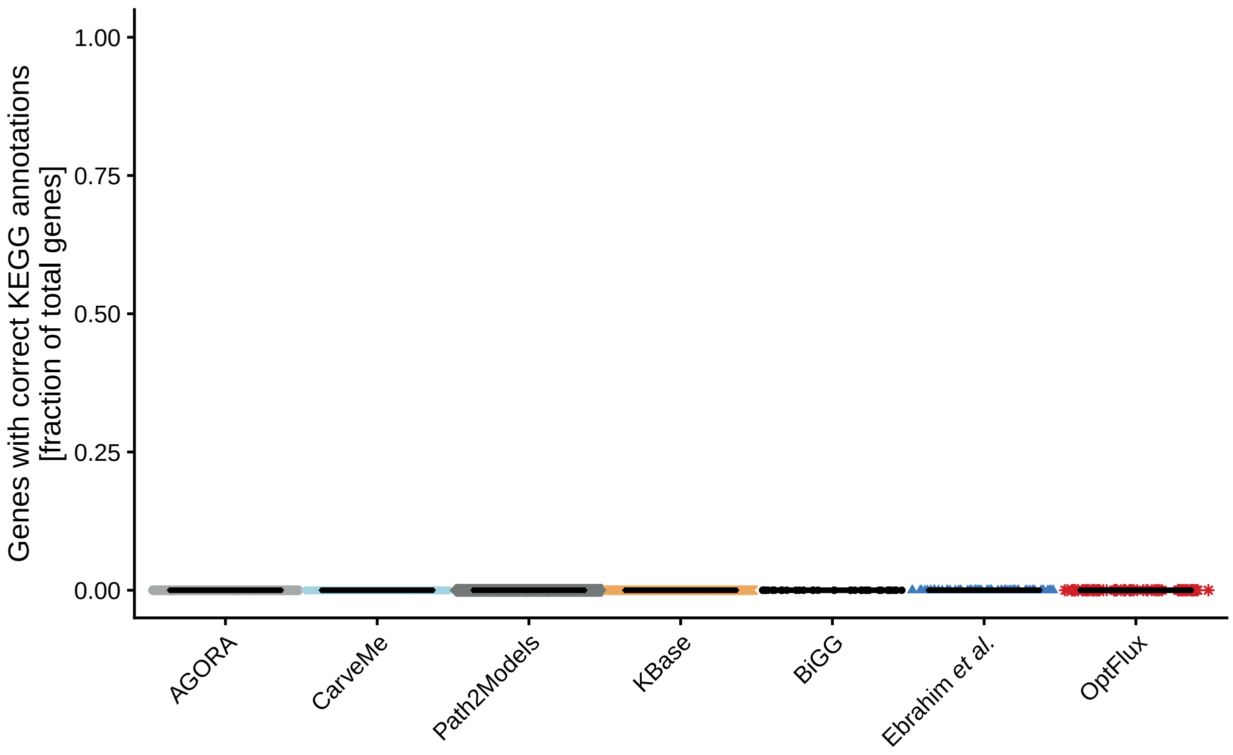

Figure 98: Correct Gene KEGG.genes Annotation

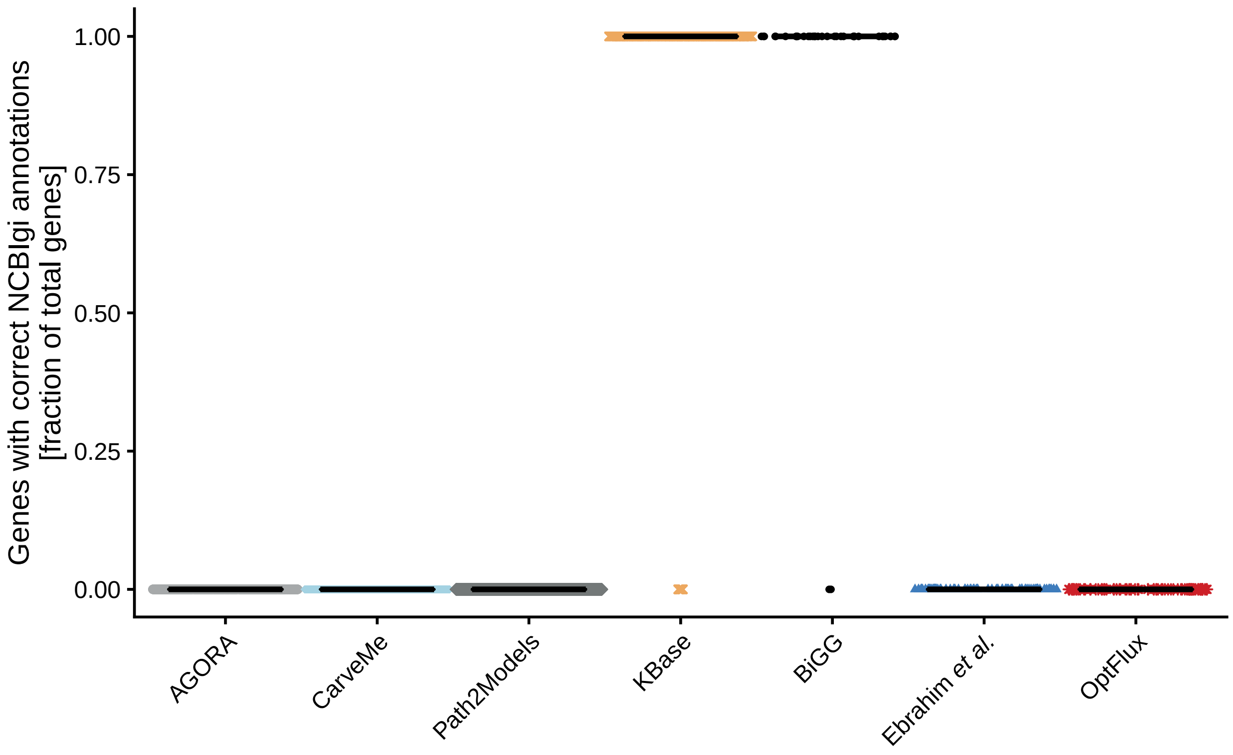

Figure 99: Correct Gene NCBIgi Annotation

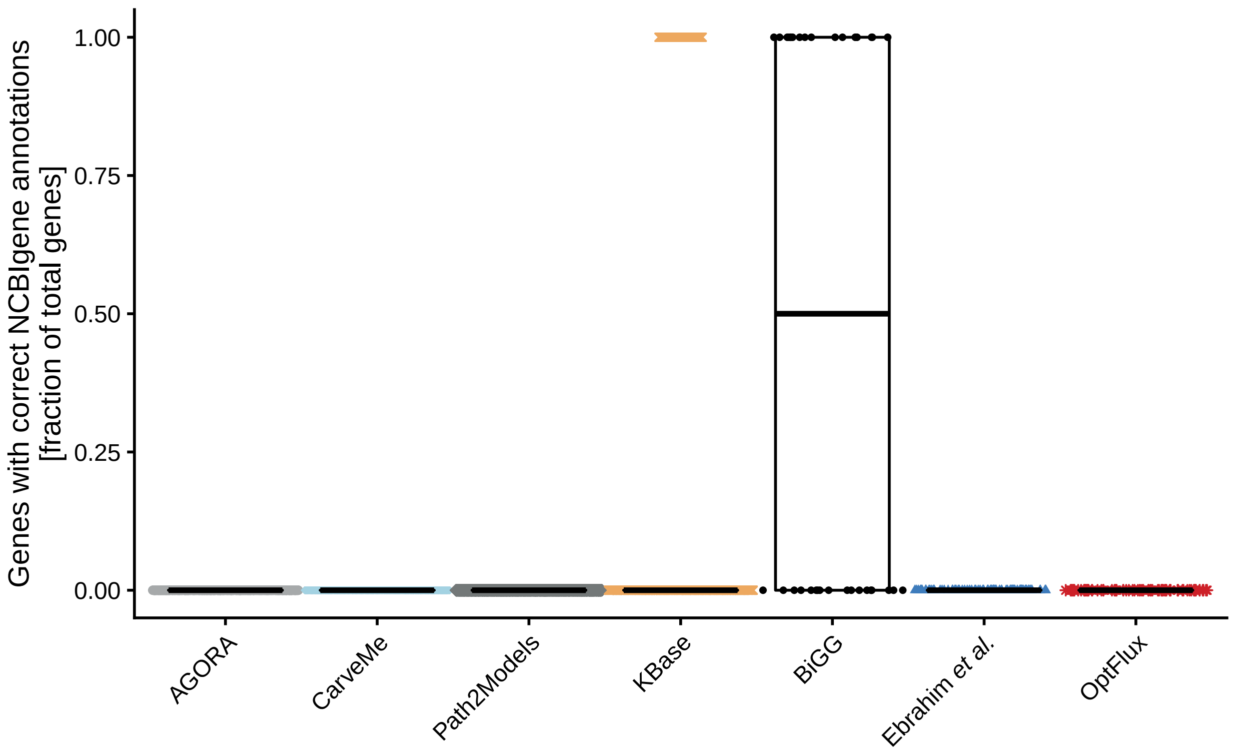

Figure 100: Correct Gene NCBIgene Annotation

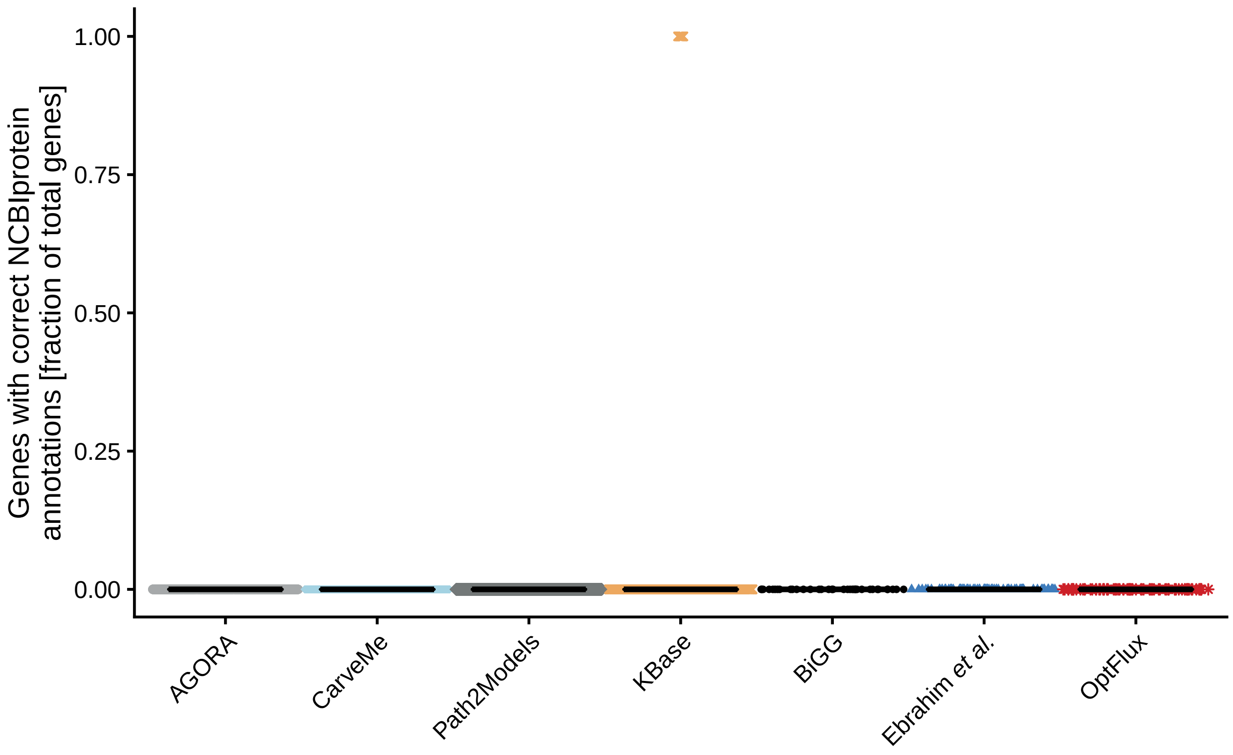

Figure 101: Correct Gene NCBIprotein Annotation

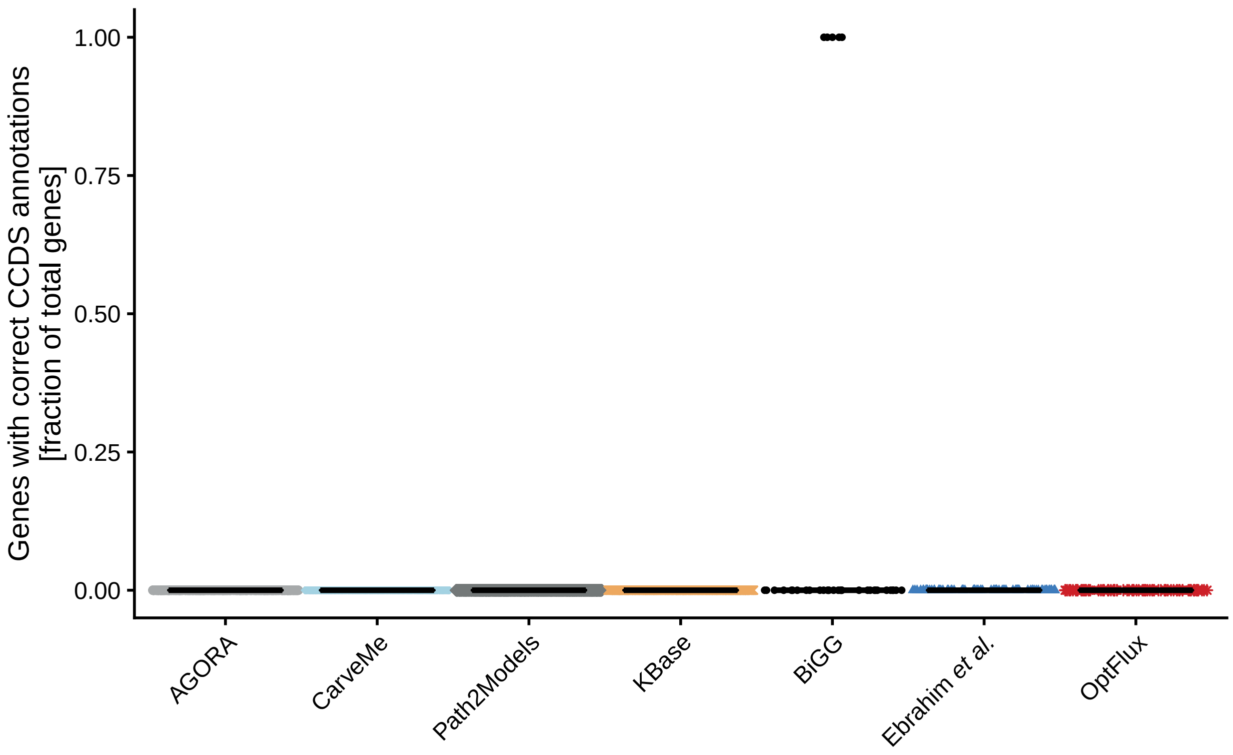

Figure 102: Correct Gene CCDS Annotation

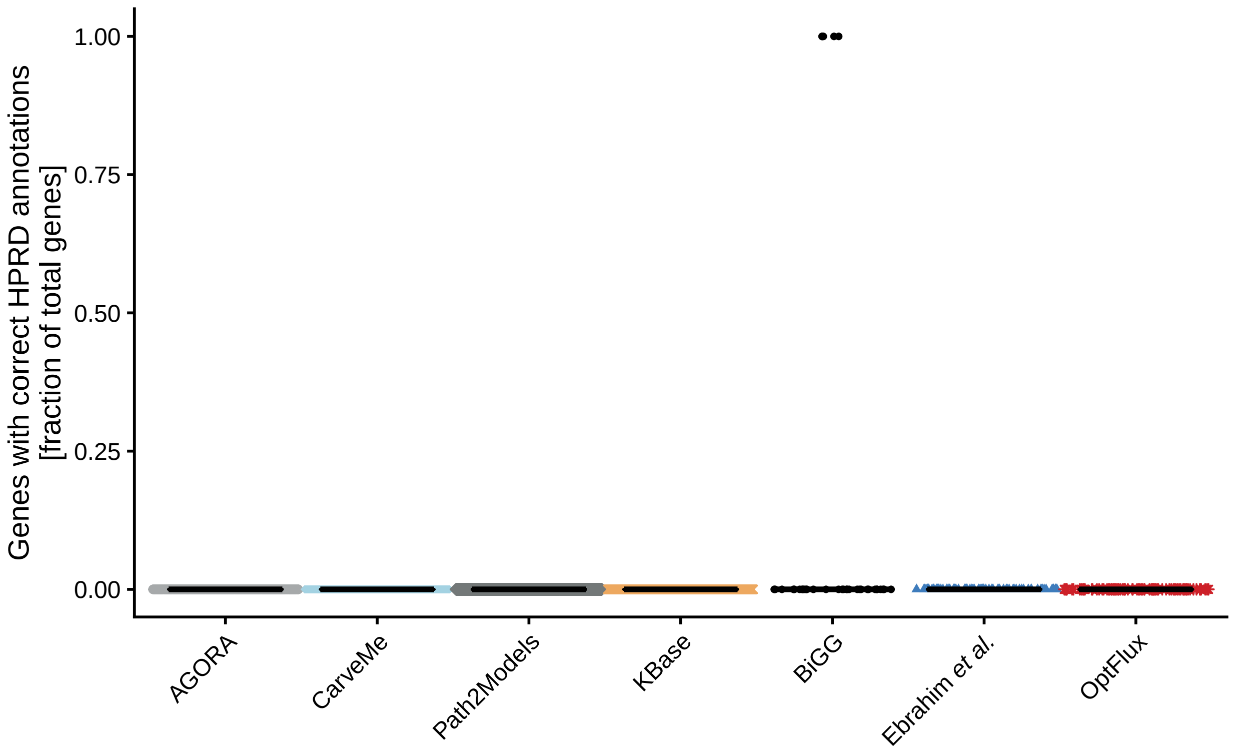

Figure 103: Correct Gene HPRD Annotation

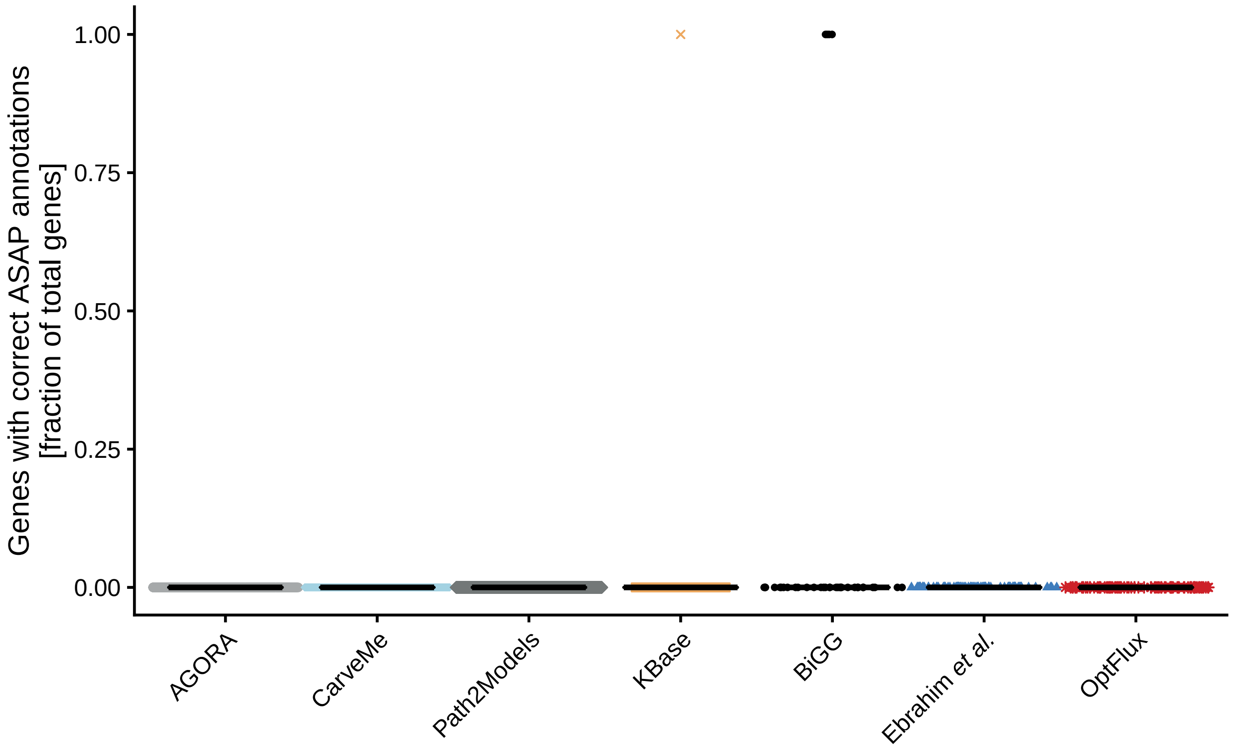

Figure 104: Correct Gene ASAP Annotation

7.3.3.5 Annotation - SBO Terms

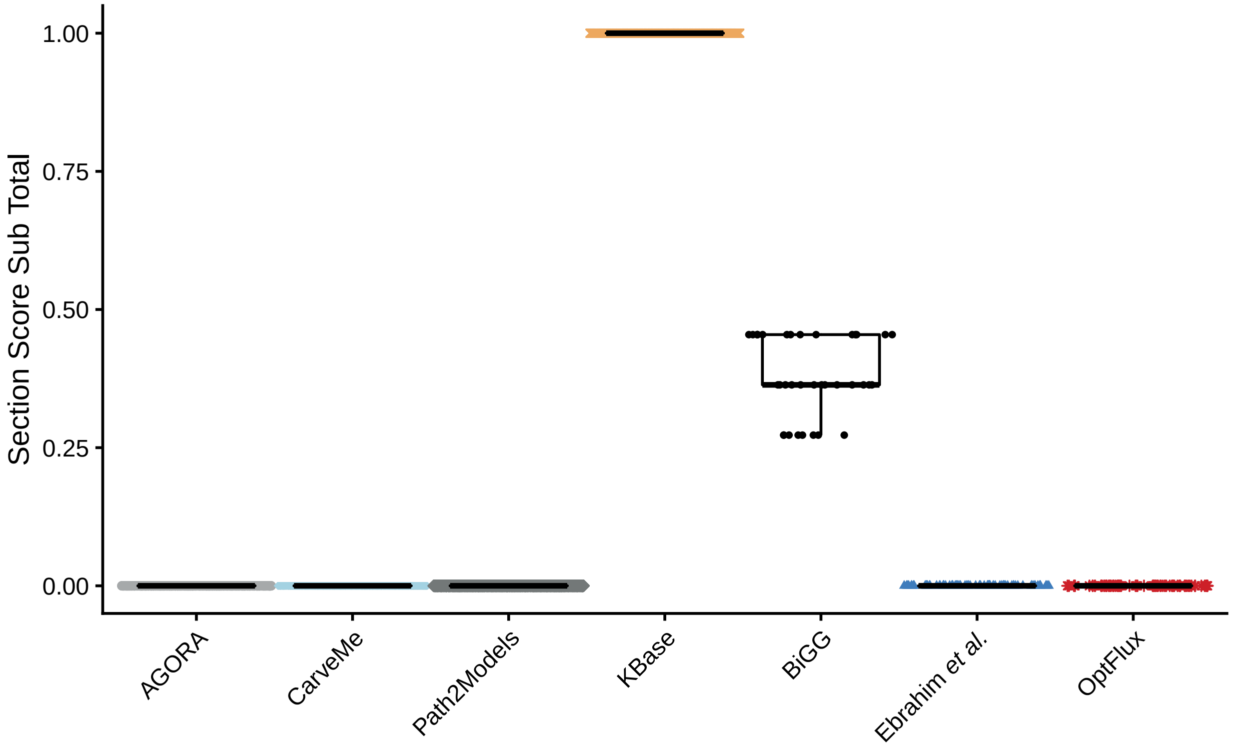

Figure 105: Annotation - SBO Terms. Depicted are the sums of all test scores in this section, applying the weights of the individual test cases as detailed in the snapshot report.

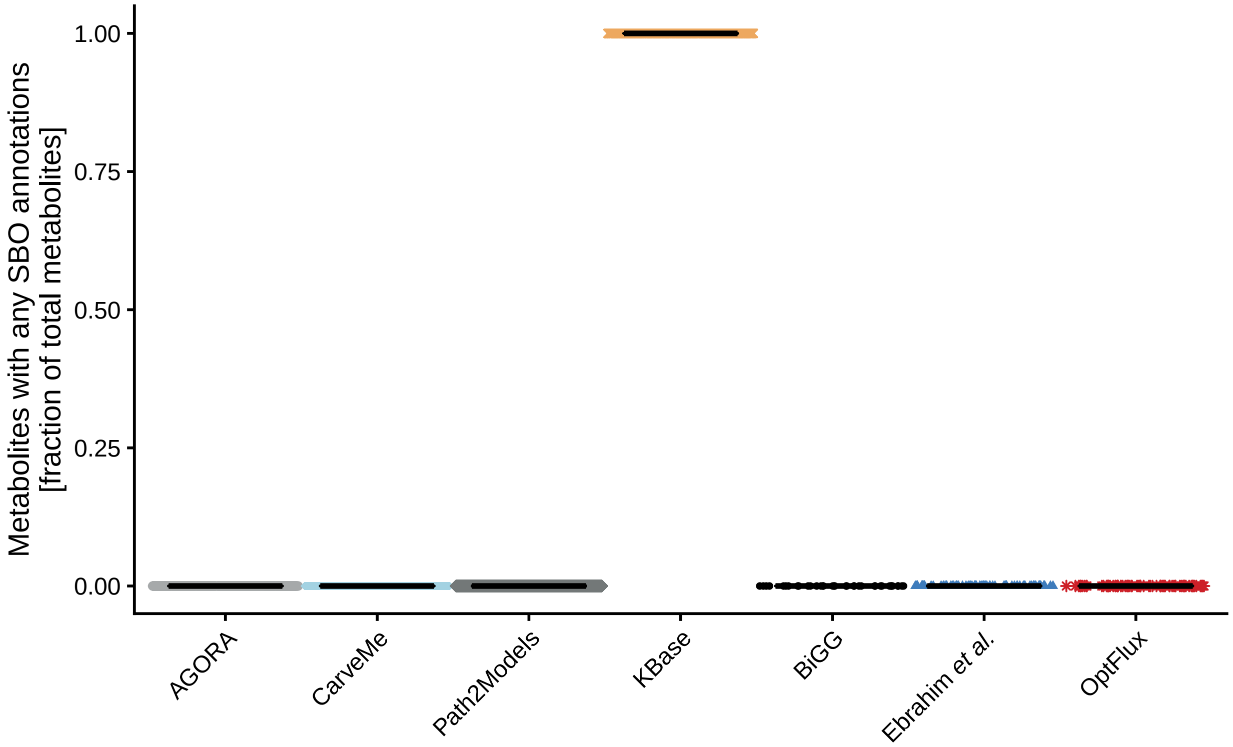

Figure 106: Metabolite General SBO Presence

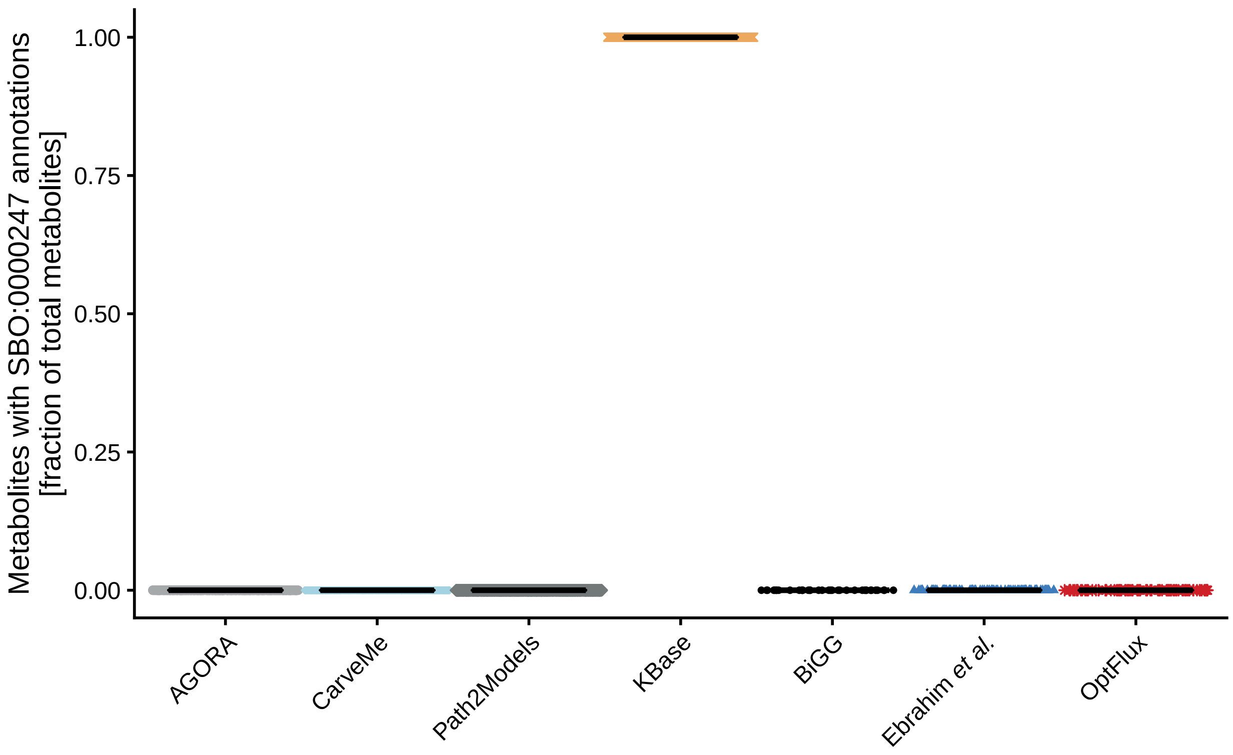

Figure 107: Metabolite SBO:0000247 Presence

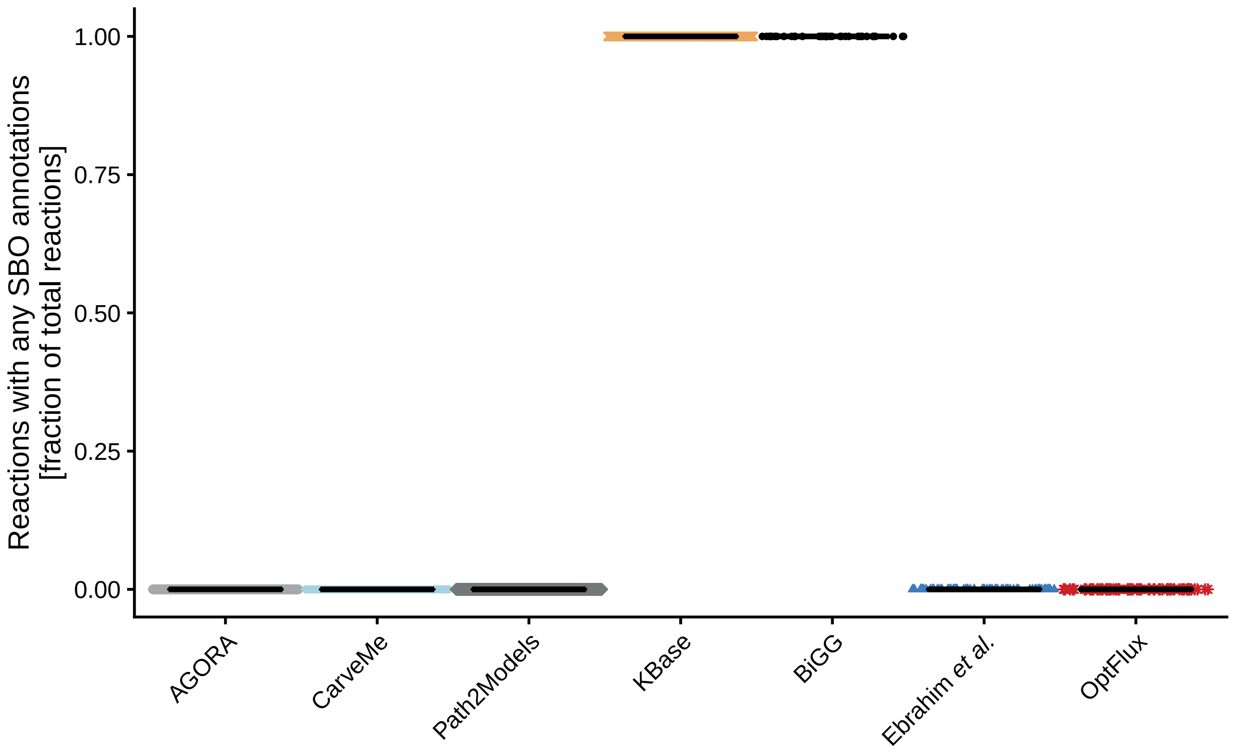

Figure 108: Reaction General SBO Presence

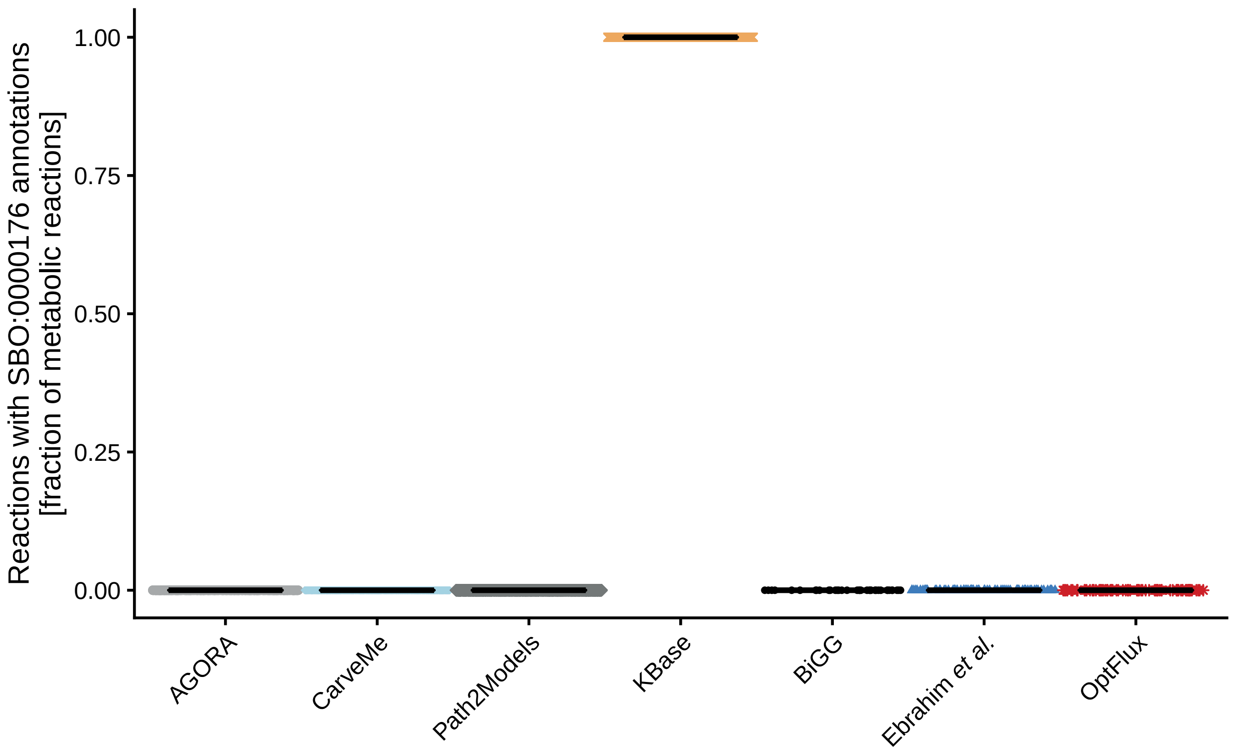

Figure 109: Metabolic Reaction SBO:0000176 Presence

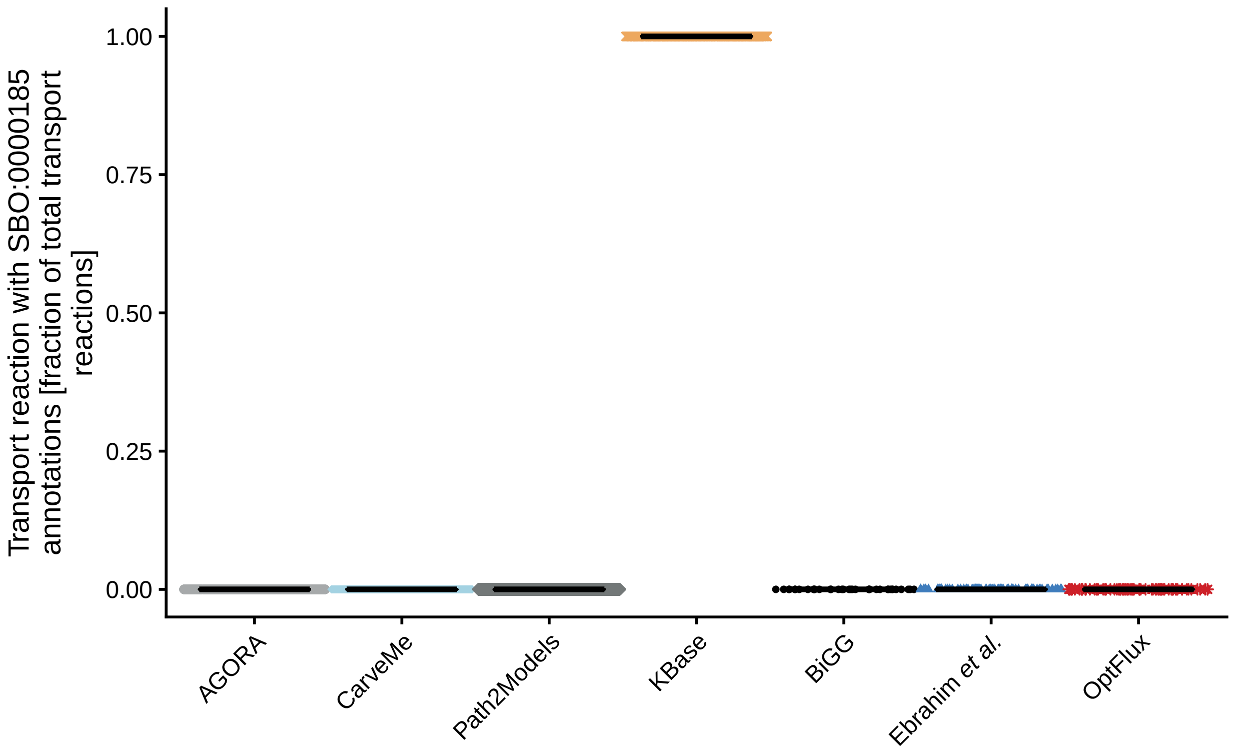

Figure 110: Transport Reaction SBO:0000185 Presence

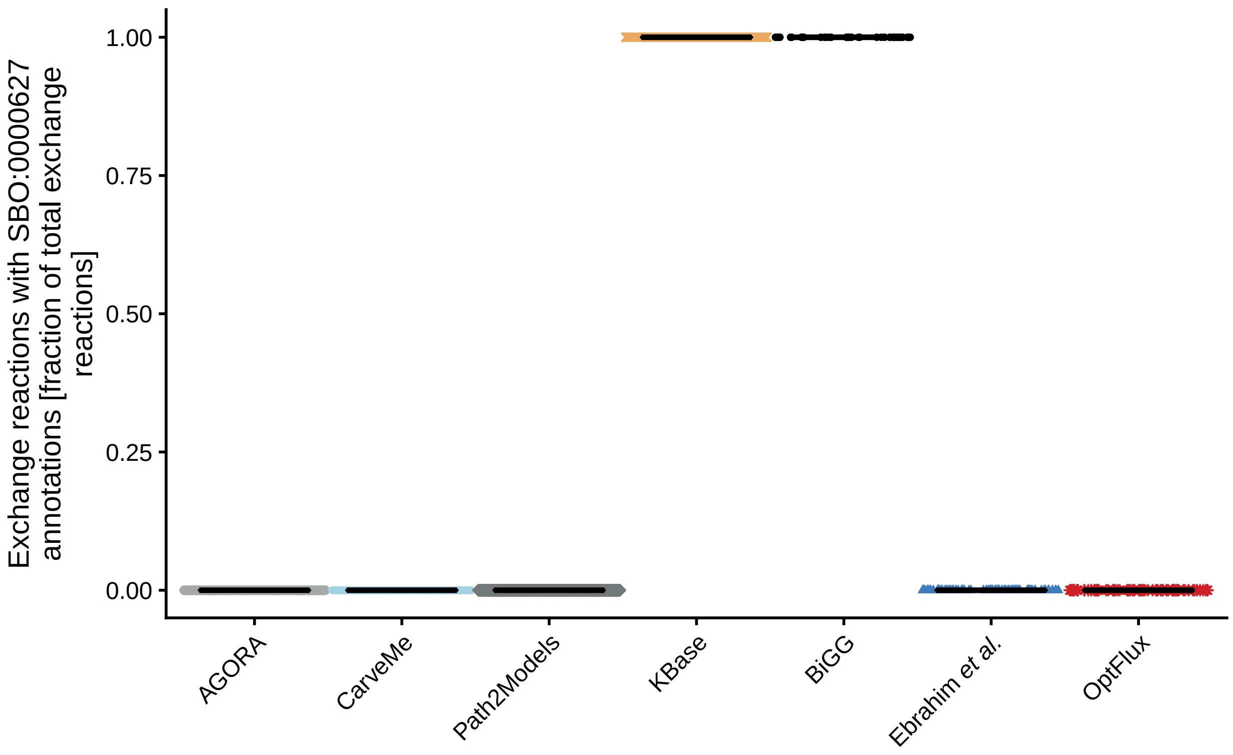

Figure 111: Exchange Reaction SBO:0000627 Presence

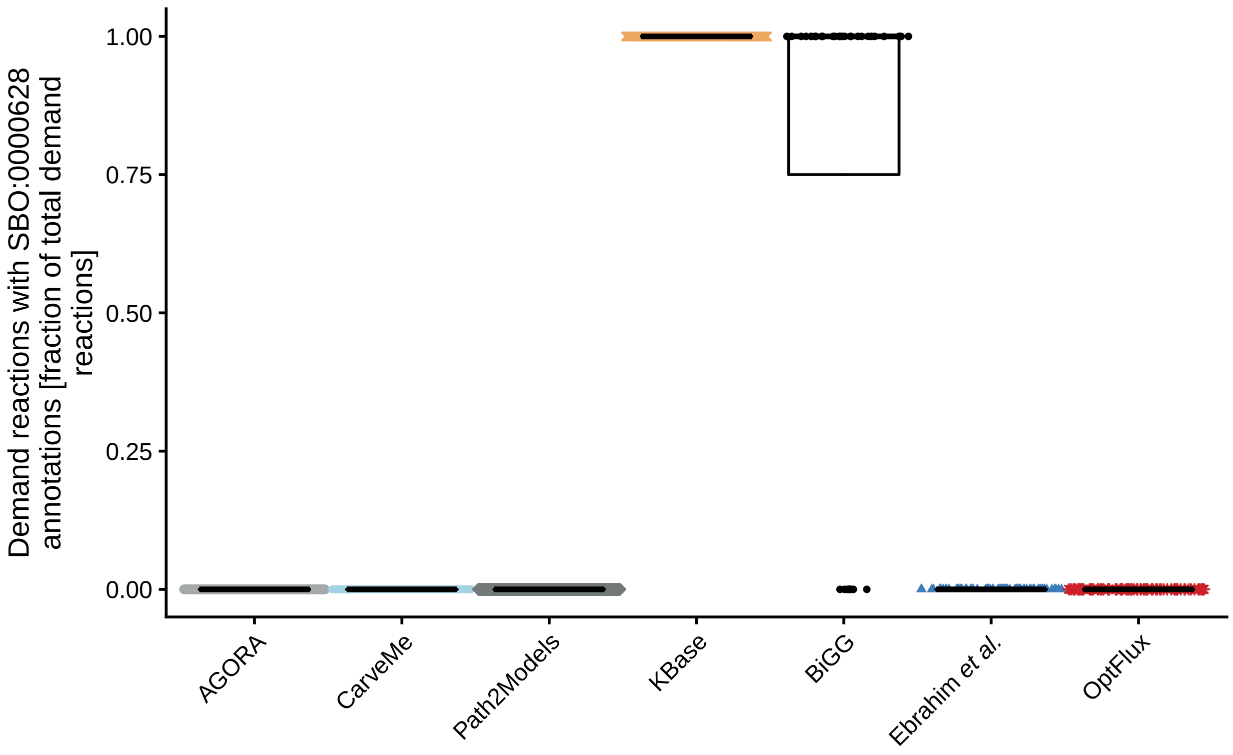

Figure 112: Demand Reaction SBO:0000628 Presence

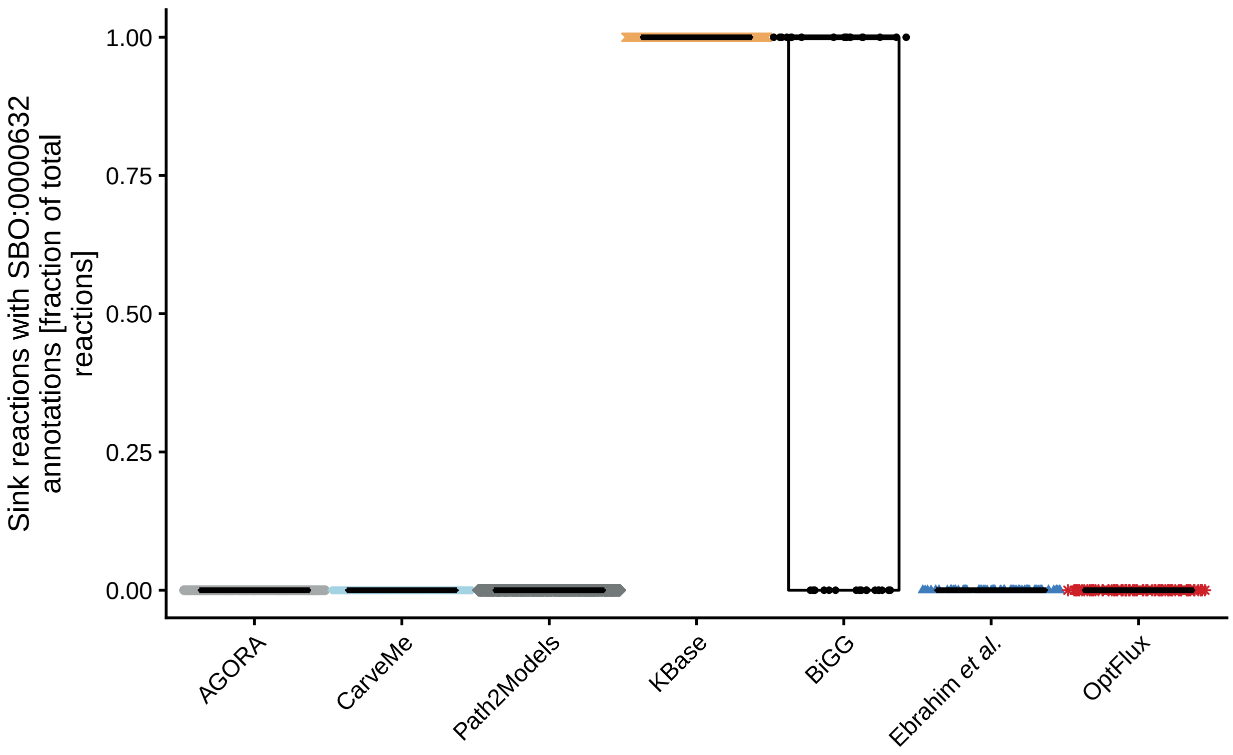

Figure 113: Sink Reaction SBO:0000632 Presence

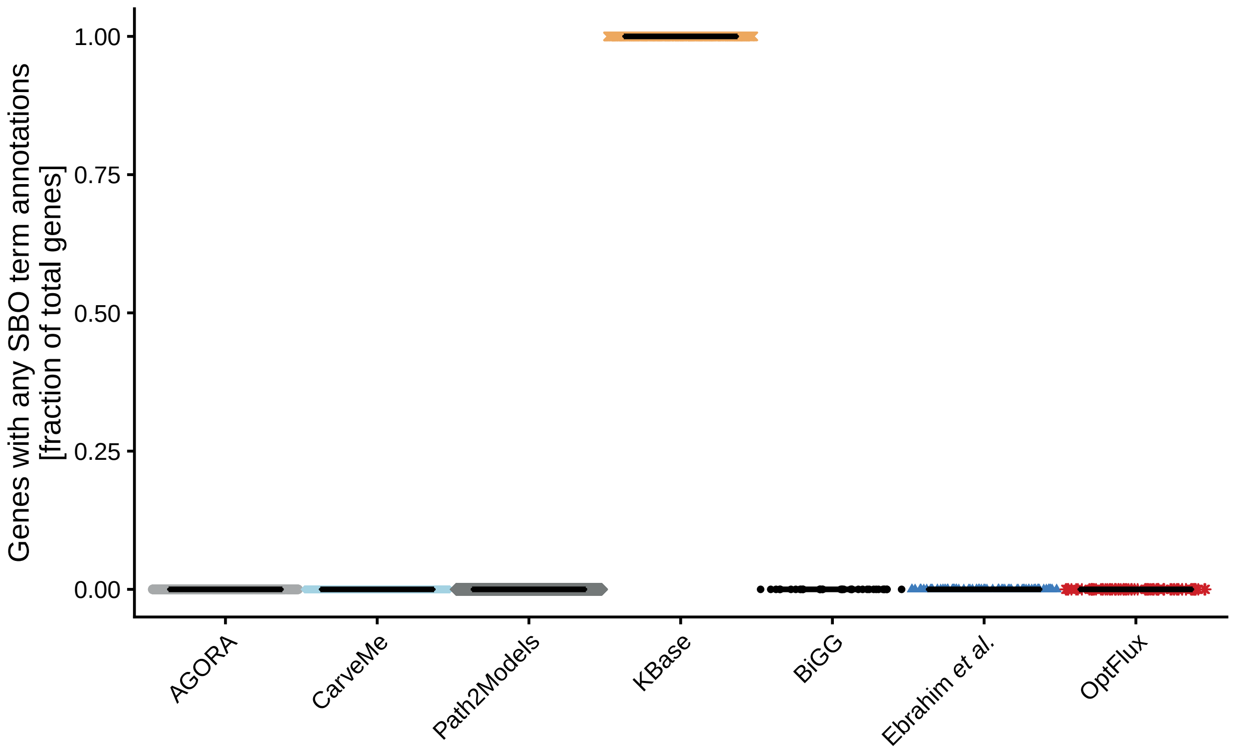

Figure 114: Gene General SBO Presence

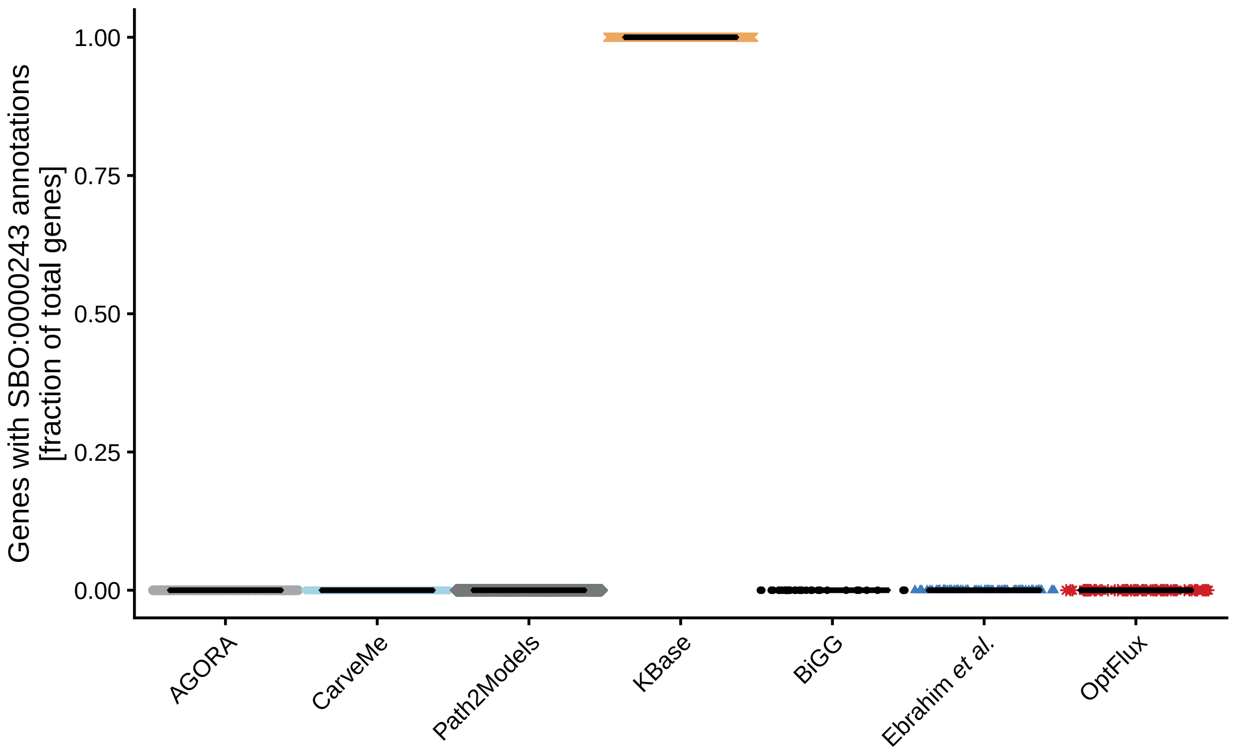

Figure 115: Gene SBO:0000243 Presence

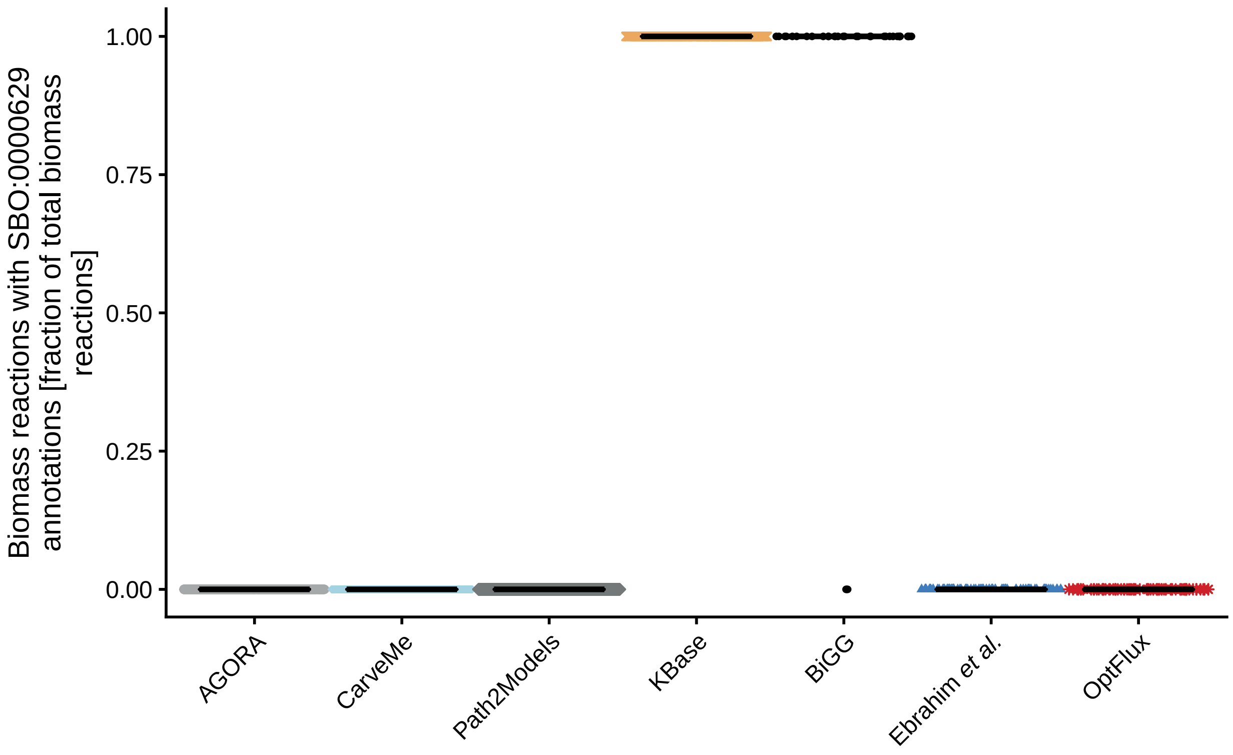

Figure 116: Biomass Reaction SBO:0000629 Presence

7.3.4 Specific Section

7.3.4.1 SBML

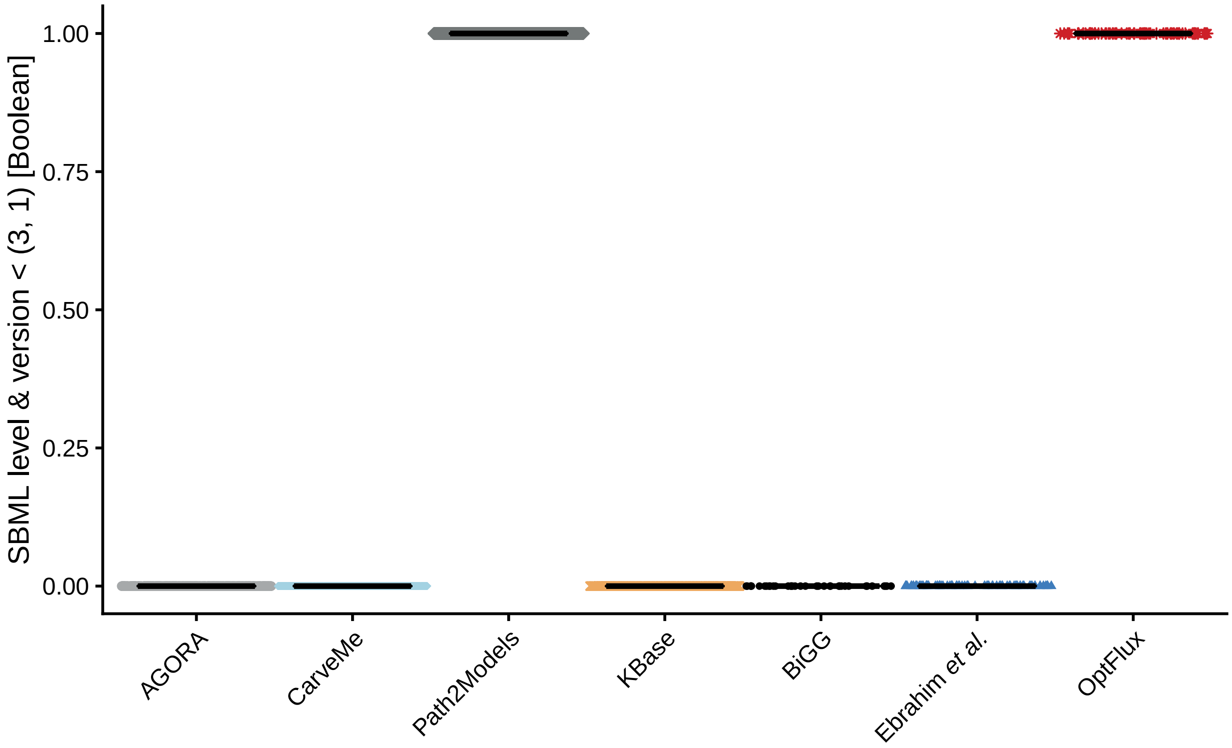

Figure 117: SBML Level and Version

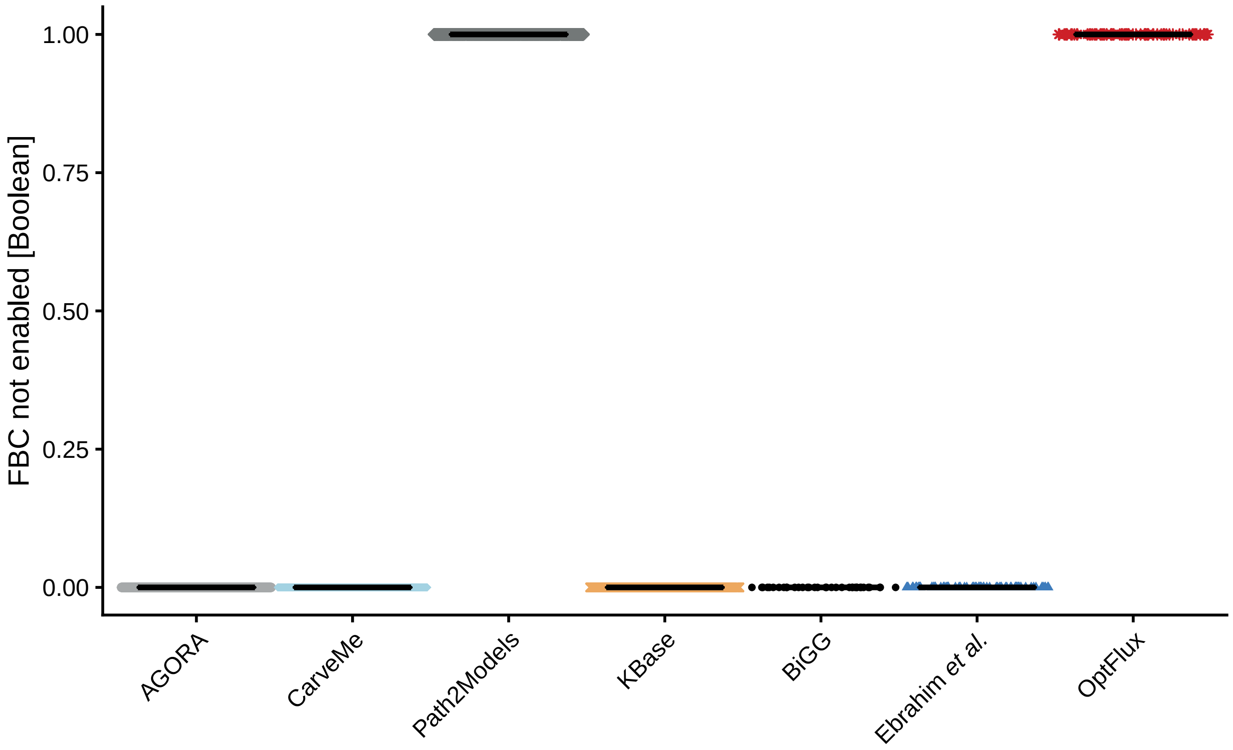

Figure 118: FBC not Enabled

7.3.4.2 Basic Information

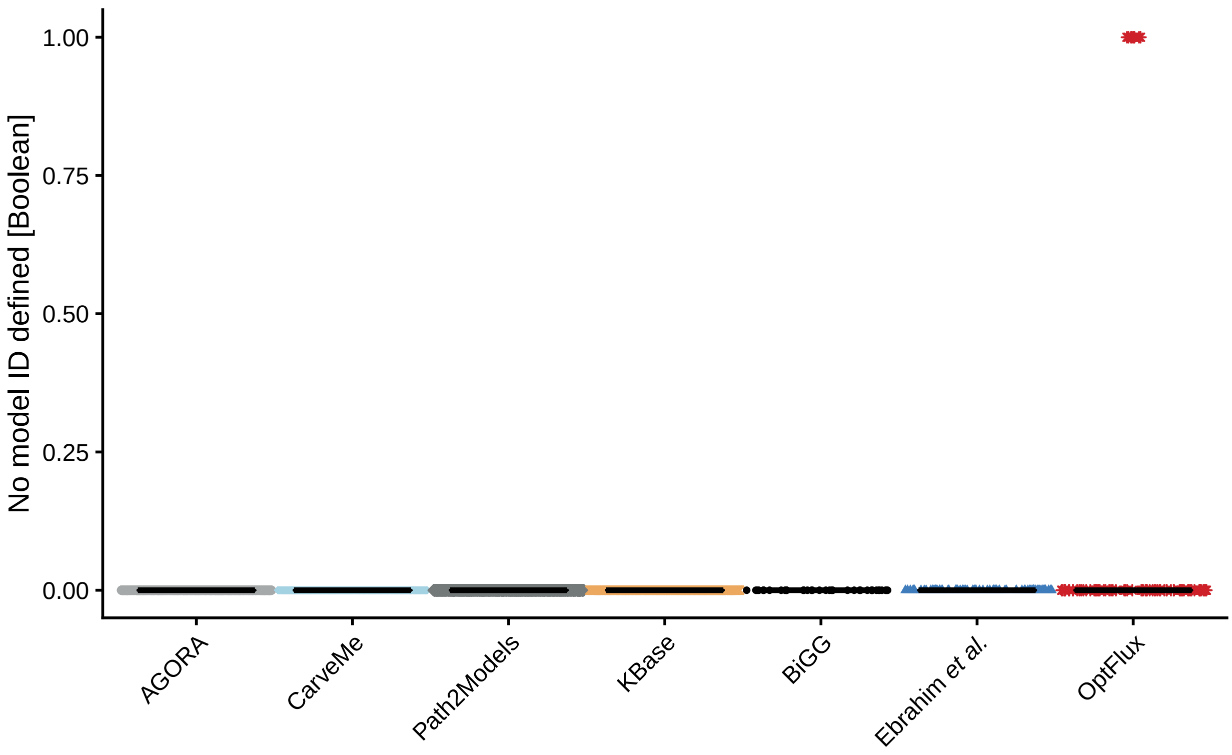

Figure 119: Model Identifier Presence

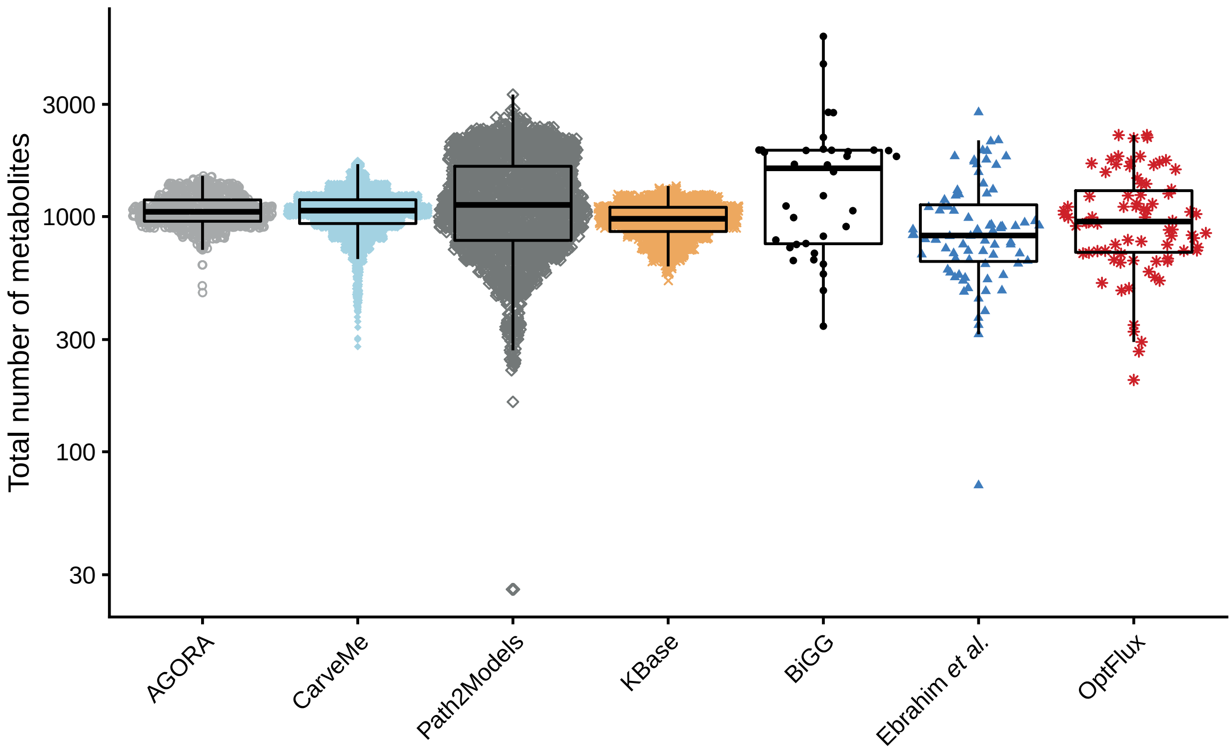

Figure 120: Number of Metabolites

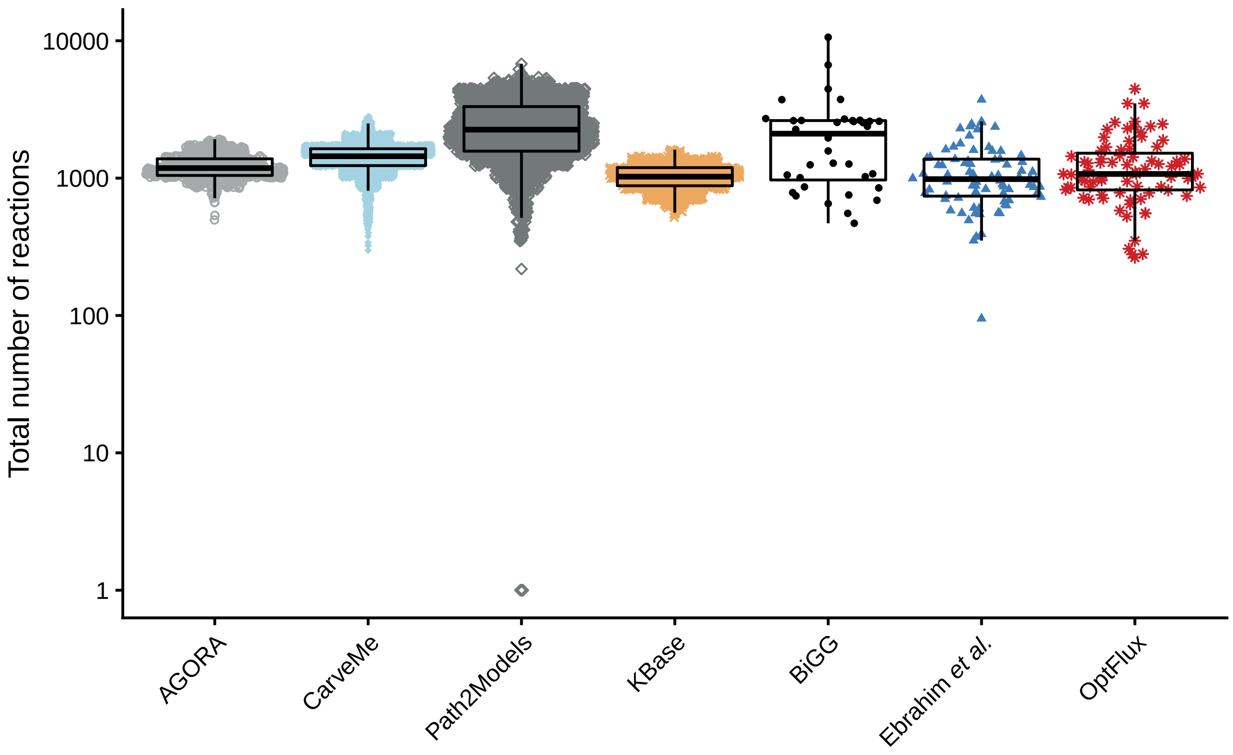

Figure 121: Number of Reactions

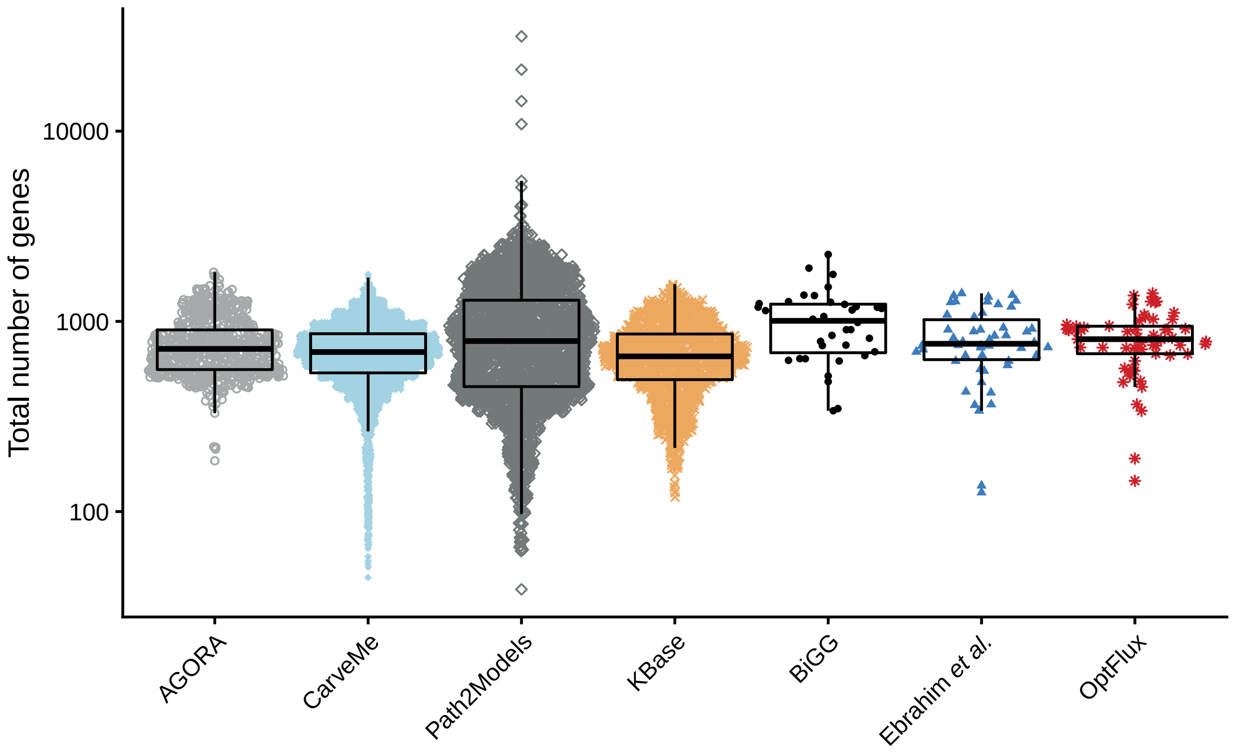

Figure 122: Number of Genes

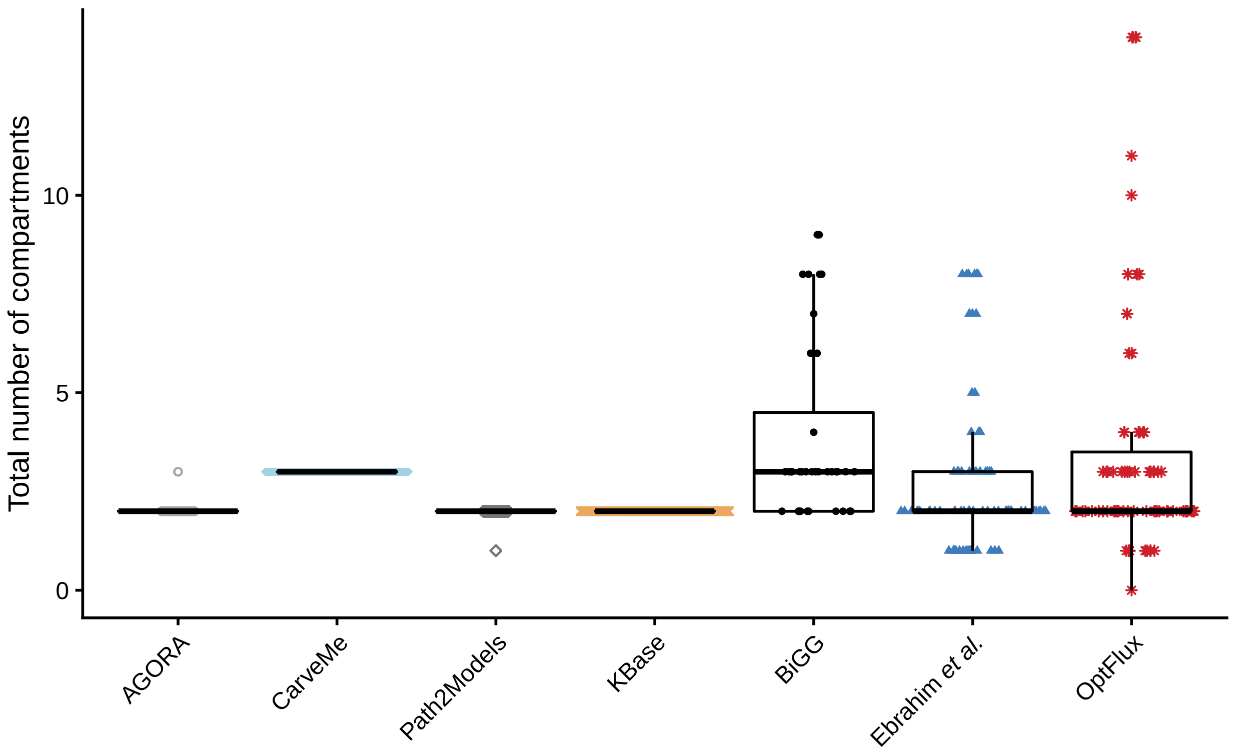

Figure 123: Number of Compartments

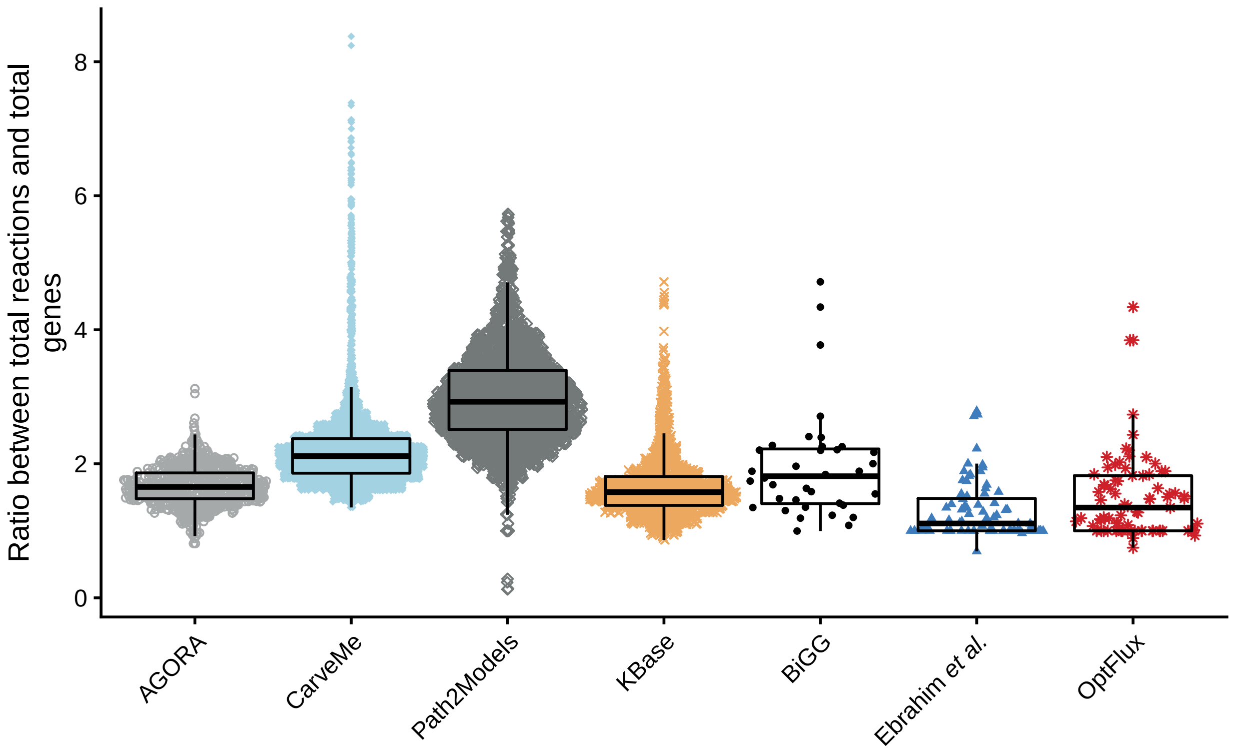

Figure 124: Metabolic Coverage

7.3.4.3 Metabolite Information

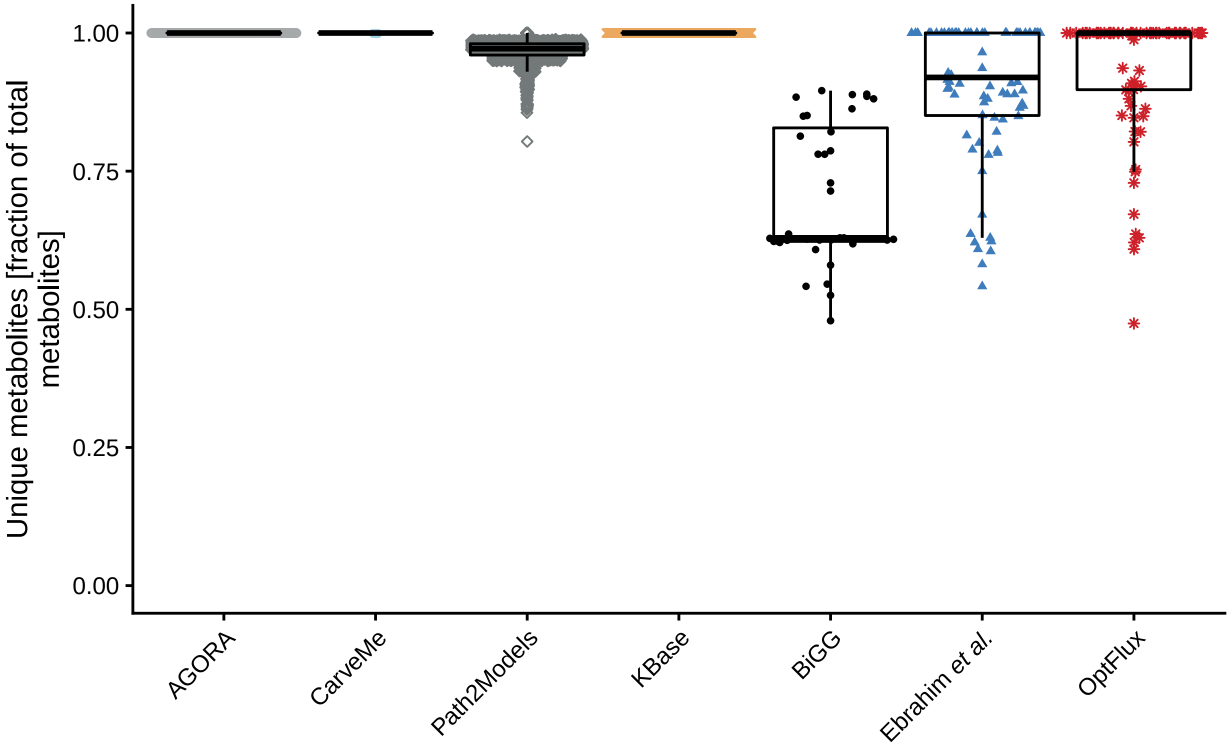

Figure 125: Unique Metabolites

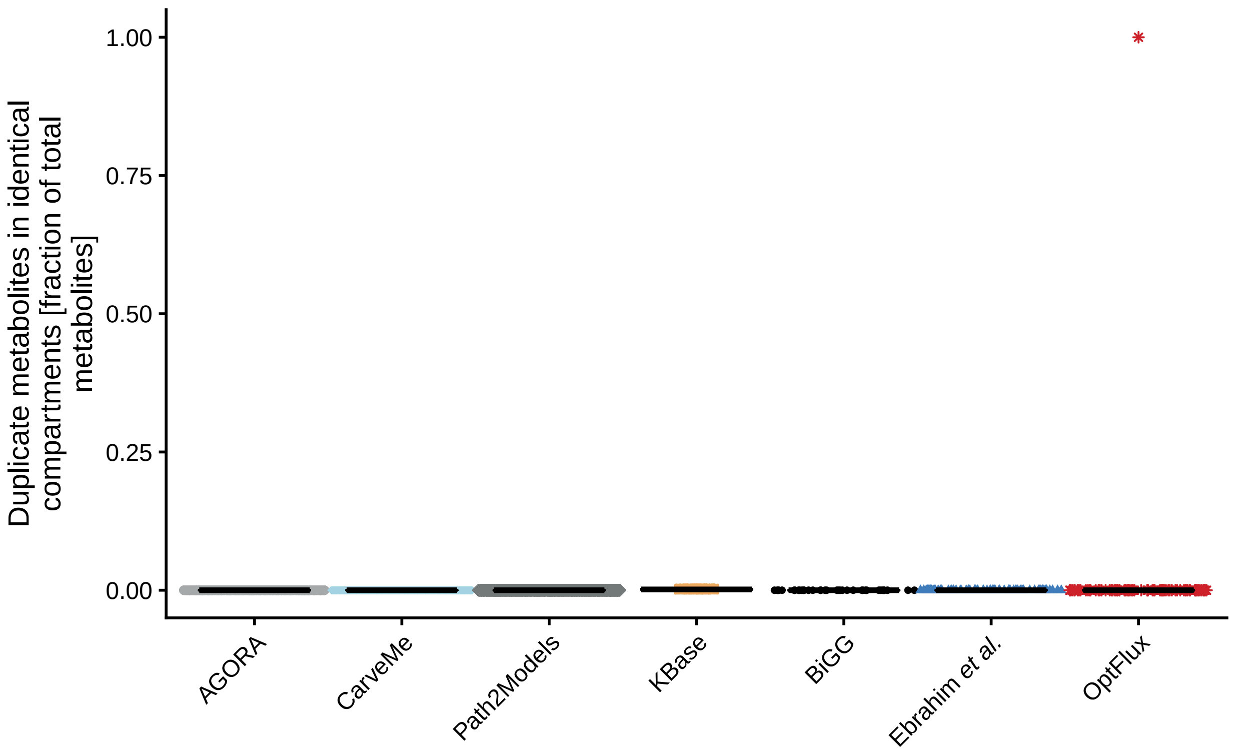

Figure 126: Duplicate Metabolites in Identical Compartments

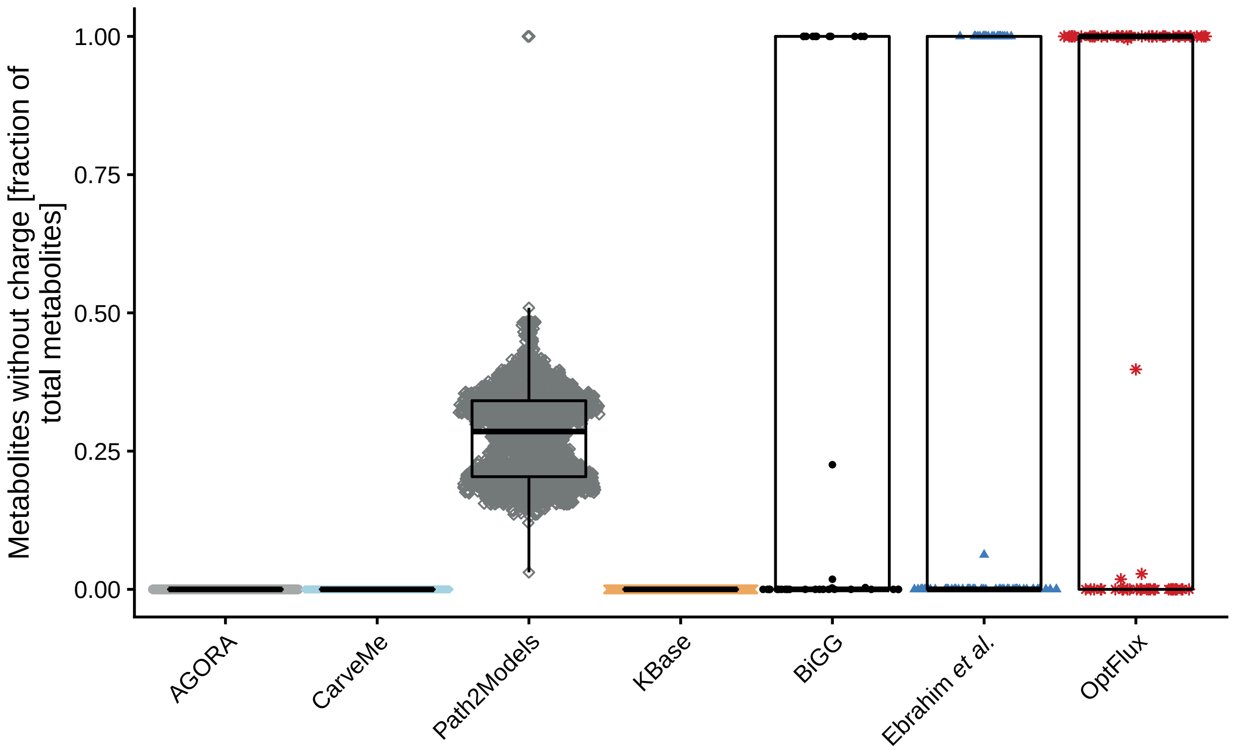

Figure 127: Metabolites Without Charge

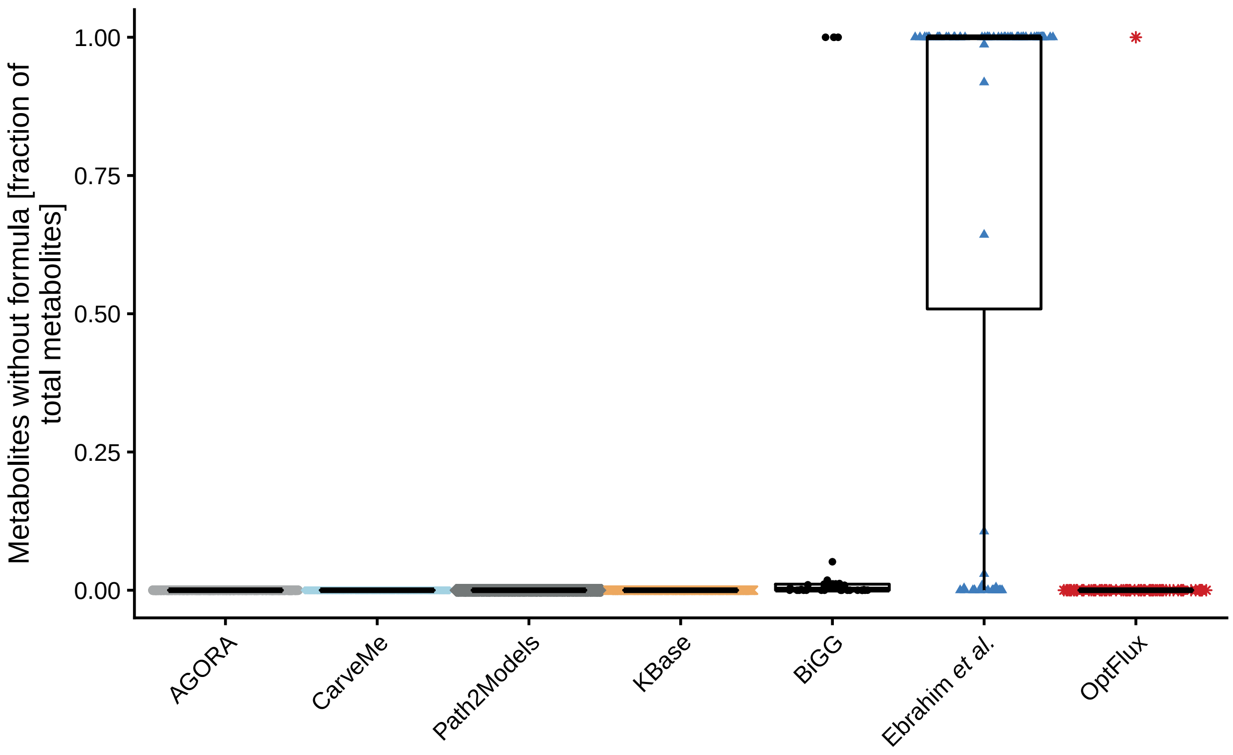

Figure 128: Metabolites Without Formula

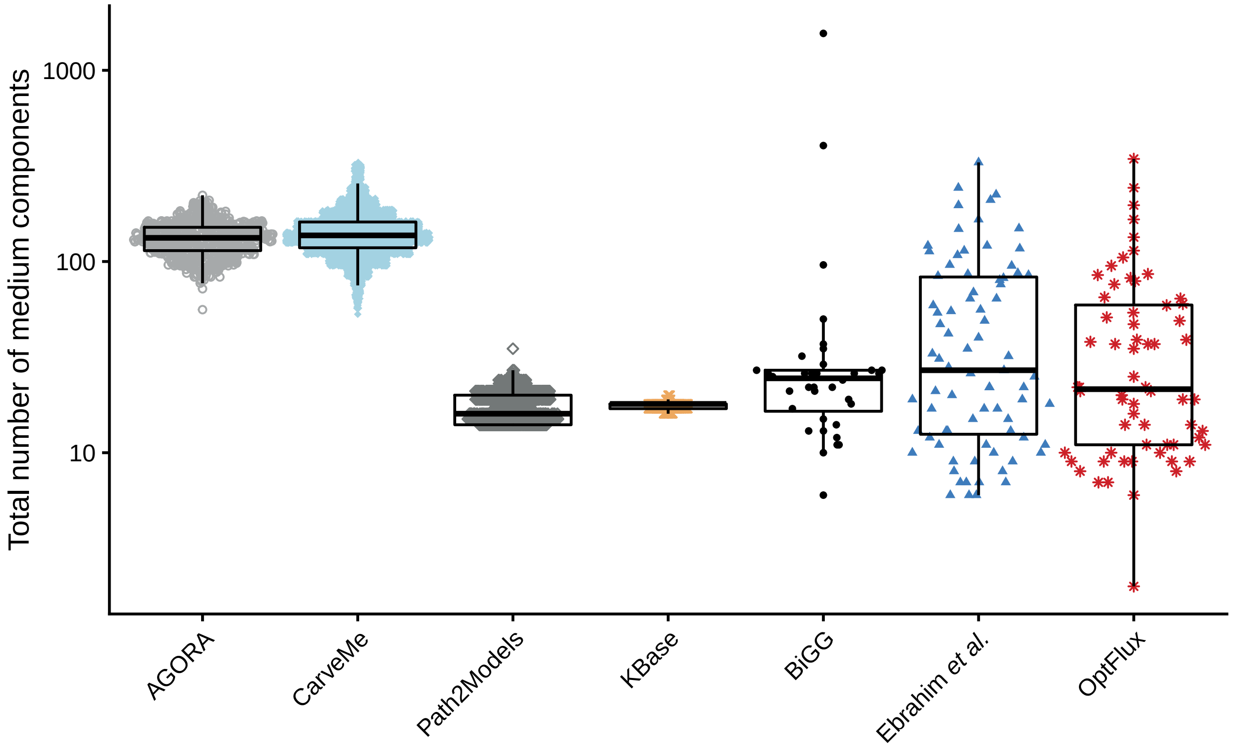

Figure 129: Number of Medium Components

7.3.4.4 Reaction Information

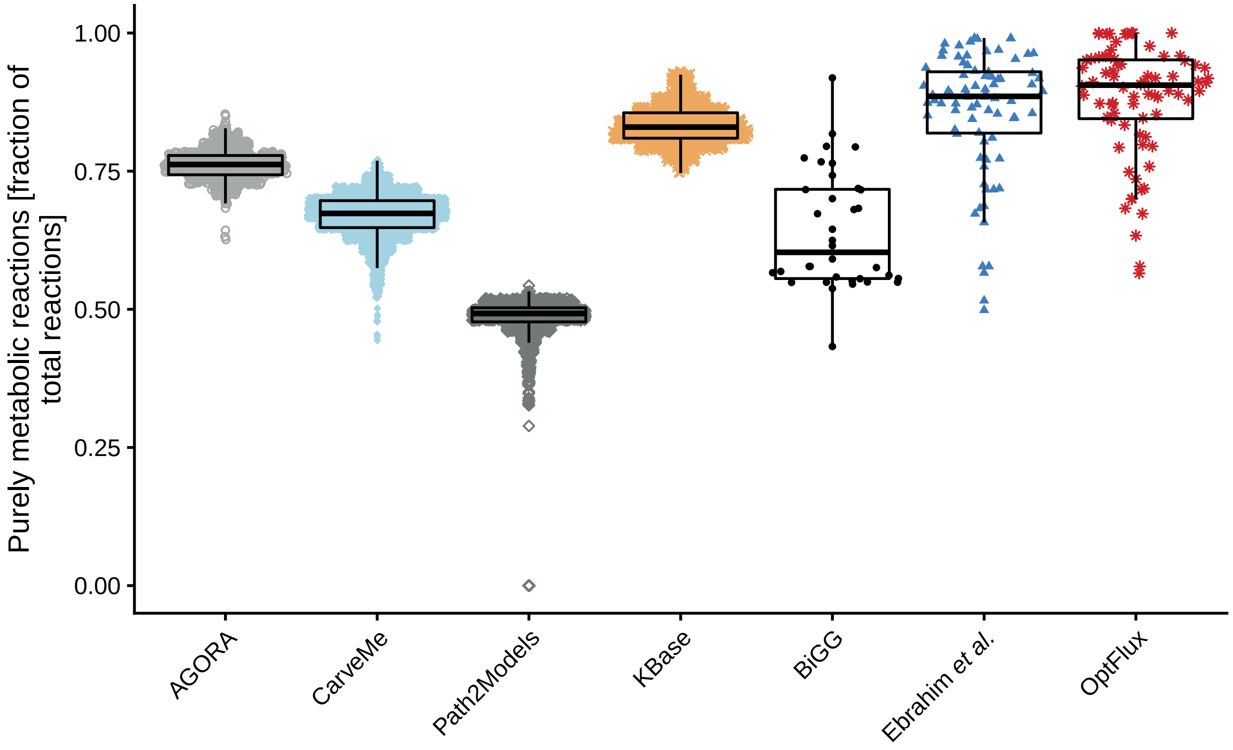

Figure 130: Purely Metabolic Reactions

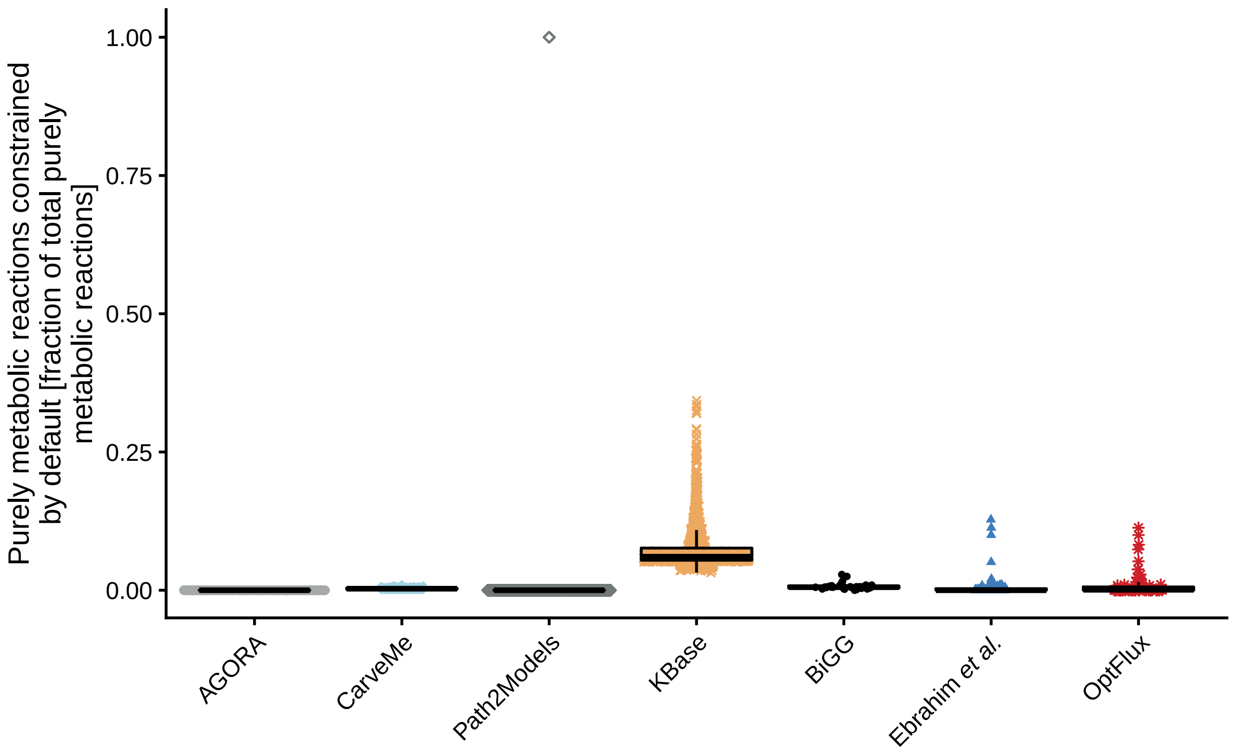

Figure 131: Purely Metabolic Reactions with Constraints

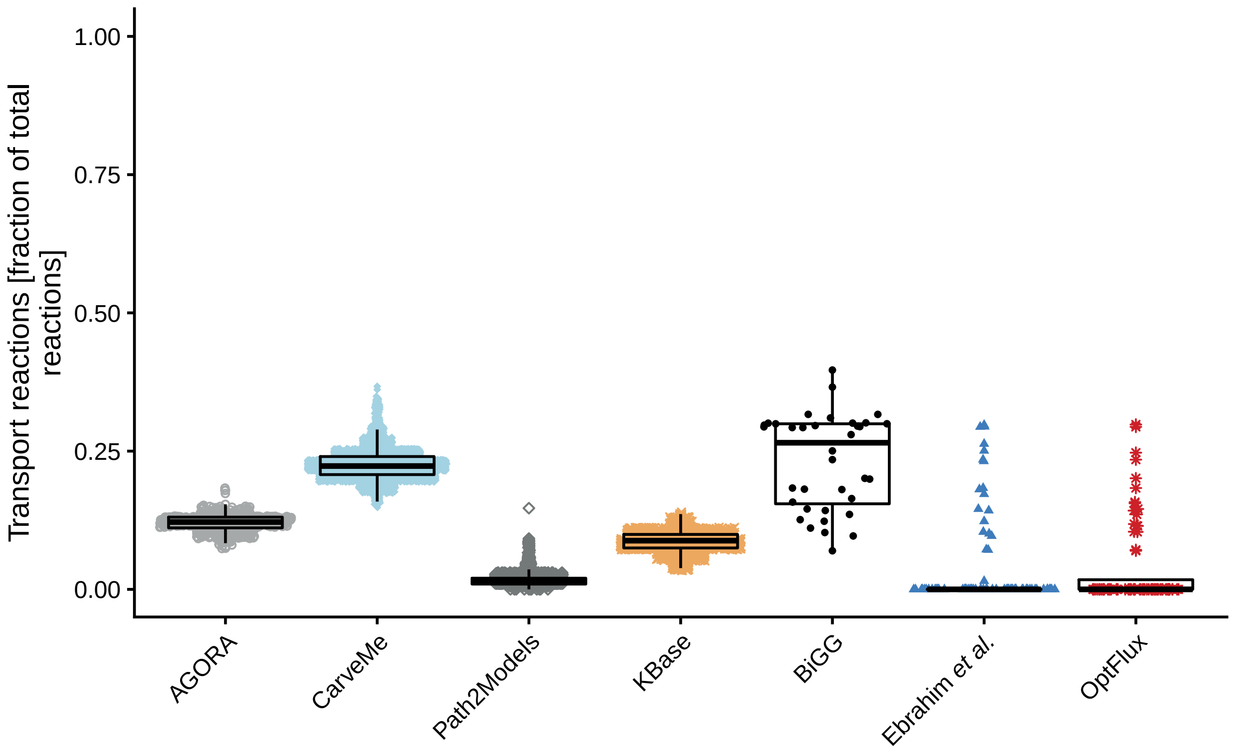

Figure 132: Transport Reactions

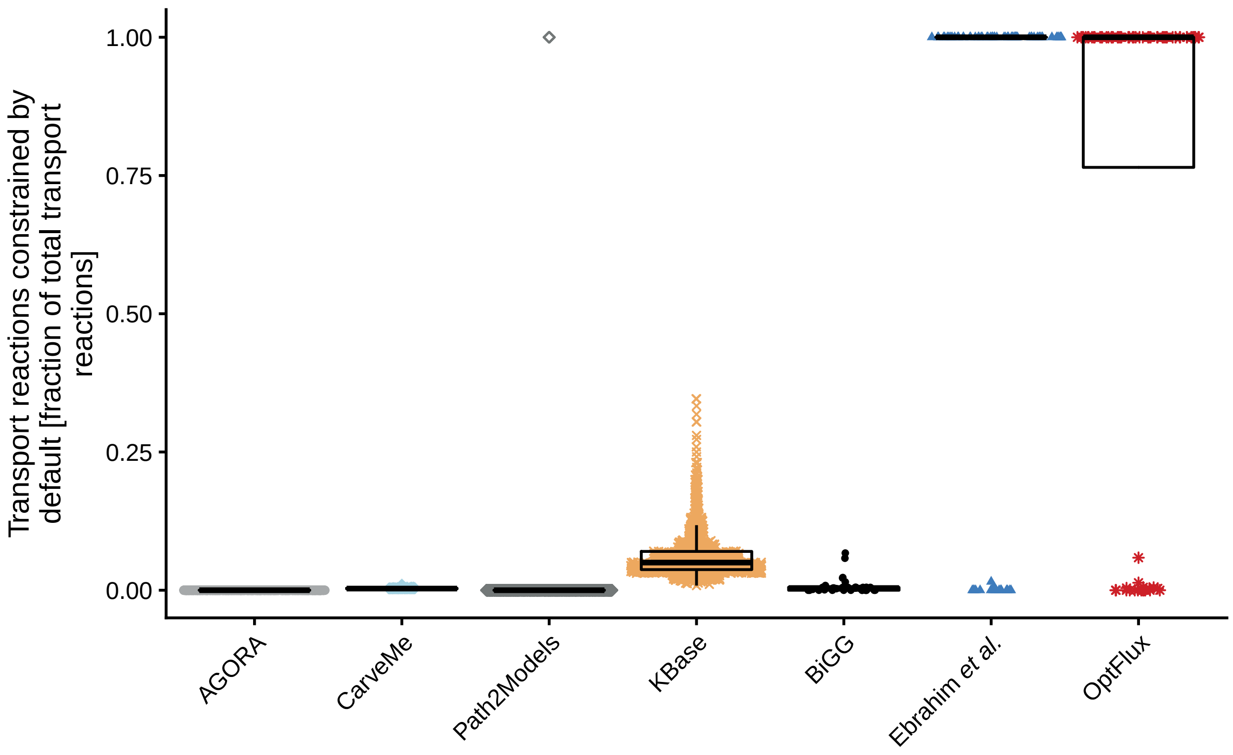

Figure 133: Transport Reactions with Constraints

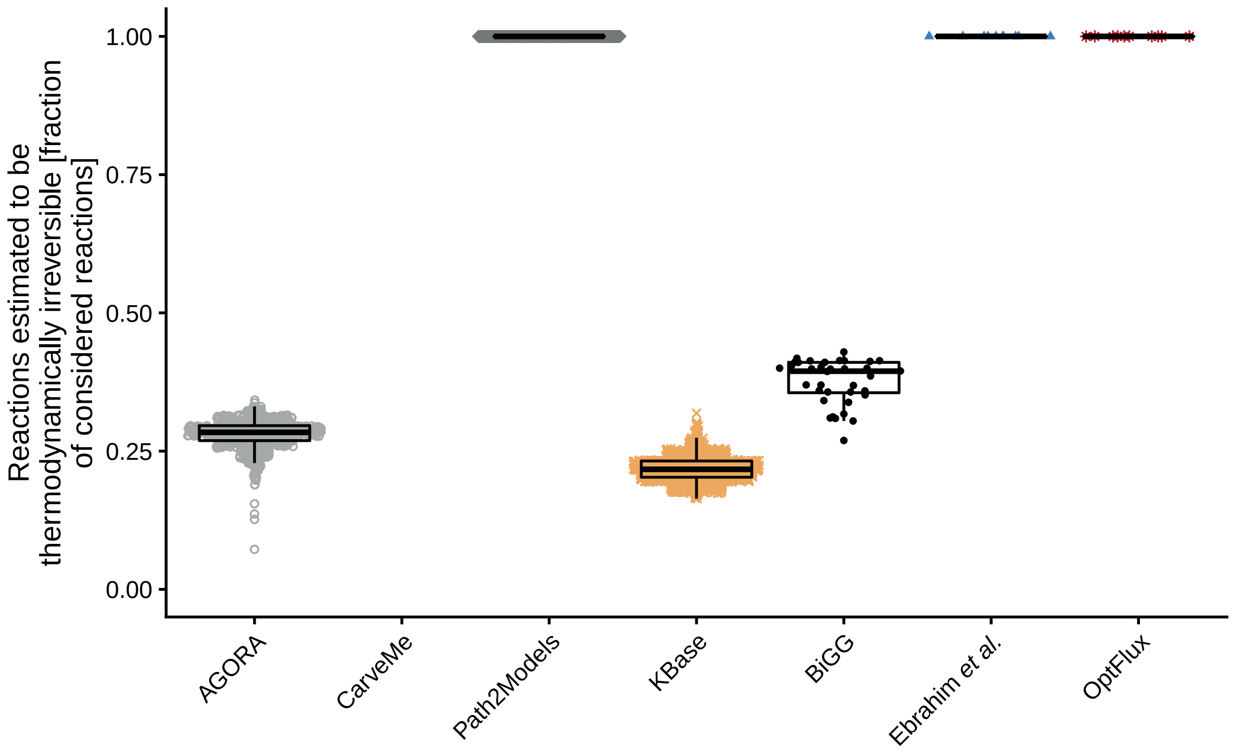

Figure 134: Thermodynamic Reversibility of Purely Metabolic Reactions

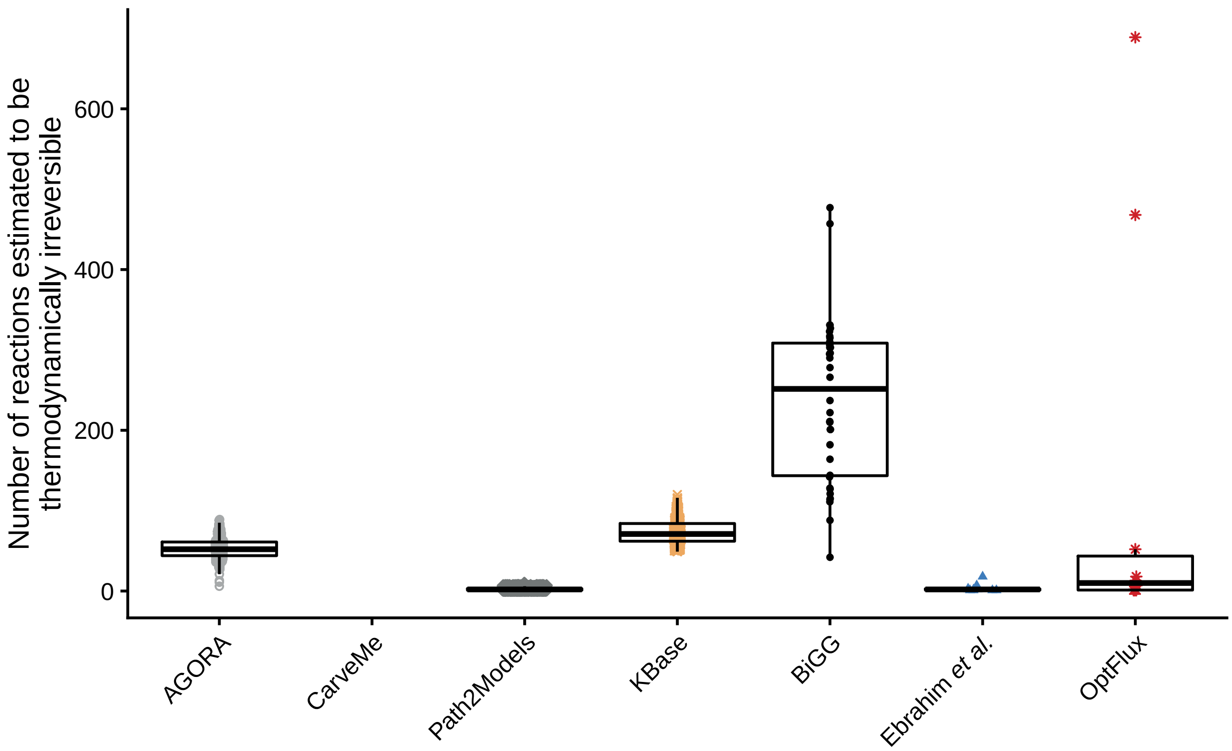

Figure 135: Thermodynamic Reversibility of Purely Metabolic Reactions

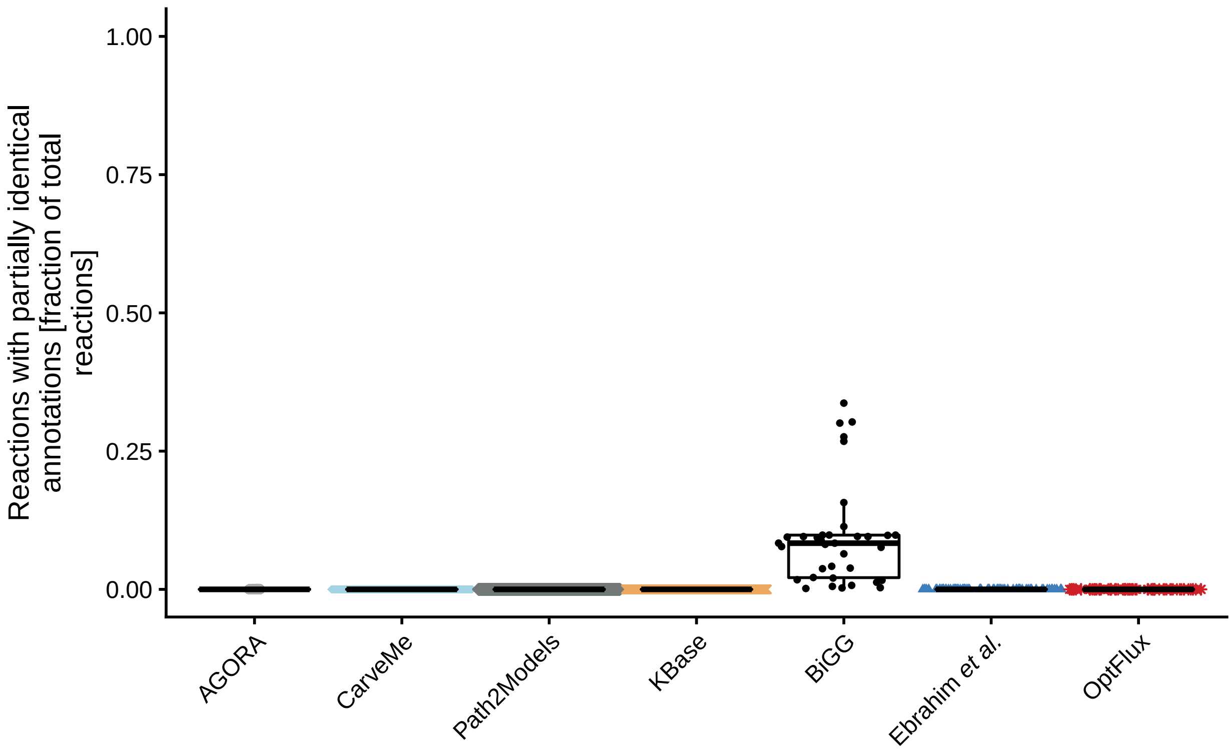

Figure 136: Reactions with Partially Identical Annotations

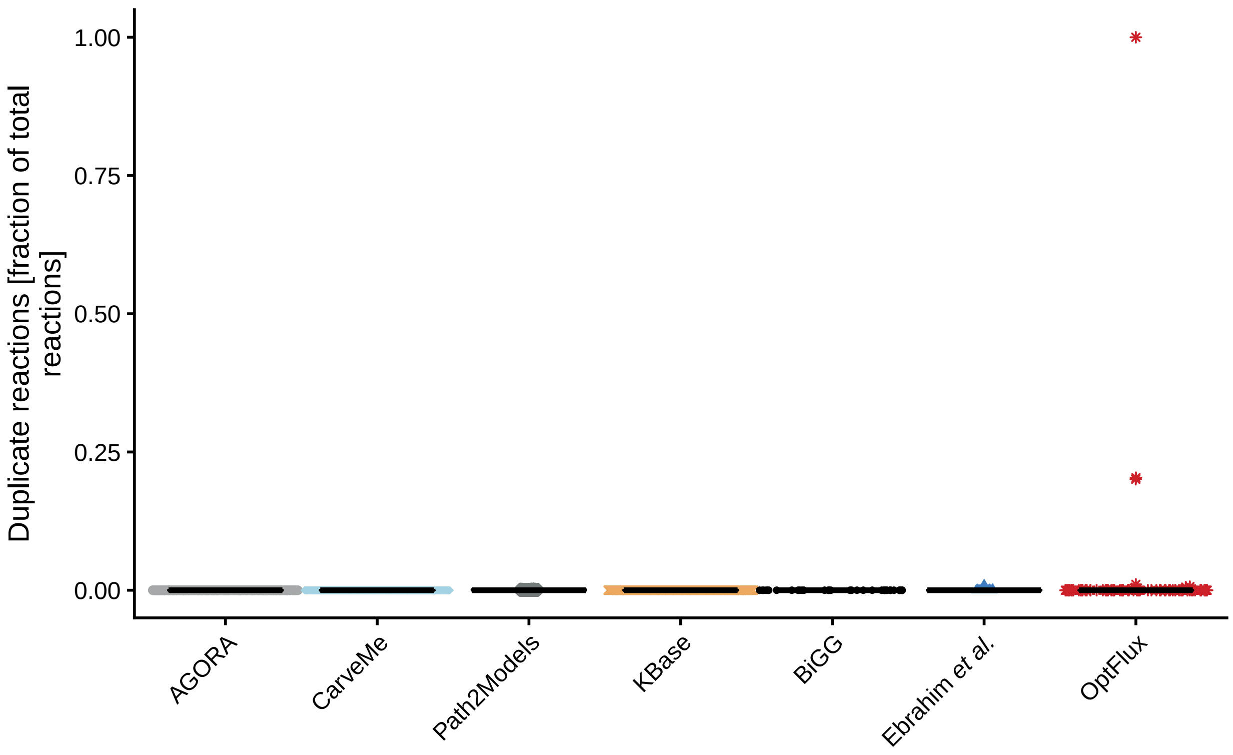

Figure 137: Duplicate Reactions

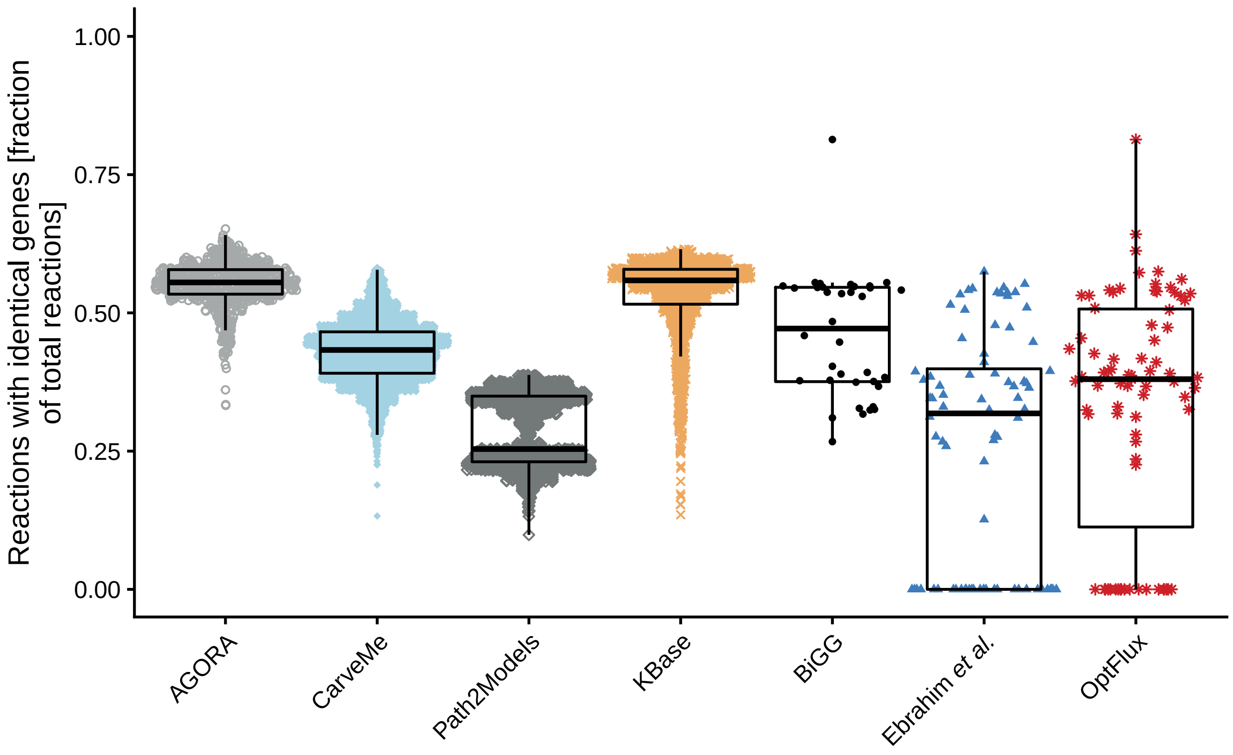

Figure 138: Reactions with Identical Genes

7.3.4.5 Gene-Protein-Reaction (GPR) Association

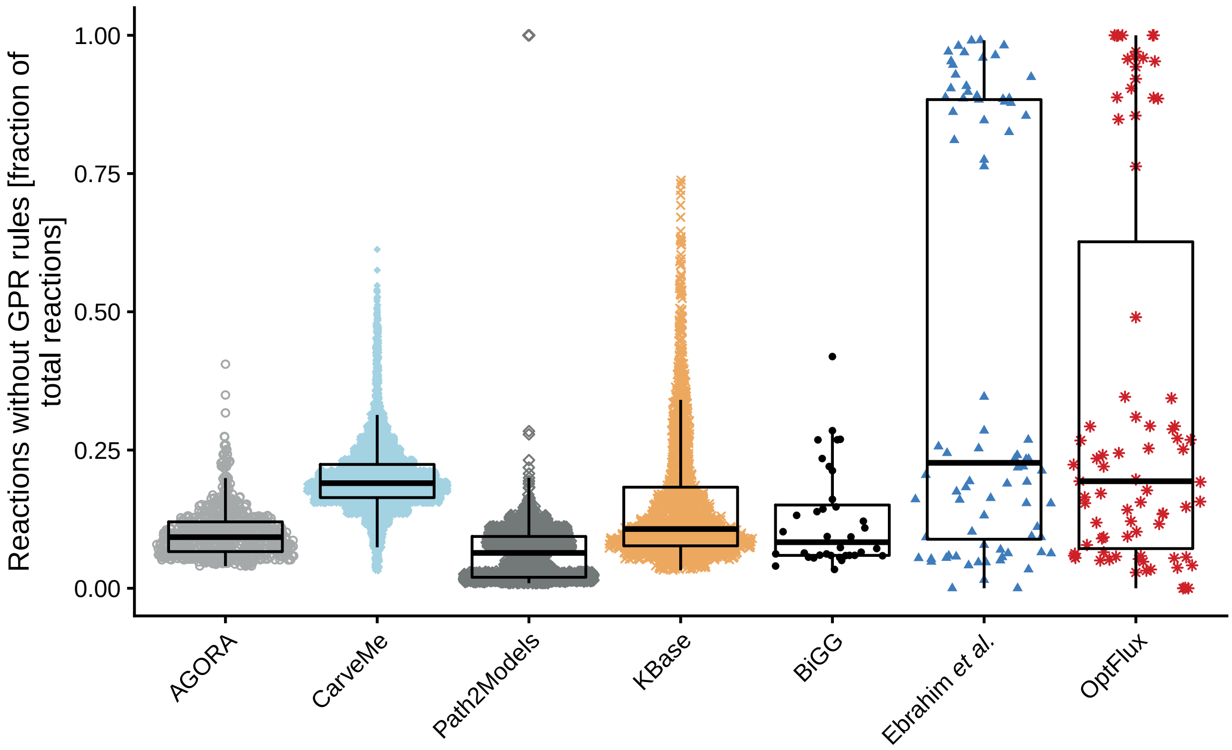

Figure 139: Reactions without GPR

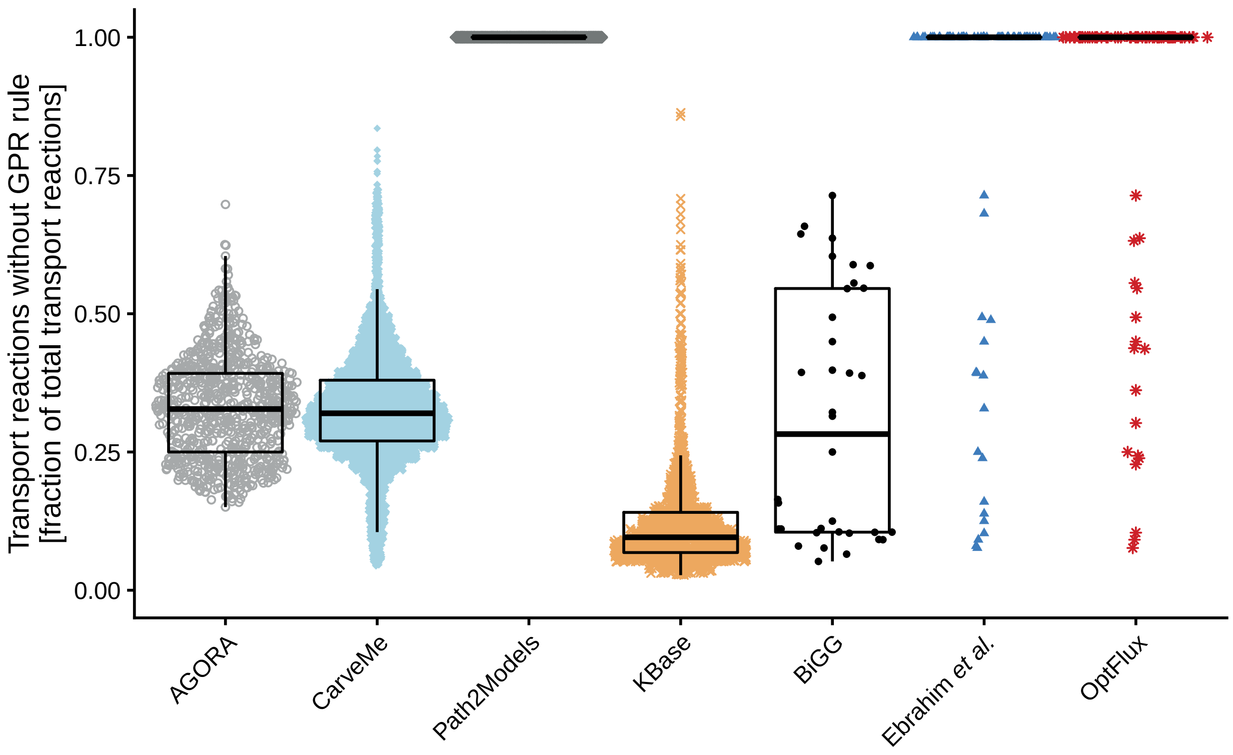

Figure 140: Fraction of Transport Reactions without GPR

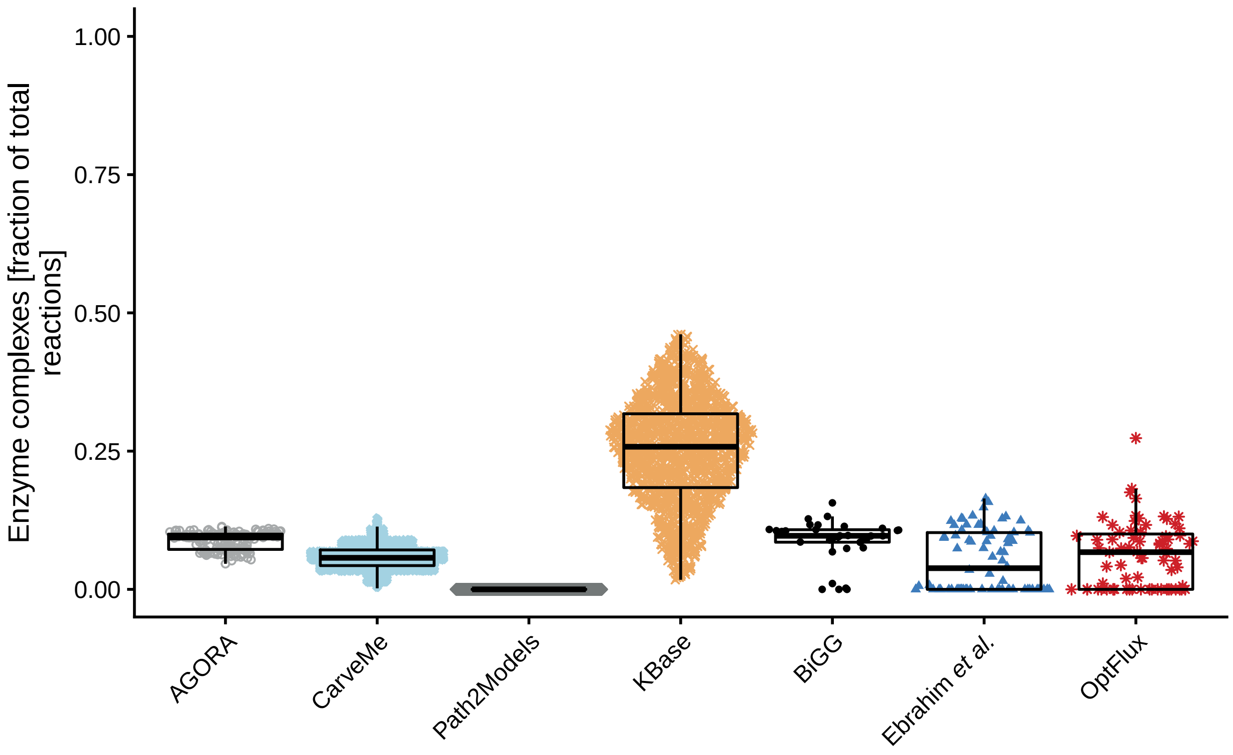

Figure 141: Enzyme Complexes

7.3.4.6 Biomass

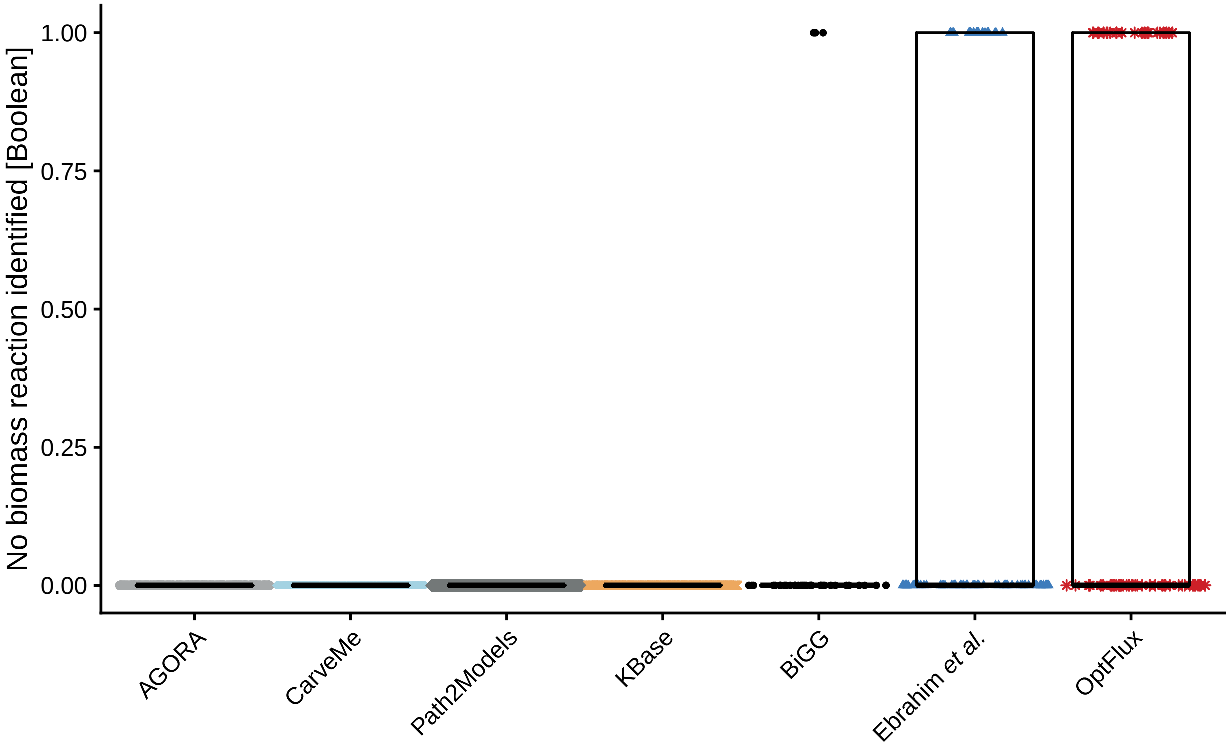

Figure 142: Biomass Reactions Identified

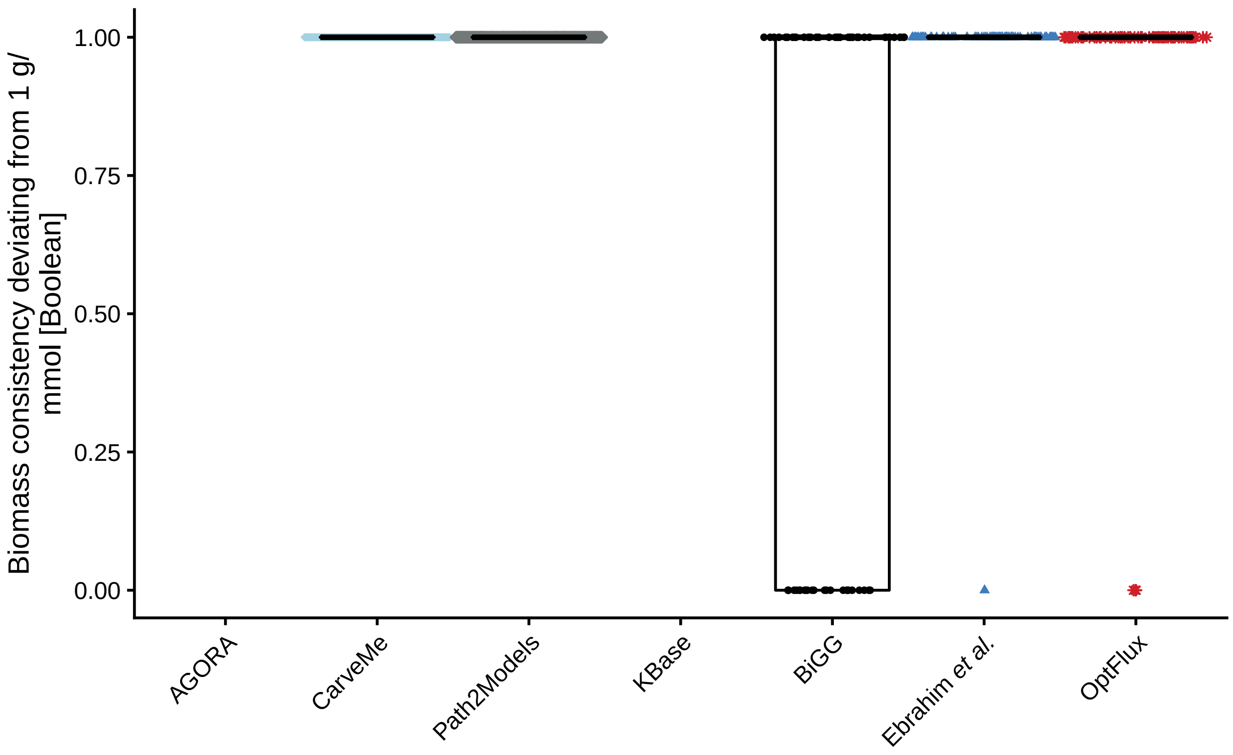

Figure 143: Biomass Consistency

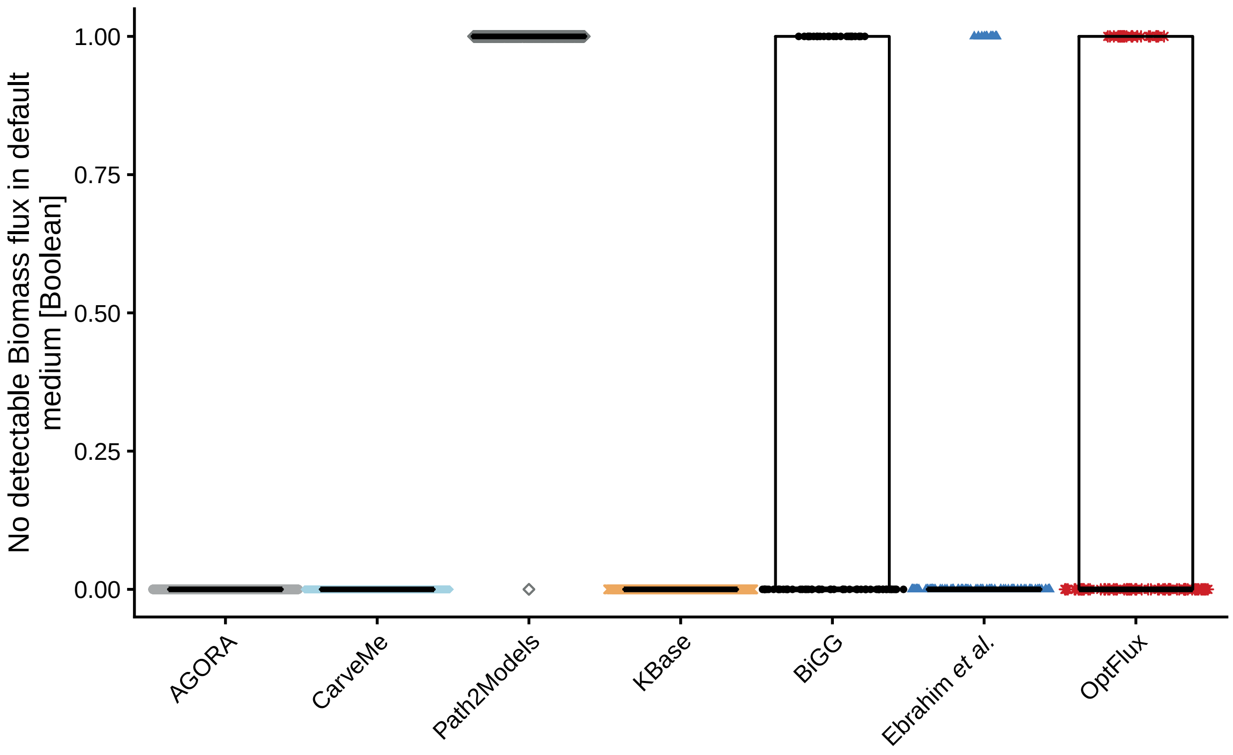

Figure 144: Biomass Production in Default Medium

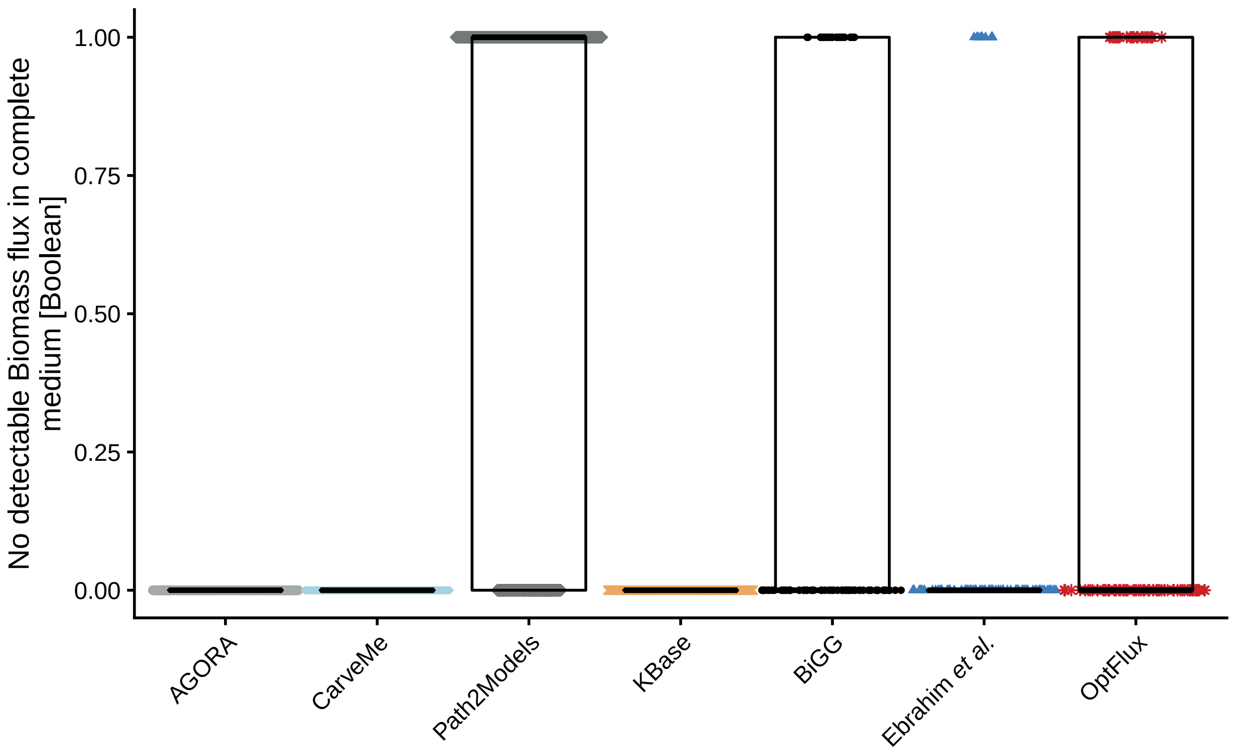

Figure 145: Biomass Production in Complete Medium

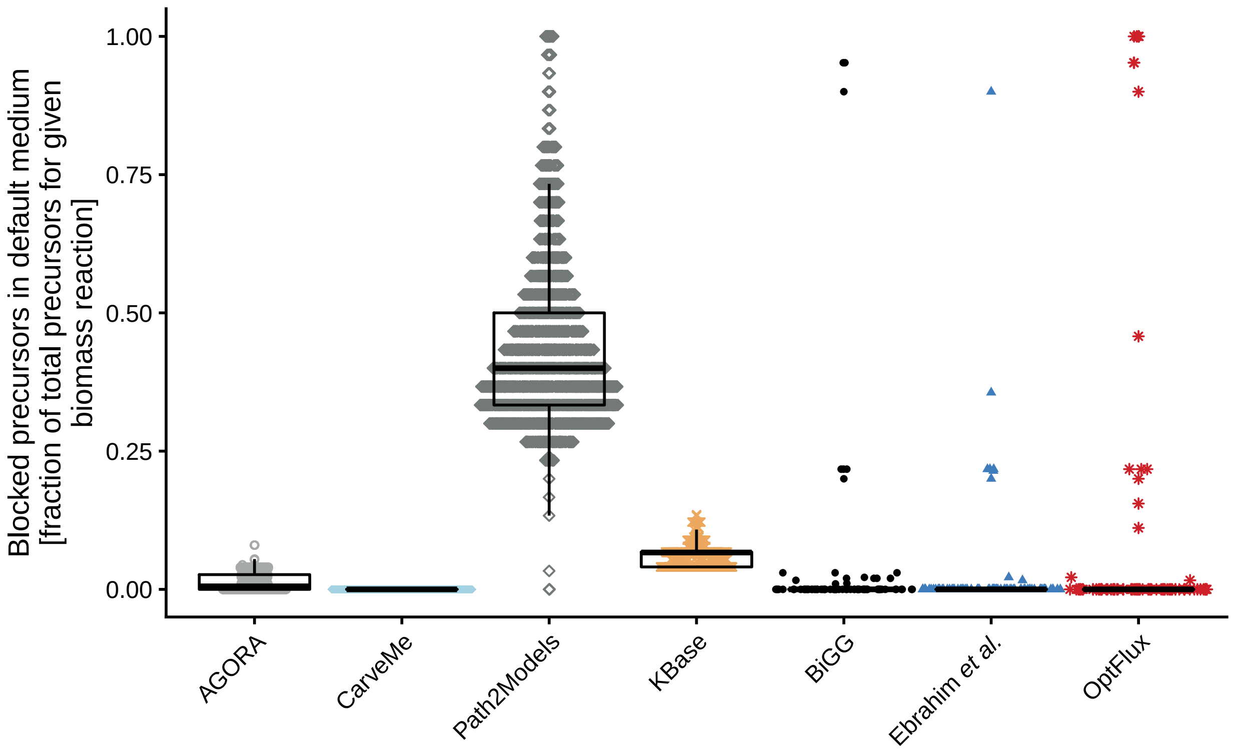

Figure 146: Blocked Biomass Precursors in Default Medium

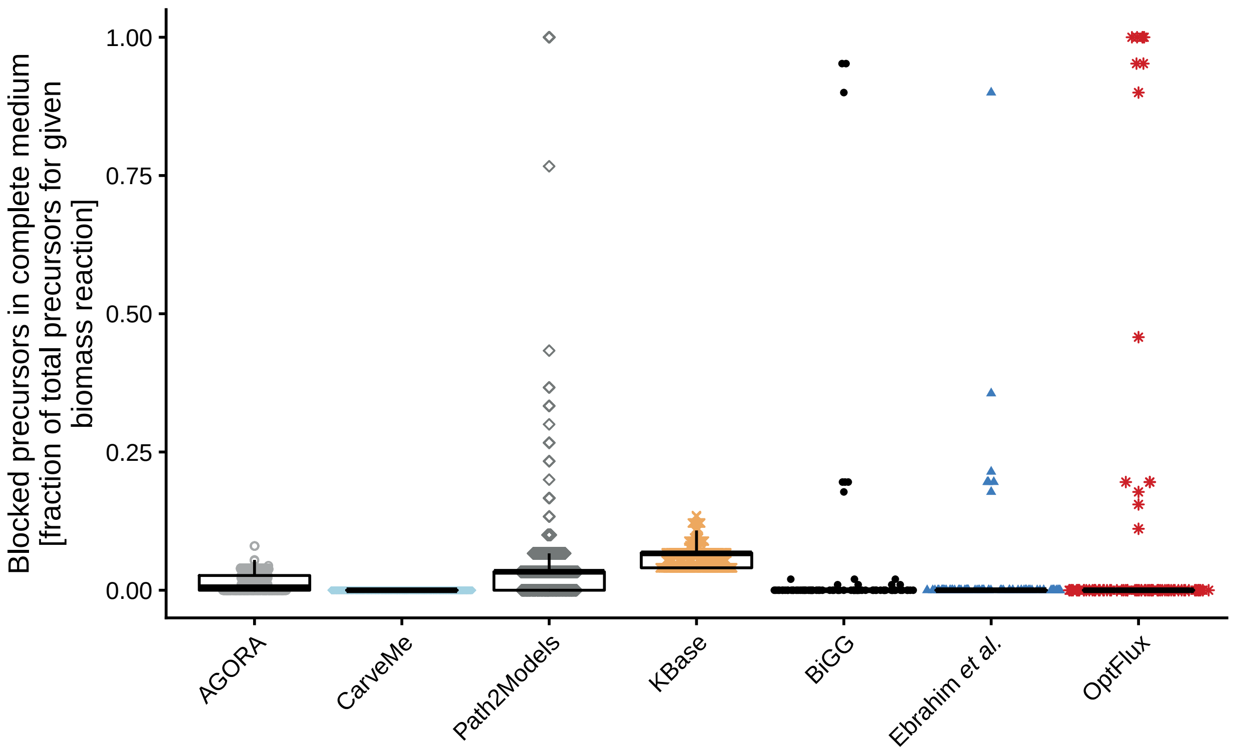

Figure 147: Blocked Biomass Precursors in Complete Medium

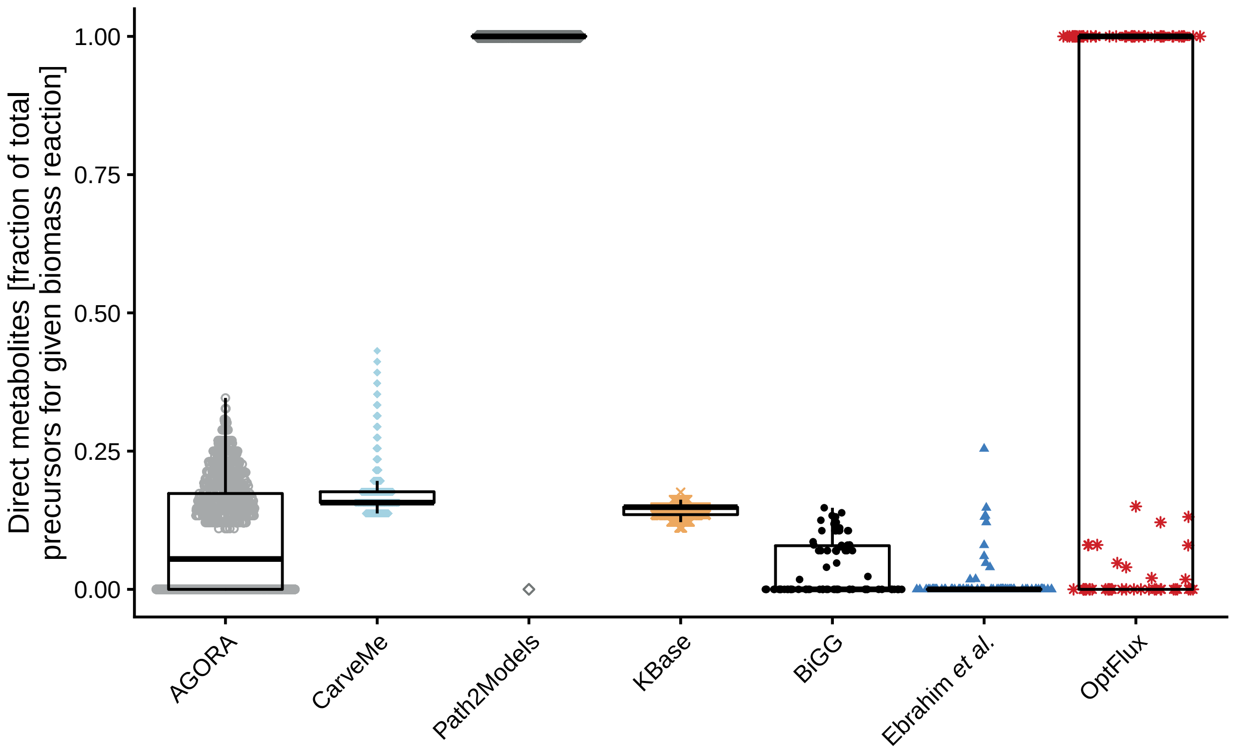

Figure 148: Ratio of Direct Metabolites in Biomass Reaction

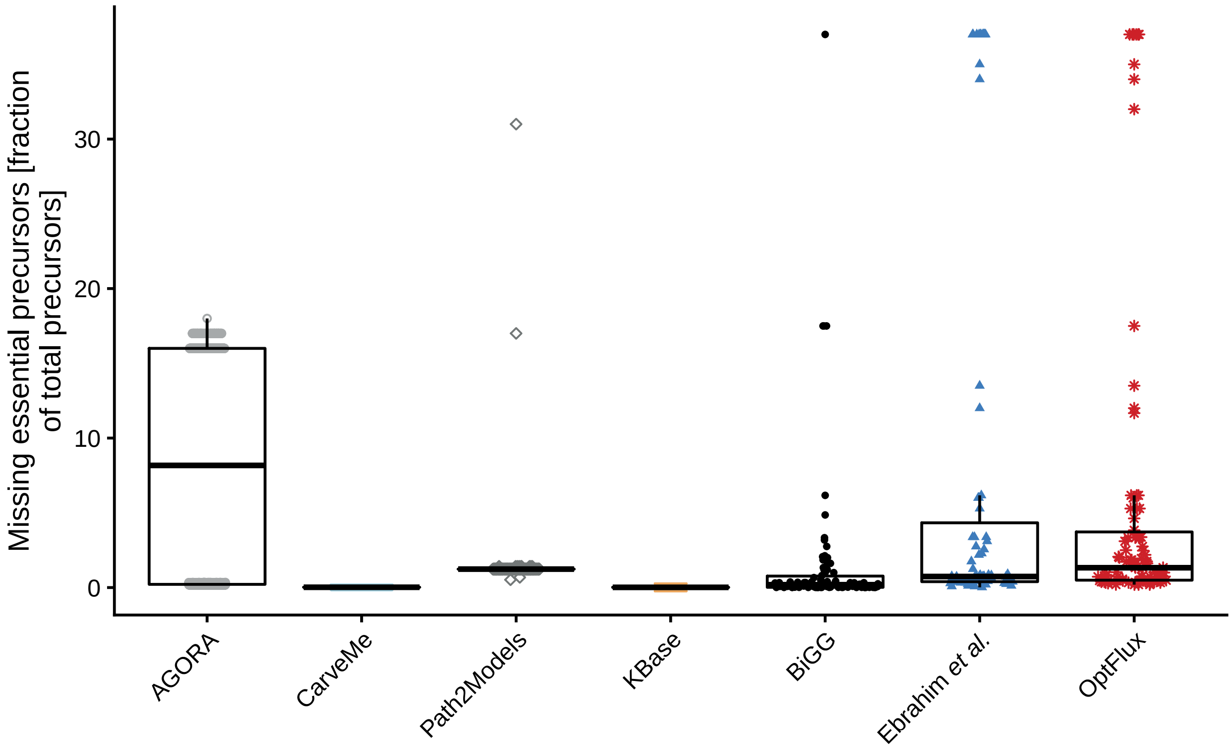

Figure 149: Number of Missing Essential Biomass Precursors

7.3.4.7 Energy Metabolism

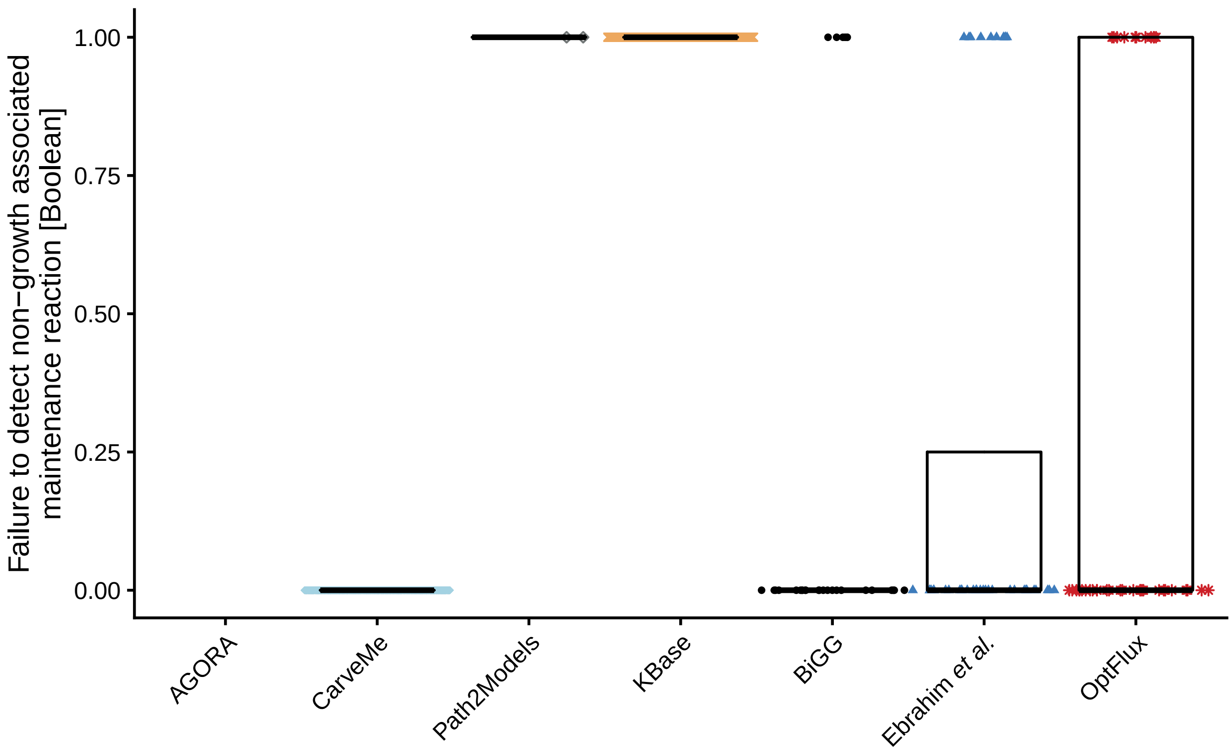

Figure 150: Non-Growth Associated Maintenance Reaction

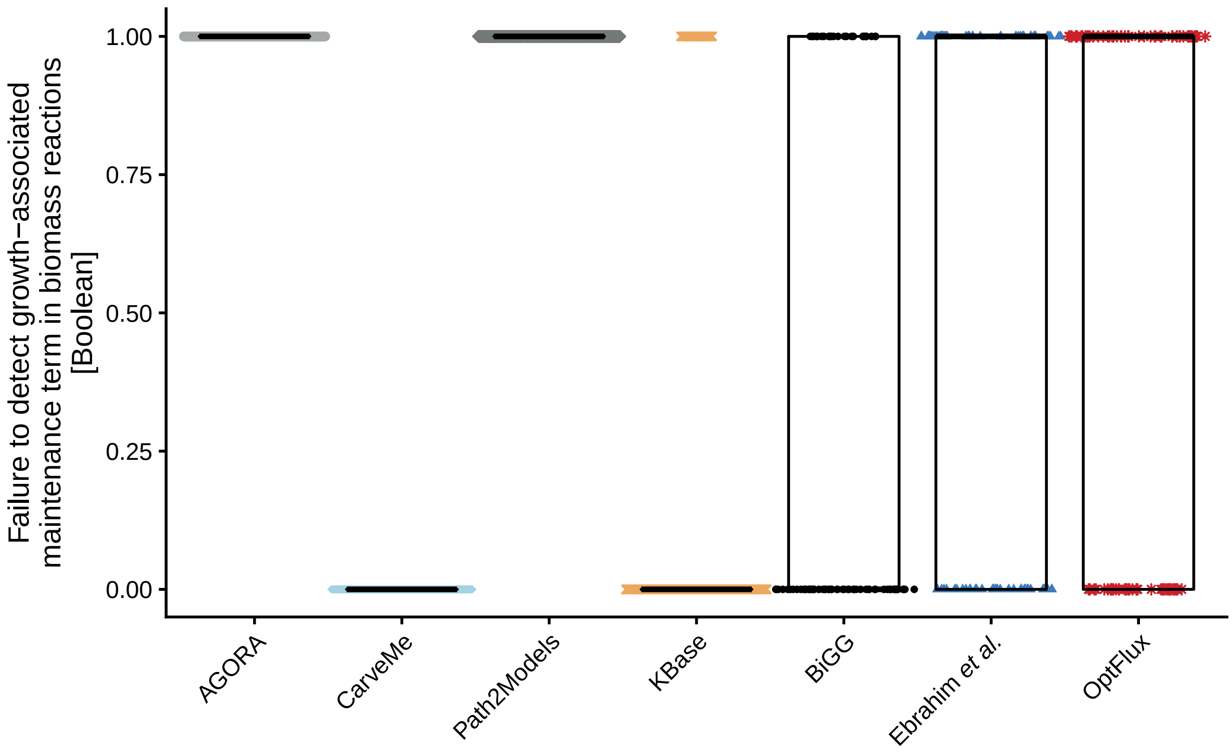

Figure 151: Growth-associated Maintenance in Biomass Reaction

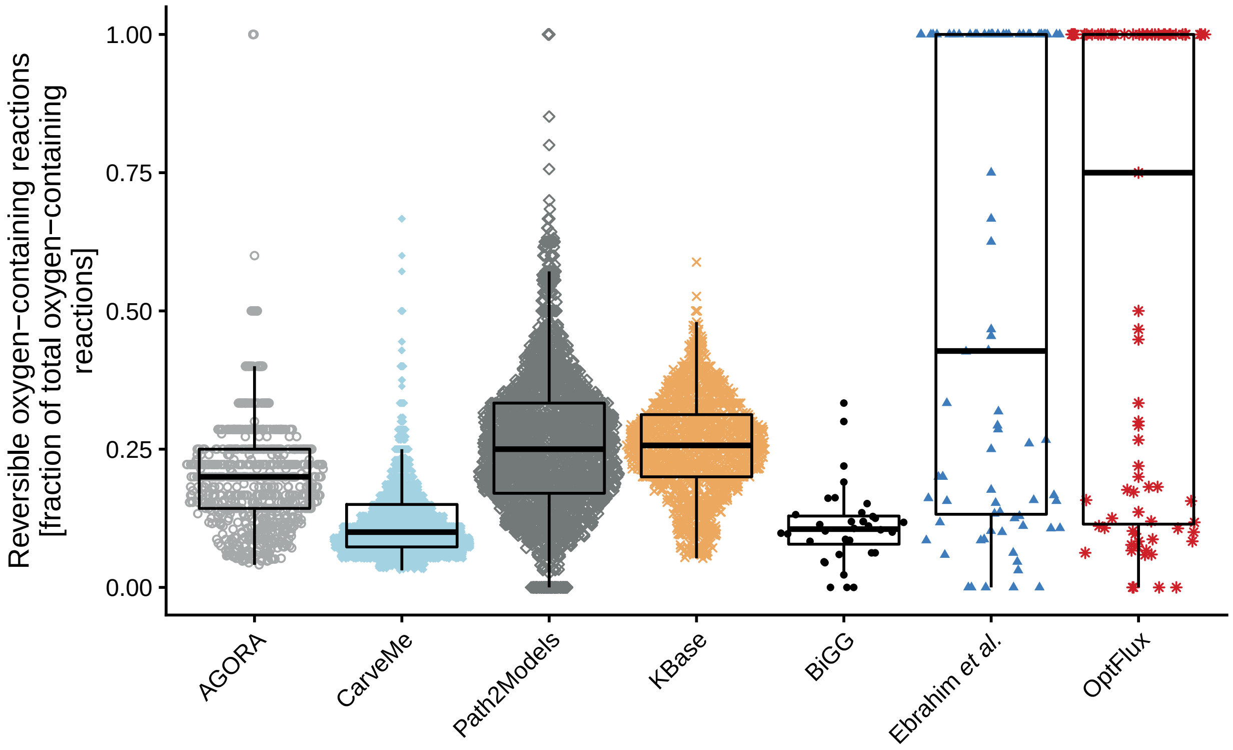

Figure 152: Number of Reversible Oxygen-Containing Reactions

7.3.4.8 Network Topology

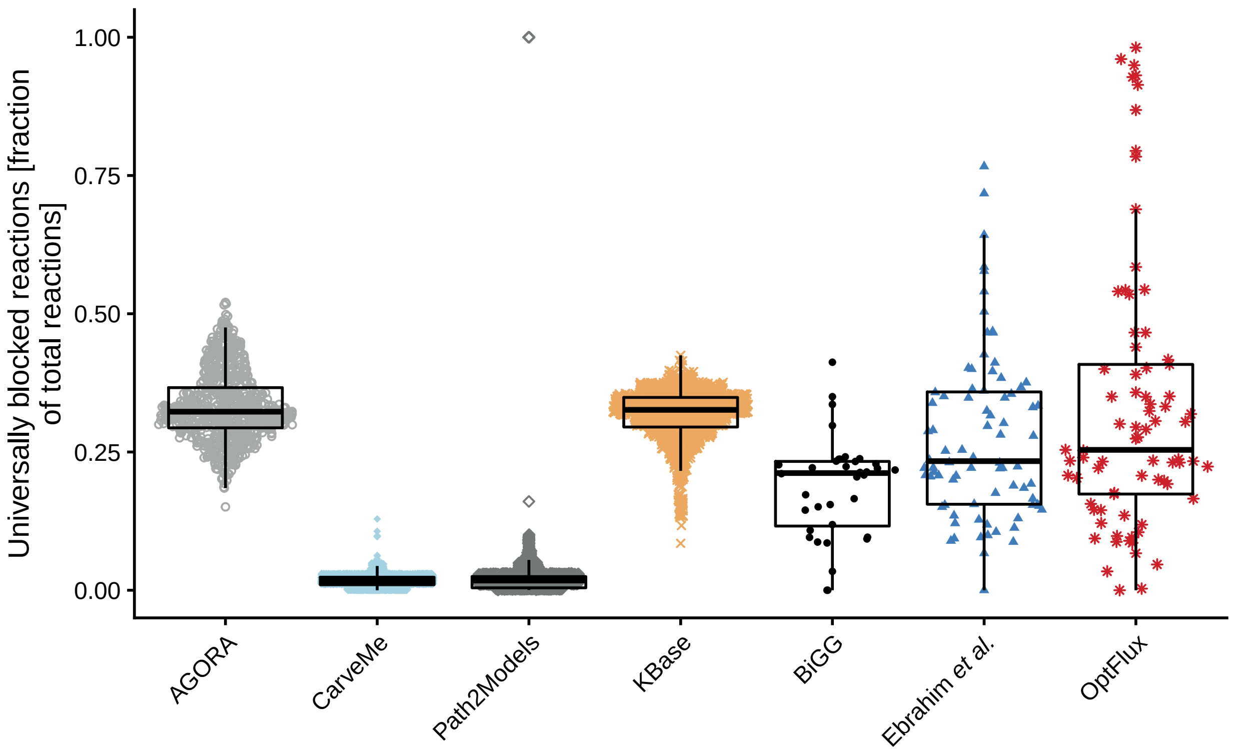

Figure 153: Universally Blocked Reactions

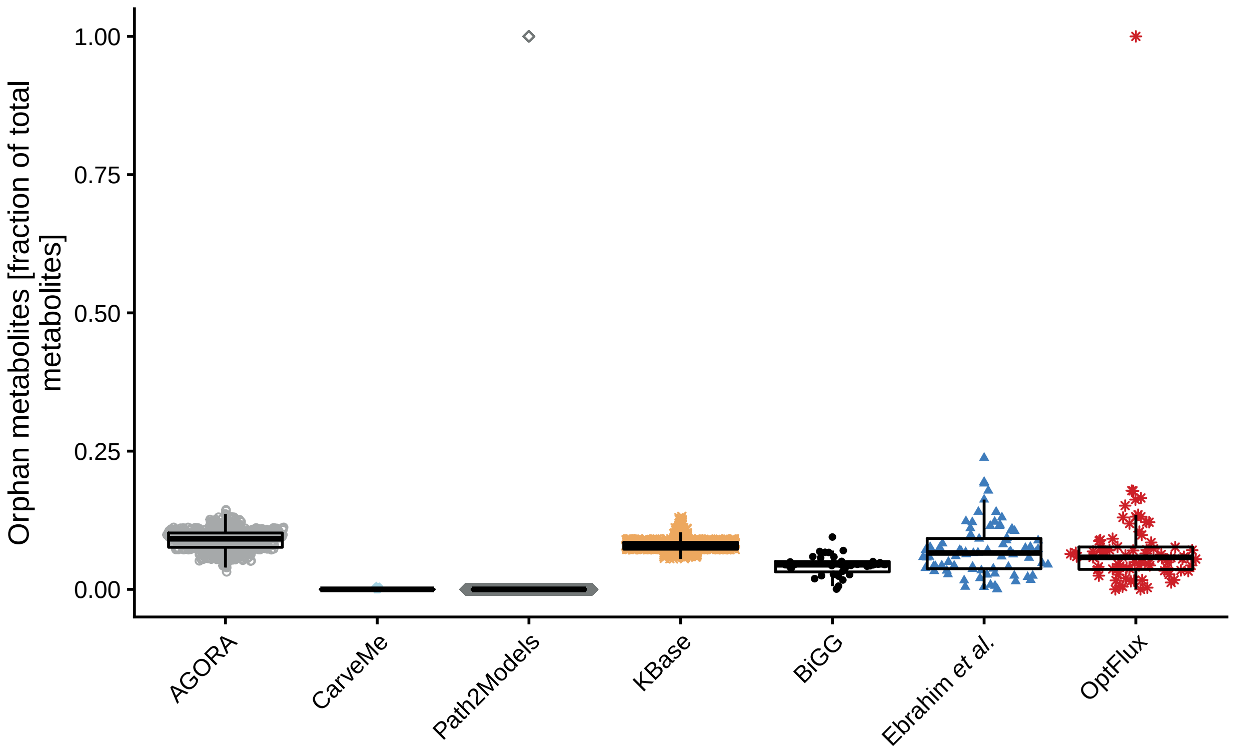

Figure 154: Orphan Metabolites

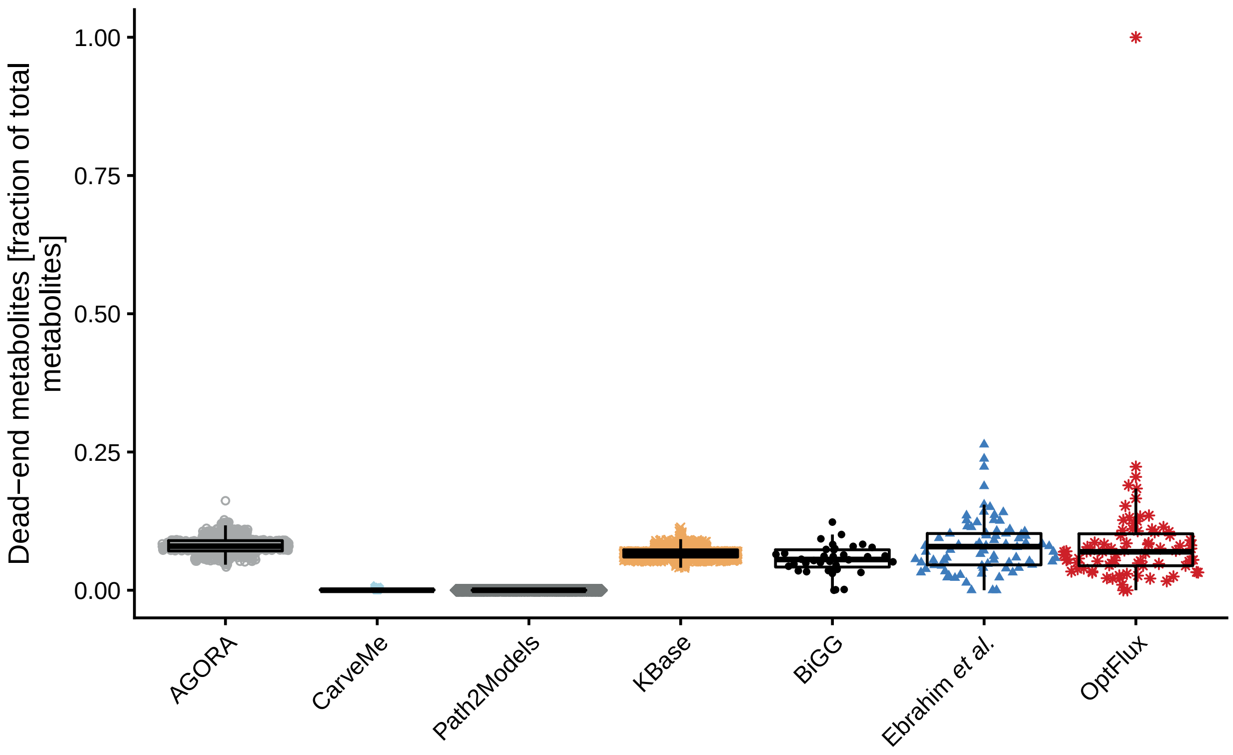

Figure 155: Dead-end Metabolites

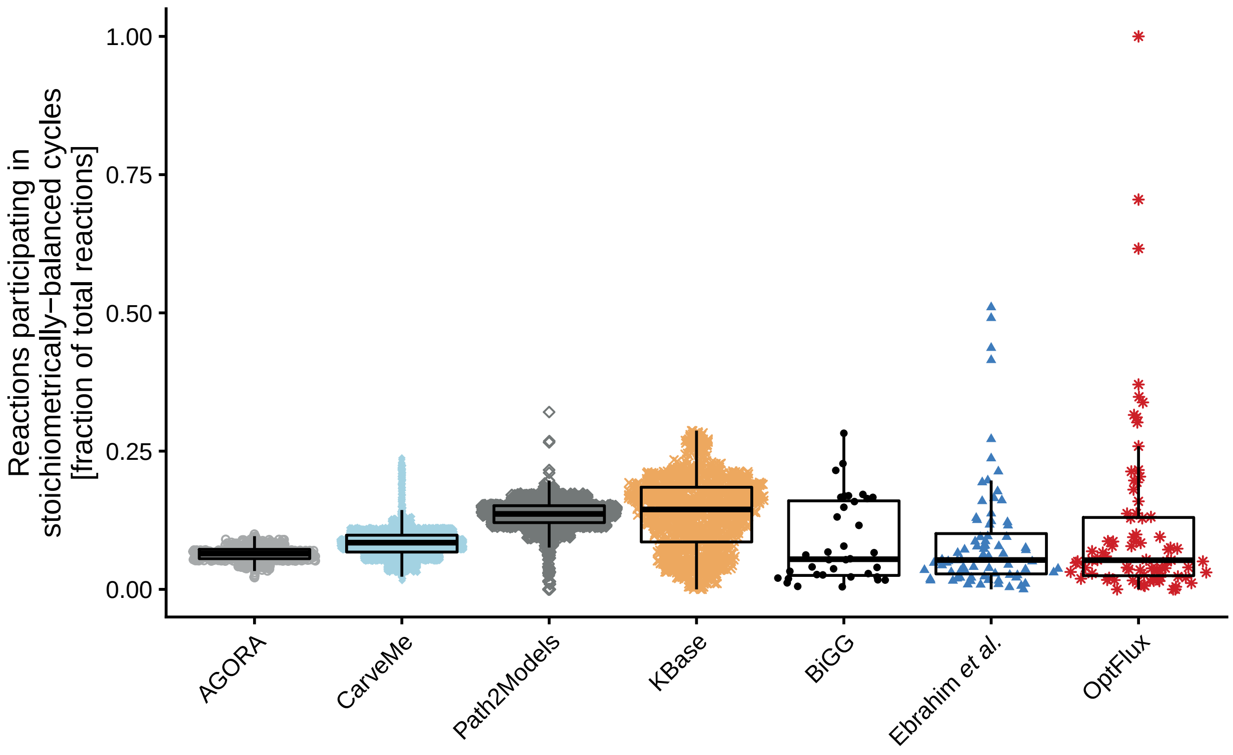

Figure 156: Stoichiometrically Balanced Cycles

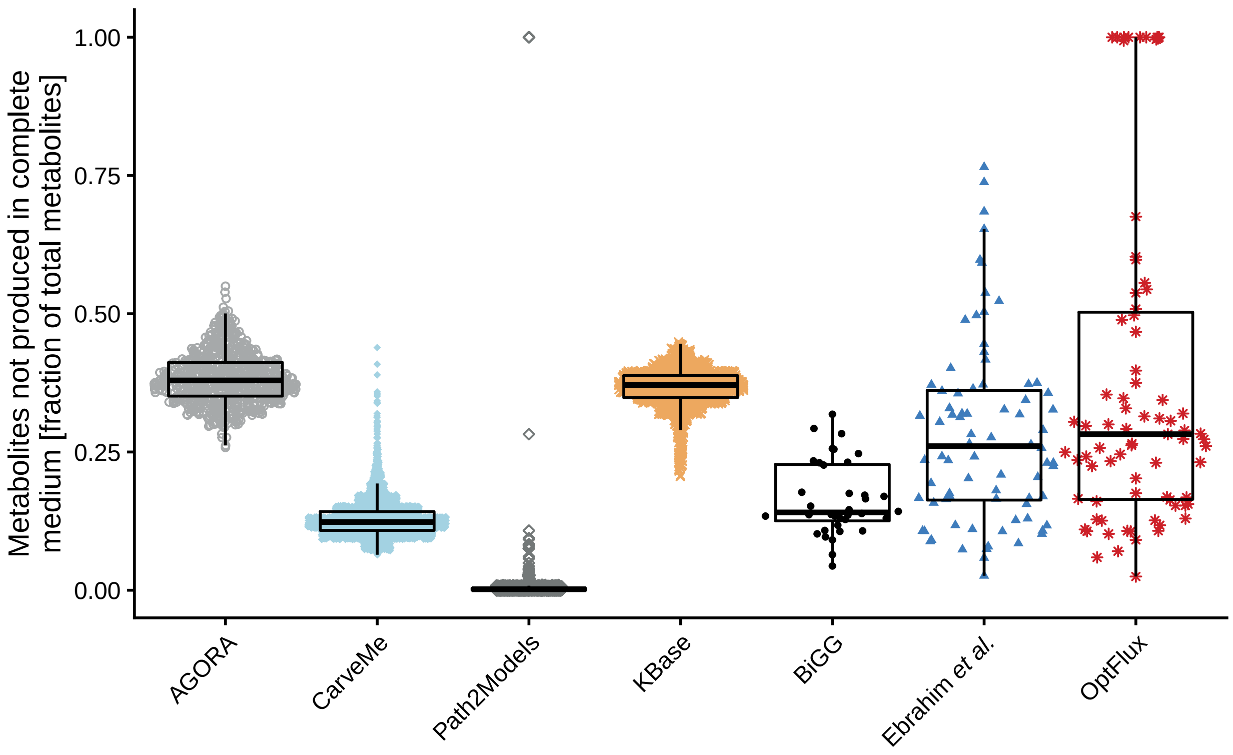

Figure 157: Metabolite Production in Complete Medium

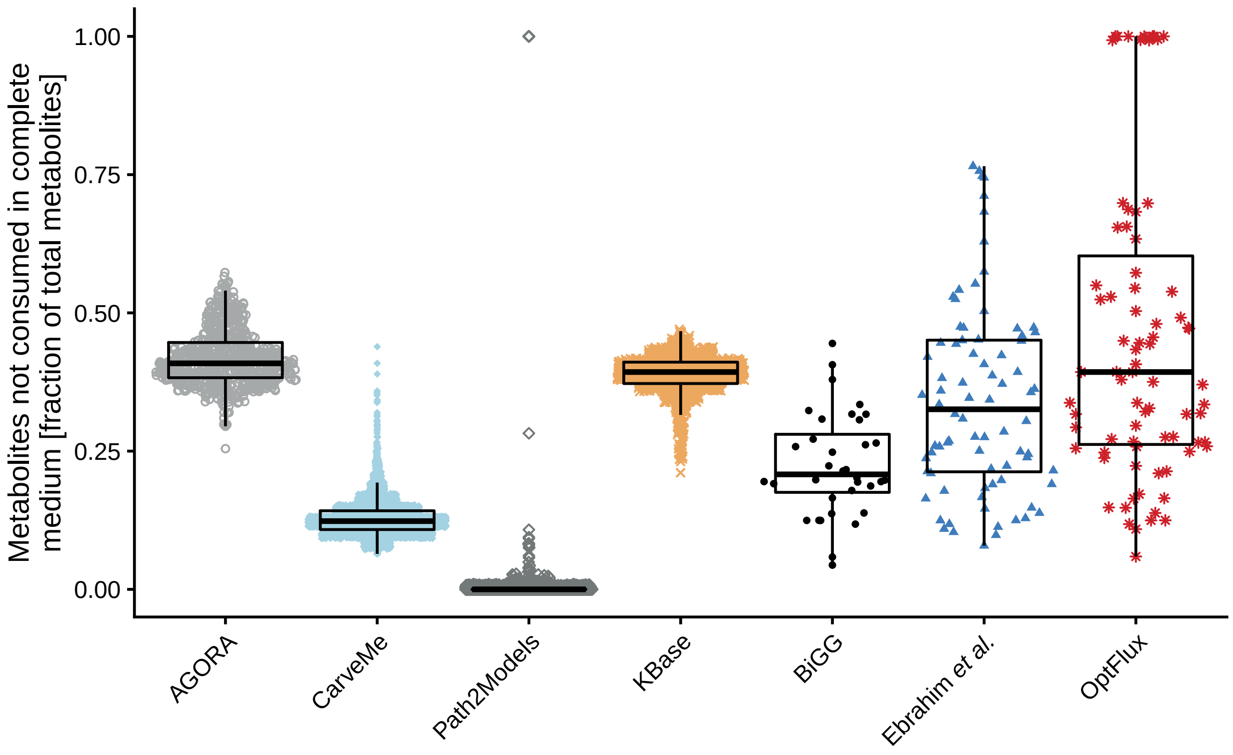

Figure 158: Metabolite Consumption in Complete Medium

7.3.4.9 Matrix Conditioning

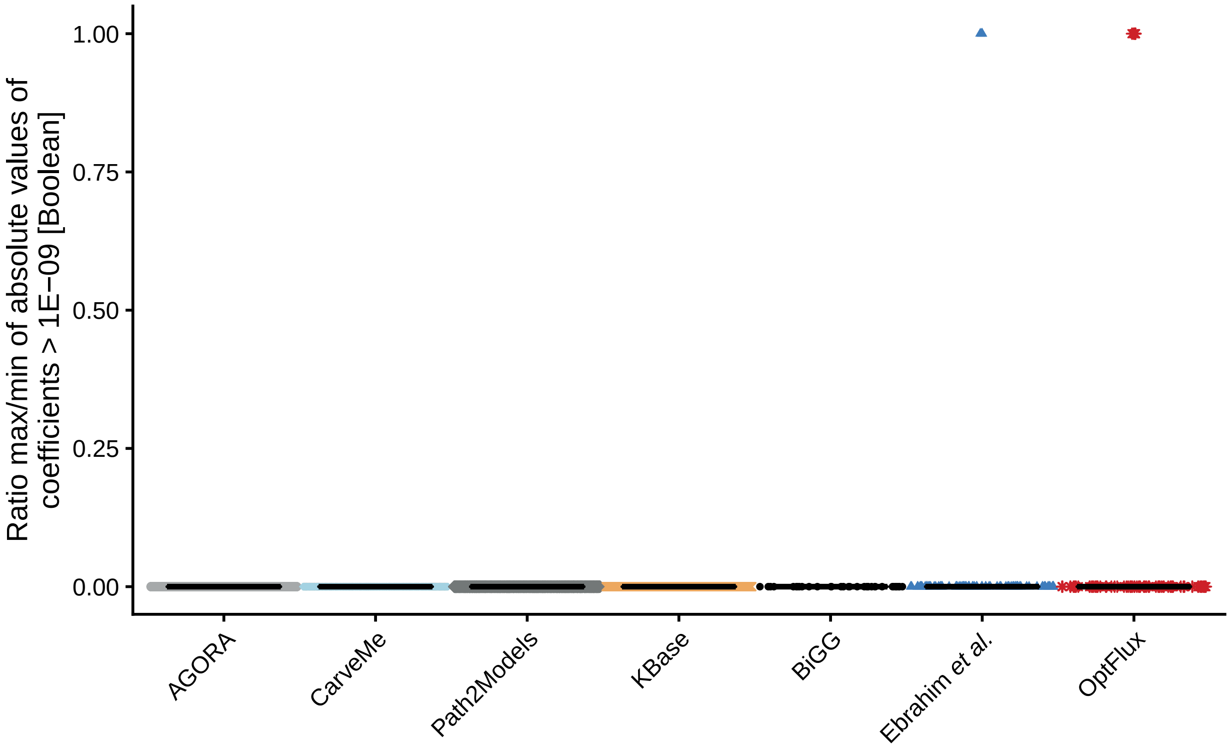

Figure 159: Ratio Min/Max Non-Zero Coefficients

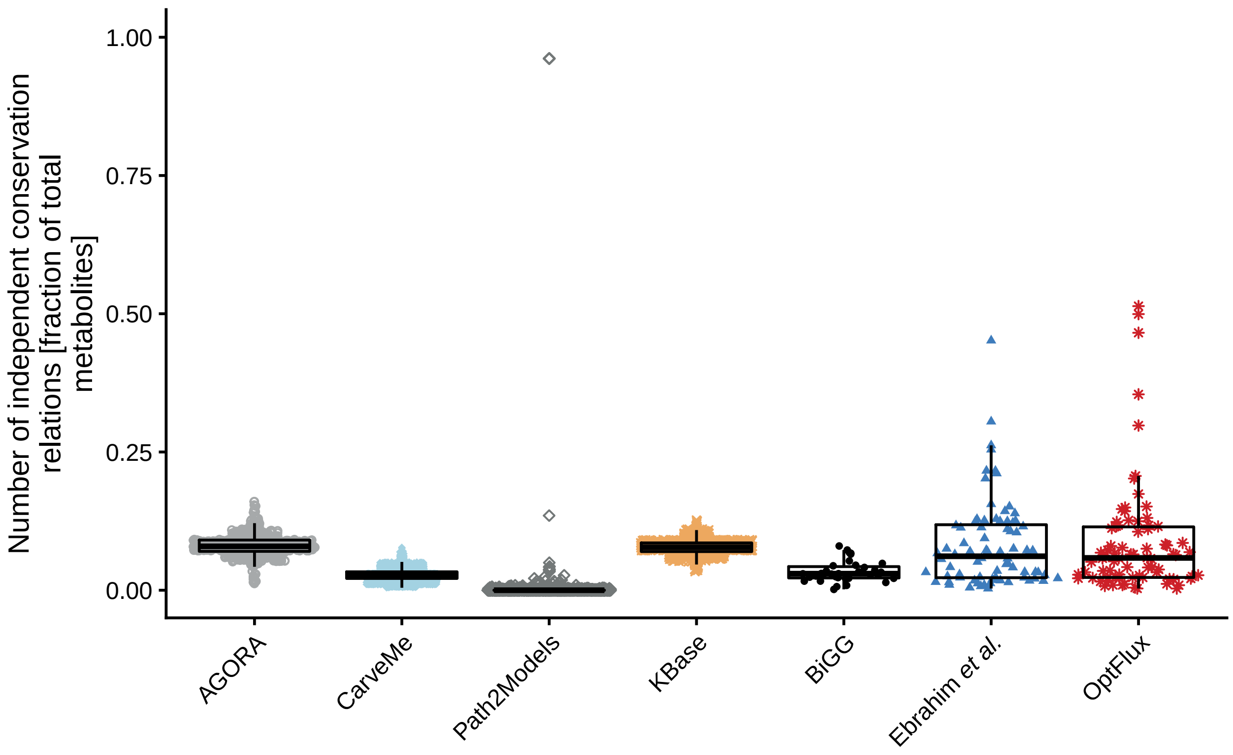

Figure 160: Independent Conservation Relations

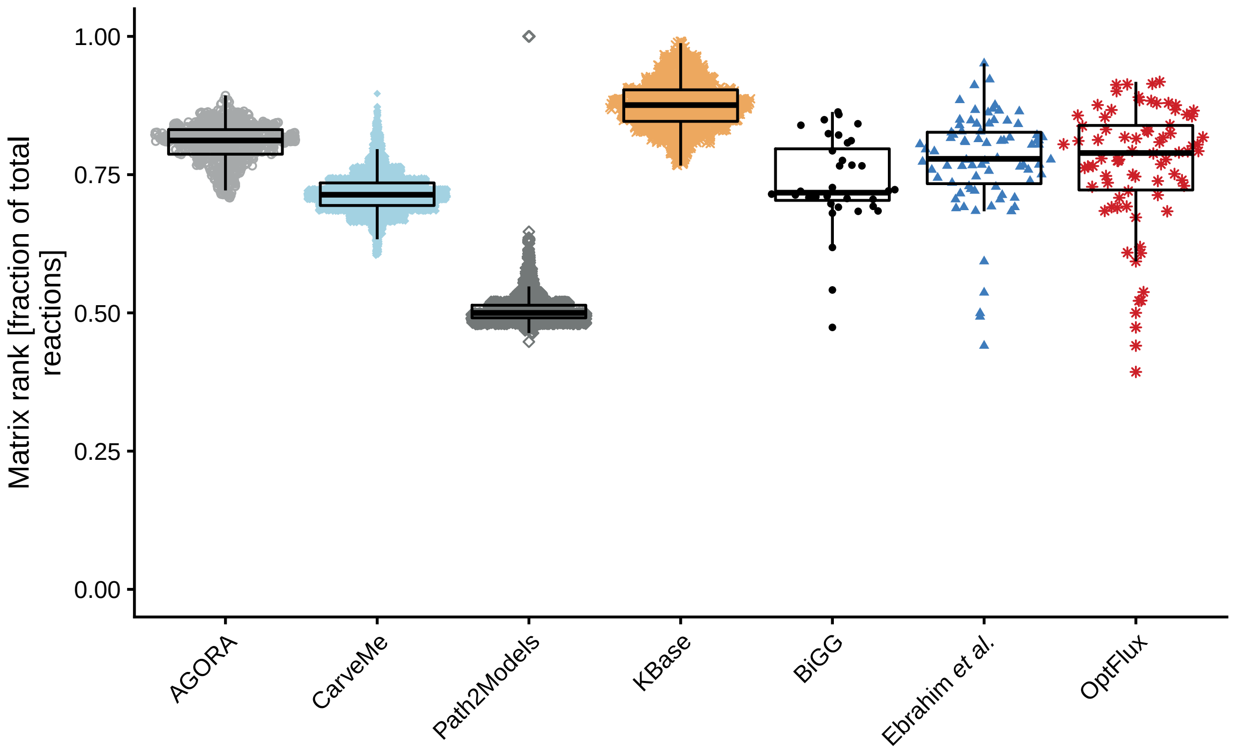

Figure 161: Rank

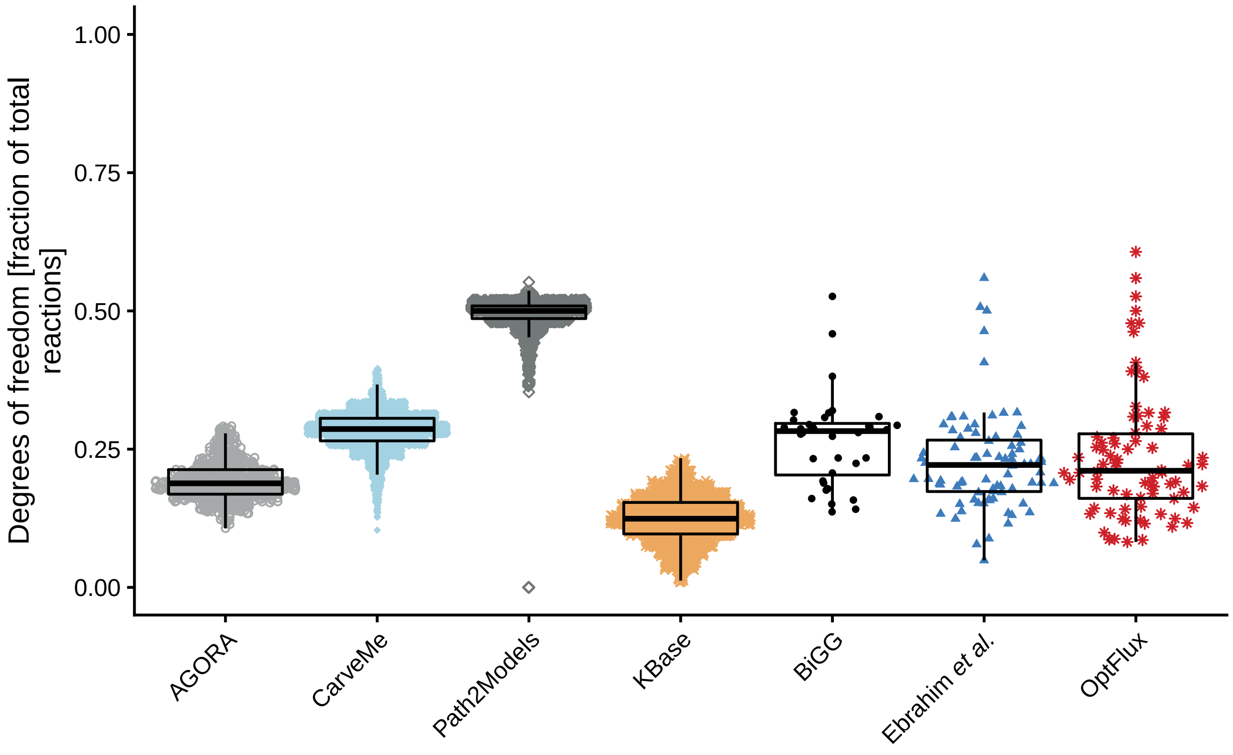

Figure 162: Degrees of Freedom

8 Author Contributions

Christian Lieven, Moritz E. Beber and Nikolaus Sonnenschein conceived the study. Moritz E. Beber, Christian Lieven, Siddharth Chauhan, Ali Kaafarani and Nikolaus Sonnenschein wrote the software memote. Moritz E. Beber carried out the assessment of publicly available models. Wout van Helvoirt and Jon O. Vik alpha-tested memote and provided early ideas for the memote report interface.

Christian Lieven drafted all parts of the manuscript except for the paragraph about SBML which was drafted by Brett G. Olivier and Frank T. Bergmann. Jon O. Vik helped shape the introduction. Christian Lieven wrote the Supplementary Guide, Moritz E. Beber composed the Supplementary Material, and Beatriz García-Jiménez conducted the Supplementary Clustering Analysis.

Paulo Maia and Paulo Vilaça created a memote plug-in for OptFlux. Jasper J. Koehorst provided a configuration for continuous integration with Gitlab. Filipe Liu, José P. Faria, Janaka N. Edirisinghe and Christopher S. Henry created a memote app for KBase.

Christian Lieven, Moritz E. Beber, Brett G. Olivier, Frank T. Bergmann, Meric Ataman, Parizad Babaei, Jennifer A. Bartell, Lars M. Blank, Siddharth Chauhan, Kevin Correia, Christian Diener, Andreas Dräger, Birgitta E. Ebert, Janaka N. Edirisinghe, José P. Faria, Adam Feist, Georgios Fengos, Ronan M. T. Fleming, Beatriz García-Jiménez, Vassily Hatzimanikatis, Wout van Helvoirt, Christopher S. Henry, Henning Hermjakob, Markus J. Herrgård, Ali Kaafarani, Hyun Uk Kim, Zachary King, Jasper J. Koehorst, Matthias König, Steffen Klamt, Edda Klipp, Meiyappan Lakshmanan, Dong-Yup Lee, Sang Yup Lee, Sunjae Lee, Nathan E. Lewis, Filipe Liu, Hongwu Ma, Daniel Machado, Radhakrishnan Mahadevan, Paulo Maia, Adil Mardinoglu, Gregory L. Medlock,

Jonathan M. Monk, Jens Nielsen, Lars Keld Nielsen, Juan Nogales, Intawat Nookaew, Osbaldo Resendis-Antonio, Bernhard O. Palsson, Jason A. Papin, Kiran R. Patil, Mark Poolman, Nathan D. Price, Anne Richelle, Isabel Rocha, Benjamín J. Sánchez, Peter J. Schaap, Rahuman S. Malik Sheriff, Saeed Shoaie, Nikolaus Sonnenschein, Bas Teusink, Paulo Vilaça, Jon Olav Vik, Judith A.H. Wodke, Joana C. Xavier, Qianqian Yuan, Maksim Zakhartsev, and Cheng Zhang beta-tested memote and provided feedback and suggestions which shaped the software.

All authors read, corrected and approved the manuscript.

Supplementary References

Agren, Rasmus, Liming Liu, Saeed Shoaie, Wanwipa Vongsangnak, Intawat Nookaew, and Jens Nielsen. 2013. "The RAVEN Toolbox and Its Use for Generating a Genome-Scale Metabolic Model for Penicillium Chrysogenum." Edited by Costas D. Maranas. *PLoS Computational Biology* 9 (3). Public Library of Science (PLoS): e1002980. https://doi.org/10.1371/journal.pcbi.1002980.

Aite, Méziane, Marie Chevallier, Clémence Frioux, Camille Trottier, Jeanne Got, Marı'a Paz Cortés, Sebastián N. Mendoza, et al. 2018. "Traceability, Reproducibility and Wiki-Exploration for "à-La-Carte" Reconstructions of Genome-Scale Metabolic Models." Edited by Jens Nielsen. *PLOS Computational Biology* 14 (5). Public Library of Science (PLoS): e1006146. https://doi.org/10.1371/journal.pcbi.1006146.

Arkin, Adam P, Robert W Cottingham, Christopher S Henry, Nomi L Harris, Rick L Stevens, Sergei Maslov, Paramvir Dehal, et al. 2018. "KBase: The United States Department of Energy Systems Biology Knowledgebase." *Nature Biotechnology* 36 (7). Springer Science; Business Media LLC: 566–69. https://doi.org/10.1038/nbt.4163.

Babaei, Parizad, Saeed Shoaie, Boyang Ji, and Jens Nielsen. 2018. "Challenges in Modeling the Human Gut Microbiome." *Nature Biotechnology* 36 (8). Springer Science; Business Media LLC: 682–86. https://doi.org/10.1038/nbt.4213.

Beaulieu-Jones, Brett K, and Casey S Greene. 2017. "Reproducibility of Computational Workflows Is Automated Using Continuous Analysis." *Nat. Biotechnol.* 35 (4): 342–46.

Bergmann, Frank T, Richard Adams, Stuart Moodie, Jonathan Cooper, Mihai Glont, Martin Golebiewski, Michael Hucka, et al. 2014. "COMBINE Archive and OMEX Format: One File to Share All Information to Reproduce a Modeling Project." *BMC Bioinformatics* 15 (1). Springer Science; Business Media LLC. https://doi.org/10.1186/s12859-014-0369-z.

Breiman, Leo. 2001. "Random Forests." *Machine Learning* 45 (1): 5–32. https://doi.org/10.1023/A:1010933404324.

Büchel, Finja, Nicolas Rodriguez, Neil Swainston, Clemens Wrzodek, Tobias Czauderna, Roland Keller, Florian Mittag, et al. 2013. "Path2Models: Large-Scale Generation of Computational Models from Biochemical Pathway Maps." *BMC Systems Biology* 7 (1). Springer Science; Business Media LLC: 116. https://doi.org/10.1186/1752-0509-7-116.

Caspi, Ron, Tomer Altman, Joseph M. Dale, Kate Dreher, Carol A. Fulcher, Fred Gilham, Pallavi Kaipa, et al. 2009. "The MetaCyc Database of Metabolic Pathways and Enzymes and the BioCyc Collection of Pathway/Genome Databases." *Nucleic Acids Research* 38 (suppl_1). Oxford University Press (OUP): D473–D479. https://doi.org/10.1093/nar/gkp875.

Chelliah, Vijayalakshmi, Nick Juty, Ishan Ajmera, Raza Ali, Marine Dumousseau, Mihai Glont, Michael Hucka, et al. 2014. “BioModels: Ten-Year Anniversary.” *Nucleic Acids Research* 43 (D1). Oxford University Press (OUP): D542–D548. https://doi.org/10.1093/nar/gku1181.

Consortium, The UniProt. 2018. “UniProt: The Universal Protein Knowledgebase.” *Nucleic Acids Research* 46 (5). Oxford University Press (OUP): 2699–9. https://doi.org/10.1093/nar/gky092.

Ebrahim, Ali, Eivind Almaas, Eugen Bauer, Aarash Bordbar, Anthony P Burgard, Roger L Chang, Andreas Dräger, et al. 2015. “Do Genome-Scale Models Need Exact Solvers or Clearer Standards?” *Molecular Systems Biology* 11 (10). EMBO: 831. https://doi.org/10.15252/msb.20156157.

Ebrahim, Ali, Joshua A Lerman, Bernhard O Palsson, and Daniel R Hyduke. 2013. “COBRApy: COnstraints-Based Reconstruction and Analysis for Python.” *BMC Syst. Biol.* 7 (August): 74.

Fabregat, Antonio, Steven Jupe, Lisa Matthews, Konstantinos Sidiropoulos, Marc Gillespie, Phani Garapati, Robin Haw, et al. 2017. “The Reactome Pathway Knowledgebase.” *Nucleic Acids Research* 46 (D1). Oxford University Press (OUP): D649–D655. https://doi.org/10.1093/nar/gkx1132.

Gilbert, James, Nicole Pearcy, Rupert Norman, Thomas Millat, Klaus Winzer, John King, Charlie Hodgman, Nigel Minton, and Jamie Twycross. 2019. “Gsmodutils: A Python Based Framework for Test-Driven Genome Scale Metabolic Model Development.” Edited by Russell Schwartz. *Bioinformatics* 35 (18). Oxford University Press (OUP): 3397–3403. https://doi.org/10.1093/bioinformatics/btz088.

Glasner, J. D. 2003. “ASAP, a Systematic Annotation Package for Community Analysis of Genomes.” *Nucleic Acids Research* 31 (1). Oxford University Press (OUP): 147–51. https://doi.org/10.1093/nar/gkg125.

Hackett, S. R., V. R. T. Zanotelli, W. Xu, J. Goya, J. O. Park, D. H. Perlman, P. A. Gibney, D. Botstein, J. D. Storey, and J. D. Rabinowitz. 2016. “Systems-Level Analysis of Mechanisms Regulating Yeast Metabolic Flux.” *Science* 354 (6311). American Association for the Advancement of Science (AAAS): aaf2786–aaf2786. https://doi.org/10.1126/science.aaf2786.

Hastings, Janna, Gareth Owen, Adriano Dekker, Marcus Ennis, Namrata Kale, Venkatesh Muthukrishnan, Steve Turner, Neil Swainston, Pedro Mendes, and Christoph Steinbeck. 2015. “ChEBI in 2016: Improved Services and an Expanding Collection of Metabolites.” *Nucleic Acids Research* 44 (D1). Oxford University Press (OUP): D1214–D1219. https://doi.org/10.1093/nar/gkv1031.

Heavner, Benjamin D, and Nathan D Price. 2015. “Transparency in Metabolic Network Reconstruction Enables Scalable Biological Discovery.” *Curr. Opin. Biotechnol.* 34 (August): 105–9.

Heirendt, Laurent, Sylvain Arreckx, Thomas Pfau, Sebastián N Mendoza, Anne Richelle, Almut Heinken, Hulda S Haraldsdóttir, et al. 2017. “Creation and Analysis of Biochemical Constraint-Based Models: The COBRA Toolbox V3.0,” October.

Henry, Christopher S, Matthew DeJongh, Aaron A Best, Paul M Frybarger, Ben Linsay, and Rick L Stevens. 2010. “High-Throughput Generation, Optimization and Analysis of Genome-Scale Metabolic Models.” *Nature Biotechnology* 28 (9). Springer Nature: 977–82. https://doi.org/10.1038/nbt.1672.

Jeske, Lisa, Sandra Placzek, Ida Schomburg, Antje Chang, and Dietmar Schomburg. 2018. “BRENDA in 2019: A European ELIXIR Core Data Resource.” *Nucleic Acids Research* 47 (D1).

Oxford University Press (OUP): D542–D549. https://doi.org/10.1093/nar/gky1048.

Kanehisa, Minoru. 2019. "Toward Understanding the Origin and Evolution of Cellular Organisms." *Protein Science* 28 (11). Wiley: 1947–51. https://doi.org/10.1002/pro.3715.

Karp, P. D., S. M. Paley, M. Krummenacker, M. Latendresse, J. M. Dale, T. J. Lee, P. Kaipa, et al. 2009. "Pathway Tools Version 13.0: Integrated Software for Pathway/Genome Informatics and Systems Biology." *Briefings in Bioinformatics* 11 (1). Oxford University Press (OUP): 40–79. https://doi.org/10.1093/bib/bbp043.

King, Zachary A., Justin Lu, Andreas Dräger, Philip Miller, Stephen Federowicz, Joshua A. Lerman, Ali Ebrahim, Bernhard O. Palsson, and Nathan E. Lewis. 2015. "BiGG Models: A Platform for Integrating, Standardizing and Sharing Genome-Scale Models." *Nucleic Acids Research* 44 (D1). Oxford University Press (OUP): D515–D522. https://doi.org/10.1093/nar/gkv1049.

Li, Chen, Marco Donizelli, Nicolas Rodriguez, Harish Dharuri, Lukas Endler, Vijayalakshmi Chelliah, Lu Li, et al. 2010. "BioModels Database: An Enhanced, Curated and Annotated Resource for Published Quantitative Kinetic Models." *BMC Systems Biology* 4 (1). Springer Nature: 92. https://doi.org/10.1186/1752-0509-4-92.

Louppe, Gilles, Louis Wehenkel, Antonio Sutera, and Pierre Geurts. 2013. "Understanding Variable Importances in Forests of Randomized Trees." In *Proceedings of the 26th International Conference on Neural Information Processing Systems - Volume 1*, 431–39. NIPS'13. USA: Curran Associates Inc. http://dl.acm.org/citation.cfm?id=2999611.2999660.

Machado, Daniel, Sergej Andrejev, Melanie Tramontano, and Kiran Raosaheb Patil. 2018. "Fast Automated Reconstruction of Genome-Scale Metabolic Models for Microbial Species and Communities." *Nucleic Acids Research* 46 (15). Oxford University Press (OUP): 7542–53. https://doi.org/10.1093/nar/gky537.

Magnúsdóttir, Stefanı'a, Almut Heinken, Ronan M T Fleming, and Ines Thiele. 2018. "Reply to "Challenges in Modeling the Human Gut Microbiome"." *Nature Biotechnology* 36 (8). Springer Science; Business Media LLC: 686–91. https://doi.org/10.1038/nbt.4212.

Magnúsdóttir, Stefanı'a, Almut Heinken, Laura Kutt, Dmitry A Ravcheev, Eugen Bauer, Alberto Noronha, Kacy Greenhalgh, et al. 2016. "Generation of Genome-Scale Metabolic Reconstructions for 773 Members of the Human Gut Microbiota." *Nature Biotechnology* 35 (1). Springer Science; Business Media LLC: 81–89. https://doi.org/10.1038/nbt.3703.

McDonald, A. G., S. Boyce, and K. F. Tipton. 2009. "ExplorEnz: The Primary Source of the IUBMB Enzyme List." *Nucleic Acids Research* 37 (Database). Oxford University Press (OUP): D593–D597. https://doi.org/10.1093/nar/gkn582.

Moretti, Sébastien, Olivier Martin, T. Van Du Tran, Alan Bridge, Anne Morgat, and Marco Pagni. 2015. "MetaNetX/MNXref Reconciliation of Metabolites and Biochemical Reactions to Bring Together Genome-Scale Metabolic Networks." *Nucleic Acids Research* 44 (D1). Oxford University Press (OUP): D523–D526. https://doi.org/10.1093/nar/gkv1117.

Morgat, Anne, Thierry Lombardot, Kristian B. Axelsen, Lucila Aimo, Anne Niknejad, Nevila Hyka-Nouspikel, Elisabeth Coudert, et al. 2016. "Updates in Rhea an Expert Curated Resource of Biochemical Reactions." *Nucleic Acids Research* 45 (D1). Oxford University Press (OUP): D415–D418. https://doi.org/10.1093/nar/gkw990.

Novere, N. Le. 2006. "BioModels Database: A Free, Centralized Database of Curated, Published, Quantitative Kinetic Models of Biochemical and Cellular Systems." *Nucleic Acids Research* 34 (90001). Oxford University Press (OUP): D689–D691. https://doi.org/10.1093/nar/gkj092.

OBrien, Edward J, Joshua A Lerman, Roger L Chang, Daniel R Hyduke, and Bernhard Ø Palsson. 2013. "Genome-Scale Models of Metabolism and Gene Expression Extend and Refine Growth Phenotype Prediction." *Molecular Systems Biology* 9 (1). EMBO: 693. https://doi.org/10.1038/msb.2013.52.

Prasad, T. S. Keshava, R. Goel, K. Kandasamy, S. Keerthikumar, S. Kumar, S. Mathivanan, D. Telikicherla, et al. 2009. "Human Protein Reference Database–2009 Update." *Nucleic Acids Research* 37 (Database). Oxford University Press (OUP): D767–D772. https://doi.org/10.1093/nar/gkn892.

Pujar, Shashikant, Nuala A O'Leary, Catherine M Farrell, Jane E Loveland, Jonathan M Mudge, Craig Wallin, Carlos G Girón, et al. 2017. "Consensus Coding Sequence (CCDS) Database: A Standardized Set of Human and Mouse Protein-Coding Regions Supported by Expert Curation." *Nucleic Acids Research* 46 (D1). Oxford University Press (OUP): D221–D228. https://doi.org/10.1093/nar/gkx1031.

Richa Agarwala, Tanya Barrett, Jeff Beck, Dennis A Benson, Colleen Bollin, Evan Bolton, Devon Bourexis, et al. 2017. "Database Resources of the National Center for Biotechnology Information." *Nucleic Acids Research* 46 (D1). Oxford University Press (OUP): D8–D13. https://doi.org/10.1093/nar/gkx1095.

Rocha, Isabel, Paulo Maia, Pedro Evangelista, Paulo Vilaça, Simão Soares, José P Pinto, Jens Nielsen, Kiran R Patil, Eugénio C Ferreira, and Miguel Rocha. 2010. "OptFlux: An Open-Source Software Platform for in Silico Metabolic Engineering." *BMC Systems Biology* 4 (1). Springer Nature. https://doi.org/10.1186/1752-0509-4-45.

Stein, Stephen, S.R. Heller, and Dmitrii Tchekhovskoi. 2003. "An Open Standard for Chemical Structure Representation: The Iupac Chemical Identifier." *Nimes International Chemical Information Conference Proceedings*, January, 131–43.

Thiel, Christoph, Henrik Cordes, Lorenzo Fabbri, Hélène Eloise Aschmann, Vanessa Baier, Ines Smit, Francis Atkinson, Lars Mathias Blank, and Lars Kuepfer. 2017. "A Comparative Analysis of Drug-Induced Hepatotoxicity in Clinically Relevant Situations." Edited by Donna K. Slonim. *PLOS Computational Biology* 13 (2). Public Library of Science (PLoS): e1005280. https://doi.org/10.1371/journal.pcbi.1005280.

Thorleifsson, S. G., and I. Thiele. 2011. "rBioNet: A COBRA Toolbox Extension for Reconstructing High-Quality Biochemical Networks." *Bioinformatics* 27 (14). Oxford University Press (OUP): 2009–10. https://doi.org/10.1093/bioinformatics/btr308.

Vasilakou, Eleni, Daniel Machado, Axel Theorell, Isabel Rocha, Katharina Nöh, Marco Oldiges, and S Aljoscha Wahl. 2016. "Current State and Challenges for Dynamic Metabolic Modeling." *Current Opinion in Microbiology* 33 (October). Elsevier BV: 97–104. https://doi.org/10.1016/j.mib.2016.07.008.

Waltemath, Dagmar, Richard Adams, Frank T Bergmann, Michael Hucka, Fedor Kolpakov, Andrew K Miller, Ion I Moraru, et al. 2011. "Reproducible Computational Biology Experiments with SED-ML - the Simulation Experiment Description Markup Language." *BMC Systems Biology* 5 (1). Springer Nature: 198. https://doi.org/10.1186/1752-0509-5-198.

Wishart, David S, Yannick Djoumbou Feunang, Ana Marcu, An Chi Guo, Kevin Liang, Rosa Vázquez-Fresno, Tanvir Sajed, et al. 2017. "HMDB 4.0: The Human Metabolome Database for 2018." *Nucleic Acids Research* 46 (D1). Oxford University Press (OUP): D608–D617. https://doi.org/10.1093/nar/gkx1089.

Zhou, Jindan, and Kenneth E. Rudd. 2012. "EcoGene 3.0." *Nucleic Acids Research* 41 (D1). Oxford University Press (OUP): D613–D624. https://doi.org/10.1093/nar/gks1235.
